# Whole-grain consumption and cardiometabolic risk: a meta-analysis of randomized trials

**DOI:** 10.1093/eurheartj/ehag519

**Published:** 2026-09-15

**Authors:** Sina Naghshi, Sevil Kiani, Alireza Ostadrahimi, Ahmad Jayedi, Majid Mobasseri, Sara Arefhosseini, Helda Tutunchi, Dagfinn Aune

**Affiliations:** Student Research Committee, Tabriz University of Medical Sciences, Tabriz, Iran; Student Research Committee, Tabriz University of Medical Sciences, Tabriz, Iran; Nutrition Research Center, Department of Clinical Nutrition, School of Nutrition and Food Science, Tabriz University of Medical Sciences, Tabriz, Iran; Department of Epidemiology and Biostatistics, School of Public Health, Imperial College London, London, UK; Endocrine Research Center, Tabriz University of Medical Sciences, Tabriz, Iran; Student Research Committee, Tabriz University of Medical Sciences, Tabriz, Iran; Endocrine Research Center, Tabriz University of Medical Sciences, Tabriz, Iran; Department of Chemical and Biochemical Engineering, University of Western Ontario, London, ON, Canada; Department of Epidemiology and Biostatistics, School of Public Health, Imperial College London, London, UK; Department of Research, Cancer Registry of Norway, Norwegian Institute of Public Health, Oslo, Norway; Department of Nutrition, Oslo New University College, Oslo, Norway

**Keywords:** Whole grain, Adiposity, Blood pressure, Blood lipids, Glycaemic control

## Abstract

**Background and Aims:**

Evidence suggests that higher whole-grain intake is associated with lower cardiometabolic disease risks, but underlying mechanisms remain unclear.

**Methods:**

Randomized trials evaluating the effects of whole grains on cardiometabolic risk factors were included. Random-effects meta-analyses were used to estimate mean differences (MDs) with 95% confidence intervals (CIs) per 48 g/day increment of whole grains (dry weight; equivalent to ∼90 g or 3 servings of fresh weight).

**Results:**

Eighty-seven trials (101 publications) were included. Each 48 g/day whole-grain intake was associated with reductions in body weight (MD: −.20 kg; 95% CI: −.31 to −.09; *n* = 41), waist circumference (−.22 cm; 95% CI: −.39 to −.06; *n* = 27), total cholesterol (−.10 mmol/L; 95% CI: −.13 to −.07; *n* = 54), and interleukin-6 (MD: −.12 pg/mL; 95% CI: −.23 to −.00; *n* = 12), with high certainty; low-density lipoprotein cholesterol (−.07 mmol/L; 95% CI: −.10 to −.05; *n* = 52), triglycerides (−.04 mmol/L; 95% CI: −.06 to −.02; *n* = 52), fasting plasma glucose (−.03 mmol/L; 95% CI: −.06 to −.00; *n* = 44), and systolic blood pressure (−.82 mmHg; 95% CI: −1.49 to −.15; *n* = 32), with moderate certainty. Non-linear analyses indicated greatest effects at 60–100 g/day for most outcomes, but at higher intakes (up to 180–220 g/day) for a few outcomes.

**Conclusions:**

Whole-grain intake was associated with improvements in multiple cardiometabolic risk factors, with larger effects generally observed at intakes of ∼60–100 g/day. However, the certainty of evidence varied across outcomes, and sparse data at higher intake levels limit firm conclusions regarding dose thresholds.

## Structured Graphical Abstract

**Structured Graphical Abstract:**
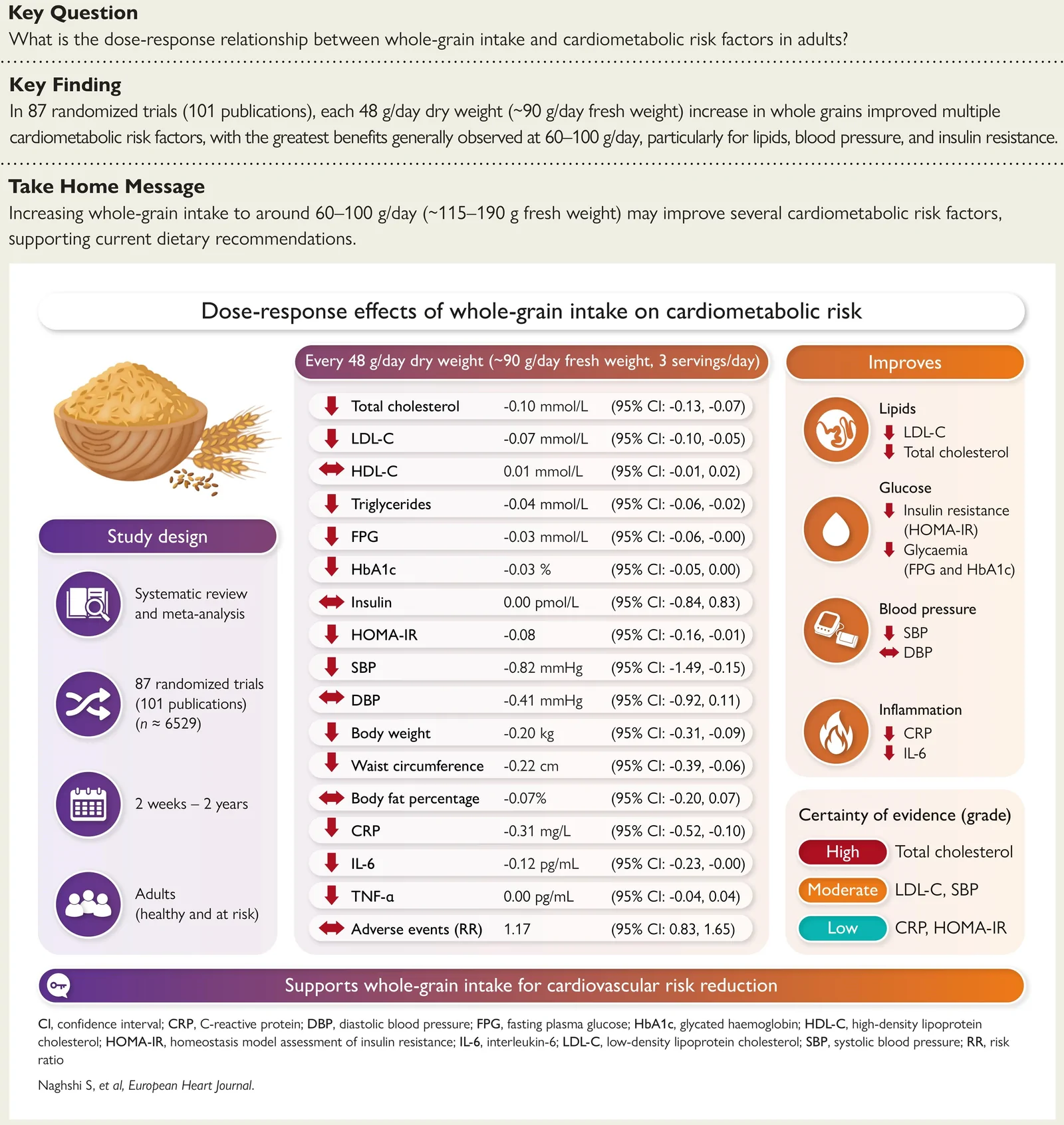
Dose–response meta-analysis of 87 randomized trials showing that each 48 g/day increase in dry-weight whole-grain intake improved several lipid, glycaemic, adiposity, blood-pressure, and inflammatory risk factors, with the largest benefits for most outcomes generally observed at intakes of ∼60–100 g/day.

**See the editorial comment for this article ‘Whole grains and cardiometabolic health: strong evidence, new questions in an ultra-processed world’, by C. Kyrø**  ***et al*****., https://doi.org/10.1093/eurheartj/ehag724.**

## Introduction

Cardiometabolic diseases are a cluster of disorders, including cardiovascular disease (CVD) and type 2 diabetes.^1^ CVD is of great interest because it remains the leading cause of premature mortality and a major cause of health loss globally.^2,3^ According to the World Health Organization (WHO), CVD is expected to account for 22.2 million deaths per year by 2030.^4^

A healthy diet is associated with improvement in cardiometabolic risk factors.^5^ Whole grains, an essential component of healthy dietary patterns,^6^ are rich in cardioprotective compounds that support long-term health.^7,8^ Additionally, whole grains play a central role in feeding the world’s projected population of ∼9.6 billion people by 2050.^9^ Despite these benefits, low intake of whole grains remains a leading dietary risk factor contributing to mortality and disease burden.^10^

Evidence from prospective cohort studies and randomized controlled trials (RCTs) suggests a link between high whole-grain intake and a reduced risk of chronic disease.^11^ Well-designed dose–response meta-analyses of cohort studies have reported that higher whole-grain consumption is linked to a reduced risk of type 2 diabetes, hypertension, coronary heart disease (CHD), CVD, total cancer, and mortality, with the maximum risk reduction for most outcomes observed at around 210–225 g/day, equal to seven to seven and a half servings a day.^11–16^ Although epidemiological studies are relatively consistent in showing benefits of whole grains on cardiometabolic risk, the effect of whole grains on cardiometabolic risk factors, which may mediate associations with hard outcomes, has been less clear in RCTs. For example, two meta-analyses of RCTs found no significant effect of increased whole-grain intake on body weight measures,^17,18^ while another meta-analysis only found small effects on blood lipids.^19^ Thus, clarification of the impact of whole grains on a wide range of cardiometabolic risk factors could fill this important knowledge gap.

Previous meta-analyses of whole-grain RCTs^11,17–27^ have focused primarily on traditional pairwise comparisons between intervention and control groups and ignored the prescribed amount of whole grain. Furthermore, none of them reported effect estimates with consideration of minimally important difference (MID) thresholds, which provide an appropriate clinical interpretation of changes on a numerical scale.^28^ Previous meta-analyses also have other limitations, including the omission of several eligible studies^17,21,23,24,29^ and the inclusion of multiple publications from the same study.^22,25,29^ Other shortcomings include the use of standardized mean differences (SMD) to combine continuous data, which is difficult to interpret,^17,24^ the occurrence of unit of analysis error in studies with multiple intervention groups,^25^ the omission of patient-important outcomes like quality of life and adverse events,^17–27^ and the lack of use of a valid approach to assess the credibility of the observed subgroup effects.^17–27^ Therefore, we conducted a systematic review and dose–response meta-analysis of RCTs on the effects of whole-grain consumption on cardiovascular risk factors in adults. We particularly wanted to clarify the strength and shape of the dose–response relationship between whole-grain consumption and cardiovascular risk factors. The results of the present study, together with the evidence already provided by dose–response meta-analyses of prospective cohort studies, may provide stronger evidence for food-based dietary recommendations.

## Methods

This meta-analysis followed the recommendations in the Cochrane Handbook for Systematic Reviews of Interventions^30^ and reported findings according to the Preferred Reporting Items for Systematic reviews and Meta-Analyses (PRISMA) guidelines.^31^ The review protocol was registered on PROSPERO (registration number CRD42024565726).^32^

### Literature search

A comprehensive search of PubMed, Scopus, Web of Science, CENTRAL (Cochrane Central Register of Controlled Trials), and Google Scholar was conducted until 1 November 2024 (see Supplementary data online, *Table S1*). Studies were included if they were: (i) RCTs (either parallel or crossover design) investigating the effect of whole grain (mixed or individually) consumption with or without caloric restriction; (ii) compared a diet rich in whole grain with low whole-grain diets, usual or conventional diets, routine care, or lifestyle and behavioural recommendations; (iii) conducted in adults aged ≥18 years, excluding pregnant women and individuals with critical conditions such as cancer; (iv) had a minimum intervention duration of 2 weeks; and (v) assessed one of study outcomes including measures of glycaemic control, blood lipids, adiposity, blood pressure, inflammation markers, quality of life, and adverse events. Detailed information on systematic search and criteria applied for excluding studies is provided in Supplementary text  *S1*.

### Definition of eligible interventions

While there are various definitions for whole-grain foods,^33,34^ for the purposes of the present study, a grain was deemed whole if it contained all three parts of the original kernel, including bran, germ, and endosperm.

### Outcomes

The primary outcomes were body weight (kg), low-density lipoprotein cholesterol (LDL-C) (mmol/L), fasting plasma glucose (FPG) (mmol/L), and adverse events. Secondary outcomes included waist circumference (cm), body fat percentage, systolic blood pressure (SBP) (mmHg), diastolic blood pressure (DBP) (mmHg), glycated haemoglobin (HbA_1c_) (%), fasting insulin (pmol/L), homeostasis model assessment of insulin resistance (HOMA-IR), high-density lipoprotein cholesterol (HDL-C) (mmol/L), total cholesterol (mmol/L), triglycerides (mmol/L), C-reactive protein (CRP) (mg/L), interleukin-6 (IL-6) (pg/mL), tumour necrosis factor alpha (TNF-α) (pg/mL), and health-related quality of life (HRQoL).

### Data extraction and risk of bias assessment

Three investigators (S.N., S.K., and S.A.) independently reviewed and extracted relevant data from included studies. We assessed risk of bias of the trials using version 2 of the Cochrane risk of bias tool.^35^ Detailed information on data extraction and risk of bias assessment is provided in Supplementary Text  *S2* and Supplementary data online, *Table S2*. When at least 10 trials were available, subgroup analyses were conducted. The Instrument to assess the Credibility of Effect Modification Analyses (ICEMAN) was used to determine the credibility of the observed subgroup effects.^36^

### Data analyses

For pairwise meta-analyses, continuous outcomes were expressed as mean differences (MD) with 95% confidence intervals (CIs), while for adverse events, we calculated measures of relative (risk ratio) and absolute (risk difference) effects, together with their 95% CIs, using the number of events and non-events across study arms. Absolute risk difference was calculated from the overall risk ratio and baseline risk using the formula: absolute risk difference = baseline risk × (risk ratio − 1). Pooled estimates were calculated using random-effects DerSimonian–Laird meta-analysis when the number of studies was >5; for outcomes with ≤5 studies, pooled estimates were generated using random-effects restricted maximum likelihood (REML) models with the Knapp–Hartung correction. Heterogeneity between studies was evaluated with *I*^2^ and *τ*^2^ statistics. To assess the robustness of the findings, sensitivity analyses were performed. Publication bias was investigated by inspection of contour-enhanced funnel plots^37^ and formal testing using the Egger’s test.^38,39^ We also used the method described by Crippa and Orsini^40^ for linear and non-linear dose–response analyses. Non-linearity and the overall dose–response association were assessed using Wald-type tests. Analyses were conducted using STATA software, version 17. Detailed information on data analysis and protocol amendment is provided in Supplementary Text  *S3* and Supplementary data online, *Tables S3–5*.

### Grading the evidence

We used the Grading of Recommendations Assessment, Development, and Evaluation (GRADE) approach to assess the overall certainty of the evidence and produce profiles in which evidence was graded as high, moderate, low, or very low certainty.^41,42^ We adapted a recently published GRADE minimally contextualized approach to rate imprecision based on MID thresholds.^43,44^ Accordingly, we considered whether the point estimate of effect size was greater than, less than, or equal to the MID, and whether the 95% CIs overlapped with that threshold. Point estimates larger than the MID thresholds were considered important, and those smaller than or equal to the MID were considered clinically irrelevant/unimportant. A detailed description of the criteria used for GRADE certainty rating is presented in Supplementary Text  *S4*.

## Results

In total, 101 publications (87 studies) were included in the current systematic review and meta-analysis (see Supplementary data online, *Figure S1*). Of these articles, several were published on the same populations^45–70^; however, because they reported data on different types of cardiovascular risk factors, they were not considered duplicates and were included in the analysis. The list of studies excluded through full-text assessment, with reasons for exclusions, is provided in Supplementary data online, *Table S6*. The main characteristics of included studies are summarized in Supplementary Text  *S5* and Supplementary data online, *Tables S7–8*.

### Primary outcomes (pairwise meta-analyses)

*Table 1* and Supplementary data online, *Figures S2–6* present the summary estimates for the primary outcomes comparing whole grain interventions (with varying daily doses across studies, typically ranging from ∼12–213 g/day dry weight) with their respective control groups (which included low-whole-grain diets, usual diet, routine care, and refined grain comparators). A random-effects pairwise meta-analysis yielded an important reduction, greater than the MID threshold, in LDL-C (MD: −.13 mmol/L; 95% CI: −.17 to −.08; *I*^2^ = 57.9%; *n* = 69 trials and 5932 participants; moderate certainty evidence), as well as clinically irrelevant reductions in body weight (MD: −.51 kg; 95% CI: −.72 to −.30; *I*^2^ = 40.4%; *n* = 60 trials and 4390 participants; high certainty evidence) and FPG (MD: −.07 mmol/L; 95% CI: −.13 to −.02; *I*^2^ = 69.3%; *n* = 62 trials and 4728 participants; moderate certainty evidence). The findings of the adverse events, with low certainty evidence, are provided in Supplementary Text  *S6* and Supplementary data online, *Table S9*.

**Table 1:** Summary of the effects of whole-grain consumption on primary and secondary outcomes

		Pairwise meta-analysis				Dose–response meta-analysis (per 48 g/day dry weight)	GRADE certainty	Minimally important difference
Outcomes	Trials/participants	Mean difference (95% CI)^a^	*I* ^2^ (%) (95% CI)^b^	Tau^2^	*P*-value^c^			
Primary outcomes								
LDL-C (mmol/L)	69/5932	−.13 (−.17 to −.08)	57.9 (35.7–70.3)	.0170	<.001	−.07 (−.10 to −.05)	Moderate	.10 mmol/L
Body weight (kg)	60/4390	−.51 (−.72 to −.30)	40.4 (6.00–58.9)	.2110	.001	−.20 (−.31 to −.09)	High	2.5 kg
FPG (mmol/L)	62/4728	−.07 (−.13 to −.02)	69.3 (51.3–78.8)	.0268	<.001	−.03 (−.06 to .00)	Moderate	1.6 mmol/L
Adverse events	7/742	RR: 1.53 (.72 to 3.22)	.00 (.00–41.9)	<.001	.67	1.17 (.83–1.65)	Low	-
	7/742	RD: .01 (−.01 to .06)	-	-	-	-		RD .02 (2 more per 100)
Secondary outcomes								
Waist circumference (cm)	38/3207	−.60 (−.98 to −.22)	54.8 (20.3–71.0)	.6826	<.001	−.22 (−.39 to −.06)	High	2 cm
Body fat percentage	29/2682	−.22 (−.47 to .02)	41.1 (.00–63.6)	.1657	.01	−.07 (−.20 to .07)	Moderate	2%
Insulin (pmol/L)	42/3143	−.37 (−2.19 to 1.46)	12.8 (.00–41.2)	4.6976	.24	−.00 (−.84 to .83)	Moderate	5 pmol/L
HOMA-IR	30/2486	−.24 (−.39 to −.09)	66.4 (29.4–80.4)	.1026	<.001	−.08 (−.16 to −.01)	Low	.05
HbA_1c_ (%)	24/1908	−.05 (−.10 to −.01)	58.1 (1.70–76.9)	.0057	<.001	−.03 (−.05 to .00)	Moderate	.5%
Triglycerides (mmol/L)	70/5745	−.05 (−.09 to −.02)	48.2 (19.3–63.9)	.0079	<.001	−.04 (−.06 to −.02)	Moderate	.09 mmol
Total cholesterol (mmol/L)	73/5907	−.17 (−.22 to −.12)	71.5 (28.4–84.8)	.0267	<.001	−.10 (−.13 to −.07)	High	.26 mmol
HDL-C (mmol/L)	71/5754	.01 (−.01 to .03)	81.9 (69.4–88.1)	.0064	<.001	.01 (−.01 to .02)	Moderate	.10 mmol
SBP (mmHg)	46/3609	−1.50 (−2.62 to −.38)	62.5 (36.6–75.3)	8.2369	<.001	−.82 (−1.49 to −.15)	Moderate	2 mmHg
DBP (mmHg)	45/3522	−.82 (−1.59 to −.05)	65.4 (41.1–77.2)	4.0162	<.001	−.41 (−.92 to .11)	Moderate	2 mmHg
CRP (mg/L)	31/2509	−.38 (−.55 to −.21)	83.1 (.00–93.9)	0.1023	<0.001	−.31 (−.52 to −.10)	Low	0.5 mg/L
IL-6 (pg/mL)	20/1219	−.22 (−.44 to −.01)	67.3 (7.50–83.5)	.1155	<.001	−.12 (−.23 to −.004)	High	1 pg/mL
TNF-α (pg/mL)	14/697	−.04 (−0.16 to .08)	64.4 (.00–84.4)	.0196	<.001	−.00 (−.04 to .04)	Low	.9 pg/mL
HRQoL	1/39	−.90 (−11.7 to 9.93)	-	-	-	-	Very low	7 points

RR, risk ratio; RD, risk difference; CI, confidence interval; GRADE, Grading of Recommendations Assessment, Development, and Evaluation; SBP, systolic blood pressure; DBP, diastolic blood pressure; LDL-C, low-density lipoprotein cholesterol; HDL-C, high-density lipoprotein cholesterol; FPG, fasting plasma glucose; HbA_1c_, glycated haemoglobin; HOMA-IR, homeostatic model assessment for insulin resistance; CRP, C-reactive protein; IL-6, interleukin-6; TNF-α, tumour necrosis factor-alpha; HRQoL, health-related quality of life.

^a^Obtained from random effects model.

^b^Inconsistency—percentage of variation across studies due to heterogeneity.

^c^Obtained from *Q* test.

### Secondary outcomes (pairwise meta-analyses)

*Table 1* and Supplementary data online, *Figures S7–20* present the effect of whole-grain consumption on the secondary outcomes. Whole-grain interventions (with daily dry-weight doses ranging from ∼12 to 213 g/day across studies) were compared against control conditions that included low whole-grain diets, usual or routine care, and refined-grain comparators. In terms of adiposity measurements, whole grain consumption led to a clinically irrelevant change in waist circumference (MD: −.60 cm; 95% CI: −.98 to −.22; *I*^2^ = 54.8%; *n* = 38 trials and 3207 participants; high certainty evidence) compared with the control group. Reduction in body fat percentage was clinically irrelevant, and the 95% CI included the null effect (moderate certainty evidence).

In terms of insulin sensitivity and glycaemic control, whole-grain consumption was associated with a clinically irrelevant reduction in HbA_1c_ levels (MD: −.05%; 95% CI: −.10 to −.01; *I*^2^ = 58.1%; *n* = 24 trials and 1908 participants; moderate certainty evidence). Change in fasting insulin levels was clinically irrelevant, and the 95% CI included the null effect (moderate certainty evidence).

Combining data from studies that reported information on blood lipids yielded clinically irrelevant reductions in triglycerides (MD: −.05 mmol/L; 95% CI: −.09 to −.02; *I*^2^ = 48.2%; *n* = 70 trials and 5745 participants; moderate certainty evidence) and total cholesterol (MD: −.17 mmol/L; 95% CI: −.22 to −.12; *I*^2^ = 71.5%; *n* = 73 trials and 5907 participants; high certainty evidence) compared with the control group. Improvement in HDL-C was clinically irrelevant, and the 95% CI overlapped with a null effect (moderate certainty evidence).

When the results of studies that reported blood pressure and inflammatory data were pooled, clinically irrelevant changes were found for SBP (MD: −1.50 mmHg; 95% CI: −2.62 to −.38; *I*^2^ = 62.5%; *n* = 46 trials and 3609 participants; moderate certainty evidence), DBP (MD: −.82 mmHg; 95% CI: −1.59 to −.05; *I*^2^ = 65.4%; *n* = 45 trials and 3522 participants; moderate certainty evidence), and IL-6 (MD: −.22 pg/mL; 95% CI: −.44 to −.01; *I*^2^ = 67.3%; *n* = 20 trials and 1219 participants; high certainty evidence). The findings for HOMA-IR, CRP, TNF-α, and HRQoL, with low or very low certainty evidence, are provided in the Supplementary Text  *S6*.

### Publication bias, sensitivity, and subgroup analyses (pairwise meta-analyses)

For all outcomes, no publication bias was identified from the contour-enhanced funnel plots (see Supplementary data online, *Figures S21–37*). Findings from the sensitivity analyses indicated that the results of DBP, HbA_1c_, IL-6, and body fat percentage were not robust and sensitive to the influence of single studies (see Supplementary data online, *Figures S38–54*). Detailed findings of publication bias, sensitivity, and subgroup analyses are presented in Supplementary Text  *S7* and Supplementary data online, *Tables S10–16*. Using the ICEMAN tool, the credibility of the observed subgroup effects was rated as low (see Supplementary data online, *Table S17*).

### Dose–response meta-analyses

*Figure 1*, *Table 1* and Supplementary data online, *Figures S55–71* present linear dose–response analyses. Every 48 g/day increment in whole grain consumption in the form of dry weight, which is equal to 90 g fresh weight or 3 servings of whole grains, was associated with a reduction in LDL-C, body weight, FPG, waist circumference, HOMA-IR, triglycerides, total cholesterol, SBP, CRP, and IL-6. For other outcomes, the 95% CIs overlapped null effects.

**Figure 1:**
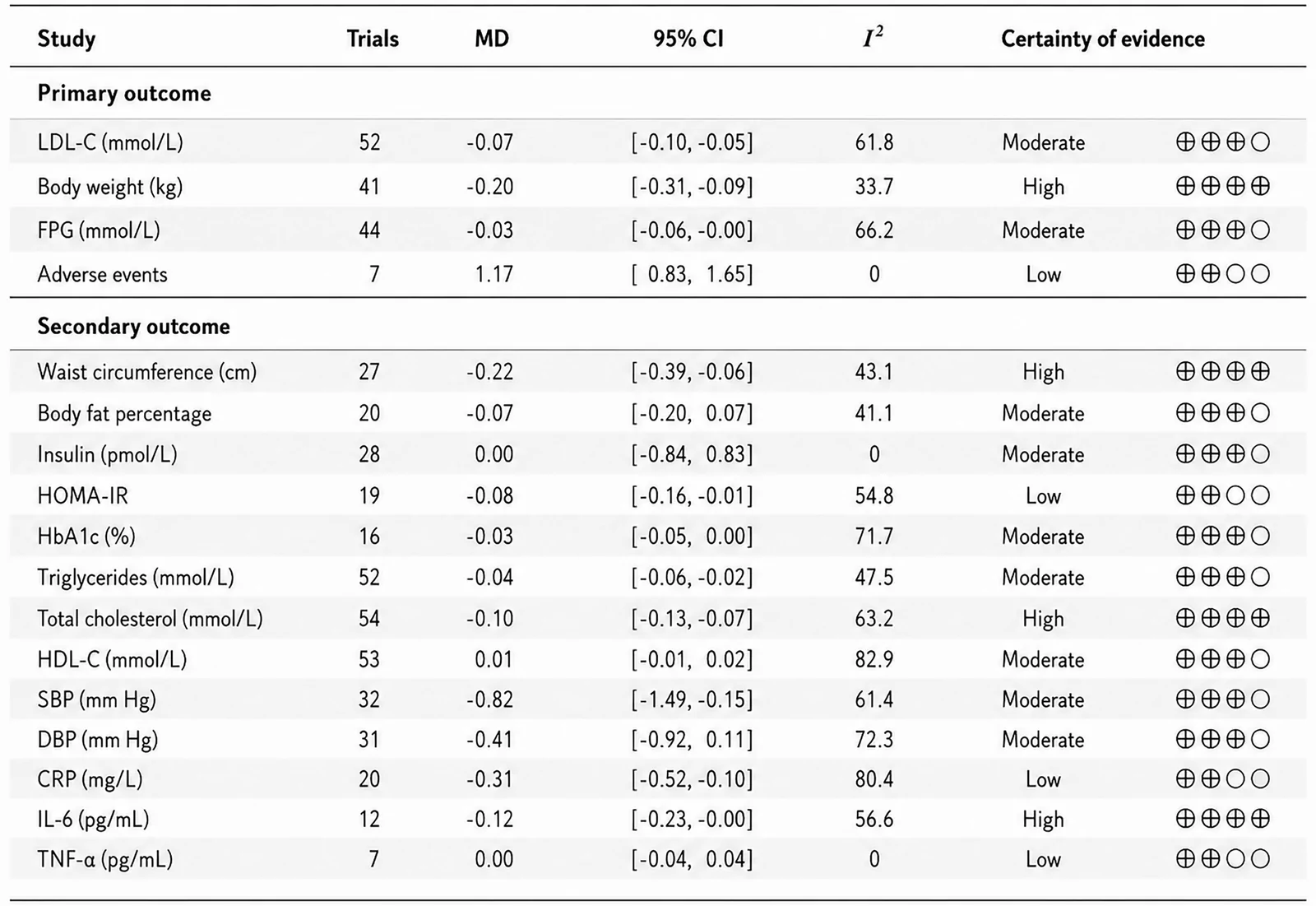
Summary plot for cardiometabolic outcomes based on a 48 g increase in whole-grain consumption in adults aged ≥18 years. MD, mean difference; CI, confidence interval; LDL-C, low-density lipoprotein cholesterol; FPG, fasting plasma glucose; HDL-C, high-density lipoprotein cholesterol; HOMA-IR, homeostasis model assessment of insulin resistance; HbA_1c_, glycated haemoglobin; SBP, systolic blood pressure; DBP, diastolic blood pressure; CRP, C-reactive protein; IL-6, interleukin-6; TNF-α, tumour necrosis factor alpha. All values are presented as MD (95% CI), except for adverse events, which are reported as a risk ratio

Supplementary data online, *Tables S18–21*, and *Figures 2–6* present the results of the non-linear dose–response meta-analyses. For adiposity measures, changes in effect estimates remained clinically irrelevant and unimportant across the full spectrum of daily whole grain intake, ranging from 0 to 220 g/day dry weight (*Figure 2* and Supplementary data online, *Table S18*). For measures of FPG, insulin, and HbA1c, the magnitude of the effects remained clinically irrelevant within the full range of intake (*Figure 3* and Supplementary data online, *Table S19*).

**Figure 2:**
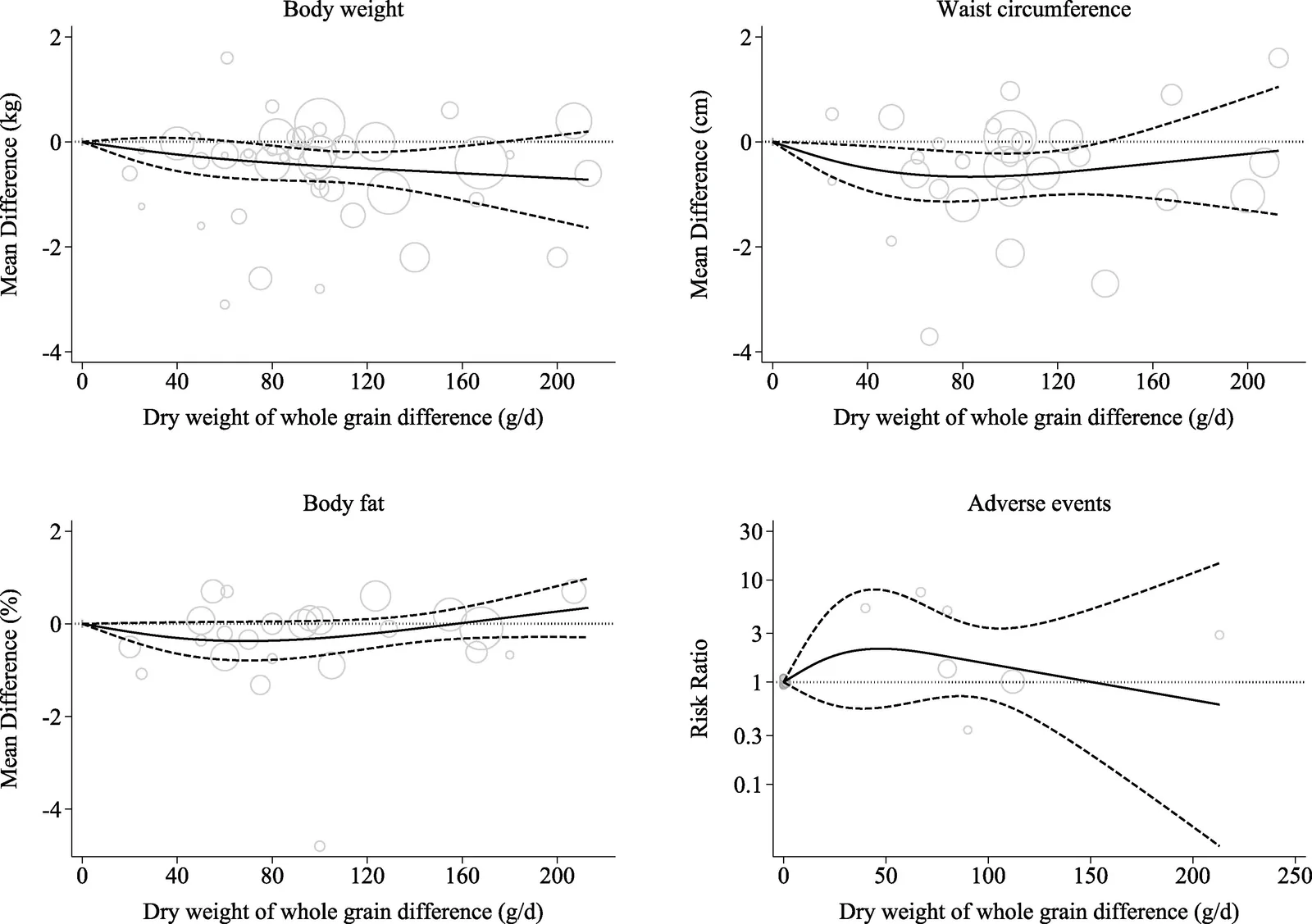
Dose–response effects of whole grain consumption on weight, waist circumference, body fat percentage, and adverse events in adults aged ≥18 years

**Figure 3:**
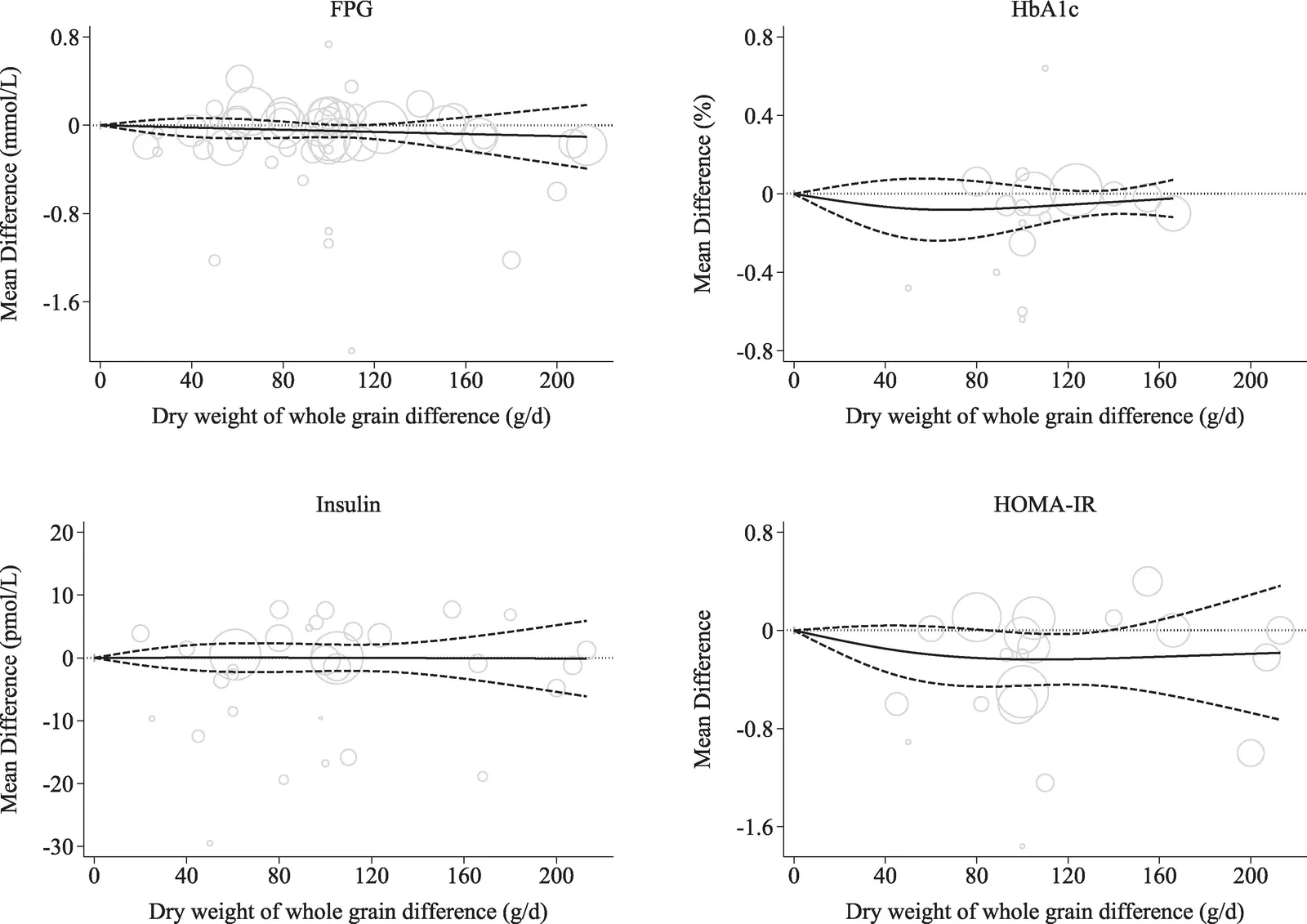
Dose–response effects of whole grain consumption on FPG, HbA_1c_, insulin, and HOMA-IR in adults aged ≥18 years. FPG, fasting plasma glucose; HbA_1c_, glycated haemoglobin; HOMA-IR, homeostasis model assessment of insulin resistance

For blood lipids, there was evidence of a non-linear effect, with the greatest reductions observed at around 80 g/day for total cholesterol (MD_80 g/day_: −.21 mmol/L, 95% CI: −.27 to −.15), LDL-C (MD_80 g/day_: −.16 mmol/L, 95% CI: −.22 to −.10), and triglycerides (MD_80 g/day_: −.08 mmol/L, 95% CI: −.13 to −.04), surpassing the threshold set as MID for these outcomes (*Figure 4* and Supplementary data online, *Table S20*). For HDL-C, a slight reduction in serum levels was seen up to 60 g/day followed by an upward curve thereafter, with the greatest increases observed at around 220 g/day (MD_220 g/day_: .19 mmol/L, 95% CI: .05–.34), surpassing the MID threshold (*Figure 4* and Supplementary data online, *Table S20*).

**Figure 4:**
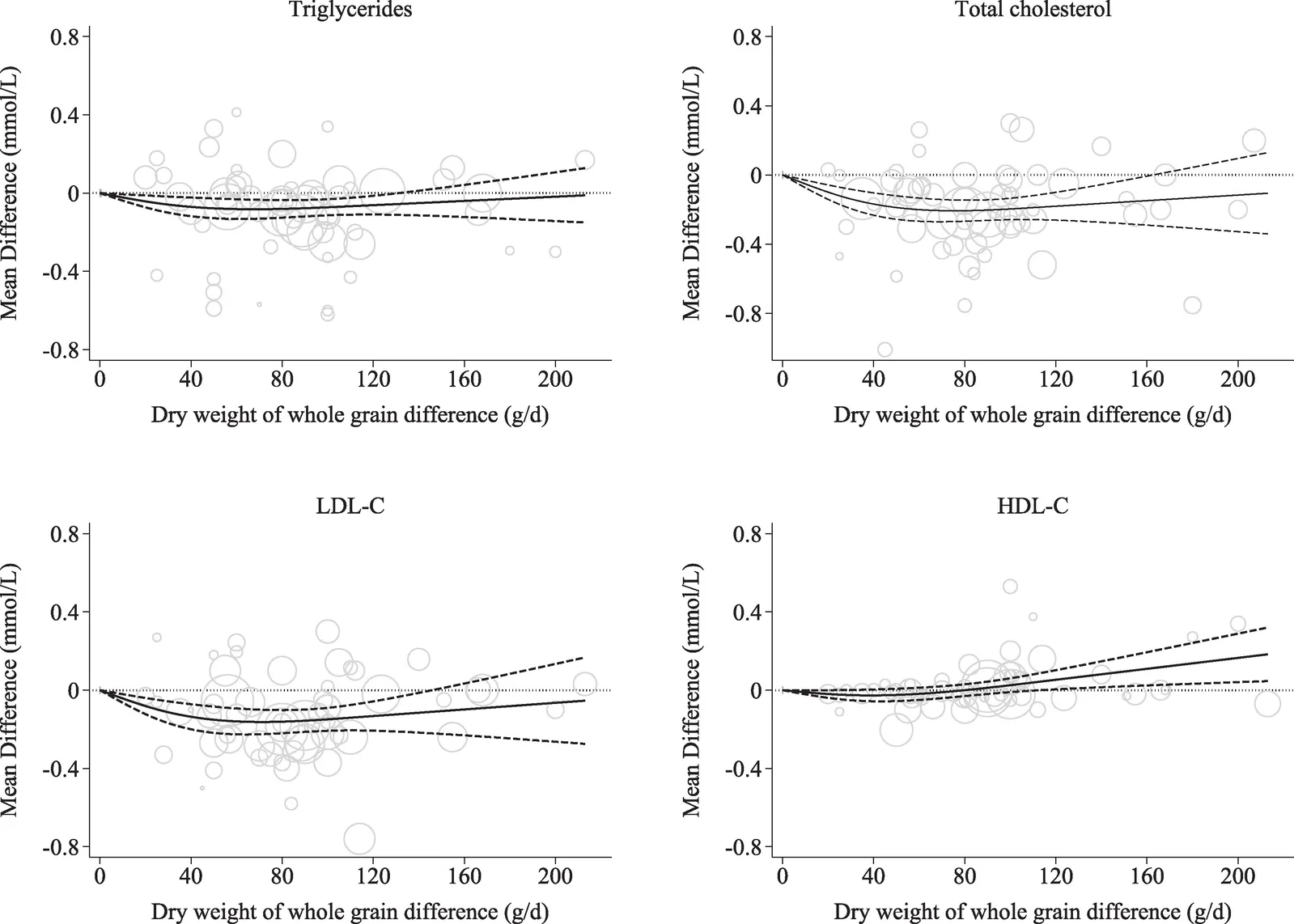
Dose–response effects of whole grain consumption on triglycerides, total cholesterol, LDL-C, and HDL-C in adults aged ≥18 years. LDL-C, low-density lipoprotein cholesterol; HDL-C, high-density lipoprotein cholesterol

When considering blood pressure and inflammatory biomarkers, the magnitude of the effects remained clinically irrelevant across the full range of whole grain intake for DBP, TNF-α, and IL-6 (*Figures 5* and *6*, and Supplementary data online, *Table S21*). However, the greatest reductions in SBP were observed at around 60 g/day (MD: −2.44 mmHg, 95% CI: −4.29 to −.58) and 80 g/day (MD: −2.35 mmHg, 95% CI: −4.09 to −.62), exceeding the MID threshold (*Figures 5* and Supplementary data online, *Table S21*). There was evidence of a departure from linearity for CRP, with the greatest reductions observed at doses of 60 g/day (MD_60 g/day_: −.58 mg/L, 95% CI: −.86 to −.30), followed by an upward trend at higher doses (*Figure 6* and Supplementary data online, *Table S21*).

**Figure 5:**
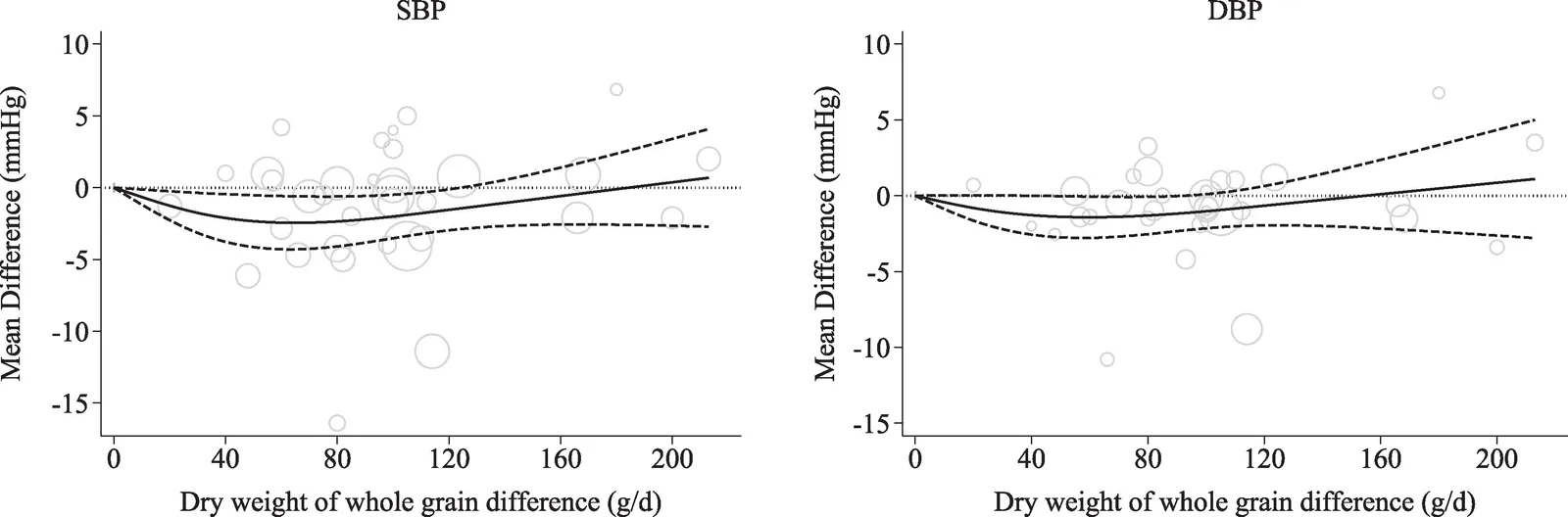
Dose–response effects of whole grain consumption on SBP and DBP in adults aged ≥18 years. SBP, systolic blood pressure; DBP, diastolic blood pressure

**Figure 6:**
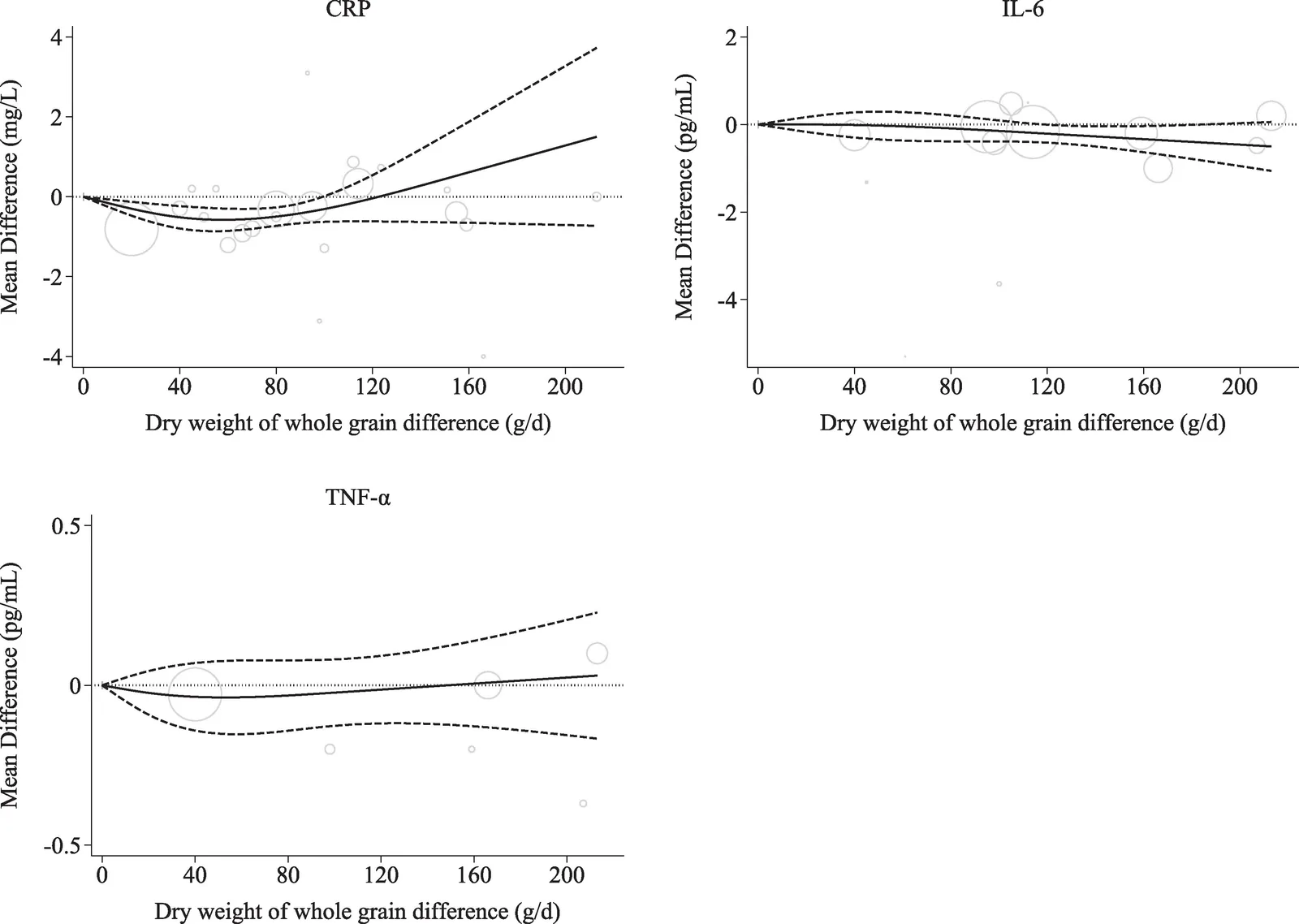
Dose–response effects of whole grain consumption on CRP, IL-6, and TNF-α in adults aged ≥18 years. CRP, C-reactive protein; IL-6, interleukin-6; TNF-α, tumour necrosis factor alpha

For cardiometabolic outcomes, we performed sensitivity analyses using alternative model specifications and evaluated model fit. The restricted cubic spline model with three knots provided a better fit for all outcomes than the other models based on Akaike information criteria (see Supplementary data online, *Tables S22–25* and Supplementary data online, *Figures S72–101*).

### Grading the evidence

The evidence was graded as high for body weight, waist circumference, total cholesterol and IL-6. The evidence was graded as moderate for triglycerides, LDL-C, HDL-C, FPG, HbA_1c_, insulin, SBP, DBP, and body fat percentage. The evidence was graded as low for adverse events, HOMA-IR, CRP, and TNF-α. The evidence was graded as very low for HRQoL (see Supplementary data online, *Table S26*).

## Discussion

In this systematic review and meta-analysis, whole-grain intake was associated with improvements in several cardiometabolic risk factors. Pairwise and linear dose–response analyses consistently showed reductions in LDL-C, body weight, FPG, total cholesterol, triglycerides, blood pressure, waist circumference, HbA_1c_, HOMA-IR, CRP, and IL-6. In non-linear analyses, benefits generally emerged at intakes of 30–40 g/day (dry weight), with the greatest improvements observed at around 60 g/day for CRP and SBP, 80 g/day for lipids, and 220 g/day for HDL-C, exceeding MID thresholds. For other outcomes, changes remained clinically small across the intake range, although the greatest reductions were observed at 80 g/day for waist circumference, 180 g/day for body weight, and 200 g/day for IL-6 (*Structured Graphical Abstract*).

### Findings in relation to the literature

Our findings are broadly consistent with previous meta-analyses, although some earlier reviews reported no association with cardiometabolic risk factors, inflammatory markers, glycaemic control, or adiposity. Compared with prior reviews, our analysis included substantially more trials, evaluated multiple outcomes simultaneously, incorporated both linear and non-linear dose–response analyses using one-stage mixed-effects models, and accounted for differences in whole-grain dose ranges and subtypes, which may explain the clearer associations observed.

In the current study, the greatest reductions in total cholesterol, LDL-C, and triglycerides were seen at 80 g/day of whole grain intake, while the greatest increase for HDL-C was observed at 220 g/day, although the number of data points at the high range of intakes was limited. These changes exceeded predefined MID thresholds for each outcome. Observational epidemiologic studies consistently associate lower LDL-C and higher HDL-C plasma concentrations with reduced CHD risk.^71^ The greatest reductions observed for LDL-C and triglycerides at moderate intake levels of whole grains (80 g/day) compared with the much higher threshold needed to increase HDL-C (220 g/day) suggest that the cardioprotective effects of whole grains may arise primarily from their LDL-C- and triglyceride-lowering effects rather than their HDL-C-raising potential. However, unlike non-linear dose–response analysis, our pairwise analysis did not yield a clinically relevant effect for HDL-C levels. It seems that findings from the dose–response analyses are more reliable than those from the pairwise comparison, in which estimates might encounter misclassification bias because of the varying ranges of whole-grain doses across studies.

In the non-linear dose–response meta-analysis, improvements in triglycerides, LDL-C, HDL-C, CRP, and SBP exceeded respective MID thresholds. For the remaining variables (total cholesterol, FPG, body weight, waist circumference, body fat percentage, DBP, HbA_1c_, fasting insulin, IL-6, and TNF-α), changes across the full spectrum of daily whole grain intake were unimportant and clinically irrelevant. When interpreting these findings, it should be considered that the median duration of follow-up was 8 weeks, which may be insufficient to observe larger effects. It is possible that there may be cumulative and larger effects of long-term adherence to diets high in whole grains, and this might be one explanation for the stronger inverse associations observed between whole grain intakes and reduced risk of CVD in observational studies, than what is predicted based on shorter-term randomized trials on intermediary cardiovascular risk factors. Another possibility is that the effects of whole grains on multiple cardiovascular risk factors are additive and may collectively reduce cardiovascular risk to a larger extent than when considering each risk factor on its own. This is consistent with evidence from patients with diabetes, where simultaneously addressing multiple cardiovascular risk factors (glycaemic, blood pressure, and lipid control) provides greater and long-lasting benefits for preventing or slowing CVD compared to managing individual risk factors.^72,73^

Our findings are in line with and provide support for the results from cohort studies, which report lower risks of all-cause mortality, CVD, CHD, type 2 diabetes, and hypertension incidence with higher intakes of whole grains.^12–16^ The consistency between trial and prospective cohort results, together with observed dose–response relationships, suggests that the effect on risk factors is likely causal and not a consequence of confounding variables. However, discrepancies exist regarding the most effective intake levels. Meta-analyses of cohort studies observed risk reductions up to intakes of 210–225 g/day of whole-grain foods (∼112–120 g/day dry weight) for most outcomes,^12–14^ whereas our analysis found that the greatest reductions for most CVD risk factors were around 60–100 g/day whole-grain intake in the form of dry weight (equivalent to an average of 115–190 g fresh weight or 4–6 servings daily of whole grains). These differences may be partially attributable to variations in study populations (e.g. high-risk vs general populations), exposure sources (e.g. single vs mixed whole-grain sources), outcomes (e.g. surrogate biomarkers vs mortality/morbidity), follow-up durations (typically decades in observational cohort studies vs a few weeks in RCTs), and sample sizes (typically from ten to a few hundred in randomized trials to tens to hundreds of thousands in cohort studies). In addition, we had relatively few data points at higher levels of whole grain intakes, resulting in wider CIs at the very high range of intakes (>140 g/day) than at medium levels of intakes (80–100 g/day). This imprecision reflects the limited number of trials that tested high doses, rather than evidence of no effect. Therefore, the limited data and wide CIs observed at higher intake levels suggest that this figure should be interpreted as a range where benefits may plateau, rather than as a definitive target. However, for HDL-C, there were indications of greater benefits at even higher intakes. Additional large, randomized trials could clarify the impact of very high intakes of whole grains across cardiometabolic risk factors.

Whole-grain intake was not associated with increased mild-to-moderate adverse events. However, adverse events were inconsistently reported, and most trials were not designed or powered to systematically capture minor or subjective symptoms, particularly given the short follow-up periods and limited sample sizes.

Substantial heterogeneity was observed in some analyses, likely reflecting differences in whole-grain dose and type, intervention duration, participant characteristics, background diet, comparators, and chance. Heterogeneity was reduced in sensitivity analyses after exclusion of outlier studies. Variability in the sodium content of commercially prepared whole-grain products may also have contributed to inconsistent blood pressure findings. Associations were attenuated in analyses accounting for baseline whole-grain intake, although these findings may reflect limited power, measurement error, or other study differences rather than true effect modification.

Subgroup analyses suggested some heterogeneity across study design, participant characteristics, and whole-grain type, although most findings were inconsistent, based on a few studies, and should be considered hypothesis-generating. Subgroup analyses suggested stronger effects on HOMA-IR in some lower risk-of-bias trials, in men, and on FPG among individuals with type 2 diabetes, whereas medication use did not materially influence findings in the available analyses. Effects also appeared to vary by grain type, with oats showing stronger lipid-lowering effects, brown rice improving lipids and inflammation, and rye reducing adiposity, consistent with differences in fibre composition and food structure. Trials without caloric restriction or run-in periods generally reported larger effects, suggesting possible independent metabolic effects beyond weight loss and potential influence of baseline dietary patterns. Longer-duration trials (≥16 weeks) showed smaller and clinically irrelevant changes for some outcomes, possibly reflecting limited statistical power, declining adherence, spillover effects, or compensatory behaviours over time.

Interpretation of these findings should consider that higher whole-grain intake may reflect dietary substitution, as increased whole-grain consumption replaces other foods within a relatively stable energy intake. Trials comparing whole grains with refined grain equivalents provide the most robust evidence by isolating substitution effects, whereas comparisons with usual or healthy diets are more susceptible to confounding from concurrent dietary changes. Consequently, the magnitude and interpretation of associations may depend partly on the comparator and substitution pattern.

### Strengths and limitations

Strengths of this study include the large number of RCTs and cardiometabolic outcomes evaluated, detailed linear and non-linear dose–response analyses, and assessment of clinically relevant effects using MID thresholds. The generally consistent findings across populations with different baseline risks also support the broader generalizability of the results.

Several limitations should be acknowledged. Although we assessed the dose–response effect of whole grain intake on cardiometabolic risk factors, studies may also differ by type of whole grain. Therefore, the metabolic effects of whole grains may depend on both the amount and type consumed. Since the included studies may be susceptible to a range of experimental biases, the overall quality of the body of evidence was rated as moderate for most outcomes. For example, most studies handled missing data in the analysis by including only participants who adhered to the trial protocol. However, this approach can introduce bias and is less robust than an intention-to-treat analysis.^74^ Another point was the lack of blinding among the participants or staff delivering the intervention to the hypotheses of the study. Limited reporting of background whole-grain intake restricted assessment of the influence of habitual diet, and the exclusion, in the included trials, of participants using medications affecting body weight or inflammation limited subgroup analyses. Finally, substantial variation in whole-grain doses across studies precluded assessment of time-response relationships.

### Implications

Current dietary recommendations for whole-grain intake vary substantially across organizations and countries, ranging from general advice to increase intake to quantitative targets between 48 and 150 g/day (dry weight). These differences likely reflect variation in whole grain definitions, intake assessment methods, interpretation of evidence, and local dietary practices. Our findings, based largely on trials quantifying intake as dry weight using dietary records or recalls, suggest that intakes of ∼60–100 g/day dry weight (∼115–190 g/day fresh weight or 4–6 servings) may be associated with improvements in multiple cardiovascular risk factors. This estimate is broadly consistent with existing guidelines while providing a quantitative dose–response perspective that may help inform future guidance. However, these targets should be interpreted as evidence-based benchmarks rather than universal prescriptions, as feasibility and implementation will depend on baseline intake, food availability, cultural dietary patterns, and the degree of refined grain substitution.

The observed improvements in LDL-C, triglycerides, SBP, HOMA-IR, and CRP are also clinically relevant when interpreted alongside evidence from pharmacological and epidemiological studies linking reductions in these risk factors with lower CVD risk. If additive, the combined effects of whole-grain intake on multiple cardiometabolic pathways could plausibly translate into meaningful reductions in CHD and CVD risk, particularly when sustained long-term, consistent with evidence from cohort studies.

## Conclusion

Findings from this meta-analysis of randomized trials suggest that higher whole-grain intake is associated with improvements in multiple cardiometabolic risk factors. Our results suggest increasing intake to around 60–100 g/day (dry weight; ∼115–190 g fresh weight or ∼4–6 servings), although additional benefits at higher intake cannot be ruled out. Dose–response relationships differed across outcomes, with the greatest improvements generally observed at around 60–100 g/day for multiple cardiometabolic risk factors, whereas HDL-C showed potential further benefits at higher intakes. However, sparse data and wide CIs at higher intake levels limit certainty. Given the moderate-to-low certainty of evidence and limited number of low-risk-of-bias trials, these findings should be interpreted cautiously. Further well-designed, longer-term trials are needed to more clearly define the intake range that may maximize health benefits.

## Supplementary Material

ehag519_Supplementary_Data

## Declarations

### Data Availability

Data are available upon reasonable request.

### Pre-registered Clinical Trial Number

The review protocol was registered on PROSPERO (registration number CRD42024565726).

## References

[1] Tahir UA, Gerszten RE. Molecular biomarkers for cardiometabolic disease: risk assessment in young individuals. Circ Res 2023;132:1663–73. 10.1161/circresaha.123.322000 PMC10320574 37289904

[2] GBD 2019 Diseases and Injuries Collaborators . Global burden of 369 diseases and injuries in 204 countries and territories, 1990-2019: a systematic analysis for the Global Burden of Disease Study 2019. Lancet 2020;396:1204–22. 10.1016/s0140-6736(20)30925-9 PMC7567026 33069326

[3] Mensah GA, Roth GA, Fuster V. The global burden of cardiovascular diseases and risk factors: 2020 and beyond. J Am Coll Cardiol 2019;74:2529–32. 10.1016/j.jacc.2019.10.009 31727292

[4] World Health O . Global status Report on Noncommunicable Diseases 2014. Geneva: World Health Organization, 2014, p 280. Summary in French, Spanish, Arabic, Russian and Chinese published as technical document WHO/NMH/NVI/15.1.

[5] Mozaffarian D . Dietary and policy priorities for cardiovascular disease, diabetes, and obesity: a comprehensive review. Circulation 2016;133:187–225. 10.1161/circulationaha.115.018585 PMC4814348 26746178

[6] Satija A, Bhupathiraju SN, Spiegelman D, Chiuve SE, Manson JE, Willett W, et al Healthful and unhealthful plant-based diets and the risk of coronary heart disease in U.S. Adults. J Am Coll Cardiol 2017;70:411–22. 10.1016/j.jacc.2017.05.047 PMC5555375 28728684

[7] Fardet A . New hypotheses for the health-protective mechanisms of whole-grain cereals: what is beyond fibre? Nutr Res Rev 2010;23:65–134. 10.1017/s0954422410000041 20565994

[8] Hemler EC, Hu FB. Plant-based diets for personal, population, and planetary health. Adv Nutr 2019;10:S275–83. 10.1093/advances/nmy117 PMC6855934 31728495

[9] Rockström J, Thilsted SH, Willett WC, Gordon LJ, Herrero M, Hicks CC, et al The EAT-Lancet Commission on healthy, sustainable, and just food systems. Lancet 2025;406:1625–700. 10.1016/s0140-6736(25)01201-2 41046857

[10] GBD 2023 Nordic and Baltic Diet Collaborators . The burden of dietary risk factors in the Nordic and Baltic countries: a systematic analysis for the Global Burden of Disease Study 2023. Lancet Reg Health Eur 2026;61:. 10.1016/j.lanepe.2025.101543 PMC12686888 41376666

[11] Reynolds A, Mann J, Cummings J, Winter N, Mete E, Te Morenga L. Carbohydrate quality and human health: a series of systematic reviews and meta-analyses. Lancet 2019;393:434–45. 10.1016/s0140-6736(18)31809-9 30638909

[12] Aune D, Keum N, Giovannucci E, Fadnes LT, Boffetta P, Greenwood DC, et al Whole grain consumption and risk of cardiovascular disease, cancer, and all cause and cause specific mortality: systematic review and dose-response meta-analysis of prospective studies. BMJ 2016;353:i2716. 10.1136/bmj.i2716 PMC4908315 27301975

[13] Zong G, Gao A, Hu FB, Sun Q. Whole grain intake and mortality from all causes, cardiovascular disease, and cancer: a meta-analysis of prospective cohort studies. Circulation 2016;133:2370–80. 10.1161/circulationaha.115.021101 PMC4910651 27297341

[14] Chen GC, Tong X, Xu JY, Han SF, Wan ZX, Qin JB, et al Whole-grain intake and total, cardiovascular, and cancer mortality: a systematic review and meta-analysis of prospective studies. Am J Clin Nutr 2016;104:164–72. 10.3945/ajcn.115.122432 27225432

[15] Aune D, Norat T, Romundstad P, Vatten LJ. Whole grain and refined grain consumption and the risk of type 2 diabetes: a systematic review and dose-response meta-analysis of cohort studies. Eur J Epidemiol 2013;28:845–58. 10.1007/s10654-013-9852-5 24158434

[16] Aune D, Metoudi M, Sadler I, Kassam S. Whole grain and refined grain consumption and the risk of hypertension: a systematic review and meta-analysis of prospective studies. Sci Rep 2025;15:21447. 10.1038/s41598-025-05197-5 PMC12214891 40592949

[17] Maki KC, Palacios OM, Koecher K, Sawicki CM, Livingston KA, Bell M, et al The relationship between whole grain intake and body weight: results of meta-analyses of observational studies and randomized controlled trials. Nutrients 2019;11:1245. 10.3390/nu11061245 PMC6627338 31159235

[18] Sadeghi O, Sadeghian M, Rahmani S, Maleki V, Larijani B, Esmaillzadeh A. Whole-grain consumption does not affect obesity measures: an updated systematic review and meta-analysis of randomized clinical trials. Adv Nutr 2020;11:280–92. 10.1093/advances/nmz076 PMC7073321 31390462

[19] Hollænder PLB, Ross AB, Kristensen M. Whole-grain and blood lipid changes in apparently healthy adults: a systematic review and meta-analysis of randomized controlled studies. Am J Clin Nutr 2015;102:556–72. 10.3945/ajcn.115.109165 26269373

[20] Rahmani S, Sadeghi O, Sadeghian M, Sadeghi N, Larijani B, Esmaillzadeh A. The effect of whole-grain intake on biomarkers of subclinical inflammation: a comprehensive meta-analysis of randomized controlled trials. Adv Nutr 2020;11:52–65. 10.1093/advances/nmz063 PMC7442343 31301131

[21] Xu Y, Wan Q, Feng J, Du L, Li K, Zhou Y. Whole grain diet reduces systemic inflammation: a meta-analysis of 9 randomized trials. Medicine (Baltimore) 2018;97:e12995. 10.1097/md.0000000000012995 PMC6221555 30412134

[22] Li Z, Yan H, Chen L, Wang Y, Liang J, Feng X, et al Effects of whole grain intake on glycemic control: a meta-analysis of randomized controlled trials. J Diabetes Investig 2022;13:1814–24. 10.1111/jdi.13866 PMC9623515 35678196

[23] Marventano S, Vetrani C, Vitale M, Godos J, Riccardi G, Grosso G. Whole grain intake and glycaemic control in healthy subjects: a systematic review and meta-analysis of randomized controlled trials. Nutrients 2017;9:769. 10.3390/nu9070769 PMC5537883 28753929

[24] Marshall S, Petocz P, Duve E, Abbott K, Cassettari T, Blumfield M, et al The effect of replacing refined grains with whole grains on cardiovascular risk factors: a systematic review and meta-analysis of randomized controlled trials with GRADE clinical recommendation. J Acad Nutr Diet 2020;120:1859–83.e31. 10.1016/j.jand.2020.06.021 32933853

[25] Li S, Zong A, An R, Wang H, Liu L, Liu J, et al Effects of whole grain intake on glycemic traits: a systematic review and meta-analysis of randomized controlled trials. Crit Rev Food Sci Nutr 2023;63:4351–70. 10.1080/10408398.2021.2001429 34793262

[26] Pol K, Christensen R, Bartels EM, Raben A, Tetens I, Kristensen M. Whole grain and body weight changes in apparently healthy adults: a systematic review and meta-analysis of randomized controlled studies. Am J Clin Nutr 2013;98:872–84. 10.3945/ajcn.113.064659 23945718

[27] Xu D, Fu L, Pan D, Lu Y, Yang C, Wang Y, et al Role of whole grain consumption in glycaemic control of diabetic patients: a systematic review and meta-analysis of randomized controlled trials. Nutrients 2021;14:109. 10.3390/nu14010109 PMC8746707 35010985

[28] Guyatt GH, Juniper EF, Walter SD, Griffith LE, Goldstein RS. Interpreting treatment effects in randomised trials. BMJ 1998;316:690–3. 10.1136/bmj.316.7132.690 PMC1112685 9522799

[29] Hajihashemi P, Haghighatdoost F. Effects of whole-grain consumption on selected biomarkers of systematic inflammation: a systematic review and meta-analysis of randomized controlled trials. J Am Coll Nutr 2019;38:275–85. 10.1080/07315724.2018.1490935 30183552

[30] Higgins JP, Thomas J, Chandler J, Cumpston M, Li T, Page MJ, et al Cochrane Handbook for Systematic Reviews of Interventions. Chichester, West Sussex: John Wiley & Sons, 2019.

[31] Page MJ, McKenzie JE, Bossuyt PM, Boutron I, Hoffmann TC, Mulrow CD, et al The PRISMA 2020 statement: an updated guideline for reporting systematic reviews. BMJ 2021;372:n71. 10.1136/bmj.n71 PMC8005924 33782057

[32] Naghshi S, Kiani S, Ostadrahimi A, Jayedi A, Mobaseri M, Arefhosseini S, et al Effect of whole grain consumption on cardiometabolic risk factors: a protocol for a systematic review and dose-response meta-analysis of randomised controlled trials. PROSPERO. 2024. https://www.crd.york.ac.uk/PROSPERO/view/CRD42024565726.

[33] Korczak R, Marquart L, Slavin JL, Ringling K, Chu Y, O'Shea M, et al Thinking critically about whole-grain definitions: summary report of an interdisciplinary roundtable discussion at the 2015 Whole Grains Summit. Am J Clin Nutr 2016;104:1508–14. 10.3945/ajcn.115.126672 27806974

[34] Ferruzzi MG, Jonnalagadda SS, Liu S, Marquart L, McKeown N, Reicks M, et al Developing a standard definition of whole-grain foods for dietary recommendations: summary report of a multidisciplinary expert roundtable discussion. Adv Nutr 2014;5:164–76. 10.3945/an.113.005223 PMC3951798 24618757

[35] Sterne JAC, Savović J, Page MJ, Elbers RG, Blencowe NS, Boutron I, et al Rob 2: a revised tool for assessing risk of bias in randomised trials. BMJ 2019;366:l4898. 10.1136/bmj.l4898 31462531

[36] Schandelmaier S, Briel M, Varadhan R, Schmid CH, Devasenapathy N, Hayward RA, et al Development of the Instrument to assess the Credibility of Effect Modification Analyses (ICEMAN) in randomized controlled trials and meta-analyses. CMAJ 2020;192:E901–6. 10.1503/cmaj.200077 PMC7829020 32778601

[37] Peters JL, Sutton AJ, Jones DR, Abrams KR, Rushton L. Contour-enhanced meta-analysis funnel plots help distinguish publication bias from other causes of asymmetry. J Clin Epidemiol 2008;61:991–6. 10.1016/j.jclinepi.2007.11.010 18538991

[38] Egger M, Davey Smith G, Schneider M, Minder C. Bias in meta-analysis detected by a simple, graphical test. BMJ 1997;315:629–34. 10.1136/bmj.315.7109.629 PMC2127453 9310563

[39] Sterne JA, Sutton AJ, Ioannidis JP, Terrin N, Jones DR, Lau J, et al Recommendations for examining and interpreting funnel plot asymmetry in meta-analyses of randomised controlled trials. BMJ 2011;343:d4002. 10.1136/bmj.d4002 21784880

[40] Crippa A, Orsini N. Dose-response meta-analysis of differences in means. BMC Med Res Methodol 2016;16:91. 10.1186/s12874-016-0189-0 PMC4971698 27485429

[41] Schünemann H, Brożek J, Guyatt G, Oxman A. GRADE Handbook for Grading Quality of Evidence and Strength of Recommendations. The GRADE Working Group, 2013. guidelinedevelopment.org/handbook.

[42] Guyatt GH, Oxman AD, Vist GE, Kunz R, Falck-Ytter Y, Alonso-Coello P, et al GRADE: an emerging consensus on rating quality of evidence and strength of recommendations. BMJ 2008;336:924–6. 10.1136/bmj.39489.470347.AD PMC2335261 18436948

[43] Hultcrantz M, Rind D, Akl EA, Treweek S, Mustafa RA, Iorio A, et al The GRADE Working Group clarifies the construct of certainty of evidence. J Clin Epidemiol 2017;87:4–13. 10.1016/j.jclinepi.2017.05.006 PMC6542664 28529184

[44] Zeng L, Brignardello-Petersen R, Hultcrantz M, Siemieniuk RAC, Santesso N, Traversy G, et al GRADE guidelines 32: GRADE offers guidance on choosing targets of GRADE certainty of evidence ratings. J Clin Epidemiol 2021;137:163–75. 10.1016/j.jclinepi.2021.03.026 33857619

[45] Xu D, Feng M, Chu Y, Wang S, Shete V, Tuohy KM, et al The prebiotic effects of oats on blood lipids, gut microbiota, and short-chain fatty acids in mildly hypercholesterolemic subjects compared with rice: a randomized, controlled trial. Front Immunol 2021;12:787797. 10.3389/fimmu.2021.787797 PMC8697019 34956218

[46] Xu D, Wang S, Feng M, Shete V, Chu Y, Kamil A, et al Serum metabolomics reveals underlying mechanisms of cholesterol-lowering effects of oat consumption: a randomized controlled trial in a mildly hypercholesterolemic population. Mol Nutr Food Res 2021;65:e2001059. 10.1002/mnfr.202001059 33793078

[47] Wan W, Jiang X, Zhao R, Cai F, Wu F, Hu Y, et al Branched chain amino acids, new target of germinated brown rice against type 2 diabetes mellitus: a randomized controlled trial. Mol Nutr Food Res 2023;67:e2200481. 10.1002/mnfr.202200481 36564930

[48] Ding Q, Ren J, Zhou Y, Bai Z, Yan J, Na G, et al Whole grain germinated brown rice regulates intestinal immune homeostasis and gastrointestinal hormones in type 2 diabetic patients-a randomized control trial. Food Funct 2022;13:8274–82. 10.1039/d2fo00477a 35833889

[49] de Mello VDF, Schwab U, Kolehmainen M, Koenig W, Siloaho M, Poutanen K, et al A diet high in fatty fish, bilberries and wholegrain products improves markers of endothelial function and inflammation in individuals with impaired glucose metabolism in a randomised controlled trial: the Sysdimet study. Diabetologia 2011;54:2755–67. 10.1007/s00125-011-2285-3 21870174

[50] Lankinen M, Schwab U, Kolehmainen M, Paananen J, Poutanen K, Mykkänen H, et al Whole grain products, fish and bilberries alter glucose and lipid metabolism in a randomized, controlled trial: the Sysdimet study. PLoS One 2011;6:e22646. 10.1371/journal.pone.0022646 PMC3161986 21901116

[51] Lankinen M, Kolehmainen M, Jääskeläinen T, Paananen J, Joukamo L, Kangas AJ, et al Effects of whole grain, fish and bilberries on serum metabolic profile and lipid transfer protein activities: a randomized trial (Sysdimet). PLoS One 2014;9:e90352. 10.1371/journal.pone.0090352 PMC3938672 24587337

[52] Schutte S, Esser D, Hoevenaars FPM, Hooiveld GJEJ, Priebe MG, Vonk RJ, et al A 12-wk whole-grain wheat intervention protects against hepatic fat: the Graandioos study, a randomized trial in overweight subjects. Am J Clin Nutr 2018;108:1264–74. 10.1093/ajcn/nqy204 30541093

[53] Hoevenaars FPM, Esser D, Schutte S, Priebe MG, Vonk RJ, van den Brink WJ, et al Whole grain wheat consumption affects postprandial inflammatory response in a randomized controlled trial in overweight and obese adults with mild hypercholesterolemia in the Graandioos study. J Nutr 2019;149:2133–44. 10.1093/jn/nxz177 PMC6887734 31504709

[54] Giacco R, Lappi J, Costabile G, Kolehmainen M, Schwab U, Landberg R, et al Effects of rye and whole wheat versus refined cereal foods on metabolic risk factors: a randomised controlled two-centre intervention study. Clin Nutr 2013;32:941–9. 10.1016/j.clnu.2013.01.016 23462537

[55] Giacco R, Costabile G, Della Pepa G, Anniballi G, Griffo E, Mangione A, et al A whole-grain cereal-based diet lowers postprandial plasma insulin and triglyceride levels in individuals with metabolic syndrome. Nutr Metab Cardiovasc Dis 2014;24:837–44. 10.1016/j.numecd.2014.01.007 24598599

[56] Vanegas SM, Meydani M, Barnett JB, Goldin B, Kane A, Rasmussen H, et al Substituting whole grains for refined grains in a 6-wk randomized trial has a modest effect on gut microbiota and immune and inflammatory markers of healthy adults. Am J Clin Nutr 2017;105:635–50. 10.3945/ajcn.116.146928 PMC5320415 28179226

[57] Karl JP, Meydani M, Barnett JB, Vanegas SM, Goldin B, Kane A, et al Substituting whole grains for refined grains in a 6-wk randomized trial favorably affects energy-balance metrics in healthy men and postmenopausal women. Am J Clin Nutr 2017;105:589–99. 10.3945/ajcn.116.139683 PMC5320410 28179223

[58] Ma X, Gu J, Zhang Z, Jing L, Xu M, Dai X, et al Effects of Avena nuda L. on metabolic control and cardiovascular disease risk among Chinese patients with diabetes and meeting metabolic syndrome criteria: secondary analysis of a randomized clinical trial. Eur J Clin Nutr 2013;67:1291–7. 10.1038/ejcn.2013.201 24129363

[59] Li X, Cai X, Ma X, Jing L, Gu J, Bao L, et al Short- and long-term effects of wholegrain oat intake on weight management and glucolipid metabolism in overweight type-2 diabetics: a randomized control trial. Nutrients 2016;8:549. 10.3390/nu8090549 PMC5037534 27618090

[60] MacKay KA, Tucker AJ, Duncan AM, Graham TE, Robinson LE. Whole grain wheat sourdough bread does not affect plasminogen activator inhibitor-1 in adults with normal or impaired carbohydrate metabolism. Nutr Metab Cardiovasc Dis 2012;22:704–11. 10.1016/j.numecd.2010.10.018 21195592

[61] Tucker AJ, Mackay KA, Robinson LE, Graham TE, Bakovic M, Duncan AM. The effect of whole grain wheat sourdough bread consumption on serum lipids in healthy normoglycemic/normoinsulinemic and hyperglycemic/hyperinsulinemic adults depends on presence of the APOE E3/E3 genotype: a randomized controlled trial. Nutr Metab (Lond) 2010;7:37. 10.1186/1743-7075-7-37 PMC2877680 20444273

[62] Juntunen KS, Laaksonen DE, Poutanen KS, Niskanen LK, Mykkänen HM. High-fiber rye bread and insulin secretion and sensitivity in healthy postmenopausal women. Am J Clin Nutr 2003;77:385–91. 10.1093/ajcn/77.2.385 12540398

[63] Moazzami AA, Bondia-Pons I, Hanhineva K, Juntunen K, Antl N, Poutanen K, et al Metabolomics reveals the metabolic shifts following an intervention with rye bread in postmenopausal women—a randomized control trial. Nutr J 2012;11:88. 10.1186/1475-2891-11-88 PMC3493305 23088297

[64] Ampatzoglou A, Atwal KK, Maidens CM, Williams CL, Ross AB, Thielecke F, et al Increased whole grain consumption does not affect blood biochemistry, body composition, or gut microbiology in healthy, low-habitual whole grain consumers. J Nutr 2015;145:215–21. 10.3945/jn.114.202176 25644340

[65] Ampatzoglou A, Williams CL, Atwal KK, Maidens CM, Ross AB, Thielecke F, et al Effects of increased wholegrain consumption on immune and inflammatory markers in healthy low habitual wholegrain consumers. Eur J Nutr 2016;55:183–95. 10.1007/s00394-015-0836-y 25618418

[66] Pavadhgul P, Bumrungpert A, Harjani Y, Kurilich A. Oat porridge consumption alleviates markers of inflammation and oxidative stress in hypercholesterolemic adults. Asia Pac J Clin Nutr 2019;28:260–5. 10.6133/apjcn.201906_28(2).0008 31192555

[67] Thongoun P, Pavadhgul P, Bumrungpert A, Satitvipawee P, Harjani Y, Kurilich A. Effect of oat consumption on lipid profiles in hypercholesterolemic adults. J Med Assoc Thai 2013;96 Suppl 5:S25–32. 24851570

[68] Qiu J, Li Z, Qin Y, Yue Y, Liu Y. Protective effect of tartary buckwheat on renal function in type 2 diabetics: a randomized controlled trial. Ther Clin Risk Manag 2016;12:1721–7. 10.2147/tcrm.s123008 PMC5125721 27920542

[69] Qiu J, Liu Y, Yue Y, Qin Y, Li Z. Dietary tartary buckwheat intake attenuates insulin resistance and improves lipid profiles in patients with type 2 diabetes: a randomized controlled trial. Nutr Res 2016;36:1392–401. 10.1016/j.nutres.2016.11.007 27919453

[70] Ye M, Sun J, Chen Y, Ren Q, Li Z, Zhao Y, et al Oatmeal induced gut microbiota alteration and its relationship with improved lipid profiles: a secondary analysis of a randomized clinical trial. Nutr Metab (Lond) 2020;17:85. 10.1186/s12986-020-00505-4 PMC7542720 33042205

[71] Di Angelantonio E, Sarwar N, Perry P, Kaptoge S, Ray KK, Thompson A, et al Major lipids, apolipoproteins, and risk of vascular disease. JAMA 2009;302:1993–2000. 10.1001/jama.2009.1619 PMC3284229 19903920

[72] Khunti K, Kosiborod M, Ray KK. Legacy benefits of blood glucose, blood pressure and lipid control in individuals with diabetes and cardiovascular disease: time to overcome multifactorial therapeutic inertia? Diabetes Obes Metab 2018;20:1337–41. 10.1111/dom.13243 29405543

[73] American Diabetes Association Professional Practice Committee . 10. Cardiovascular Disease and Risk Management: Standards of Care in Diabetes-2025. Diabetes Care 2025;48:S207–38. 10.2337/dc25-S010 PMC11635050 39651970

[74] Detry MA, Lewis RJ. The intention-to-treat principle: how to assess the true effect of choosing a medical treatment. JAMA 2014;312:85–6. 10.1001/jama.2014.7523 25058221

