# Supplementary material for: Whole-grain consumption and cardiometabolic risk: a meta-analysis of randomized trials
Source: Eur Heart J. 2026 Sep 15;47(42):6235–47. doi: 10.1093/eurheartj/ehag519 (PMC13749457; doi:10.1093/eurheartj/ehag519)
Supplement: Supplementary file 1 [file ehag519_supplementary_data.docx]

**Supplementary Online Content**

[Supplementary Text 1: Detailed information on systematic search and criteria applied for excluding studies	8](#_Toc229069344)

[Supplementary Text 2: Detailed information on data extraction and risk of bias assessment	9](#_Toc229069345)

[Supplementary Text 3: Detailed information on data analysis and protocol amendment	10](#_Toc229069346)

[Supplementary Text 4: Instructions to rate the certainty of evidence using the GRADE approach	13](#_Toc229069347)

[Supplementary Text 5: Detailed information on the characteristics of studies included in the systematic review and meta-analysis	14](#_Toc229069348)

[Supplementary Text 6: Detailed findings on adverse events, HOMA-IR, CRP, TNF-α, and HRQoL with low or very low certainty evidence	16](#_Toc229069349)

[Supplementary Text 7: Detailed findings of publication bias, sensitivity, subgroup, and dose‑response meta‑analyses	17](#_Toc229069350)

[Supplementary Table 1: The terms used to search relevant publications on the effect of whole grains on the cardiometabolic risk factors	19](#_Toc229069351)

[Supplementary Table 2: Conversion factor used to convert primary and secondary outcome units for data extraction	21](#_Toc229069352)

[Supplementary Table 3: Minimal clinically important differences (MID) for outcomes	22](#_Toc229069353)

[Supplementary Table 4: List of rules for data estimation and conversion	23](#_Toc229069354)

[Supplementary Table 5: Description of the ICEMAN domains and how to judge each domain	25](#_Toc229069355)

[Supplementary Table 6: List of publications excluded by full text assessment	27](#_Toc229069356)

[Supplementary Table 7: Characteristics of included studies on the effect of whole grain on the CMRFs in adults aged >18 years	36](#_Toc229069357)

[Supplementary Table 8: Risk‑of‑bias assessment of the included studies according to Version 2 of the Cochrane risk‑of‑bias tool	44](#_Toc229069358)

[Supplementary Table 9: Adverse events reported in the trials on the effect of whole grains on the cardiometabolic risk factors	47](#_Toc229069359)

[Supplementary Table 10: Pairwise sensitivity analyses using different correlation coefficients	50](#_Toc229069360)

[Supplementary Table 11: Sensitivity analyses restricting to trials with a background diet with low whole grain intake.	52](#_Toc229069361)

[Supplementary Table 12: Subgroup analyses on the effect of whole grains on adiposity measures in adults aged ≥18 years (pairwise meta-analyses).	53](#_Toc229069362)

[Supplementary Table 13: Subgroup analyses on the effect of whole grains on glycemic control in adults aged ≥18 years (pairwise meta-analyses).	57](#_Toc229069363)

[Supplementary Table 14: Subgroup analyses on the effect of whole grains on blood lipids in adults aged ≥18 years (pairwise meta-analyses).	62](#_Toc229069364)

[Supplementary Table 15: Subgroup analyses on the effect of whole grains on blood pressure in adults aged ≥18 years (pairwise meta-analyses).	67](#_Toc229069365)

[Supplementary Table 16: Subgroup analyses on the effect of whole grains on inflammatory biomarkers in adults aged ≥18 years (pairwise meta-analyses).	70](#_Toc229069366)

[Supplementary Table 17: Assessment of credibility of subgroup difference based on ICEMAN	74](#_Toc229069367)

[Supplementary Table 18: Summary of the effects of whole grain consumption on adiposity measures obtained from non-linear dose-response analysis	77](#_Toc229069368)

[Supplementary Table 19: Summary of the effects of whole grain consumption on glycemic control obtained from non-linear dose-response analysis	78](#_Toc229069369)

[Supplementary Table 20: Summary of the effects of whole grain consumption on blood lipids obtained from non-linear dose-response analysis	79](#_Toc229069370)

[Supplementary Table 21: Summary of the effects of whole grain consumption on blood pressure and inflammatory factors obtained from non-linear dose-response analysis	80](#_Toc229069371)

[Supplementary Table 22: Goodness-of-fit tests and measures for dose-response meta-analysis of whole grain consumption on adiposity measures and adverse events	81](#_Toc229069372)

[Supplementary Table 23: Goodness-of-fit tests and measures for dose-response meta-analysis of whole grain consumption on glycemic control	82](#_Toc229069373)

[Supplementary Table 24: Goodness-of-fit tests and measures for dose-response meta-analysis of whole grain consumption on blood lipids	83](#_Toc229069374)

[Supplementary Table 25: Goodness-of-fit tests and measures for dose-response meta-analysis of whole grain consumption on blood pressure and inflammatory factors	84](#_Toc229069375)

[Supplementary Table 26: GRADE assessment for primary and secondary outcomes	85](#_Toc229069376)

[Supplementary Figure 1: Flow diagram of study selection	93](#_Toc229069377)

[Supplementary Figure 2: Forest plot for the effect of whole grain consumption on LDL-C concentrations (mmol/L) in adults aged ≥18 years, expressed as mean differences between the intervention and control groups	94](#_Toc229069378)

[Supplementary Figure 3: Forest plot for the effect of whole grain consumption on body weight (kg) in adults aged ≥18 years, expressed as mean differences between the intervention and control groups	95](#_Toc229069379)

[Supplementary Figure 4: Forest plot for the effect of whole grain consumption on FPG concentrations (mmol/L) in adults aged ≥18 years, expressed as mean differences between the intervention and control groups	96](#_Toc229069380)

[Supplementary Figure 5: Forest plot for the effect of whole grain consumption on adverse events in adults aged ≥18 years, expressed as risk ratio.	97](#_Toc229069381)

[Supplementary Figure 6: Forest plot for the effect of whole grain consumption on adverse events in adults aged ≥18 years, expressed as risk difference.	98](#_Toc229069382)

[Supplementary Figure 7: Forest plot for the effect of whole grain consumption on waist circumference (cm) in adults aged ≥18 years, expressed as mean differences between the intervention and control groups	99](#_Toc229069383)

[Supplementary Figure 8: Forest plot for the effect of whole grain consumption on body fat percentage in adults aged ≥18 years, expressed as mean differences between the intervention and control groups	100](#_Toc229069384)

[Supplementary Figure 9: Forest plot for the effect of whole grain consumption on insulin concentrations (pmol/L) in adults aged ≥18 years, expressed as mean differences between the intervention and control groups	101](#_Toc229069385)

[Supplementary Figure 10: Forest plot for the effect of whole grain consumption on HOMA-IR in adults aged ≥18 years, expressed as mean differences between the intervention and control groups	102](#_Toc229069386)

[Supplementary Figure 11: Forest plot for the effect of whole grain consumption on HbA1c (%) in adults aged ≥18 years, expressed as mean differences between the intervention and control groups	103](#_Toc229069387)

[Supplementary Figure 12: Forest plot for the effect of whole grain consumption on triglycerides concentrations (mmol/L) in adults aged ≥18 years, expressed as mean differences between the intervention and control groups	104](#_Toc229069388)

[Supplementary Figure 13: Forest plot for the effect of whole grain consumption on total cholesterol concentrations (mmol/L) in adults aged ≥18 years, expressed as mean differences between the intervention and control groups	105](#_Toc229069389)

[Supplementary Figure 14: Forest plot for the effect of whole grain consumption on HDL-C concentrations (mmol/L) in adults aged ≥18 years, expressed as mean differences between the intervention and control groups	106](#_Toc229069390)

[Supplementary Figure 15: Forest plot for the effect of whole grain consumption on SBP (mmHg) in adults aged ≥18 years, expressed as mean differences between the intervention and control groups	107](#_Toc229069391)

[Supplementary Figure 16: Forest plot for the effect of whole grain consumption on DBP (mmHg) in adults aged ≥18 years, expressed as mean differences between the intervention and control groups	108](#_Toc229069392)

[Supplementary Figure 17: Forest plot for the effect of whole grain consumption on CRP (mg/L) in adults aged ≥18 years, expressed as mean differences between the intervention and control groups	109](#_Toc229069393)

[Supplementary Figure 18: Forest plot for the effect of whole grain consumption on IL-6 (pg/mL) in adults aged ≥18 years, expressed as mean differences between the intervention and control groups	110](#_Toc229069394)

[Supplementary Figure 19: Forest plot for the effect of whole grain consumption on TNF-α (pg/mL) in adults aged ≥18 years, expressed as mean differences between the intervention and control groups	111](#_Toc229069395)

[Supplementary Figure 20: Forest plot for the effect of whole grain consumption on HRQoL in adults aged ≥18 years, expressed as mean differences between the intervention and control groups	112](#_Toc229069396)

[Supplementary Figure 21: Contour-enhanced Funnel plot for LDL-C	113](#_Toc229069397)

[Supplementary Figure 22: Contour-enhanced Funnel plot for body weight	114](#_Toc229069398)

[Supplementary Figure 23: Contour-enhanced Funnel plot for FPG	115](#_Toc229069399)

[Supplementary Figure 24: Contour-enhanced Funnel plot for adverse events	116](#_Toc229069400)

[Supplementary Figure 25: Contour-enhanced Funnel plot for waist circumference	117](#_Toc229069401)

[Supplementary Figure 26: Contour-enhanced Funnel plot for body fat percentage	118](#_Toc229069402)

[Supplementary Figure 27: Contour-enhanced Funnel plot for insulin	119](#_Toc229069403)

[Supplementary Figure 28: Contour-enhanced Funnel plot for HOMA-IR	120](#_Toc229069404)

[Supplementary Figure 29: Contour-enhanced Funnel plot for HbA1c	121](#_Toc229069405)

[Supplementary Figure 30: Contour-enhanced Funnel plot for triglycerides	122](#_Toc229069406)

[Supplementary Figure 31: Contour-enhanced Funnel plot for total cholesterol	123](#_Toc229069407)

[Supplementary Figure 32: Contour-enhanced Funnel plot for HDL-C	124](#_Toc229069408)

[Supplementary Figure 33: Contour-enhanced Funnel plot for SBP	125](#_Toc229069409)

[Supplementary Figure 34: Contour-enhanced Funnel plot for DBP	126](#_Toc229069410)

[Supplementary Figure 35: Contour-enhanced Funnel plot for C-reactive protein	127](#_Toc229069411)

[Supplementary Figure 36: Contour-enhanced Funnel plot for IL-6	128](#_Toc229069412)

[Supplementary Figure 37: Contour-enhanced Funnel plot for TNF-α	129](#_Toc229069413)

[Supplementary Figure 38: Sensitivity analysis for the effect of whole grain consumption on LDL-C (mmol/L)	130](#_Toc229069414)

[Supplementary Figure 39: Sensitivity analysis for the effect of whole grain consumption on body weight (kg)	131](#_Toc229069415)

[Supplementary Figure 40: Sensitivity analysis for the effect of whole grain consumption on FPG concentrations (mmol/L)	132](#_Toc229069416)

[Supplementary Figure 41: Sensitivity analysis for the effect of whole grain consumption on adverse events	133](#_Toc229069417)

[Supplementary Figure 42: Sensitivity analysis for the effect of whole grain consumption on waist circumference (cm)	134](#_Toc229069418)

[Supplementary Figure 43: Sensitivity analysis for the effect of whole grain consumption on body fat percentage	135](#_Toc229069419)

[Supplementary Figure 44: Sensitivity analysis for the effect of whole grain consumption on insulin concentrations (pmol/L)	136](#_Toc229069420)

[Supplementary Figure 45: Sensitivity analysis for the effect of whole grain consumption on HOMA-IR	137](#_Toc229069421)

[Supplementary Figure 46: Sensitivity analysis for the effect of whole grain consumption on HbA1_c_ (%)	138](#_Toc229069422)

[Supplementary Figure 47: Sensitivity analysis for the effect of whole grain consumption on triglycerides concentrations (mmol/L)	139](#_Toc229069423)

[Supplementary Figure 48: Sensitivity analysis for the effect of whole grain consumption on total cholesterol concentrations (mmol/L)	140](#_Toc229069424)

[Supplementary Figure 49: Sensitivity analysis for the effect of whole grain consumption on HDL-C concentrations (mmol/L)	141](#_Toc229069425)

[Supplementary Figure 50: Sensitivity analysis for the effect of whole grain consumption on SBP (mmHg)	142](#_Toc229069426)

[Supplementary Figure 51: Sensitivity analysis for the effect of whole grain consumption on DBP (mmHg)	143](#_Toc229069427)

[Supplementary Figure 52: Sensitivity analysis for the effect of whole grain consumption on CRP (mg/L)	144](#_Toc229069428)

[Supplementary Figure 53: Sensitivity analysis for the effect of whole grain consumption on IL-6 (pg/mL)	145](#_Toc229069429)

[Supplementary Figure 54: Sensitivity analysis for the effect of whole grain consumption on TNF-α (pg/mL)	146](#_Toc229069430)

[Supplementary Figure 55: Forest plot for the LDL-C (mmol/L) based on 48 g increase in whole grain consumption in adults aged ≥18 years	147](#_Toc229069431)

[Supplementary Figure 56: Forest plot for the body weight (kg) based on 48 g increase in whole grain consumption in adults aged ≥18 years	148](#_Toc229069432)

[Supplementary Figure 57: Forest plot for the FPG (mmol/L) based on 48 g increase in whole grain consumption in adults aged ≥18 years	149](#_Toc229069433)

[Supplementary Figure 58: Forest plot for the adverse events based on 48 g increase in whole grain consumption in adults aged ≥18 years	150](#_Toc229069434)

[Supplementary Figure 59: Forest plot for the waist circumference (cm) based on 48 g increase in whole grain consumption in adults aged ≥18 years	151](#_Toc229069435)

[Supplementary Figure 60: Forest plot for the body fat percentage based on 48 g increase in whole grain consumption in adults aged ≥18 years	152](#_Toc229069436)

[Supplementary Figure 61: Forest plot for the insulin (pmol/L) based on 48 g increase in whole grain consumption in adults aged ≥18 years	153](#_Toc229069437)

[Supplementary Figure 62: Forest plot for the HOMA-IR based on 48 g increase in whole grain consumption in adults aged ≥18 years	154](#_Toc229069438)

[Supplementary Figure 63: Forest plot for the HbA1c (%) based on 48 g increase in whole grain consumption in adults aged ≥18 years	155](#_Toc229069439)

[Supplementary Figure 64: Forest plot for the triglycerides (mmol/L) based on 48 g increase in whole grain consumption in adults aged ≥18 years	156](#_Toc229069440)

[Supplementary Figure 65: Forest plot for the total cholesterol (mmol/L) based on 48 g increase in whole grain consumption in adults aged ≥18 years	157](#_Toc229069441)

[Supplementary Figure 66: Forest plot for the HDL-C (mmol/L) based on 48 g increase in whole grain consumption in adults aged ≥18 years	158](#_Toc229069442)

[Supplementary Figure 67: Forest plot for the SBP (mmHg) based on 48 g increase in whole grain consumption in adults aged ≥18 years	159](#_Toc229069443)

[Supplementary Figure 68: Forest plot for the DBP (mmHg) based on 48 g increase in whole grain consumption in adults aged ≥18 years	160](#_Toc229069444)

[Supplementary Figure 69: Forest plot for the CRP (mg/L) based on 48 g increase in whole grain consumption in adults aged ≥18 years	161](#_Toc229069445)

[Supplementary Figure 70: Forest plot for the IL-6 (pg/mL) based on 48 g increase in whole grain consumption in adults aged ≥18 years	162](#_Toc229069446)

[Supplementary Figure 71: Forest plot for the TNF-α (pg/mL) based on 48 g increase in whole grain consumption in adults aged ≥18 years	163](#_Toc229069447)

[Supplementary Figure 72: Dose‑response effects of whole grain consumption using the quadratic model on weight, waist circumference, body fat percentage, and adverse events in adults aged ≥18 years	164](#_Toc229069448)

[Supplementary Figure 73: Dose‑response effects of whole grain consumption using the quadratic model on FPG, HbA1c, insulin, and HOMA-IR in adults aged ≥18 years.	165](#_Toc229069449)

[Supplementary Figure 74: Dose‑response effects of whole grain consumption using the quadratic model triglycerides, total cholesterol, LDL-C, and HDL-C in adults aged ≥18 years.	166](#_Toc229069450)

[Supplementary Figure 75: Dose‑response effects of whole grain consumption using the quadratic model on SBP and DBP in adults aged ≥18 years.	167](#_Toc229069451)

[Supplementary Figure 76: Dose‑response effects of whole grain consumption using the quadratic model on CRP, IL-6, and TNF-α in adults aged ≥18 years	168](#_Toc229069452)

[Supplementary Figure 77: Dose‑response effects of whole grain consumption using the RCS (with four knots) model on weight, waist circumference, body fat percentage, and adverse events in adults aged ≥18 years.	169](#_Toc229069453)

[Supplementary Figure 78: Dose‑response effects of whole grain consumption using the RCS (with four knots) model on FPG, HbA1c, insulin, and HOMA-IR in adults aged ≥18 years	170](#_Toc229069454)

[Supplementary Figure 79: Dose‑response effects of whole grain consumption using the RCS (with four knots) model on triglycerides, total cholesterol, LDL-C, and HDL-C in adults aged ≥18 years	171](#_Toc229069455)

[Supplementary Figure 80: Dose‑response effects of whole grain consumption using the RCS (with four knots) model on SBP and DBP in adults aged ≥18 years	172](#_Toc229069456)

[Supplementary Figure 81: Dose‑response effects of whole grain consumption using the RCS (with four knots) model on CRP, IL-6, and TNF-α in adults aged ≥18 years	173](#_Toc229069457)

[Supplementary Figure 82: Dose‑response effects of whole grain consumption on weight, waist circumference, and body fat percentage in adults aged ≥18 years using linear, RCS, and quadratic models in the same graph	174](#_Toc229069458)

[Supplementary Figure 83: Dose‑response effects of whole grain consumption on FPG, HbA1c, insulin, and HOMA-IR in adults aged ≥18 years using linear, RCS, and quadratic models in the same graph.	175](#_Toc229069459)

[Supplementary Figure 84: Dose‑response effects of whole grain consumption on triglycerides, total cholesterol, LDL-C, and HDL-C in adults aged ≥18 years using linear, RCS, and quadratic models in the same graph	176](#_Toc229069460)

[Supplementary Figure 85: Dose‑response effects of whole grain consumption on SBP and DBP in adults aged ≥18 years using linear, RCS, and quadratic models in the same graph	177](#_Toc229069461)

[Supplementary Figure 86: Dose‑response effects of whole grain consumption on CRP, IL-6, and TNF-α in adults aged ≥18 years using linear, RCS, and quadratic models in the same graph.	178](#_Toc229069462)

[Supplementary Figure 87: Decorrelated residuals versus dose for the quadratic model of whole grain intake on weight, waist circumference, body fat percentage, and adverse events.	179](#_Toc229069463)

[Supplementary Figure 88: Decorrelated residuals versus dose for the quadratic model of whole grain intake on FPG, HbA1c, insulin, and HOMA-IR	180](#_Toc229069464)

[Supplementary Figure 89: Decorrelated residuals versus dose for the quadratic model of whole grain intake on triglycerides, total cholesterol, LDL-C, and HDL-C	181](#_Toc229069465)

[Supplementary Figure 90: Decorrelated residuals versus dose for the quadratic model of whole grain intake on SBP and DBP	182](#_Toc229069466)

[Supplementary Figure 91: Decorrelated residuals versus dose for the quadratic model of whole grain intake on CRP, IL-6, and TNF-α	183](#_Toc229069467)

[Supplementary Figure 92: Decorrelated residuals versus dose for the RCS (with three knots) model of whole grain intake on weight, waist circumference, body fat percentage, and adverse events	184](#_Toc229069467)

[Supplementary Figure 93: Decorrelated residuals versus dose for the RCS (with three knots) model of whole grain intake on FPG, HbA1c, insulin, and HOMA-IR	185](#_Toc229069468)

[Supplementary Figure 94: Decorrelated residuals versus dose for the RCS (with three knots) model of whole grain intake on triglycerides, total cholesterol, LDL-C, and HDL-C	186](#_Toc229069469)

[Supplementary Figure 95: Decorrelated residuals versus dose for the RCS (with three knots) model of whole grain intake on SBP and DBP	187](#_Toc229069470)

[Supplementary Figure 96: Decorrelated residuals versus dose for the RCS (with three knots) model of whole grain intake on CRP, IL-6, and TNF-α	188](#_Toc229069471)

[Supplementary Figure 97: Decorrelated residuals versus dose for the RCS (with four knots) model of whole grain intake on weight, waist circumference, and body fat percentage	189](#_Toc229069472)

[Supplementary Figure 98: Decorrelated residuals versus dose for the RCS (with four knots) model of whole grain intake on FPG, HbA1c, insulin, and HOMA-IR	190](#_Toc229069473)

[Supplementary Figure 99: Decorrelated residuals versus dose for the RCS (with four knots) model of whole grain intake on triglycerides, total cholesterol, LDL-C, and HDL-C	191](#_Toc229069474)

[Supplementary Figure 100: Decorrelated residuals versus dose for the RCS (with four knots) model of whole grain intake on SBP and DBP	192](#_Toc229069475)

[Supplementary Figure 101: Decorrelated residuals versus dose for the RCS (with four knots) model of whole grain intake on CRP, IL-6, and TNF-α	193](#_Toc229069476)

### Supplementary Text 1: Detailed information on systematic search and criteria applied for excluding studies

Two authors (SN and SK) developed the search strategy and performed the systematic search. The full search strategy for each database is provided in **Supplementary Table 1**. Paired reviewers (SN and SK) independently screened the titles, abstracts, and full texts of published articles. Any disagreement between the two authors was resolved by discussion. The full texts of relevant articles and the reference lists of relevant meta-analyses were then reviewed. There were no restrictions on date or publication status. However, due to financial constraints, we limited this systematic review to articles published in English. Our team lacked the resources to professionally translate non‑English articles or subscribe to non‑English databases. This decision was based on budgetary limitations, not on any conflict of interest. We acknowledge that this may have introduced language bias.

We excluded observational studies, quasi-experimental trials, studies that had an active control group (defined as a comparator arm that received a structured, intended-to-be-beneficial dietary intervention other than whole grains (e.g., a diet enriched with fruits, vegetables, legumes, or an intensive “healthy diet” program) that was designed to actively improve health outcomes, rather than serving as a neutral or baseline reference, or as trials that compared the effects of two types of whole grains against each other), crossover studies that lacked a washout period and did not report first-phase intervention data, studies that were in children, adolescents, pregnant women, or individuals with critical conditions (defined as any life‑threatening or severely disabling illness that would be expected to dominate the effects of a dietary intervention or significantly alter metabolic pathways, such as cancer and end‑stage organ disease), and those that did not report sufficient data to estimate change in outcome measures.

Acute feeding trials investigating only postprandial effects were not included, as it was beyond the scope of the current meta-analysis. Studies that investigated the effects of dietary fiber, cereal fiber, or high-fiber diets were excluded if the specific effect of whole-grain foods or diets could not be distinguished. We also excluded studies that compared the effect of whole grains combined with other food groups. Studies that implemented a co-intervention (e.g., dietary interventions and behavioral support) were included if that co-intervention was implemented in both study arms. For studies for which several publications were published, in general, we used the publication with the largest number of participants. Publications that reported on different outcomes from the same study were not considered duplicates and were included in the relevant meta-analyses.

### Supplementary Text 2: Detailed information on data extraction and risk of bias assessment

Extracted data included information on first author, year of publication, RCT registration number, study design, use of run-in period, randomization, blinding, population size and characteristics (age, sex, background diet, medication use, and health status), whole-grain form, dose, and comparator, co-intervention in both study arms (e.g., calorie restriction, behavioral support, etc.), intervention duration, and outcome data. For crossover studies that did not have a washout period, we extracted data from the first period of the intervention only. Numerical data was extracted from the figures using a WebPlotDigitizer. Studies with effect estimates presented as fat mass were converted to body fat percentage using average weight in each group. Measures of total cholesterol, LDL-C, HDL-C, triglycerides, and FPG were expressed as mmol/L, insulin as pmol/L, CRP as mg/L, and IL-6 and TNF-α as pg/mL.

For each study included in the dose‑response meta‑analysis, we extracted the information from each intervention arm as follows: 1) the mean or median daily whole grain consumption (in grams dry weight) during the intervention period – when both were reported, we preferentially used the mean (including the control arm when whole grain intake was reported as a non‑zero value); 2) the mean change from baseline and its standard deviation (taken at the time point immediately following the completion of the whole grain intervention, typically the last follow‑up measurement while participants were still on the assigned diet); we did not use post‑intervention follow‑up measurements taken after participants had returned to their habitual diet; 3) the number of participants contributing to each dose level; 4) when a study presented multiple doses of whole grains (e.g., 30 g/day and 60 g/day), we extracted data for each dose level as separate “treatment arms” in the dose‑response dataset; 5) if a study reported whole grain intake in fresh weight (e.g., cooked rice weight) or whole grain product weight rather than dry weight, we converted the values to dry weight using standard conversion factors (these conversions are described in **Supplementary Text 3**); and 6) if a study reported both post‑intervention and change‑from‑baseline values for the same outcome, we preferentially extracted the change‑from‑baseline data.

When a study was a randomized controlled trial, we treated the control group (lowest whole‑grain intake) as the reference category. We set the dose of the control group to zero, regardless of whether the control group had a reported background whole‑grain intake or consumed no whole grains. For each intervention arm, the dose was calculated as the difference between the reported whole‑grain intake in that arm and the reported intake in the control arm (i.e., intervention dose − control dose). This difference was then used as the dose value for that intervention arm in the dose‑response analysis. The corresponding mean outcome change from baseline was extracted for each arm.

Included trials were independently assessed for risk of bias by three investigators (SN, SK, and SA) using version 2 of the Cochrane risk of bias tool (RoB 2). This tool includes the following domains: bias arising from the randomization process, bias due to deviations from intended interventions, bias due to missing outcome data, bias in measurement of the outcome, and bias in selection of the reported result. Each domain was rated as low risk, some concern, or high risk.

### Supplementary Text 3: Detailed information on data analysis and protocol amendment

***Data analysis***

Data were expressed as mean differences (MD) with 95% confidence intervals (CIs), calculated from changes from baseline values, for continuous outcomes. For adverse events, we conducted pairwise meta-analyses to calculate measures of relative (risk ratio) and absolute (risk difference) effects, together with their 95% CIs, using the number of events and non-events across study arms. Absolute risk difference was calculated from the overall risk ratio and baseline risk using the formula: absolute risk difference = baseline risk × (risk ratio − 1). We also interpreted the magnitude of the effect estimates on the basis of known or estimated MID thresholds for continuous outcomes **(Supplementary Table 3)**.

For continuous outcomes, we first conducted pairwise meta-analyses to calculate the difference in change from baseline values across study arms. List of rules for data estimation and conversion are provided in **Supplementary Table 4**. If the change from baseline values was not reported in the included trials, we calculated these values by using data from measures before and after the intervention. When these data were unavailable, endpoint data were used ^1^. If mean values and standard deviations (SDs) were not reported, we estimated them from available data ^2-5^. When it was not possible to estimate SDs, an imputed pooled SD from the other trials included in the meta-analysis was applied ^6^. For within-group differences, we calculated a correlation coefficient from studies that provided sufficient data. The correlation factors were calculated in the intervention group and the control group for each of the outcomes (correlation factors: body weight: intervention= 0.93, control= 0.91; waist circumference: intervention= 0.86, control= 0.88; body fat percentage: intervention= 0.90, control= 0.93; total cholesterol: intervention= 0.68, control= 0.68; LDL-C: intervention=0.62, control= 0.67; HDL-C: intervention= 0.75, control=0.77; triglycerides: intervention= 0.66, control= 0.74; SBP: intervention= 0.67, control= 0.63; DBP: intervention= 0.69, control= 0.70; FPG: intervention= 0.62, control= 0.70; HbA_1c_: intervention= 0.71, control= 0.79; fasting insulin: intervention= 0.60, control= 0.62; HOMA-IR: intervention= 0.74, control= 0.74; CRP: intervention= 0.64, control= 0.77; IL-6: intervention= 0.60, control= 0.50; TNF-α: intervention= 0.75, control= 0.82; and HRQoL: intervention= 0.50, control= 0.50). For crossover studies that reported their results as parallel group trials, a correlation of 0.5 was imputed in pairwise analysis to compute standard errors of differences ^7-9^. To mitigate the unit-of-analysis error from including trials with multiple intervention groups, we combined the intervention groups to create single pairwise comparisons ^10^. When two separate control phases were present within the same crossover study, both phases were averaged and compared with the whole grain intervention. Pooled MDs were calculated using random-effects DerSimonian and Laird ^11^ meta-analyses to incorporate within and between study components of variance. For outcomes with ≤5 studies, pooled estimates were generated using random-effects restricted maximum likelihood models (REMLs) with Knapp-Hartung correction ^12,13^. Heterogeneity between studies was evaluated with I^2^ and τ^2^ statistics. To assess the uncertainty around heterogeneity estimates, we calculated 95% CIs ^14^.

When at least 10 trials were available, a series of a priori specified subgroup analyses were conducted based on the risk of bias (low risk vs. high risk/some concerns; stronger effects in high-risk trials), health status (metabolically healthy vs. at risk; stronger effects in those who are at risk), and study duration (<8 weeks vs. ≥8 weeks; stronger effects at shorter durations). Additional post hoc subgroup analyses were performed by study design (parallel vs. crossover), whole-grain type (mixed whole grains, whole wheat, brown rice, barley, oat, rye, quinoa, buckwheat, amaranth, and corn), and presence of effect modifiers including calorie restriction (yes vs. no), exercise (yes vs. no), and behavioral support (yes vs. no). Exploratory analyses further examined the effects of longer-term intervention durations (<8, 8 to <12, 12 to <16, and ≥16 weeks), use of glucose lowering medications for measures of glycemic control, use of lipid lowering medications for measures of blood lipids, and use of blood pressure lowering medications for SBP and DBP, sex, baseline risk (low risk vs. high risk), comparator type (minimal intervention, low fiber/refined grains, and healthy diet), and use of run-in-period. For baseline risk, high baseline risk was defined as individuals with metabolic disease, medication users (e.g., glucose-, lipid-, and blood pressure-lowering medications), or those who are overweight/obese, while low baseline risk was defined as healthy individuals. For comparator type, the minimum intervention was defined as the usual or conventional diets, routine care, no intervention or placebo, while low-fiber/refined grains were defined as refined wheat, refined corn, white rice, and mixed refined grains. The Instrument to assess the Credibility of Effect Modification Analyses (ICEMAN) consists of eight criteria that were used to determine the credibility of the observed subgroup effects ^15^. **Supplementary** **Table 5** provides an overview of the domains covered by the ICEMAN tool and outlines the evaluation criteria for each domain. To assess the robustness of the findings, a series of sensitivity analyses were performed. First, the influence of individual studies was assessed by systematically removing each study from the meta-analysis and recalculating the pooled effect estimates. Second, to assess whether the method of outcome reporting influenced the results, we excluded all studies that provided only endpoint data (i.e., final follow‑up values without baseline values) and re‑analyzed the remaining studies with change‑from‑baseline data. This complements the first sensitivity analysis (leave‑one‑out sensitivity analysis) which examined the impact of any single study, regardless of data type. Third, correlation coefficients of 0.3, 0.6, and 0.9 were used to determine whether the overall results were robust to the use of different correlation coefficients ^5^. Fourth, the influence of background whole-grain intake was assessed by excluding studies that did not report this information. Fifth, we fitted quadratic and cubic (RCS) (with four knots) models. Due to the highly skewed distribution of the exposure variable and the large proportion of zero values, the default percentile-based knot selection (4th, 35th, 65th, 95th) resulted in non-unique knot locations and model convergence issues. Therefore, we specified knots manually at pre-defined, meaningful exposure levels to ensure appropriate model fitting and stability. Model fit was assessed by comparing Akaike information criteria (AIC) across competing models, with lower AIC values indicating better fit. Publication bias was investigated by inspection of contour-enhanced funnel plots ^16^ and formal testing using the Egger test ^17,18^.

We also used the method described by Crippa and Orsini ^19^ for linear and non-linear dose-response analyses. This method was applied using the dose of whole grain intake as dry weight (g/day), mean changes in the outcome measures and their standard errors, and the number of participants across trial arms. Because whole-grain foods vary widely in preparation and may be consumed either dry (e.g., breakfast cereals) or with considerable water content (e.g., cooked oatmeal or pasta). To address these inconsistencies and ensure comparability across trials, we harmonized all exposures to a consistent dry-weight metric ^20^.

Our approach was as follows: 1) many of the included studies used whole grains as the intervention, for which the dry weight was known, allowing for direct inclusion in the dose-response analysis; 2) for studies where the intervention consisted of whole-grain foods (e.g., bread, cereal, crackers), we included those studies only if they provided the dry weight of whole grains contained in the food product or reported sufficient data to compute the dry weight; 3) studies that reported only the total weight of the food product without specifying the dry weight of whole grain content were excluded from the dose-response analysis to avoid misclassification and ensure accuracy in the dose-response relationship; 4) for studies reporting the weight of cooked brown rice, we applied a conversion factor of 2.25 obtained from another included study^21^ to estimate the corresponding dry weight ^22,23^; 5) for studies that provided whole grains in prepared foods, we multiplied the amount of whole grain content by the reported serving size to estimate the weight of whole grains consumed ^24-31^; and 6) in one study, whole grains were given based on participant body weight ^32^. We estimated the total amount by multiplying the provided amount per kilogram by the mean body weight. By systematically harmonizing all exposure data to a consistent dry-weight metric, we minimized misclassification and improved comparability across trials, thereby enhancing the validity of our dose-response analysis.

Linear dose response analysis was calculated for an increase of 48 g/day, which approximates the intake level (3 servings/day) recommended by dietary guidelines ^33^. Non-linearity was modeled using one-stage restricted cubic splines with 3 knots (10%, 50%, and 90%). Analyses were conducted using STATA software, version 17. A two-tailed P-value of less than 0.05 was considered significant.

***Protocol amendment***

In our registered protocol in PROSPERO (CRD42024565726) ^34^, we planned to assess the dose-dependent effects of each one serving /day (30 g/day fresh weight) increase in whole grain consumption. However, considering that the majority of studies reported dry weight of whole grains, as recommended ^20^, we changed our increment unit and conducted our dose-response analyses accordingly. Moreover, due to the large variation in the amount of whole grain consumed, performing linear dose-response analysis per serving would have led to overly narrow CIs. Therefore, dose-response analysis was performed for a 48 g/day increment, equivalent to approximately 90 g fresh weight or 3 servings/day of whole grain. Regarding HRQoL, the protocol specified SMD as the summary statistic. However, since only one study was identified, we reported the results as MD. Originally, we also planned to include prediction intervals for the summary estimates; however, we decided against this because they were deemed less informative as they predict where a future study would lie relative to the summary estimate, but not where the summary estimate would lie with additional data. We also planned to evaluate the risk of bias using the original version of the Cochrane risk of bias tool. Because of the limitations of this tool, however, we decided to use the ROB-2.

### Supplementary Text 4: Instructions to rate the certainty of evidence using the GRADE approach

The certainty of the evidence was rated using the GRADE approach. Paired reviewers (SN and SK) independently performed GRADE assessments for each outcome. GRADE rates the certainty of evidence as high, moderate, low, or very low. Randomized controlled trials start as high certainty evidence that can then be downgraded based on pre-specified criteria. The criteria used to downgrade evidence include:

*Study limitations*-weight of studies showing risk of bias as assessed by the Cochrane Risk of Bias instrument ^35^. We rated down for risk of bias if most studies were at high risk of bias. For outcomes that included trials with both high and low risk of biases, we performed subgroup analyses based on study quality. When P for subgroup difference was not significant, all trials were included in the main analysis. When P for subgroup difference was significant, only the result in the subgroup of trials with a low risk of bias was reported.

*Inconsistency*-substantial between-study heterogeneity, I^2^ ≥ 50% and P_heterogeneity_< 0.10 in pairwise meta-analyses which remained unexplained in priori subgroup and sensitivity analyses ^36^.

*Indirectness-*presence of population, intervention, or comparator factors that limit the generalizability of the results ^37^.

*Imprecision*-the 95% CI for the mean difference is wide or the point estimate and its corresponding 95% CI do not surpass the minimal important difference (MID) ^38^. For imprecision, we did not rate down for imprecision where the point estimate and its 95% CI for the pairwise meta-analyses surpassed MID thresholds. Where the point estimates surpassed MID, we rated down if the 95% CI overlapped MID threshold. Please see **Supplementary Table 3** for MID thresholds. Point estimates larger than the MID thresholds were considered important, and those smaller than MID were considered clinically irrelevant.

*Publication bias*-compelling evidence of publication bias ^39^.

We also upgraded evidence as a result of the presence of a *dose response and large effect size.*

### Supplementary Text 5: Detailed information on the characteristics of studies included in the systematic review and meta-analysis

In total, 101 publications (87 studies) were included in the current systematic review and meta-analysis ^21-32,40-128^ (**Supplementary Figure 1**). Of these articles, several were published on the same populations ^25,26,40-54,56-59,91,95,117,122,124^, however, because they reported data on different types of cardiovascular risk factors, were not considered duplicates and were included in the analysis. The studies were published between 1986 and 2024. The number of participants in these studies ranged from 10 to 266, with an age range between 18 and 80 years. In total, 6529 participants were included in the 87 studies considered in this systematic review. Overall, 28 publications described studies that were conducted in Europe ^44-49,54,56-60,62,66,70,77,78,90,92,93,96,99,104,106,110,113,123,128^, 24 in the US ^27-31,50,53,64,71,72,76,82,86,89,97,98,100,102,103,107-109,119,125^, 44 in Asia ^21-24,32,40-43,51,52,55,61,63,65,67-69,73-75,79-81,83-85,88,91,94,95,101,105,112,114-118,120,122,124,126,127^, two in Australia ^87,111^, two in Canada ^25,26^, and one in Brazil ^121^. Duration of treatment ranged from two weeks to two years (median [interquartile range] = 8 weeks [6-12]). Two studies included only men ^79,94^ and seven included only women ^22,32,66,67,74,77,78^. Of the included trials, 15 (18 publications) implemented behavioral support in parallel with whole-grain intervention ^27,30,42,43,51,52,70,71,77,78,83,91,97,107,111,120,122,127^, 12 implemented calorie restriction ^27,30,70,71,74,78,80,102,103,108,116,127^, and three implemented physical activity programs ^30,71,108^. The clear majority of included trials had a parallel design whereas only 24 trials (27 publications) were of crossover design ^22,25,26,32,56-60,66,79,81,82,84,86,89,92-95,104-106,110,113,117,125^. Twenty-six publications (18 studies) ^22,28,29,44-54,56-59,70,75,77,78,81,84,96,108^ reported a run-in period, while the others either had no run-in period or did not report one. Thirty-six publications (33 studies) ^21,30,50-53,55,58,59,61,62,64,65,67,68,71,73,75-78,80,81,84,85,93,96,99-102,104,108,116,127,128^ reported that participants in their background diet had a low intake of whole grains, as assessed through food frequency questionnaires, food recalls/records, and serum levels of alkylresorcinols. In contrast, one study ^106^ reported a high intake of whole grains, while the remaining studies did not provide any information on this topic. Trials were conducted among adults with type 2 diabetes ^42,43,51,52,75,83,84,88,91,105,122^, prediabetes ^23,100,120^, metabolic syndrome ^21,44,45,48,49,54,63,71,80,94,114,115^, hypercholesterolemia ^24,28,29,31,40,41,46,47,56,57,68,69,82,92,95,98,101,107,109,111,113,117,124,125^, glucose intolerance or hypercholesterolemia ^110^, fatty liver ^65^, hypertension ^72,126^, H. pylori infection ^55^, and coeliac disease ^123^. Remaining trials were performed on healthy adults ^32,50,53,58,59,61,64,66,73,85,90,93,96,97,103,104,112,119^, those with overweight or obesity ^22,27,30,60,62,70,74,76-79,81,86,87,89,99,102,108,116,118,121,127,128^, and individuals with normoglycemia/hyperglycemia ^25,26^. Two other studies included women with premenstrual syndrome ^67^ and adults at high risk of metabolic syndrome ^106^. Three publications were rated to have a low risk of bias ^46,47,110^ and the remaining were rated as having some concerns or a high risk of bias (**Supplementary Table 8**)**.** Included studies assessed the effect of mixed whole grains ^25,26,44,45,48-50,53,54,58-60,62,64-67,71,76,77,82,86,89,93,102,106,108,126^, oat ^24,28-31,40,41,51,52,69,72,90,95,97,98,101,103,109-111,117-119,123,124^, black ^115^ or brown rice ^21-23,42,43,63,68,75,80,81,83-85,94,100,105,112,114,116^, whole wheat ^27,46,47,73,78,88,99,104,127^, rye ^55-57,70,113^, quinoa ^79,87,120,121^, barley ^32,61^, buckwheat ^91,122^, amaranth ^107^, and corn flour ^125^. In the study by Tighe et al.,^96^ the effects of mixed and whole wheat; in Suhr et al.^128^, rye and whole wheat; in Reiners et al.,^92^ barley and oat; and in Kim et al.,^74^ black and brown rice were investigated. The daily whole-grain dose ranged from 12 g/day ^61^ to 213 g/day ^102^ in the form of dry weight. The control arms consisted of a usual diet ^32,42,43,61,62,67,69,85,87,88,98,103,120,123^, mixed refined grains ^44-50,53,54,58-60,64-66,71,76-78,82,86,89,91,93,96,102,106,108,110,111,122,126,128^, refined rice ^21-23,40,41,63,68,74,75,80,81,83,84,94,95,100,105,112,114-117,124^, refined wheat ^24-26,55-57,70,73,79,83,92,99,101,104,113,127^, refined corn ^29,31,90,121,125^, low-fiber cereals ^30,72^, and a placebo ^109,118^. The remaining trials in the control arm consumed no oat products ^51,52,97,107,119^ or any additional fiber ^27^ throughout the study.

### Supplementary Text 6: Detailed findings on adverse events, HOMA-IR, CRP, TNF-α, and HRQoL with low or very low certainty evidence

**Supplementary Table 9** present the adverse events reported in the identified studies. Assessment of adverse events were reported in forty-two studies, of which twenty-six trials reported no adverse events and four reported no difference between groups in the incidence of adverse events over the study period. Twelve trials reported mild adverse events including bloating, abdominal pain, nausea, and increased stool frequency. Based on seven RCTs that reported sufficient information on adverse events, whole grain consumption was not clearly associated with mild to moderate adverse events (risk ratio: 1.53; 95% CI: 0.72 to 3.22; I^2^ = 0%; n=7 trials and 742 participants; low certainty evidence).

Whole grain consumption resulted in an important reduction in HOMA-IR (MD: -0.24; 95% CI: -0.39 to -0.09; I2 = 66.4%; n= 30 trials and 2486 participants; low certainty evidence). Combining data from studies that reported information on CRP yielded a clinically irrelevant reduction (MD: -0.38 mg/L; 95% CI: -0.55 to -0.21; I^2^ = 83.1%; n= 31 trials and 2509 participants; low certainty evidence). However, change in TNF-α levels was clinically irrelevant and the 95% CI overlapped with null effect (low certainty evidence). Based on information provided in one RCT, whole grain intake resulted in a clinically irrelevant reduction in HRQoL, but the 95% CI is wide and crossed the null effect (very low certainty evidence).

### Supplementary Text 7: Detailed findings of publication bias, sensitivity, subgroup, and dose‑response meta‑analyses

There was no evidence of small study bias such as publication bias with Egger’s test for most of the investigated outcomes, but for FPG, HbA_1c_, insulin, IL-6, and TNF-α Egger’s test indicated possible small study bias. However, Egger’s test attenuated to non-significance when we excluded outlying studies that reported on HbA_1c_ ^83^, insulin ^94^, IL-6 ^117^, and TNF-α ^99^ from the analyses. Moreover, for all outcomes, the contour-enhanced funnel plots suggested that evidence of asymmetry is not due to publication bias (**Supplementary Figures 21-37**).

Findings from the sensitivity analyses indicated that the findings of DBP, HbA_1c_, IL-6, and body fat percentage were not robust and were sensitive to the influence of single studies. However, for any of the other outcomes, the results were not substantially altered by the exclusion of any individual study, including the study ^82^ in which endpoint data was used for analysis. (**Supplementary Figures 38-54**). Sensitivity analyses using different correlation coefficients (0.3, 0.6, and 0.9), when calculating within-group differences and for crossover trials in pairwise analyses, had a minor impact on the results (**Supplementary Table 10**). The use of a correlation coefficient of 0.3 for IL-6 and HbA1c, and of 0.9 for DBP, yielded clinically irrelevant reductions with 95% CIs overlapping the null effect. However, for body fat percentage, a correlation coefficient of 0.9 yielded a clinically irrelevant reduction, with the 95% CI excluding the null effect. In the sensitivity analyses assessing the influence of background whole-grain intake, conducted by excluding studies with insufficient data, the results were less evident, with 95%CI crossing the null value for several outcomes including FPG, waist circumference, HOMA-IR, HbA_1c_, triglycerides, SBP, CRP, and IL-6 (**Supplementary Table 11**).

There were several differences across subgroups based on risk of bias (HOMA-IR), caloric restriction (triglycerides, body fat percentage, HOMA-IR, and IL-6), sex (HOMA-IR and CRP), health status (HDL-C), whole-grain type (blood lipids, HOMA-IR, CRP, and IL-6), use of run-in period (FPG, triglycerides, total cholesterol, and HDL-C), and sex (HOMA-IR and CRP) (**Supplementary Tables** **12-16**). There was only one group difference for risk of bias, where trials with a low risk of bias indicated stronger effects on HOMA-IR (MD: -0.60, 95%CI: -0.89 to -0.31; n=2) than trials with high risk of bias/some concerns (MD: -0.21, 95%CI: -0.36 to -0.05; n=28) (p group difference = 0.019, ICEMAN credibility = low; **Supplementary Table 13**). For caloric restriction, trials that implemented caloric restriction indicated greater effect on body fat percentage (MD: -0.68%, 95%CI: -1.08 to -0.27; n=4, p group difference=0.023; **Supplementary Table 12**), but in contrast, trials that did not implement caloric restriction indicated stronger effects on HOMA-IR (**Supplementary Table 13**), triglycerides (**Supplementary Table 14**), and IL-6 (**Supplementary Table 16**). Only a few trials implemented physical activity along with whole grains supplementation, precluding firm conclusions regarding the potential synergistic effects of physical activity. However, subgroup analyses by behavioral support did not indicate any meaningful impacts of implementing behavioral support along with whole grains supplementations. Subgroup analyses by sex indicated stronger effects on HOMA-IR (**Supplementary Table 13**) and CRP (**Supplementary Table 16)** in men, though based on very few studies, while the analyses of other outcomes did not indicate any difference across either sex.

The subgroup analyses by health status indicated similar effects in metabolically healthy vs at risk individuals, except for HDL-C, which indicated a decreasing effect in metabolically healthy individuals and a modest increase in HDL-C in at risk ones (p group difference= 0.017, **Supplementary Table 14**). This was also the case for baseline risk, where the effects were similar in individuals at low or high baseline risk, except for a stronger effect on waist circumference in individuals at high baseline risk (p group difference=0.037; **Supplementary Figure 12**). Of note, the effect of whole grains on FPG was stronger in individuals with type 2 diabetes (MD: -0.71 mmol/L, 95%CI: -1.30 to -0.12; n=7) than those without diabetes or unknown diabetes status (p group difference=0.03; **Supplementary Figure 13**). In addition, the subgroup analyses by medication use did not indicate any evidence of stronger effects on cardiometabolic outcomes in individuals who used respective medications (glucose-, lipid- or blood pressure-lowering medications).

There were some indications of a difference in effect by type of whole grains consumed, where rye trials indicated greater reductions in waist circumference (MD: -1.69 cm; 95% CI: -2.94 to -0.45; I^2^ = 0; n=2), brown rice trials indicated greater reductions in triglycerides (MD: -0.13 mmol/L; 95% CI: -0.23 to -0.02; I^2^ = 48.3%; n=16), total cholesterol (MD: -0.15 mmol/L; 95% CI: -0.28 to -0.02; I^2^ = 78.2%; n=16), and CRP (MD: -0.67 mg/L; 95% CI: -1.21 to -0.12; I^2^ = 83.1%; n=5), and oat trials indicated greater reductions in total cholesterol (MD: -0.30 mmol/L; 95% CI: -0.38 to -0.22; I^2^ = 60.5%; n=21) and LDL-C (MD: -0.20 mmol/L; 95% CI: -0.25 to -0.15; I^2^ = 0%; n=20). Interestingly, in the subgroup analysis by run-in period (yes vs no), trials that did not have a run-in period indicated stronger effects on FPG, triglycerides, total cholesterol, HDL-C, and probably SBP than trials that implemented a run-in period before intervention.

We assessed the credibility of the observed subgroup effects based on eight criteria introduced by the ICEMAN tool, when the p-value for subgroup differences was less than 0.10 (**Supplementary Table 17**). However, the credibility of subgroup differences was rated low.

***Dose-response meta-analyses***

For cardiometabolic outcomes, we fitted several dose‑response models. The restricted cubic spline model with 3 knots provided a better fit for all outcomes than the other models based on AIC. Visual inspection of the decorrelated residuals‑versus‑dose plot revealed no systematic patterns, further supporting the adequacy of the model. The results of Goodness-of-fit tests and measures for dose-response meta-analysis of whole grain consumption on cardiometabolic risk factors are provided in **Supplementary Tables 22-25** and **Supplementary Figures 72-101.**

### Supplementary Table 1: The terms used to search relevant publications on the effect of whole grains on the cardiometabolic risk factors

|  | Concept |  |  |
| --- | --- | --- | --- |
| PubMed | 1 | “whole grain”[All Fields] OR “wholegrain”[All Fields] OR “wholemeal”[All Fields] OR “whole meal”[All Fields] OR “wholewheat”[All Fields] OR “whole wheat”[All Fields] OR “wheat”[All Fields] OR “brown rice”[All Fields] OR “rice”[All Fields] OR “barley”[All Fields] OR “maize”[All Fields] OR “corn”[All Fields] OR “rye”[All Fields] OR “oat”[All Fields] OR “millet”[All Fields] OR “sorghum”[All Fields] OR “tef”[All Fields] OR “triticale”[All Fields] OR “canary seed”[All Fields] OR “job’s tears”[All Fields] OR “fonio”[All Fields] OR “wild rice”[All Fields] OR “amaranth”[All Fields] OR “buckwheat”[All Fields] OR “quinoa”[All Fields] OR “spelt”[All Fields] OR “emmer”[All Fields] OR “faro”[All Fields] OR “einkorn”[All Fields] OR “kamut”[All Fields] OR “durum”[All Fields] OR “bread”[All Fields] OR “cereals”[All Fields] OR “flour”[All Fields] OR whole grains [Mesh] |  |
|  | 2 | “trial”[All Fields] OR “randomized”[All Fields] OR “randomized”[All Fields] OR “placebo”[All Fields] OR “randomly”[All Fields] OR “crossover”[All Fields] OR “cross-over”[All Fields] OR “parallel”[All Fields] OR “clinical trials as topic”[Mesh] OR “randomized controlled trials as topic”[Mesh] OR “randomized controlled trial”[Publication Type] OR "controlled clinical trial"[Publication Type] |  |
|  | 3 | #1 AND #2 | 26684 |
| Scopus | 1 | TITLE-ABS-KEY("whole grain") OR TITLE-ABS-KEY("wholegrain") OR TITLE-ABS-KEY("wholemeal") OR TITLE-ABS-KEY("whole meal") OR TITLE-ABS-KEY("wholewheat") OR TITLE-ABS-KEY("whole wheat") OR TITLE-ABS-KEY("wheat") OR TITLE-ABS-KEY("brown rice") OR TITLE-ABS-KEY("rice") OR TITLE-ABS-KEY("barley") OR TITLE-ABS-KEY("maize") OR TITLE-ABS-KEY("corn") OR TITLE-ABS-KEY("rye") OR TITLE-ABS-KEY("oat") OR TITLE-ABS-KEY("millet") OR TITLE-ABS-KEY("sorghum") OR TITLE-ABS-KEY("tef") OR TITLE-ABS-KEY("triticale") OR TITLE-ABS-KEY("canary seed") OR TITLE-ABS-KEY("job's tears") OR TITLE-ABS-KEY("fonio") OR TITLE-ABS-KEY("wild rice") OR TITLE-ABS-KEY("amaranth") OR TITLE-ABS-KEY("buckwheat") OR TITLE-ABS-KEY("quinoa") OR TITLE-ABS-KEY("spelt") OR TITLE-ABS-KEY("emmer") OR TITLE-ABS-KEY("faro") OR TITLE-ABS-KEY("einkorn") OR TITLE-ABS-KEY("kamut") OR TITLE-ABS-KEY("durum") OR TITLE-ABS-KEY("bread") OR TITLE-ABS-KEY("cereals") OR TITLE-ABS-KEY("flour") |  |
|  | 2 | TITLE-ABS-KEY(trial) OR TITLE-ABS-KEY("randomized") OR TITLE-ABS-KEY("randomized") OR TITLE-ABS-KEY("placebo") OR TITLE-ABS-KEY("randomly") OR TITLE-ABS-KEY("crossover") OR TITLE-ABS-KEY("cross-over") OR TITLE-ABS-KEY("parallel") |  |
|  | 3 | ALL("human*") OR ALL("adult") OR ALL("male") OR ALL("female") OR ALL("man") OR TITLE-ABS-KEY("men") OR TITLE-ABS-KEY("woman") OR TITLE-ABS-KEY("women") |  |
|  | 4 | #1 AND #2 AND #3 | 37721 |
| Web of science | 1 | “whole grain” (Topic) or “wholegrain” (Topic) or “wholemeal” (Topic) or “whole meal” (Topic) or “wholewheat” (Topic) or “whole wheat” (Topic) or “wheat” (Topic) or “brown rice” (Topic) or “rice” (Topic) or “barley” (Topic) or “maize” (Topic) or “corn” (Topic) or “rye” (Topic) or “oat” (Topic) or “millet” (Topic) or “sorghum” (Topic) or “tef” (Topic) or “triticale” (Topic) or “canary seed” (Topic) or “job’s tears” (Topic) or “fonio” (Topic) or “wild rice” (Topic) or “amaranth” (Topic) or “buckwheat” (Topic) or “quinoa” (Topic) or “spelt” (Topic) or “emmer” (Topic) or “faro” (Topic) or “einkorn” (Topic) or “kamut” (Topic) or “durum” (Topic) or “bread” (Topic) or “cereals” (Topic) or “flour” (Topic) |  |
|  | 2 | “trial” (Topic) or “randomized” (Topic) or “randomized” (Topic) or “placebo” (Topic) or “randomly” (Topic) or “crossover” (Topic) or “cross-over” (Topic) or “parallel” (Topic) |  |
|  | 3 | “human*“ (All Fields) or “adult“ (All Fields) or “male“ (All Fields) or “female“ (All Fields) or “man“ (All Fields) or “men“ (All Fields) or “woman“ (All Fields) or “women“ (All Fields) |  |
|  | 4 | #1 AND #2 AND #3 | 14966 |
| CENTRAL | 1 | ("quinoa"):ti,ab,kw OR ("buckwheat"):ti,ab,kw OR ("amaranth"):ti,ab,kw OR ("millet"):ti,ab,kw OR ("whole grain"):ti,ab,kw OR ("maize"):ti,ab,kw  OR ("sorghum"):ti,ab,kw  OR ("rye"):ti,ab,kw  OR ("jobs tears"):ti,ab,kw OR ("canary seed"):ti,ab,kw OR ("einkorn"):ti,ab,kw  OR ("corn"):ti,ab,kw  OR ("triticale"):ti,ab,kw OR ("teff"):ti,ab,kw  OR ("fonio"):ti,ab,kw  OR ("spelt"):ti,ab,kw  OR ("emmer"):ti,ab,kw OR ("farro"):ti,ab,kw OR ("kamut"):ti,ab,kw |  |
|  | 2 | ("random"):ti,ab,kw OR ("randomized controlled trial"):ti,ab,kw OR ("clinical trial"):ti,ab,kw |  |
|  | 3 | #1 AND #2 | 3010 |
| Google scholar | 1 | “whole grain” and “randomized”  By searching the above combination in this engine, we screened the first 500 relevancy ranked papers to avoid missing any eligible studies. | 500 |
| Duplicate |  |  | 19207 |
| Total |  |  | 63674 |

A broad search strategy was intentionally employed—without restricting by outcomes, population, setting, or time frame—to maximize sensitivity and ensure comprehensive identification of all potentially eligible whole grain intervention trials.

### Supplementary Table 2: Conversion factor used to convert primary and secondary outcome units for data extraction

| 1 | Fasting plasma glucose was converted from mg/dl to mmol/l by dividing by 18 ^94^ |
| --- | --- |
| 2 | HDL-C, LDL-C, and total cholesterol were converted from mg/dl to mmol/l by multiplying by 0.02586 ^94^ |
| 3 | Triglycerides were converted from mg/dl to mmol/l by multiplying by 0.01129 ^94^ |
| 4 | HbA_1c_ was converted from mmol/mol to % using the following formula: NGSP (%) = (0.09148*IFCC(mmol/mol)) + 2.152 ^95^ |
| 5 | Insulin was converted from μIU/mL, mU/L, pg/ml, and ng/L to pmol/L by multiplying by 6.00, 6.00, 0.1721, and 0.1721, respectively ^94,129^ |

Abbreviations: LDL-C: low density lipoprotein cholesterol- HDL-C: high density lipoprotein cholesterol- HbA_1c_: glycated hemoglobin- NGSP: National Glycohemoglobin Standardization Program- IFCC: International Federation of Clinical Chemistry and Laboratory Medicine

Supplementary Table 3: Minimal clinically important differences (MID) for outcomes^1^

| Variable (ref) | MID (unit) |
| --- | --- |
| Body weight ^130^ | 2.5 (kg) |
| Waist circumference ^131^ | 2 (cm) |
| Body fat percentage ^131^ | 2 (%) |
| Systolic blood pressure ^132^ | 2 (mmHg) |
| Diastolic blood pressure ^132^ | 2 (mmHg) |
| Fasting insulin ^132^ | 5 (pmol/L) |
| Fasting plasma glucose ^132^ | 1.6 (mmol/L) |
| Hemoglobin A1c ^132^ | 0.5 (%) |
| Total cholesterol ^132^ | 0.26 (mmol/L) |
| Low density lipoprotein cholesterol ^132^ | 0.1 (mmol/L) |
| High density lipoprotein cholesterol ^132^ | 0.1 (mmol/L) |
| Triglycerides ^132^ | 0.09 (mmol/L) |
| C-reactive protein ^132^ | 0.5 (mg/L) |
| Homeostasis model assessment of insulin resistance ^132^ | 0.05 |
| Interleukin 6 ^133^ | 1 (pg/mL) |
| Tumor necrosis factor alpha ^133^ | 0.9 (pg/mL) |
| HRQoL ^133^ | 7 |

^1^ The MID for interleukin-6, tumor necrosis factor-alpha, and HRQoL was determined as half the standard deviation of the baseline values from the included trials.

### Supplementary Table 4: List of rules for data estimation and conversion

**Obtaining SD from SE ^2,5^**

$$SD=\sqrt{n}\times SE$$

**Obtaining SD from 95% confidence interval ^2^**

$$SD=\sqrt{n}\times\frac{(upper limit-lower limit)}{2\times t\_value}$$

**Obtaining SE from P-value and mean difference** **^4^**

$$SE=\frac{mean difference}{-0.862+sqrt(0.743-2.404\times\log\left( P \right))} (ignoring minus signs)$$

**Approximating mean from sample size, median and ranges ^2^**

$$Mean\approx\left( \frac{4}{4+n^{0.75}} \right)\frac{minimum value+maximum value}{2}+(\frac{n^{0.75}}{4+n^{0.75}})median$$

**Approximating SD from sample size and ranges ^2^**

$$SD\approx\frac{maximum value-minimum value}{2\emptyset^{-1}(\frac{n-0.375}{n+0.25})}$$

**Approximating mean from sample size, median and interquartile ranges ^2^**

$$Mean\approx\left( 0.7+\frac{0.39}{n} \right)\frac{first quartile+third quartile}{2}+\left( 0.3-\frac{0.39}{n} \right)median$$

**Approximating SD from sample size and interquartile ranges ^2^**

$$SD\approx\frac{third quartile-first quartile}{2\emptyset^{-1}(\frac{0.75n-0.125}{n+0.25})}$$

**Approximating SD from interquartile ranges ^5^**

$$SD\approx\frac{third quartile-first quartile}{1.35}$$

**Approximating raw mean from mean and SD** **on the log scale ^3^**

$${Mean}_{raw}\approx expotional({mean}_{log scale}+\frac{{SD}_{log scale}^{2}}{2})$$

**Approximating raw SD from mean and SD** **on the log scale ^3^**

$${SD}_{raw}\approx\sqrt{\left( expontial\left( {SD}_{log scale}^{2} \right)-1 \right)expontial(2\times{mean}_{log scale}+{SD}_{\log scale}^{2})}$$

**Calculating a correlation coefficient in the intervention group ^2,5^**

$${Correlation}_{intervention}=\frac{{SD}_{baseline}^{2}+{SD}_{final}^{2}-{SD}_{change}^{2}}{2\times{SD}_{baseline}\times{SD}_{final}}$$

**Calculating a correlation coefficient in the control group ^2,5^**

$${Correlation}_{control}=\frac{{SD}_{baseline}^{2}+{SD}_{final}^{2}-{SD}_{change}^{2}}{2\times{SD}_{baseline}\times{SD}_{final}}$$

**Imputing a change-from-baseline SD using a correlation coefficient ^2,5^**

$${SD}_{change}=\sqrt{{SD}_{baseline}^{2}+{SD}_{final}^{2}-2\times Correlation \times{SD}_{baseline}\times{SD}_{final}}$$

**Imputing SD from the other trials included in the meta-analysis ^6^**

$${SD}_{pooled}=\sqrt{\frac{\sum{(n}_{i}-1){SD}_{i}^{2}}{\sum(n_{i}-1)}}$$

**Combining the sample sizes of two subgroups into a single group ^5^***

$$n_{total}=n_{1}+n_{2}$$

**Combining the means of two subgroups into a single group ^5^***

$${Mean}_{pooled}=\frac{{n_{1}\times mean}_{1}+{n_{2}\times mean}_{2}}{n_{1}+n_{2}}$$

**Combining the SDs of two subgroups into a single group ^5^***

$${SD}_{pooled}=\sqrt{\frac{{(n}_{1}-1){SD}_{1}^{2}+{(n}_{2}-1){SD}_{2}^{2}+\frac{n_{1}\times n_{2}}{n_{1}+n_{2}}({mean}_{1}^{2}+{mean}_{2}^{2}-2\times{mean}_{1}\times{mean}_{2})}{n_{1}+n_{2}-1}}$$

Abbreviations: SD, standard deviation; SE, standard error.

∅^−1^=the percentile of the standard normal distribution.

*As mentioned in the Cochrane Handbook for Systematic Reviews of Interventions, the recommended method to overcome a unit-of-analysis error for a study that could contribute multiple, correlated comparisons is to combine all relevant experimental intervention groups of the study into a single group, and to combine all relevant comparator intervention groups into a single comparator group. Therefore, for the current study, means and standard deviations were combined using the formulas as described in the Cochrane Handbook for Systematic Reviews of Interventions.

### Supplementary Table 5: Description of the ICEMAN domains and how to judge each domain

| **1: Is the analysis of effect modification based on comparison within rather than between trials?** | | | |
| --- | --- | --- | --- |
| Completely between | Mostly between or unclear | Mostly within | Completely within |
| Subgroup analysis or meta-regression comparing overall effects of each individual trial. This is typical for aggregate data meta-analysis. | Subgroup analysis or meta-regression with most information coming from overall effects, but some trials providing within-trial subgroup information | Most trials providing within-trial subgroup information; or individual participant data analysis that combines within and between trial information | All trials providing within-trial subgroup information or individual participant data; and the analysis separates within from between trial information, e.g. meta-analysis of interactions |
| **2: For within-trial comparisons, is the effect modification similar from trial to trial?** Not applicable: no or one within-RCT comparison | | | |
| Definitely not similar | Probably not similar or unclear | Mostly similar | Definitely similar |
| Effect modification reported for two or more trials and clearly different directions | Effect modification not reported for individual trials or too imprecise to tell | Effect modification reported for two or more trials, mostly similar in direction, but considerable differences in magnitude | Effect modification reported for two or more trials, similar in direction, only some differences in magnitude |
| **3: For between-trial comparisons, is the number of trials large?** [ ] Not applicable: no between RCT comparison | | | |
| Very small | Rather small or unclear | Rather large | Large |
| 1 or 2 or in smallest subgroup; 5 or less in continuous meta-regression | 3-4 in smallest subgroup; 6-10 in continuous meta-regression | 5-9 in smallest subgroup; 11 to 15in continuous meta-regression | 10 or more in smallest subgroup; more than 15 in continuous meta-regression |
| **4: Was the direction of effect modification correctly hypothesized a priori?** | | | |
| Definitely no | Probably no or unclear | Probably yes | Definitely yes |
| Clearly post-hoc or results inconsistent with hypothesized direction or biologically very implausible | Vague hypothesis or hypothesized direction unclear | No prior protocol available but unequivocal statement of a priori hypothesis with correct direction of effect modification | Prior protocol available and includes correct specification of direction of effect modification, e.g. based on a biologic rationale |
| **5: Does a test for interaction suggest that chance is an unlikely explanation of the apparent effect modification?** (consider irrespective of number of effect modifiers) | | | |
| Chance a very likely explanation | Chance a likely explanation or unclear | Chance may not explain | Chance an unlikely explanation |
| Interaction or meta-regression p-value >0.05 | Interaction or meta-regression p-value ≤0.05 and >0.01, or no test of interaction reported and not computable | Interaction or meta-regression p-value ≤0.01 and >0.005 | Interaction or meta-regression p-value ≤0.005 |
| **6: Did the authors test only a small number of effect modifiers or consider the number in their statistical analysis?** | | | |
| Definitely no | Probably no or unclear | Probably yes | Definitely yes |
| Explicitly exploratory analysis or large number of effect modifiers tested (e.g. greater than 10) and multiplicity not considered in analysis | No mention of number or 4-10 effect modifiers tested and number not considered in analysis | No protocol available but unequivocal statement of 3 or fewer effect modifiers tested | Protocol available and 3 or fewer effect modifiers tested or number considered in analysis |
| **7: Did the authors use a random effects model?** | | | |
| Definitely no | Probably no or unclear | Probably yes | Definitely yes |
| Fixed (or common) effect or fixed effects model explicitly stated | Probably fixed effect(s) model | Probably random (or mixed) effects | Random (or mixed) effects explicitly stated |
| **8: If the effect modifier is a continuous variable, were arbitrary cut points avoided?** [ ] not applicable: not continuous | | | |
| Definitely no | Probably no or unclear | Probably yes | Definitely yes |
| Analysis based on exploratory cut point(s), e.g. picking cut point associated with highest interaction p-value | Analysis based on cut point(s) of unclear origin | Analysis based on pre-specified cut point(s), e.g. suggested by prior RCT | Analysis based on the full continuum, e.g. assuming a linear or logarithmic relationship |
| **9 Optional: Are there any additional considerations that may increase or decrease credibility?** (manual section 3.9) [ ] not applicable | | | |
| yes, probably decrease | | yes, probably increase | |
| **10. How would you rate the overall credibility of the proposed effect modification?**  Overall rating: The overall rating should be derived by the items that decrease credibility:  **Very low**: All responses definitely or probably decrease credibility or unclear  **Maximum usually low**: Two or more responses definitely decrease credibility even if all other responses satisfy credibility criteria  **Maximum usually moderate**: One response definitely decreases credibility even if all other responses satisfy credibility criteria  **Maximum usually moderate**: Two responses probably decrease credibility even if all other responses satisfy credibility criteria  **High very likely**: No response options definitely or probably decrease credibility | | | |
| **11. How would you interpreted the overall credibility of the proposed effect modification?**  **Very low**: Very likely no effect modification. Use overall effect for each subgroup.  **Maximum usually low**: Likely no effect modification. Use overall effect for each subgroup but note remaining uncertainty.  **Maximum usually moderate**: Likely effect modification. Use separate effect for each subgroup but note remaining uncertainty.  **High very likely**: Very likely effect modification. Use separate effect for each subgroup. | | | |

Abbreviation: ICEMAN: Instrument to assess the Credibility of Effect Modification Analyses

### Supplementary Table 6: List of publications excluded by full text assessment

**Duplicate publications**

1. Malin SK, Kullman EL, Scelsi AR, et al. A whole-grain diet reduces peripheral insulin resistance and improves glucose kinetics in obese adults: A randomized-controlled trial. *Metabolism: clinical and experimental* 2018;82:111-17. doi: 10.1016/j.metabol.2017.12.011 [published Online First: 2018/01/07]

2. Vuholm S, Nielsen DS, Iversen KN, et al. Whole-Grain Rye and Wheat Affect Some Markers of Gut Health without Altering the Fecal Microbiota in Healthy Overweight Adults: A 6-Week Randomized Trial. *The Journal of nutrition* 2017;147(11):2067-75. doi: 10.3945/jn.117.250647 [published Online First: 2017/09/29]

3. Turpeinen AM, Juntunen K, Mutanen M, et al. Similar responses in hemostatic factors after consumption of wholemeal rye bread and low-fiber wheat bread. European journal of clinical nutrition 2000;54(5):418-23. doi: 10.1038/sj.ejcn.1600975 [published Online First: 2000/05/24]

4. Christensen L, Vuholm S, Roager HM, et al. Prevotella Abundance Predicts Weight Loss Success in Healthy, Overweight Adults Consuming a Whole-Grain Diet Ad Libitum: A Post Hoc Analysis of a 6-Wk Randomized Controlled Trial. *The Journal of nutrition* 2019;149(12):2174-81. doi: 10.1093/jn/nxz198 [published Online First: 2019/09/11]

5. Mey JT, Godin JP, Scelsi AR, et al. A Whole-Grain Diet Increases Whole-Body Protein Balance Compared with a Macronutrient-Matched Refined-Grain Diet. *Current developments in nutrition* 2021;5(11):nzab121. doi: 10.1093/cdn/nzab121 [published Online First: 2021/11/23]

6. Tighe P, Duthie G, Brittenden J, et al. Effects of wheat and oat-based whole grain foods on serum lipoprotein size and distribution in overweight middle aged people: a randomised controlled trial. *PloS one* 2013;8(8):e70436. doi: 10.1371/journal.pone.0070436 [published Online First: 2013/08/14]

7. Polito R, Costabile G, Nigro E, et al. Nutritional factors influencing plasma adiponectin levels: results from a randomised controlled study with whole-grain cereals. *International journal of food sciences and nutrition* 2020;71(4):509-15. doi: 10.1080/09637486.2019.1680959 [published Online First: 2019/10/23]

8. Malin SK, Kullman EL, Scelsi AR, et al. A Whole-Grain Diet Increases Glucose-Stimulated Insulin Secretion Independent of Gut Hormones in Adults at Risk for Type 2 Diabetes. *Molecular nutrition & food research* 2019;63(7):e1800967. doi: 10.1002/mnfr.201800967 [published Online First: 2019/02/15]

9. Gu J, Jing L, Ma X, et al. Naked oat combined with a structured dietary intervention affects oxidative stress but not inflammation in diabetic dyslipidemia. *Nutrition, metabolism, and cardiovascular diseases : NMCD* 2014;24(12):e35-7. doi: 10.1016/j.numecd.2014.08.009 [published Online First: 2014/10/16]

10. Faits T, Walker ME, Rodriguez-Morato J, et al. Exploring changes in the human gut microbiota and microbial-derived metabolites in response to diets enriched in simple, refined, or unrefined carbohydrate-containing foods: a post hoc analysis of a randomized clinical trial. *The American journal of clinical nutrition* 2020;112(6):1631-41. doi: 10.1093/ajcn/nqaa254 [published Online First: 2020/09/17]

11. Iversen KN, Dicksved J, Zoki C, et al. The Effects of High Fiber Rye, Compared to Refined Wheat, on Gut Microbiota Composition, Plasma Short Chain Fatty Acids, and Implications for Weight Loss and Metabolic Risk Factors (the RyeWeight Study). *Nutrients* 2022;14(8) doi: 10.3390/nu14081669 [published Online First: 2022/04/24]

12. Vetrani C, Costabile G, Luongo D, et al. Effects of whole-grain cereal foods on plasma short chain fatty acid concentrations in individuals with the metabolic syndrome. *Nutrition* 2016;32(2):217-21. doi: 10.1016/j.nut.2015.08.006

13. Lappi J, Salojärvi J, Kolehmainen M, et al. Intake of whole-grain and fiber-rich rye bread versus refined wheat bread does not differentiate intestinal microbiota composition in Finnish adults with metabolic syndrome. *The Journal of nutrition* 2013;143(5):648-55. doi: 10.3945/jn.112.172668 [published Online First: 2013/03/22]

14. Ross AB, Bourgeois A, Macharia HN, et al. Plasma alkylresorcinols as a biomarker of whole-grain food consumption in a large population: results from the WHOLEheart Intervention Study. *The American journal of clinical nutrition* 2012;95(1):204-11. doi: 10.3945/ajcn.110.008508 [published Online First: 2011/12/16]

15. Linko AM, Juntunen KS, Mykkänen HM, et al. Whole-grain rye bread consumption by women correlates with plasma alkylresorcinols and increases their concentration compared with low-fiber wheat bread. *The Journal of nutrition* 2005;135(3):580-3. doi: 10.1093/jn/135.3.580 [published Online First: 2005/03/01]

16. Gijbels A, Schutte S, Esser D, et al. Effects of a 12-week whole-grain or refined wheat intervention on plasma acylcarnitines, bile acids and signaling lipids, and association with liver fat: A post-hoc metabolomics study of a randomized controlled trial. *Frontiers in nutrition* 2022;9:1026213. doi: 10.3389/fnut.2022.1026213 [published Online First: 2022/11/05]

17. Hanhineva K, Lankinen MA, Pedret A, et al. Nontargeted metabolite profiling discriminates diet-specific biomarkers for consumption of whole grains, fatty fish, and bilberries in a randomized controlled trial. *The Journal of nutrition* 2015;145(1):7-17. doi: 10.3945/jn.114.196840 [published Online First: 2014/12/21]

**In patients with cancer**

18. Kapoor N, Naufahu J, Tewfik S, et al. A Prospective Randomized Controlled Trial to Study the Impact of a Nutrition-Sensitive Intervention on Adult Women With Cancer Cachexia Undergoing Palliative Care in India. *Integrative cancer therapies* 2017;16(1):74-84. doi: 10.1177/1534735416651968 [published Online First: 2016/06/03]

19. Landberg R, Andersson SO, Zhang JX, et al. Rye whole grain and bran intake compared with refined wheat decreases urinary C-peptide, plasma insulin, and prostate specific antigen in men with prostate cancer. *The Journal of nutrition* 2010;140(12):2180-6. doi: 10.3945/jn.110.127688 [published Online First: 2010/10/29]

20. Zamaratskaia G, Mhd Omar NA, Brunius C, et al. Consumption of whole grain/bran rye instead of refined wheat decrease concentrations of TNF-R2, e-selectin, and endostatin in an exploratory study in men with prostate cancer. *Clinical nutrition (Edinburgh, Scotland)* 2020;39(1):159-65. doi: 10.1016/j.clnu.2019.01.007 [published Online First: 2019/01/28]

**Comparison of two types of whole grains**

21. Saglam D, Saka M, Sayaca N. The effect of consumption of low glycemic index, high fat content bread on anthropometric measurement and cardiometabolic risk factors in women with type 2 diabetes mellitus. *International Journal of Diabetes in Developing Countries* 2019;39(1):166-72. doi: 10.1007/s13410-018-0624-3

22. Stefoska-Needham A, Beck EJ, Johnson SK, et al. A Diet Enriched with Red Sorghum Flaked Biscuits, Compared to a Diet Containing White Wheat Flaked Biscuits, Does Not Enhance the Effectiveness of an Energy-Restricted Meal Plan in Overweight and Mildly Obese Adults. *Journal of the American College of Nutrition* 2017;36(3):184-92. doi: 10.1080/07315724.2016.1237314 [published Online First: 2017/02/01]

23. Anunciação PC, Cardoso LM, Alfenas RCG, et al. Extruded sorghum consumption associated with a caloric restricted diet reduces body fat in overweight men: A randomized controlled trial. *Food research international (Ottawa, Ont)* 2019;119:693-700. doi: 10.1016/j.foodres.2018.10.048 [published Online First: 2019/03/20]

24. Kemppainen T, Heikkinen M, Ristikankare M, et al. Effect of unkilned and large amounts of oats on nutritional state of celiac patients in remission. *e-SPEN, the European e-Journal of Clinical Nutrition and Metabolism* 2009;4(1):e30-e34. doi: <https://doi.org/10.1016/j.eclnm.2008.10.001>

25. Gamel TH, Abdel-Aal EM, Tucker AJ, et al. Consumption of whole purple and regular wheat modestly improves metabolic markers in adults with elevated high-sensitivity C-reactive protein: a randomised, single-blind parallel-arm study. *The British journal of nutrition* 2020;124(11):1179-89. doi: 10.1017/s0007114520002275 [published Online First: 2020/07/01]

26. Eriksen AK, Brunius C, Mazidi M, et al. Effects of whole-grain wheat, rye, and lignan supplementation on cardiometabolic risk factors in men with metabolic syndrome: a randomized crossover trial. *The American journal of clinical nutrition* 2020;111(4):864-76. doi: 10.1093/ajcn/nqaa026 [published Online First: 2020/02/26]

27. Åberg S, Mann J, Neumann S, et al. Whole-Grain Processing and Glycemic Control in Type 2 Diabetes: A Randomized Crossover Trial. *Diabetes care* 2020;43(8):1717-23. doi: 10.2337/dc20-0263 [published Online First: 2020/05/20]

28. Martínez I, Lattimer JM, Hubach KL, et al. Gut microbiome composition is linked to whole grain-induced immunological improvements. *The ISME journal* 2013;7(2):269-80. doi: 10.1038/ismej.2012.104 [published Online First: 2012/10/06]

29. Kallio P, Kolehmainen M, Laaksonen DE, et al. Inflammation markers are modulated by responses to diets differing in postprandial insulin responses in individuals with the metabolic syndrome. *The American journal of clinical nutrition* 2008;87(5):1497-503. doi: 10.1093/ajcn/87.5.1497 [published Online First: 2008/05/13]

30. Davy BM, Melby CL, Beske SD, et al. Oat consumption does not affect resting casual and ambulatory 24-h arterial blood pressure in men with high-normal blood pressure to stage I hypertension. *The Journal of nutrition* 2002;132(3):394-8. doi: 10.1093/jn/132.3.394 [published Online First: 2002/03/07]

31. Lúcio H, Anunciação P, da Silva B, et al. Consumption of Extruded Sorghum SC319 Improved Gut Microbiota at Genus Level and Reduced Anthropometric Markers in Men with Overweight: A Randomized Controlled Clinical Trial. *Nutrients* 2023;15(17) doi: 10.3390/nu15173786 [published Online First: 2023/09/09]

32. McIntosh GH, Whyte J, McArthur R, et al. Barley and wheat foods: influence on plasma cholesterol concentrations in hypercholesterolemic men. *The American journal of clinical nutrition* 1991;53(5):1205-9. doi: 10.1093/ajcn/53.5.1205 [published Online First: 1991/05/01]

33. Behall KM, Scholfield DJ, Hallfrisch J. Whole-grain diets reduce blood pressure in mildly hypercholesterolemic men and women. *Journal of the American Dietetic Association* 2006;106(9):1445-9. doi: 10.1016/j.jada.2006.06.010 [published Online First: 2006/09/12]

34. Behall KM, Scholfield DJ, Hallfrisch J. Diets containing barley significantly reduce lipids in mildly hypercholesterolemic men and women. *The American journal of clinical nutrition* 2004;80(5):1185-93. doi: 10.1093/ajcn/80.5.1185 [published Online First: 2004/11/09]

35. Behall KM, Scholfield DJ, Hallfrisch J. Lipids significantly reduced by diets containing barley in moderately hypercholesterolemic men. *Journal of the American College of Nutrition* 2004;23(1):55-62. doi: 10.1080/07315724.2004.10719343 [published Online First: 2004/02/14]

**Consumption with semi-whole grain in the control group**

36. Spisni E, Valerii MC, De Fazio L, et al. A Khorasan wheat-based diet improves systemic inflammatory profile in semi-professional basketball players: a randomized crossover pilot study. *Journal of the science of food and agriculture* 2020;100(11):4101-07. doi: 10.1002/jsfa.9947 [published Online First: 2019/07/28]

37. Sofi F, Whittaker A, Cesari F, et al. Characterization of Khorasan wheat (Kamut) and impact of a replacement diet on cardiovascular risk factors: cross-over dietary intervention study. *European journal of clinical nutrition* 2013;67(2):190-5. doi: 10.1038/ejcn.2012.206 [published Online First: 2013/01/10]

**Multicomponent interventions**

38. Ginos BNR, Navarro SL, Schwarz Y, et al. Circulating bile acids in healthy adults respond differently to a dietary pattern characterized by whole grains, legumes and fruits and vegetables compared to a diet high in refined grains and added sugars: A randomized, controlled, crossover feeding study. *Metabolism: clinical and experimental* 2018;83:197-204. doi: 10.1016/j.metabol.2018.02.006 [published Online First: 2018/02/20]

39. Kris-Etherton PM, Taylor DS, Smiciklas-Wright H, et al. High-soluble-fiber foods in conjunction with a telephone-based, personalized behavior change support service result in favorable changes in lipids and lifestyles after 7 weeks. *Journal of the American Dietetic Association* 2002;102(4):503-10. doi: 10.1016/s0002-8223(02)90116-1 [published Online First: 2002/05/03]

40. Angelino D, Martina A, Rosi A, et al. Glucose- and Lipid-Related Biomarkers Are Affected in Healthy Obese or Hyperglycemic Adults Consuming a Whole-Grain Pasta Enriched in Prebiotics and Probiotics: A 12-Week Randomized Controlled Trial. *The Journal of nutrition* 2019;149(10):1714-23. doi: 10.1093/jn/nxz071 [published Online First: 2019/06/05]

41. Lee YM, Kim SA, Lee IK, et al. Effect of a Brown Rice Based Vegan Diet and Conventional Diabetic Diet on Glycemic Control of Patients with Type 2 Diabetes: A 12-Week Randomized Clinical Trial. *PloS one* 2016;11(6):e0155918. doi: 10.1371/journal.pone.0155918 [published Online First: 2016/06/03]

42. Xiao S, Fei N, Pang X, et al. A gut microbiota-targeted dietary intervention for amelioration of chronic inflammation underlying metabolic syndrome. *FEMS microbiology ecology* 2014;87(2):357-67. doi: 10.1111/1574-6941.12228 [published Online First: 2013/10/15]

43. Foerster J, Maskarinec G, Reichardt N, et al. The influence of whole grain products and red meat on intestinal microbiota composition in normal weight adults: a randomized crossover intervention trial. *PloS one* 2014;9(10):e109606. doi: 10.1371/journal.pone.0109606 [published Online First: 2014/10/10]

44. Venn BJ, Perry T, Green TJ, et al. The effect of increasing consumption of pulses and wholegrains in obese people: a randomized controlled trial. *Journal of the American College of Nutrition* 2010;29(4):365-72. doi: 10.1080/07315724.2010.10719853 [published Online First: 2010/11/03]

45. Hong SH, Kim M, Woo M, et al. The amelioration of plasma lipids by Korean traditional confectionery in middle-aged women: A cross-over study with western cookie. *Nutrition research and practice* 2016;10(6):590-96. doi: 10.4162/nrp.2016.10.6.590 [published Online First: 2016/12/03]

46. Laaksonen DE, Toppinen LK, Juntunen KS, et al. Dietary carbohydrate modification enhances insulin secretion in persons with the metabolic syndrome. *The American journal of clinical nutrition* 2005;82(6):1218-27. doi: 10.1093/ajcn/82.6.1218 [published Online First: 2005/12/08]

47. Sobhana PP, Kandlakunta B, Nagaraju R, et al. Human clinical trial to assess the effect of consumption of multigrain Indian bread on glycemic regulation in type 2 diabetic participants. *Journal of food biochemistry* 2020;44(11):e13465. doi: 10.1111/jfbc.13465 [published Online First: 2020/10/03]

**Investigating the effect of pearled barley**

48. Goto Y, Nishimoto Y, Murakami S, et al. Metabologenomic Approach Reveals Intestinal Environmental Features Associated with Barley-Induced Glucose Tolerance Improvements in Japanese: A Randomized Controlled Trial. *Nutrients* 2022;14(17) doi: 10.3390/nu14173468 [published Online First: 2022/09/10]

**Cross-over trials without washout period and lack of information of the first phase**

49. Giacco R, Clemente G, Cipriano D, et al. Effects of the regular consumption of wholemeal wheat foods on cardiovascular risk factors in healthy people. *Nutrition, metabolism, and cardiovascular diseases : NMCD* 2010;20(3):186-94. doi: 10.1016/j.numecd.2009.03.025 [published Online First: 2009/06/09]

50. Bird AR, Vuaran MS, King RA, et al. Wholegrain foods made from a novel high-amylose barley variety (Himalaya 292) improve indices of bowel health in human subjects. *The British journal of nutrition* 2008;99(5):1032-40. doi: 10.1017/s000711450783902x [published Online First: 2007/10/09]

51. McIntosh GH, Noakes M, Royle PJ, et al. Whole-grain rye and wheat foods and markers of bowel health in overweight middle-aged men. *The American journal of clinical nutrition* 2003;77(4):967-74. doi: 10.1093/ajcn/77.4.967 [published Online First: 2003/03/29]

52. Poulter N, Chang CL, Cuff A, et al. Lipid profiles after the daily consumption of an oat-based cereal: a controlled crossover trial. *The American journal of clinical nutrition* 1994;59(1):66-9. doi: 10.1093/ajcn/59.1.66 [published Online First: 1994/01/01]

53. McGeoch SC, Johnstone AM, Lobley GE, et al. A randomized crossover study to assess the effect of an oat-rich diet on glycaemic control, plasma lipids and postprandial glycaemia, inflammation and oxidative stress in Type 2 diabetes. *Diabetic medicine : a journal of the British Diabetic Association* 2013;30(11):1314-23. doi: 10.1111/dme.12228 [published Online First: 2013/05/15]

54. Keski-Rahkonen P, Kolehmainen M, Lappi J, et al. Decreased plasma serotonin and other metabolite changes in healthy adults after consumption of wholegrain rye: an untargeted metabolomics study. *The American journal of clinical nutrition* 2019;109(6):1630-39. doi: 10.1093/ajcn/nqy394 [published Online First: 2019/05/29]

55. Pick ME, Hawrysh ZJ, Gee MI, et al. Barley Bread products improve glycemic control of Type 2 subjects. *International journal of food sciences and nutrition* 1998;49(1):71-78. doi: 10.3109/09637489809086406

**Active control group**

56. Fatahi S, Daneshzad E, Kord-Varkaneh H, et al. Impact of Diets Rich in Whole Grains and Fruits and Vegetables on Cardiovascular Risk Factors in Overweight and Obese Women: A Randomized Clinical Feeding Trial. *Journal of the American College of Nutrition* 2018;37(7):568-77. doi: 10.1080/07315724.2018.1444520 [published Online First: 2018/04/28]

57. Arabzadegan N, Daneshzad E, Fatahi S, et al. Effects of dietary whole grain, fruit, and vegetables on weight and inflammatory biomarkers in overweight and obese women. *Eating and weight disorders : EWD* 2020;25(5):1243-51. doi: 10.1007/s40519-019-00757-x [published Online First: 2019/08/11]

58. De Oliveira M, Sichieri R. Meal frequency and serum cholesterol of women in a fruit-or-fiber supplemented diet. *Revista de Nutrição* 2004;17:449-59.

59. Rodríguez-Rodríguez E, Aparicio A, Bermejo LM, et al. Changes in the sensation of hunger and well-being before and after meals in overweight/obese women following two types of hypoenergetic diet. *Public health nutrition* 2009;12(1):44-50. doi: 10.1017/s1368980008001912 [published Online First: 2008/03/08]

60. Jenkins DJA, Chiavaroli L, Mirrahimi A, et al. Glycemic Index Versus Wheat Fiber on Arterial Wall Damage in Diabetes: A Randomized Controlled Trial. *Diabetes care* 2022;45(12):2862-70. doi: 10.2337/dc22-1028 [published Online First: 2022/11/04]

61. Rave K, Roggen K, Dellweg S, et al. Improvement of insulin resistance after diet with a whole-grain based dietary product: results of a randomized, controlled cross-over study in obese subjects with elevated fasting blood glucose. *The British journal of nutrition* 2007;98(5):929-36. doi: 10.1017/s0007114507749267 [published Online First: 2007/06/15]

**Non-randomized studies**

62. Bui TN, Le TH, Nguyen do H, et al. Pre-germinated brown rice reduced both blood glucose concentration and body weight in Vietnamese women with impaired glucose tolerance. *Journal of nutritional science and vitaminology* 2014;60(3):183-7. doi: 10.3177/jnsv.60.183 [published Online First: 2014/08/01]

63. Størsrud S, Hulthén LR, Lenner RA. Beneficial effects of oats in the gluten-free diet of adults with special reference to nutrient status, symptoms and subjective experiences. *The British journal of nutrition* 2003;90(1):101-7. doi: 10.1079/bjn2003872 [published Online First: 2003/07/08]

**Examining the acute (postprandial) effects of whole grains**

64. Wolever TMS, Jones PJH, Jenkins AL, et al. Glycaemic and insulinaemic impact of oats soaked overnight in milk vs. cream of rice with and without sugar, nuts, and seeds: a randomized, controlled trial. *European journal of clinical nutrition* 2019;73(1):86-93. doi: 10.1038/s41430-018-0329-1 [published Online First: 2018/10/10]

65. Callcott ET, Blanchard CL, Snell P, et al. The anti-inflammatory and antioxidant effects of pigmented rice consumption in an obese cohort. *Food & function* 2019;10(12):8016-25. doi: 10.1039/c9fo02261a [published Online First: 2019/11/22]

66. Hajira B, Khan I. Effect of sorghum and barley-containing bread on plasma total polyphenols, antioxidant status and inflammation in healthy subjects. *Journal of food science and technology* 2022;59(12):4935-44. doi: 10.1007/s13197-022-05582-2 [published Online First: 2022/10/25]

67. Belobrajdic DP, Regina A, Klingner B, et al. High-Amylose Wheat Lowers the Postprandial Glycemic Response to Bread in Healthy Adults: A Randomized Controlled Crossover Trial. *The Journal of nutrition* 2019;149(8):1335-45. doi: 10.1093/jn/nxz067 [published Online First: 2019/06/05]

68. Hamad S, Zafar TA, Sidhu J. Parboiled rice metabolism differs in healthy and diabetic individuals with similar improvement in glycemic response. *Nutrition (Burbank, Los Angeles County, Calif)* 2018;47:43-49. doi: 10.1016/j.nut.2017.09.010 [published Online First: 2018/02/13]

69. Costabile G, Griffo E, Cipriano P, et al. Subjective satiety and plasma PYY concentration after wholemeal pasta. *Appetite* 2018;125:172-81. doi: 10.1016/j.appet.2018.02.004 [published Online First: 2018/02/11]

70. Sandberg JC, Björck IME, Nilsson AC. Effects of whole grain rye, with and without resistant starch type 2 supplementation, on glucose tolerance, gut hormones, inflammation and appetite regulation in an 11-14.5 hour perspective; a randomized controlled study in healthy subjects. *Nutrition journal* 2017;16(1):25. doi: 10.1186/s12937-017-0246-5 [published Online First: 2017/04/23]

71. Santaliestra-Pasías AM, Garcia-Lacarte M, Rico MC, et al. Effect of two bakery products on short-term food intake and gut-hormones in young adults: a pilot study. *International journal of food sciences and nutrition* 2015;67(5):562-70. doi: 10.1080/09637486.2016.1180348 [published Online First: 2016/05/21]

72. Sandberg JC, Björck IM, Nilsson AC. Rye-Based Evening Meals Favorably Affected Glucose Regulation and Appetite Variables at the Following Breakfast; A Randomized Controlled Study in Healthy Subjects. *PloS one* 2016;11(3):e0151985. doi: 10.1371/journal.pone.0151985 [published Online First: 2016/03/19]

73. Johansson DP, Lee I, Risérus U, et al. Effects of unfermented and fermented whole grain rye crisp breads served as part of a standardized breakfast, on appetite and postprandial glucose and insulin responses: a randomized cross-over trial. *PloS one* 2015;10(3):e0122241. doi: 10.1371/journal.pone.0122241 [published Online First: 2015/04/01]

74. Forsberg T, Åman P, Landberg R. Effects of whole grain rye crisp bread for breakfast on appetite and energy intake in a subsequent meal: two randomised controlled trails with different amounts of test foods and breakfast energy content. *Nutrition journal* 2014;13:26. doi: 10.1186/1475-2891-13-26 [published Online First: 2014/03/26]

75. Kristensen M, Jensen MG, Riboldi G, et al. Wholegrain vs. refined wheat bread and pasta. Effect on postprandial glycemia, appetite, and subsequent ad libitum energy intake in young healthy adults. *Appetite* 2010;54(1):163-9. doi: 10.1016/j.appet.2009.10.003 [published Online First: 2009/10/20]

76. Lioger D, Fardet A, Foassert P, et al. Influence of sourdough prefermentation, of steam cooking suppression and of decreased sucrose content during wheat flakes processing on the plasma glucose and insulin responses and satiety of healthy subjects. *Journal of the American College of Nutrition* 2009;28(1):30-6. doi: 10.1080/07315724.2009.10719758 [published Online First: 2009/07/03]

77. Sandberg JC, Björck IME, Nilsson AC. Impact of rye-based evening meals on cognitive functions, mood and cardiometabolic risk factors: a randomized controlled study in healthy middle-aged subjects. *Nutrition journal* 2018;17(1):102. doi: 10.1186/s12937-018-0412-4 [published Online First: 2018/11/08]

78. Ulmius M, Johansson A, Onning G. The influence of dietary fibre source and gender on the postprandial glucose and lipid response in healthy subjects. *European journal of nutrition* 2009;48(7):395-402. doi: 10.1007/s00394-009-0026-x [published Online First: 2009/05/06]

79. Priebe MG, Wang H, Weening D, et al. Factors related to colonic fermentation of nondigestible carbohydrates of a previous evening meal increase tissue glucose uptake and moderate glucose-associated inflammation. *The American journal of clinical nutrition* 2010;91(1):90-7. doi: 10.3945/ajcn.2009.28521 [published Online First: 2009/11/06]

80. Johansson EV, Nilsson AC, Östman EM, et al. Effects of indigestible carbohydrates in barley on glucose metabolism, appetite and voluntary food intake over 16 h in healthy adults. *Nutrition journal* 2013;12:46. doi: 10.1186/1475-2891-12-46 [published Online First: 2013/04/13]

81. Prykhodko O, Sandberg J, Burleigh S, et al. Impact of Rye Kernel-Based Evening Meal on Microbiota Composition of Young Healthy Lean Volunteers With an Emphasis on Their Hormonal and Appetite Regulations, and Blood Levels of Brain-Derived Neurotrophic Factor. *Frontiers in nutrition* 2018;5:45. doi: 10.3389/fnut.2018.00045 [published Online First: 2018/06/14]

82. Aoe S, Ikenaga T, Noguchi H, et al. Effect of cooked white rice with high β-glucan barley on appetite and energy intake in healthy Japanese subjects: a randomized controlled trial. *Plant foods for human nutrition (Dordrecht, Netherlands)* 2014;69(4):325-30. doi: 10.1007/s11130-014-0437-6 [published Online First: 2014/08/21]

83. Pletsch EA, Hamaker BR. Brown rice compared to white rice slows gastric emptying in humans. *European journal of clinical nutrition* 2018;72(3):367-73. doi: 10.1038/s41430-017-0003-z [published Online First: 2017/12/14]

84. Pletsch EA, Hayes AMR, Chegeni M, et al. Matched whole grain wheat and refined wheat milled products do not differ in glycemic response or gastric emptying in a randomized, crossover trial. *The American journal of clinical nutrition* 2022;115(4):1013-26. doi: 10.1093/ajcn/nqab434 [published Online First: 2022/01/10]

85. Breen C, Ryan M, Gibney MJ, et al. Glycemic, insulinemic, and appetite responses of patients with type 2 diabetes to commonly consumed breads. *The Diabetes educator* 2013;39(3):376-86. doi: 10.1177/0145721713479675 [published Online First: 2013/03/14]

86. Moazzami AA, Shrestha A, Morrison DA, et al. Metabolomics reveals differences in postprandial responses to breads and fasting metabolic characteristics associated with postprandial insulin demand in postmenopausal women. *The Journal of nutrition* 2014;144(6):807-14. doi: 10.3945/jn.113.188912 [published Online First: 2014/04/11]

87. Östman JR, Müllner E, Eriksson J, et al. Glucose Appearance Rate Rather than the Blood Glucose Concentrations Explains Differences in Postprandial Insulin Responses between Wholemeal Rye and Refined Wheat Breads-Results from A Cross-Over Meal Study. *Molecular nutrition & food research* 2019;63(7):e1800959. doi: 10.1002/mnfr.201800959 [published Online First: 2019/01/13]

88. Elbalshy MM, Reynolds AN, Mete E, et al. Gelatinisation and milling whole-wheat increases postprandial blood glucose: randomised crossover study of adults with type 2 diabetes. *Diabetologia* 2021;64(6):1385-88. doi: 10.1007/s00125-021-05400-y [published Online First: 2021/03/08]

89. Hayes AMR, Gozzi F, Diatta A, et al. Some pearl millet-based foods promote satiety or reduce glycaemic response in a crossover trial. *The British journal of nutrition* 2021;126(8):1168-78. doi: 10.1017/s0007114520005036 [published Online First: 2020/12/15]

90. McSweeney MB, Ferenc A, Smolkova K, et al. Glycaemic response of proso millet-based (Panicum miliaceum) products. *International journal of food sciences and nutrition* 2017;68(7):873-80. doi: 10.1080/09637486.2017.1301890 [published Online First: 2017/03/16]

**Not relevant outcomes**

91. Wu H, Kolehmainen M, Mykkänen H, et al. Alkylresorcinols in adipose tissue biopsies as biomarkers of whole-grain intake: an exploratory study of responsiveness to advised intake over 12 weeks. *European journal of clinical nutrition* 2015;69(11):1244-8. doi: 10.1038/ejcn.2015.138 [published Online First: 2015/09/04]

92. Rouhani MH, Mortazavi Najafabadi M, Surkan PJ, et al. The impact of oat (Avena sativa) consumption on biomarkers of renal function in patients with chronic kidney disease: A parallel randomized clinical trial. *Clinical nutrition (Edinburgh, Scotland)* 2018;37(1):78-84. doi: 10.1016/j.clnu.2016.11.022 [published Online First: 2016/12/23]

93. Katz DL, Evans MA, Chan W, et al. Oats, antioxidants and endothelial function in overweight, dyslipidemic adults. *Journal of the American College of Nutrition* 2004;23(5):397-403. doi: 10.1080/07315724.2004.10719384 [published Online First: 2004/10/07]

94. Peräaho M, Kaukinen K, Mustalahti K, et al. Effect of an oats-containing gluten-free diet on symptoms and quality of life in coeliac disease. A randomized study. *Scandinavian journal of gastroenterology* 2004;39(1):27-31. doi: 10.1080/00365520310007783 [published Online First: 2004/03/03]

95. Ross AB, Pere-Trépat E, Montoliu I, et al. A whole-grain-rich diet reduces urinary excretion of markers of protein catabolism and gut microbiota metabolism in healthy men after one week. *The Journal of nutrition* 2013;143(6):766-73. doi: 10.3945/jn.112.172197 [published Online First: 2013/04/26]

96. Jung SJ, Oh MR, Park SH, et al. Effects of rice-based and wheat-based diets on bowel movements in young Korean women with functional constipation. *European journal of clinical nutrition* 2020;74(11):1565-75. doi: 10.1038/s41430-020-0636-1 [published Online First: 2020/04/24]

97. van Trijp MPH, Schutte S, Esser D, et al. Minor Changes in the Composition and Function of the Gut Microbiota During a 12-Week Whole Grain Wheat or Refined Wheat Intervention Correlate with Liver Fat in Overweight and Obese Adults. *The Journal of nutrition* 2021;151(3):491-502. doi: 10.1093/jn/nxaa312 [published Online First: 2020/11/15]

98. Enright L, Slavin J. No effect of 14 day consumption of whole grain diet compared to refined grain diet on antioxidant measures in healthy, young subjects: a pilot study. *Nutrition journal* 2010;9:12. doi: 10.1186/1475-2891-9-12 [published Online First: 2010/03/23]

99. Jacobs DR, Jr., Pereira MA, Stumpf K, et al. Whole grain food intake elevates serum enterolactone. *The British journal of nutrition* 2002;88(2):111-6. doi: 10.1079/bjnbjn2002601 [published Online First: 2002/07/30]

100. Holma R, Hongisto SM, Saxelin M, et al. Constipation is relieved more by rye bread than wheat bread or laxatives without increased adverse gastrointestinal effects. *The Journal of nutrition* 2010;140(3):534-41. doi: 10.3945/jn.109.118570 [published Online First: 2010/01/22]

101. Gråsten SM, Juntunen KS, Poutanen KS, et al. Rye bread improves bowel function and decreases the concentrations of some compounds that are putative colon cancer risk markers in middle-aged women and men. *The Journal of nutrition* 2000;130(9):2215-21. doi: 10.1093/jn/130.9.2215 [published Online First: 2000/08/26]

**Not sufficient information**

102. Carvalho-Wells AL, Helmolz K, Nodet C, et al. Determination of the in vivo prebiotic potential of a maize-based whole grain breakfast cereal: a human feeding study. *The British journal of nutrition* 2010;104(9):1353-6. doi: 10.1017/s0007114510002084 [published Online First: 2010/05/22]

**Investigating the effect of whole grain intake along with reduction of antihypertensive medications**

103. Pins JJ, Geleva D, Keenan JM, et al. Do whole-grain oat cereals reduce the need for antihypertensive medications and improve blood pressure control? *The Journal of family practice* 2002;51(4):353-9. [published Online First: 2002/04/30]

**Shorter than 2 weeks**

104. Stringer DM, Taylor CG, Appah P, et al. Consumption of buckwheat modulates the post-prandial response of selected gastrointestinal satiety hormones in individuals with type 2 diabetes mellitus. *Metabolism: clinical and experimental* 2013;62(7):1021-31. doi: 10.1016/j.metabol.2013.01.021 [published Online First: 2013/03/15]

**Comparison of new and old wheat varieties**

105. Sofi F, Ghiselli L, Cesari F, et al. Effects of short-term consumption of bread obtained by an old Italian grain variety on lipid, inflammatory, and hemorheological variables: an intervention study. *Journal of medicinal food* 2010;13(3):615-20. doi: 10.1089/jmf.2009.0092 [published Online First: 2010/05/05]

106. Cicero AFG, Fogacci F, Veronesi M, et al. Short-Term Hemodynamic Effects of Modern Wheat Products Substitution in Diet with Ancient Wheat Products: A Cross-Over, Randomized Clinical Trial. *Nutrients* 2018;10(11) doi: 10.3390/nu10111666 [published Online First: 2018/11/08]

107. Trozzi C, Raffaelli F, Vignini A, et al. Evaluation of antioxidative and diabetes-preventive properties of an ancient grain, KAMUT(®) khorasan wheat, in healthy volunteers. *European journal of nutrition* 2019;58(1):151-61. doi: 10.1007/s00394-017-1579-8 [published Online First: 2017/11/17]

108. Sofi F, Whittaker A, Gori AM, et al. Effect of Triticum turgidum subsp. turanicum wheat on irritable bowel syndrome: a double-blinded randomised dietary intervention trial. *The British journal of nutrition* 2014;111(11):1992-9. doi: 10.1017/s000711451400018x [published Online First: 2014/02/14]

109. Sereni A, Cesari F, Gori AM, et al. Cardiovascular benefits from ancient grain bread consumption: findings from a double-blinded randomized crossover intervention trial. *International journal of food sciences and nutrition* 2017;68(1):97-103. doi: 10.1080/09637486.2016.1216528 [published Online First: 2016/10/01]

**Examining the effect of Job’s tears extract**

110. Djaja N. The Effect of Adding Job's Tears to Yogurt on Plasma Glycated Albumin, Weight, and Lipid Profile in Patients with Type 2 Diabetes Mellitus: A Randomized Controlled Trial. *Journal of nutrition and metabolism* 2022;2022:1876731. doi: 10.1155/2022/1876731 [published Online First: 2022/08/10]

### Supplementary Table 7: Characteristics of included studies on the effect of whole grain on the CMRFs in adults aged >18 years

| **Author** | **Location** | **Design** | **n** | **Health condition** | **BMI** | **Age, year** | **Intervention** | **Comparator** | **Duration**  **(week)** | **Background diet** | **Run-in period** | **Calorie restriction** | **Physical activity** | **Behavioral support** |
| --- | --- | --- | --- | --- | --- | --- | --- | --- | --- | --- | --- | --- | --- | --- |
| Zhang et al. 2011 | China | Randomized parallel | 202 | Metabolic syndrome | 25.6 | 49.7 | Brown rice | White rice | 16 | Brown rice is seldom available in Chinese markets | No | No | No | No |
| Zhang et al. 2012 | China | Randomized parallel | 166 | Hypercholesterolemia | 25.5 | 53.2 | Oatmeal | Wheat noodle | 6 | None of participants habitually consumed oat products. | No | No | No | No |
| Araki et al. 2017 | Japan | Randomized parallel | 41 | Prediabetes | 27.4 | 53.8 | Partially-abraded brown rice | White rice | 12 | NR | No | No | No | No |
| Geng et al. 2016 | China | Randomized parallel | 191 | Hypercholesterolemia | 25.7 | 56.7 | Pre-germinated brown rice | White rice | 12 | Participants had mean brown rice intake of<15g/d. | No | No | No | No |
| Gulati et al. 2017 | India | Randomized parallel | 80 | Hypercholesterolemia | 25.9 | 31.2 | Oat | Usual diet | 4 | NR | No | No | No | No |
| Ampatzoglou et al. 2015 | UK | Randomized  crossover | 33 | Healthy | 27.9 | 48.8 | Whole grain | Refined grain | 6 | Participants had habitual whole grain intake of <24 g/d. | Yes | No | No | No |
| Ampatzoglou et al. 2016 | UK | Randomized  crossover | 33 | Healthy | 27.9 | 48.8 | Whole grain | Refined grain | 6 | Participants had habitual whole grain intake of <24 g/d. | Yes | No | No | No |
| Juntunen et al. 2003 | Finland | Randomized  crossover | 20 | Hypercholesterolemia | 27.5 | 59 | High-fiber rye bread | White wheat bread | 8 | NR | Yes | No | No | No |
| Moazzami et al. 2012 | Finland | Randomized  crossover | 33 | Hypercholesterolemia | 27.2 | 58.8 | High-fiber rye bread | White wheat bread | 8 | NR | Yes | No | No | No |
| Ma et al. 2013 | China | Randomized parallel | 184 | Type 2 diabetes | 26.7 | 59.7 | Oat-whole grain+ Healthy diet | Healthy diet | 4 | Participants had no oats or oat products | Yes | No | No | Yes |
| Li et al. 2016 | China | Randomized parallel | 238 | Type 2 diabetes | 27.1 | 59.6 | Oat-whole grain+ Healthy diet | Healthy diet | 48 | Participants had no oats or oat products | Yes | No | No | Yes |
| Schutte et al. 2018 | Netherlands | Randomized parallel | 50 | Overweight and hypercholesterolemia | 27.8 | 61 | Whole grain wheat | Refined grain | 12 | NR | Yes | No | No | No |
| Hoevenaars et al. 2019 | Netherlands | Randomized parallel | 50 | Hypercholesterolemia | 27.8 | 61 | Whole grain Wheat | Refined grain | 12 | NR | Yes | No | No | No |
| Ding et al. 2022 | China | Randomized parallel | 85 | Type 2 diabetes | 25.6 | 64.8 | Germinated brown rice | White rice | 12 | NR | No | No | No | Yes |
| Wan et al. 2023 | China | Randomized parallel | 67 | Type 2 diabetes | 25.6 | 64.8 | Germinated brown rice | White rice | 12 | NR | No | No | No | Yes |
| Lankinen et al. 2014 | Finland | Randomized parallel | 69 | Overweight or obesity | 31.2 | 58.5 | Whole grain | Refined grain | 12 | NR | Yes | No | No | No |
| Lankinen et al. 2011 | Finland | Randomized parallel | 69 | Overweight or obesity | 31.2 | 58.5 | Whole grain | Refined grain | 12 | NR | Yes | No | No | No |
| de Mello et al.  2011 | Finland | Randomized parallel | 68 | Overweight or obesity | 31.2 | 58.5 | Whole grain | Refined grain | 12 | NR | Yes | No | No | No |
| Thongoun et al. 2013 | Thailand | Randomized  crossover | 24 | Hypercholesterolemia | 26.7 | 51 | Oatmeal | White rice | 4 | NR | No | No | No | No |
| Pavadhgul et al. 2019 | Thailand | Randomized crossover | 24 | Hypercholesterolemia | 23-30 | 30-60 | Oatmeal | White rice | 4 | NR | No | No | No | No |
| Giacco et al. 2013 | Finland | Randomized parallel | 123 | Metabolic syndrome | 31.5 | 57.2 | Whole grain cereal | Refined cereal | 12 | NR | Yes | No | No | No |
| Giacco et al. 2014 | Finland | Randomized parallel | 54 | Metabolic syndrome | 31.8 | 57.2 | Whole grain cereal | Refined cereal | 12 | NR | Yes | No | No | No |
| MacKay et al. 2012 | Canada | Randomized crossover | 28 | Normoglycemia/normoinsulinemia and hyperglycemia/hyperinsulinemia | 31.1 | 55.2 | Whole wheat sourdough | White wheat | 6 | NR | No | No | No | No |
| Tucker et al. 2010 | Canada | Randomized crossover | 28 | Normoglycemia/normoinsulinemia and hyperglycemia/hyperinsulinemia | 31.1 | 55.2 | Whole wheat sourdough | White wheat | 6 | NR | No | No | No | No |
| Karl et al. 2017 | US | Randomized parallel | 81 | Healthy | 25.7 | 54.5 | Whole grain | Refined grain | 6 | Participants had mean total plasma alkylresorcinols of <30 nmol/L. | Yes | No | No | No |
| Vanegas et al. 2017 | US | Randomized parallel | 79 | Healthy | 26 | 54.5 | Whole grain | Refined grain | 6 | Participants had mean total plasma alkylresorcinols of <30 nmol/L. | Yes | No | No | No |
| Xu et al. 2021 | China | Randomized parallel | 187 | Hypercholesterolemia | 23.3 | 48.9 | Oat meal | White rice | 6 | NR | No | No | No | No |
| Xu et al. 2021 | China | Randomized parallel | 62 | Hypercholesterolemia | 22.9 | 44.7 | Oat meal | White rice | 6 | NR | No | No | No | No |
| Iversen et al. 2021 | Sweden | Randomized parallel | 242 | Overweight or obesity | 30 | 56.7 | Rye | White wheat | 12 | NR | Yes | Yes | No | Yes |
| Kikuchi et al. 2018 | Japan | Randomized parallel | 49 | Healthy | 27.4 | 47.5 | Whole wheat | White wheat | 12 | Participants with continuous intake of dietary fiber supplements or whole grain were excluded | No | No | No | No |
| Kim et al. 2008 | Korea | Randomized parallel | 40 | Overweight or obesity | 27.4 | 20-35 | Brown and black rice | White rice | 6 | NR | No | Yes | No | No |
| Mai et al. 2020 | Vietnam | Randomized parallel | 80 | Metabolic syndrome | 25.7 | 65.1 | Pre-germinated brown rice | White rice | 12 | Participants with daily intake of pre-germinated brown rice were excluded | No | Yes | No | No |
| Malik et al. 2019 | India | Randomized  crossover | 121 | Overweight | 28.1 | 37.1 | Brown rice | White rice | 12 | Participants had mean whole grain consumption of <15g/d. | Yes | No | No | No |
| Suhr et al. 2017 | Denmark | Randomized parallel | 69 | Overweight | 27.9 | 51 | Whole wheat/whole rye | Refined grain | 6 | Participants had median total plasma alkylresorcinols of <50 nmol/L. | No | No | No | No |
| Kazemzadeh et al. 2014 | Iran | Randomized  crossover | 35 | Overweight or obesity | 29.3 | 32.6 | Brown rice | White rice | 6 | NR | Yes | No | No | No |
| Navarro-Perez et al. 2017 | Australia | Randomized parallel | 50 | Overweight or obesity | 31.4 | 37.9 | Quinoa | Usual diet | 12 | NR | No | No | No | No |
| Reiners et al. 2023 | Germany | Randomized  crossover | 32 | Hypercholesterolemia | 25.7 | 51 | Roasted and traditional oat/barley flakes | White wheat bread | 3 | NR | No | No | No | No |
| Shimabukuro et al. 2014 | Japan | Randomized  crossover | 27 | Metabolic syndrome | 26.7 | NR | Brown rice | White rice | 8 | NR | No | No | No | No |
| Van Horn et al. 1991 | US | Randomized parallel | 80 | Hypercholesterolemia | 26.2 | 42.5 | Oat meal | Usual diet | 8 | NR | No | No | No | No |
| Wang et al. 2013 | US | Randomized parallel | 57 | Prediabetes | 25.7 | 52.5 | Brown rice | White rice | 12 | Participants had a regular intake of white rice | No | No | No | No |
| Maki et al. 2010 | US | Randomized parallel | 173 | Overweight or obesity | 32.1 | 48.8 | Oat | Low-fiber cereal | 12 | Participants with daily intake of oat or barley products were excluded | No | Yes | Yes | Yes |
| Kristensen et al. 2012 | Denmark | Randomized parallel | 72 | Overweight or obesity | 30.2 | 59.7 | Whole grain wheat | Refined grain | 12 | Participants had mean total plasma alkylresorcinols of <50 nmol/L. | Yes | Yes | No | Yes |
| Katcher et al. 2008 | US | Randomized parallel | 50 | Metabolic syndrome | 35.8 | 46 | Whole grain | Refined grain | 12 | Participants had habitual whole grain intake of <3 servings/d. | No | Yes | Yes | Yes |
| Harris Jackson et al. 2014 | US | Randomized parallel | 50 | Overweight or obesity | 33.2 | 46.1 | Whole grain | Refined grain | 12 | Participants had mean total plasma alkylresorcinols of <50 nmol/L. | No | Yes | No | No |
| Akamine et al. 2022 | Japan | Randomized parallel | 40 | Metabolic syndrome | 28.8 | 57.6 | Fermented brown rice | Fermented white rice | 4 | NR | No | No | No | No |
| Kopf et al. 2018 | US | Randomized parallel | 31 | Overweight or obesity | 32 | 33.9 | Whole grain | Refined grain | 6 | Participants had low intake of fruit, vegetables, and whole grains. | No | No | No | No |
| Meng et al. 2018 | US | Randomized  crossover | 11 | Hypercholesterolemia | 29.8 | 65 | Unrefined carbohydrates | Refined carbohydrates | 4.5 | NR | No | No | No | No |
| Tighe et al. 2010 | Scotland | Randomized parallel | 206 | Healthy | 27.7 | 51.8 | Whole-wheat | Refined grain | 12 | Participants with high habitual intake of whole grains were excluded. | Yes | No | No | No |
| Durazzo et al. 2014 | Italy | Randomized  crossover | 13 | Healthy | 26.4 | 52.8 | Whole grain foods high in lignans | Refined grain | 4 | NR | No | No | No | No |
| Pushpass et al. 2023 | Ireland | Randomized parallel | 30 | Healthy | 24.8 | 49.5 | Whole rolled oats +  semi-skimmed milk  + 2placebo capsules | Cornflakes  +  semi-skimmed milk  +2 placebo capsules | 8 | NR | No | No | No | No |
| Dorosti et al. 2020 | Iran | Randomized parallel | 94 | NAFLD | 32.2 | 43 | Whole grain | Usual cereals | 12 | None of participants consumed whole grains. | No | No | No | No |
| Hsu et al. 2008 | Taiwan | Randomized  crossover | 11 | Type 2 diabetes | 25.1 | 51.5 | Pre-germinated brown rice | White rice | 6 | NR | No | No | No | No |
| Li et al. 2018 | China | Randomized  crossover | 28 | Overweight | 27.7 | 51.5 | Quinoa bread | White wheat bread | 4 | NR | No | No | No | No |
| Qiu et al. 2016 | China | Randomized parallel | 165 | Type 2 diabetes | 26.8 | 56.8 | Tartary buckwheat | White rice and white wheat flour | 4 | NR | No | No | No | Yes |
| Saltzman et al. 2001 | US | Randomized parallel | 43 | Healthy | 26.4 | 44.6 | Oat | Usual diet | 6 | NR | No | Yes | No | No |
| Pereira et al. 2002 | US | Randomized  crossover | 11 | Overweight or obesity | 30.2 | 41.6 | Whole grain | Refined grain | 6 | NR | No | No | No | No |
| Brownlee et al. 2010 | UK | Randomized parallel | 266 | Overweight or obesity | 30 | 46 | Whole grain | Usual diet | 16 | Participants had habitual whole grain intake of <30 g/d. | No | No | No | No |
| Aoe et al. 2018 | Japan | Randomized parallel | 60 | Healthy | 21.4 | 46.5 | BARLEYmax+  Wheat bran | Wheat bran | 4 | Participants had low fiber intake (<12 g/d). | No | No | No | No |
| Bodinham et al. 2011 | UK | Randomized  crossover | 14 | Healthy | 21.8 | 26 | Whole wheat bread | Refined-grain bread | 3 | Participants had median whole grain intake of 15.4 g/d. | No | No | No | No |
| Esmaeilpour et al. 2019 | Iran | Randomized parallel | 76 | Premenstrual syndrome | 25.3 | 31 | Whole grain | Refined grain | 12 | Participants had mean whole grain intake of <1 serving/d | No | No | No | No |
| Nazari et al. 2021 | Iran | Randomized parallel | 60 | Type 2 diabetes | 29.1 | 53.8 | Whole grain bread (Sangak) | Usual diet | 8 | NR | No | No | No | No |
| Cooper et al. 2017 | US | Randomized parallel | 45 | Healthy | 23.4 | 25.8 | Whole grain | Refined grain | 6 | Participants had habitual whole grain intake of <1 serving/d | No | No | No | No |
| Na et al. 2023 | China | Randomized parallel | 85 | Type 2 diabetes | 25.6 | 65.8 | Germinated brown rice | Refined rice | 12 | NR | No | No | No | Yes |
| Li et al. 2003 | Japan | Randomized  crossover | 10 | Healthy | 19.2 | 20.4 | Barley diet | Standard diet | 4 | NR | No | No | No | No |
| Van Horn et al. 1988 | US | Randomized parallel | 236 | Healthy | NR | 42.4 | Oat  +  Fat-modified diet | Fat-modified diet | 8 | NR | No | No | No | Yes |
| Ross et al. 2011 | Switzerland | Randomized  crossover | 17 | Healthy | 23.6 | 35.3 | Whole grain diet | Refined grain diet | 2 | Participants had habitual whole grain intake of <30g/d | No | No | No | No |
| Maier et al. 2000 | US | Randomized parallel | 56 | Hypercholesterolemia | 25.5 | 48 | Amaranth+  NCEP diet | NCEP diet | 4 | NR | No | No | No | Yes |
| Keenan et al. 2002 | US | Randomized parallel | 22 | Hypertension | NR | 44 | Oat cereal | Low-fiber cereal | 6 | NR | No | No | No | No |
| Nakayama et al. 2017 | Japan | Randomized  crossover | 16 | Type 2 diabetes | 25.7 | 64 | Glutenius brown rice | White rice | 8 | None of participants consumed brown rice on a daily basis. | Yes | No | No | No |
| Nakayama et al. 2020 | Japan | Randomized parallel | 41 | Healthy | 21.6 | 44.3 | Glutinous brown rice | White rice | 6 | Participants consuming white rice or white bread as part of their regular diet | No | No | No | No |
| Roager et al. 2017 | Denmark | Randomized crossover | 50 | Overweight or obesity | NR | 48.6 | Whole grain diet | Refined grain diet | 8 | Participants had mean whole grain intake of 68g/day. | No | No | No | No |
| Melanson et al.  2006 | US | Randomized parallel | 91 | Overweight or obesity | 30.8 | 42.1 | Whole grain cereals  +  hypocaloric diet | Hypocaloric diet | 24 | Participants consuming whole-grain cereals for breakfast >2 times/week were excluded | Yes | Yes | Yes | No |
| Kondo et al. 2017 | Japan | Randomized parallel | 28 | Type 2 diabetes | 24.6 | 66.6 | Brown rice | White rice | 8 | Participants consuming brown rice or dietary fiber supplements were excluded | Yes | No | No | No |
| Vitaglione et al. 2014 | Italy | Randomized parallel | 68 | Overweight or obesity | 29.8 | 38.5 | Whole grain wheat products | Refined wheat products | 8 | Participants did not habitually consume whole-grain cereals | No | No | No | No |
| Kristensen et al. 2017 | France | Randomized parallel | 169 | Overweight or obesity | 30.1 | 35.7 | Whole grain diet | Refined grain diet | 12 | Participants had habitual whole grain intake of <16g/d | Yes | No | No | Yes |
| Kirwan et al. 2016 | US | Randomized crossover | 33 | Overweight or obesity | 33.1 | 39 | Whole grain diet | Refined grain diet | 8 | NR | No | No | No | No |
| Andersson et al. 2007 | Sweden | Randomized crossover | 30 | Overweight | 28.3 | 59 | Whole grain diet | Refined grain diet | 6 | NR | No | No | No | No |
| Reynold et al. 2000 | US | Randomized parallel | 43 | Hypercholesterolemia | 24.4 | 51.6 | Oat | Refined corn flakes | 6 | NR | No | No | No | No |
| Davidson et al. 1991 | US | Randomized parallel | 76 | Hypercholesterolemia | 25.6 | 52.5 | Oat meal | Farina | 6 | NR | No | No | No | No |
| Connolly et al. 2016 | UK | Randomized crossover | 30 | Hypercholesterolemia or glucose intolerance | 26.4 | 42 | Whole grain oat granola | Refined cereal | 6 | NR | No | No | No | No |
| Johnston et al. 1998 | US | Randomized parallel | 124 | Hypercholesterolemia | NR | 57 | Oat | Refined corn flakes | 6 | NR | Yes | No | No | No |
| Kuroda et al. 2019 | Japan | Randomized parallel | 34 | Healthy elderly | 23.6 | 75 | UHHP brown rice | White rice | 96 | NR | No | No | No | No |
| Charlton et al. 2012 | Australia | Randomized parallel | 61 | Hypercholesterolemia & Overweight | 27.26 | 51 | Oat porridge and cereal bars | Cornflakes, puffed rice and wheat bars | 6 | NR | No | No | No | Yes |
| Leinonen et al. 2000 | Finland | Randomized crossover | 40 | Hypercholesterolemia | 24.5 | 43 | Whole rye bread | White wheat bread | 4 | NR | No | No | No | No |
| Ren et al. 2020 | China | Randomized parallel | 56 | Metabolic syndrome | ≥ 22 | 18-70 | Brown rice | Polished rice | 4 | NR | No | No | No | No |
| Joo et al. 2020 | Korea | Randomized parallel | 49 | Metabolic syndrome | 29.3 | 44.3 | Black rice | White rice | 12 | NR | No | No | No | No |
| Laborde et al. 2021 | Philippines | Randomized parallel | 52 | Overweight or obesity | 27.1 | 30-59 | Brown rice | White rice | 6 | None of participants consumed brown rice and/or pigmented rice. | No | Yes | No | No |
| Liao et al. 2018 | Taiwan | Randomized parallel | 74 | Hypercholesterolemia or healthy | 23.5 | 38-76 | Oat noodles | Wheat noodles | 10 | NR | No | No | No | No |
| Chang et al. 2013 | Taiwan | Randomized parallel | 34 | Overweight or obesity | 29.4 | 38.5 | Oat | Control | 12 | NR | No | No | No | No |
| Gilhooly et al. 2008 | US | Randomized parallel | 33 | Overweight | 27.5 | 34.6 | High fiber cereal + Caloric restriction | Caloric restriction | 6 | NR | No | Yes | No | Yes |
| Karmally et al. 2005 | US | Randomized parallel | 152 | Hypercholesterolemia | 29.2 | 49 | Oat cereal | Refined Corn cereal | 6 | NR | Yes | No | No | No |
| Xue et al. 2021 | China | Randomized parallel | 158 | Helicobacter pylori | 24.2 | 45 | High-fiber wholegrain rye foods | Refined wheat foods | 12 | Participants had median total plasma alkylresorcinols of <3 nmol/L. | No | No | No | No |
| De Carvalho et al. 2013 | Brazil | Randomized parallel | 35 | Overweight | 28.8 | 61 | Quinoa flakes | Corn flakes | 4 | NR | No | No | No | No |
| Zeng et al. 2023 | China | Randomized parallel | 133 | Prediabetes | 24.3 | 56.5 | Quinoa | Usual diet | 48 | NR | No | No | No | Yes |
| Van Horn et al. 1986 | US | Randomized parallel | 139 | Healthy | 25.2 | 44.8 | Oat meal+ Fat-modified diet | Fat-modified diet | 6 | NR | No | No | No | No |
| Qiu et al. 2016 | China | Randomized parallel | 104 | Type 2 diabetes | 26.8 | 58.8 | Tartary buckwheat | White rice or wheat flour | 4 | NR | No | No | No | Yes |
| Peräaho et al. 2004 | Finland | Randomized parallel | 39 | Patients with coeliac | NR | 22-69 | Oat+ gluten-free diet | gluten-free diet | 48 | NR | No | No | No | No |
| Ye et al. 2020 | China | Randomized parallel | 28 | Hypercholesterolemia | 23.5 | 47.5 | Oat meal | White rice | 6 | NR | No | No | No | No |
| Li et al. 2024 | China | Randomized parallel | 120 | Hypertension or healthy | 26 | 63.7 | Brown rice and whole grain flour | Wheat flour and refined rice | 6 | NR | No | No | No | No |
| Liedike et al. 2024 | US | Randomized crossover | 36 | Hypercholesterolemia | 28.4 | 39.8 | Whole grain corn meal | Refined grain corn meal | 4 | NR | No | No | No | No |
| Khoramnazari et al. 2017 | Iran | Randomized parallel | 76 | Overweight or obesity | 29.6 | 60.4 | Whole Wheat | White bread | 12 | Participants had habitual whole grain products intake of <3 serving/d | No | Yes | No | Yes |

Abbreviations: CMRFs: cardiometabolic risk factors- NCEP: National Cholesterol Education Program- AHA: American Heart Association- US: United States- UK: United Kingdom- UHHP: ultra-high hydrostatic pressurizing- NR: not-reported

*Presented as mean, median, or range

### Supplementary Table 8: Risk‑of‑bias assessment of the included studies according to Version 2 of the Cochrane risk‑of‑bias tool

| **Author** | **Randomization process** | **Deviations from intended interventions** | **Missing outcome data** | **Measurement of the outcome** | **Selection of the reported result** | **Overall risk of bias ^1^** |
| --- | --- | --- | --- | --- | --- | --- |
| Zhang et al. 2011 | S | H | L | L | L | High risk |
| Zhang et al. 2012 | S | H | H | S | L | High risk |
| Araki et al. 2017 | S | H | L | S | L | High risk |
| Geng et al. 2016 | S | H | H | S | L | High risk |
| Gulati et al. 2017 | S | H | L | S | L | High risk |
| Ampatzoglou et al. 2015 | L | H | L | S | L | High risk |
| Ampatzoglou et al. 2016 | L | H | L | S | L | High risk |
| Juntunen et al. 2003 | S | H | L | S | L | High risk |
| Moazzami et al. 2012 | S | H | L | S | L | High risk |
| Ma et al. 2013 | S | H | L | L | L | High risk |
| Li et al. 2016 | S | H | L | L | L | High risk |
| Schutte et al. 2018 | L | L | L | L | L | Low risk |
| Hoevenaars et al. 2019 | L | L | L | L | L | Low risk |
| Ding et al. 2022 | S | H | H | S | L | High risk |
| Wan et al. 2023 | S | H | L | S | L | High risk |
| Lankinen et al. 2014 | S | H | H | S | L | High risk |
| Lankinen et al. 2011 | S | H | H | S | L | High risk |
| de Mello et al. 2011 | S | H | H | L | L | High risk |
| Thongoun et al. 2013 | S | H | L | S | L | High risk |
| Pavadhgul et al. 2019 | S | H | L | S | L | High risk |
| Giacco et al. 2013 | L | H | L | L | L | High risk |
| Giacco et al. 2014 | L | H | L | L | L | High risk |
| MacKay et al. 2012 | L | H | L | S | L | High risk |
| Tucker et al. 2010 | L | H | L | S | L | High risk |
| Karl et al. 2017 | L | H | L | L | L | High risk |
| Vanegas et al. 2017 | L | H | L | L | L | High risk |
| Xu et al. 2021 | L | H | S | L | L | High risk |
| Xu et al. 2021 | L | H | L | L | L | High risk |
| Iversen et al. 2021 | L | H | L | L | L | High risk |
| Kikuchi et al. 2018 | S | L | L | S | L | Some concerns |
| Kim et al. 2008 | S | H | L | S | L | High risk |
| Mai et al. 2020 | S | H | L | S | L | High risk |
| Malik et al. 2019 | S | H | H | L | L | High risk |
| Suhr et al. 2017 | L | H | L | L | S | High risk |
| Kazemzadeh et al. 2014 | **S** | H | L | S | L | High risk |
| Navarro-Perez et al. 2017 | L | H | H | L | L | High risk |
| Reiners et al. 2023 | S | L | L | S | L | Some concerns |
| Shimabukuro et al. 2014 | S | H | L | S | L | High risk |
| Van Horn et al. 1991 | S | H | H | S | L | High risk |
| Wang et al. 2013 | S | H | H | S | L | High risk |
| Maki et al. 2010 | S | H | H | S | S | High risk |
| Kristensen et al. 2012 | S | H | L | S | L | High risk |
| Katcher et al. 2008 | S | H | L | S | L | High risk |
| Harris Jackson et al. 2014 | S | H | H | L | L | High risk |
| Akamine et al. 2022 | S | H | L | S | L | High risk |
| Kopf et al. 2018 | L | H | L | S | L | High risk |
| Meng et al. 2018 | L | H | L | L | L | High risk |
| Tighe et al. 2010 | S | H | H | S | L | High risk |
| Durazzo et al. 2014 | L | L | L | S | S | Some concerns |
| Pushpass et al. 2023 | L | H | L | L | L | High risk |
| Dorosti et al. 2020 | L | H | H | L | L | High risk |
| Hsu et al. 2008 | S | H | L | L | L | High risk |
| Li et al. 2018 | S | H | H | L | L | High risk |
| Qiu et al. 2016 | S | H | H | L | L | High risk |
| Saltzman et al. 2001 | S | H | L | S | L | High risk |
| Pereira et al. 2002 | S | H | L | S | L | High risk |
| Brownlee et al. 2010 | L | H | H | S | L | High risk |
| Aoe et al. 2018 | S | L | L | S | L | Some concerns |
| Bodinham et al. 2011 | S | H | L | S | L | High risk |
| Esmaeilpour et al. 2019 | S | H | H | S | L | High risk |
| Nazari et al. 2021 | S | H | L | S | L | High risk |
| Cooper et al. 2017 | S | H | L | S | L | High risk |
| Na et al. 2023 | S | H | H | S | L | High risk |
| Li et al. 2003 | S | H | L | S | L | High risk |
| Van Horn et al. 1988 | S | H | S | S | L | High risk |
| Ross et al. 2011 | S | H | H | S | L | High risk |
| Maier et al. 2000 | S | H | L | S | L | High risk |
| Keenan et al. 2002 | S | H | H | L | L | High risk |
| Nakayama et al. 2017 | S | H | L | S | S | High risk |
| Nakayama et al. 2020 | L | H | L | S | L | High risk |
| Roager et al. 2017 | L | H | L | L | L | High risk |
| Melanson et al. 2006 | L | H | H | S | S | High risk |
| Kondo et al. 2017 | L | H | L | S | L | High risk |
| Vitaglione et al. 2014 | S | H | L | L | L | High risk |
| Kristensen et al. 2017 | S | H | L | L | L | High risk |
| Kirwan et al. 2016 | L | S | L | S | L | Some concerns |
| Andersson et al. 2007 | S | H | L | H | L | High risk |
| Reynold et al. 2000 | S | L | L | S | L | Some concerns |
| Davidson et al. 1991 | S | H | L | S | L | High risk |
| Connolly et al. 2016 | L | L | L | L | L | Low risk |
| Johnston et al. 1998 | S | H | L | S | L | High risk |
| Kuroda et al. 2019 | S | H | H | S | L | High risk |
| Charlton et al. 2012 | L | H | L | S | S | High risk |
| Leinonen et al. 1999 | S | H | L | S | L | High risk |
| Ren et al. 2020 | S | H | L | S | L | High risk |
| Joo et al. 2020 | S | H | L | S | L | High risk |
| Laborde et al. 2021 | S | H | L | S | L | High risk |
| Liao et al. 2018 | S | L | L | S | L | Some concerns |
| Chang et al. 2013 | S | L | S | S | L | Some concerns |
| Gilhooly et al. 2008 | S | H | L | S | L | High risk |
| Karmally et al. 2005 | S | H | L | S | L | High risk |
| Xue et al. 2021 | S | H | H | L | L | High risk |
| De Carvalho et al. 2013 | S | L | L | S | L | Some concerns |
| Zeng et al. 2023 | S | H | H | S | L | High risk |
| Van Horn et al. 1986 | S | H | L | S | L | High risk |
| Qiu et al. 2016 | S | H | H | L | L | High risk |
| Peräaho et al. 2004 | L | H | L | S | L | High risk |
| Ye et al. 2020 | L | H | S | L | L | High risk |
| Li et al. 2024 | S | H | H | S | L | High risk |
| Liedike etal. 2024 | S | H | H | S | L | High risk |
| Khoramnazari et al. 2017 | S | H | S | S | L | High risk |

^1^ Studies are categorized as follows — Low risk of bias if judged to be at low risk for all domains; Some concerns if judged to raise concerns in at least one domain but not high risk in any; and High risk of bias if judged to be high risk in at least one domain, or to have multiple concerns that substantially lower confidence in the result.

Abbreviations: S; some concern, L; low risk of bias, H; high risk of bias

### Supplementary Table 9: Adverse events reported in the trials on the effect of whole grains on the cardiometabolic risk factors

| Nakayama et al. 2017 | No adverse events occurred in subjects who completed the study. |
| --- | --- |
| Nakayama et al. 2020 | There were no adverse effects in either group during the 6-week study period. |
| Pereira et al. 2002 | Unusual symptoms were reported more often on the daily questionnaire during the refined-grain than during the whole-grain diet period, and the difference appeared to be primarily due to common upper respiratory illnesses in 2 individuals and to chronic low-grade abdominal pain in another subject.  Dry cough and sweating tended to be scored significantly higher on the Likert scale with the refined-grain diet. Bowel movements were more frequent with the whole-grain than with the refined-grain diet. No significant differences between treatments were apparent for heartburn, indigestion, diarrhea, or loose stools. |
| Saltzman et al. 2001 | No gastrointestinal complaints were noted. |
| Van Horn et al. 1991 | There were no reports of side effects from eating the oats. |
| Vitaglione et al. 2014 | No adverse events were identified in whole grain and control groups over the study period. |
| Zhang et al. 2012 | There were no adverse events reported during the intervention period except for some complaints about the taste of the oat product. |
| Mai et al. 2020 | No subjects in either group had any symptoms related to the intake of test substances. |
| Kondo et al. 2017 | No adverse events were observed throughout the study period. |
| Kopf et al. 2018 | There were no significant changes in gastrointestinal symptoms throughout the study indicating that the treatments were well-tolerated |
| Kristensen et al. 2017 | No difference in taste preference or gastrointestinal comfort between the diets was reported by the participants, although the higher dropout rate in the whole grain group may indicate that the subjects were less willing to eat foods that were not part of their habitual diet. |
| Kuroda et al. 2019 | Side-effects that disrupt daily life of participants (such as allergic reactions, palpitations, and irritation in the stomach) were not present in either group. |
| Li et al. 2003 | There were no side effects in the subjects on the standard and barley diets. |
| Li et al. 2018 | Both breads were well tolerated by the subjects, without any apparent side effects or complaints. |
| Liao et al. 2018 | None of the subjects experience any adverse effect upon consumption with either oat (experimental) or wheat (placebo) noodles. |
| Vanegas et al. 2017 | Incidences of self-reported gastrointestinal symptoms did not differ between groups. |
| Karmally et al. 2005 | No serious side effects were reported in either group. |
| Keenan et al. 2002 | An analysis of the side effect data showed no significant difference in the occurrence of side effects between groups. There was an overall decrease in the frequency of dietary fiber-related and hypertension-related side effects in both groups, with a more substantial reduction occurring in the oat cereal group |
| Kim et al. 2008 | Research subjects experienced no side effects. |
| Geng et al. 2016 | There were no adverse events reported during the intervention period. |
| Aoe et al. 2018 | No adverse events corresponding to the intake of the test food, such as gastrointestinal problems, were observed during the trial. |
| Araki et al. 2017 | No adverse events related to the test meal were observed. |
| Chang et al. 2013 | Ingestion of oat was well tolerated and there was no adverse effect during the trial. |
| Charlton et al. 2012 | There were no reported adverse events. |
| Connolly et al. 2016 | The severity and frequency of reported changes in digestive tolerance varied greatly between volunteers, with neither treatment resulting in adverse symptomology. |
| Wan et al. 2023 | During the 3-month trial, some adverse events such as nausea, diarrhea, and headache did not occur. |
| Davidson et al. 1991 | There were no adverse effects. |
| Ampatzoglou et al. 2015 | There were no differences between interventions in the types, frequency, or duration of adverse events. |
| Ma et al. 2013 | Products were well tolerated by the participants. No serious adverse events were reported during the trial. Adverse events reported were generally hypoglycemia and mild gastrointestinal symptoms including diarrhea, gastrointestinal discomfort and constipation. Hypoglycemia usually occurred early during the study and was transient. The differences in the adverse events and safety parameters between the groups were insignificant. |
| Iversen et al. 2021 | For gastrointestinal symptoms, the groups did not differ in terms of abdominal pain or diarrhea. However, during the 12-week intervention, participants in the rye group felt more bloated and less constipated compared the participants in the wheat group. Nevertheless, it should be noted that both groups had relatively low symptom scores throughout the study, indicating that participants had relatively few symptoms both before and during the study. |
| Cooper et al. 2017 | No difference in self-reported gastrointestinal symptoms of bloating or gas, abdominal pain, nausea, flatulence, changes in stool, change in feelings about changes in stool, bowel movement frequency, and Bristol stool score between the interventions was reported by the participants. |
| Reynold et al. 2000 | The cereals were well tolerated with no serious adverse reactions reported in either treatment groups. The nonserious adverse reactions reported equally in both groups were mild flatus. |
| Johnston et al. 1998 | One subject on the control cereal discontinued because of gastrointestinal complaints. |
| Harris Jackson et al. 2014 | One participant had an adverse reaction to the whole grain diet. |
| Andersson et al. 2007 | Subjects did not report concurrent illnesses during either of the diet periods. Minor gastrointestinal symptoms during both dietary periods were reported by 5 subjects. |
| Maki et al. 2010 | The frequencies of adverse events of any type (whether or not related to the study products) were similar for both the whole-grain ready-to-eat (RTE) oat cereal and control groups. The most common adverse events in both groups were upper respiratory tract infection, sinusitis, and pharyngitis. Most adverse events were mild and not related to consumption of the study product. Adverse events considered at least possibly related to the study product included nausea (two whole-grain RTE oat cereal subjects), flatulence (two whole-grain RTE oat cereal participants), and gastroenteritis and gastroesophageal reflux (one control participant each). One participant in the control group experienced a serious adverse event (vomiting) that resolved and did not result in discontinuation from the trial. Adverse events that resulted in discontinuation of study participation included an infectious cyst and spinal stenosis (reported by one control participant each). Neither of these events was considered to be related to the study protocol. |
| Gilholy et al. 2008 | Over the 6-week fiber randomization, no subjects in the control group and 2 subjects in the high fiber cereal group reported gastrointestinal-related symptoms. |
| Brownlee et al. 2010 | Six study participants did not tolerate whole grain foods (three in each intervention group). |
| Ye et al. 2020 | Only two participants (both from the oatmeal group) reported various degrees of gastrointestinal reactions like bloating and frequent fart, yet all basically relieved within 3 weeks. |
| Akamine et al. 2022 | All subjects were obliged to report any serious or unexpected adverse events immediately to either the principal investigator and/or research ethics committee. However, the authors did not provide any report in the results section. |
| Malik et al. 2019 | Adverse effects of intervention meals were also ascertained monthly. However, the authors did not provide any report in the results section. |
| Leinonen et al. 2000 | Bowel function and possible side effects were recorded daily. However, the authors did not provide any report in the results section. |

### Supplementary Table 10: Pairwise sensitivity analyses using different correlation coefficients

| **Outcomes** | **Trials** | **Pooled MD (95% CI)*** | **I^2^ (%) (95% CI)†** |
| --- | --- | --- | --- |
|  | **Correlation Coefficient (0.3)** | | |
| **Primary outcomes** |  | | |
| LDL-C (mmol/L) | 69 | -0.134 (-0.175 to -0.093) | 32.1 (0.00 to 50.9) |
| Body weight (kg) | 60 | -0.441 (-0.641 to -0.241) | 16.4 (0.00 to 40.4) |
| FPG (mmol/L) | 62 | -0.066 (-0.120 to -0.012) | 54.8 (28.4 to 68.9) |
| **Secondary outcomes** |  |  |  |
| Waist circumference (cm) | 38 | -0.451 (-0.830 to -0.072) | 36.8 (0.00 to 60.3) |
| Body fat percentage | 29 | -0.194 (-0.411 to 0.024) | 0.00 (0.00 to 36.3) |
| Insulin (pmol/L) | 42 | -0.274 (-2.445 to 1.898) | 0.00 (0.00 to 6.90) |
| HOMA-IR | 30 | -0.201 (-0.336 to -0.066) | 7.70 (0.00 to 41.5) |
| HbA_1c_ (%) | 24 | -0.038 (-0.082 to 0.007) | 30.9 (0.00 to 59.8) |
| Triglycerides (mmol/L) | 70 | -0.055 (-0.089 to -0.021) | 28.5 (0.00 to 51.1) |
| Total cholesterol (mmol/L) | 73 | -0.163 (-0.211 to -0.116) | 38.9 (9.30 to 56.0) |
| HDL-C (mmol/L) | 71 | 0.002 (-0.018 to 0.022) | 62.2 (35.8 to 75.1) |
| SBP (mm Hg) | 46 | -1.532 (-2.654 to -0.410) | 43.3 (4.90 to 62.4) |
| DBP (mm Hg) | 45 | -0.907 (-1.618 to -0.195) | 32.9 (0.00 to 54.9) |
| CRP (mg/L) | 31 | -0.374 (-0.536 to -0.212) | 66.0 (0.00 to 87.9) |
| IL-6 (pg/mL) | 20 | -0.211 (-0.431 to 0.010) | 54.4 (0.00 to 75.9) |
| TNF-α (pg/mL) | 14 | -0.050 (-0.189 to 0.088) | 43.2 (0.00 to 72.7) |
| HRQoL | 1 | -0.900 (-13.51 to 11.71) | - |
|  | **Correlation Coefficient (0.6)** | | |
| **Primary outcomes** |  |  |  |
| LDL-C (mmol/L) | 69 | -0.127 (-0.171 to -0.082) | 57.8 (36.2 to 70.0) |
| Body weight (kg) | 60 | -0.438 (-0.636 to -0.239) | 20.6 (0.00 to 43.9) |
| FPG (mmol/L) | 62 | -0.073 (-0.128 to -0.019) | 69.4 (51.6 to 78.9) |
| **Secondary outcomes** |  |  |  |
| Waist circumference (cm) | 38 | -0.511 (-0.891 to -0.131) | 37.5 (0.00 to 59.7) |
| Body fat percentage | 29 | -0.196 (-0.422 to 0.030) | 8.60 (0.00 to 42.5) |
| Insulin (pmol/L) | 42 | -0.555 (-2.579 to 1.468) | 18.2 (0.00 to 44.9) |
| HOMA-IR | 30 | -0.226 (-0.375 to -0.077) | 46.0 (0.00 to 66.9) |
| HbA_1c_ (%) | 24 | -0.047 (-0.094 to -0.001) | 55.3 (0.00 to 75.3) |
| Triglycerides (mmol/L) | 70 | -0.054 (-0.087 to -0.020) | 49.4 (11.2 to 67.3) |
| Total cholesterol (mmol/L) | 73 | -0.170 (-0.222 to -0.119) | 62.7 (43.4 to 73.5) |
| HDL-C (mmol/L) | 71 | 0.007 (-0.014 to 0.028) | 75.6 (58.5 to 84.0) |
| SBP (mm Hg) | 46 | -1.471 (-2.579 to -0.363) | 60.9 (33.5 to 74.3) |
| DBP (mm Hg) | 45 | -0.779 (-1.530 to -0.027) | 61.4 (32.7 to 75.0) |
| CRP (mg/L) | 31 | -0.371 (-0.540 to -0.202) | 79.6 (0.00 to 92.8) |
| IL-6 (pg/mL) | 20 | -0.259 (-0.501 to -0.016) | 73.1 (11.6 to 87.2) |
| TNF-α (pg/mL) | 14 | -0.045 (-0.180 to 0.090) | 59.0 (0.00 to 81.2) |
| HRQoL | 1 | -0.900 (-10.71 to 8.913) | - |
|  | **Correlation Coefficient (0.9)** | | |
| **Primary outcomes** |  |  |  |
| LDL-C (mmol/L) | 69 | -0.124 (-0.175 to -0.072) | 92.3 (83.2 to 95.6) |
| Body weight (kg) | 60 | -0.443 (-0.630 to -0.256) | 45.5 (1.90 to 65.4) |
| FPG (mmol/L) | 62 | -0.087 (-0.138 to -0.037) | 90.7 (80.1 to 94.6) |
| **Secondary outcomes** |  |  |  |
| Waist circumference (cm) | 38 | -0.575 (-0.947 to -0.202) | 66.9 (36.4 to 79.8) |
| Insulin (pmol/L) | 42 | -0.479 (-2.441 to 1.484) | 52.1 (9.00 to 70.4) |
| HOMA-IR | 30 | -0.264 (-0.420 to -0.109) | 89.1 (52.6 to 95.3) |
| HbA_1c_ (%) | 24 | -0.078 (-0.130 to -0.026) | 91.6 (44.7 to 96.8) |
| Body fat percentage | 29 | -0.247 (-0.479 to -0.015) | 47.1 (0.00 to 68.9) |
| Triglycerides (mmol/L) | 69 | -0.059 (-0.090 to -0.027) | 85.4 (68.5 to 91.6) |
| Total cholesterol (mmol/L) | 73 | -0.175 (-0.245 to -0.105) | 94.0 (88.9 to 96.3) |
| HDL-C (mmol/L) | 71 | 0.012 (-0.012 to 0.036) | 94.0 (89.4 to 96.1) |
| SBP (mm Hg) | 46 | -1.486 (-2.529 to -0.444) | 89.1 (73.5 to 94.1) |
| DBP (mm Hg) | 45 | -0.770 (-1.629 to 0.089) | 92.2 (81.6 to 95.7) |
| CRP (mg/L) | 31 | -0.340 (-0.519 to -0.160) | 94.9 (63.3 to 98.1) |
| IL-6 (pg/mL) | 20 | -0.258 (-0.475 to -0.042) | 89.8 (47.0 to 95.8) |
| TNF-α (pg/mL) | 14 | -0.026 (-0.140 to 0.087) | 84.0 (0.00 to 93.9) |
| HRQoL | 1 | -0.900 (-6.693 to 4.893) | - |

Abbreviations: MD: mean difference- SMD: standardized mean difference- CI: confidence interval- SBP: systolic blood pressure- DBP: diastolic blood pressure- LDL-C: low-density lipoprotein cholesterol- HDL-C: high-density lipoprotein cholesterol- FPG: fasting plasma glucose- HbA_1c_: glycated hemoglobin, HOMA-IR: homeostatic model assessment for insulin resistance- CRP: C-reactive protein- IL-6: interleukin 6- TNF-α: tumor necrosis factor-alpha- HRQoL: Health-related quality of life

*Obtained from random effects model.

†Inconsistency—percentage of variation across studies due to heterogeneity.

### Supplementary Table 11: Sensitivity analyses restricting to trials with a background diet with low whole grain intake.

|  | Overall | | | Low background whole-grain intake | | |
| --- | --- | --- | --- | --- | --- | --- |
| **Outcomes** | **Trials** | **Pooled MD (95% CI)*** | **I^2^ (%)†** | **Trials** | **Pooled MD (95% CI)*** | **I^2^ (%)†** |
| **Primary outcomes** |  |  |  |  |  |  |
| LDL-C (mmol/L) | 69 | -0.127 (-0.171 to -0.083) | 57.9 | 23 | -0.115 (-0.202 to -0.028) | 71.1 |
| Body weight (kg) | 60 | -0.512 (-0.722 to -0.302) | 40.4 | 22 | -0.373 (-0.650 to -0.096) | 35.2 |
| FPG (mmol/L) | 62 | -0.072 (-0.126 to -0.019) | 69.3 | 23 | -0.031 (-0.092 to 0.031) | 50.2 |
| **Secondary outcomes** |  |  |  |  |  |  |
| Waist circumference (cm) | 38 | -0.600 (-0.977 to -0.223) | 54.8 | 18 | -0.231 (-0.662 to 0.201) | 42.5 |
| Body fat percentage | 29 | -0.222 (-0.468 to 0.023) | 41.1 | 15 | -0.024 (-0.317 to 0.268) | 30.2 |
| Insulin (pmol/L) | 42 | -0.365 (-2.187 to 1.457) | 12.8 | 17 | -0.319 (-2.404 to 1.766) | 0.00 |
| HOMA-IR | 30 | -0.240 (-0.394 to -0.085) | 66.4 | 12 | -0.153 (-0.364 to 0.059) | 50.4 |
| HbA_1c_ (%) | 24 | -0.049 (-0.096 to -0.002) | 58.1 | 9 | -0.005 (-0.038 to 0.027) | 0.6 |
| Triglycerides (mmol/L) | 70 | -0.052 (-0.087 to -0.018) | 48.2 | 22 | -0.027 (-0.090 to 0.035) | 36.6 |
| Total cholesterol (mmol/L) | 73 | -0.168 (-0.218 to -0.118) | 71.5 | 22 | -0.121 (-0.197 to -0.046) | 61.8 |
| HDL-C (mmol/L) | 71 | 0.009 (-0.013 to 0.032) | 81.9 | 23 | 0.005 (-0.030 to 0.041) | 79.0 |
| SBP (mm Hg) | 46 | -1.500 (-2.623 to -0.377) | 62.5 | 17 | -1.327 (-3.361 to 0.707) | 78.7 |
| DBP (mm Hg) | 45 | -0.820 (-1.589 to -0.052) | 65.4 | 18 | -1.172 (-2.519 to 0.175) | 78.2 |
| CRP (mg/L) | 31 | -0.381 (-0.550 to -0.212) | 83.1 | 13 | -0.081 (-0.186 to 0.024) | 32.0 |
| IL-6 (pg/mL) | 20 | -0.224 (-0.444 to -0.004) | 67.3 | 8 | -0.077 (-0.273 to 0.119) | 0.00 |
| TNF-α (pg/mL) | 14 | -0.038 (-0.157 to 0.081) | 64.4 | 5 | -0.324 (-1.146 to 0.498) | 71.5 |
| HRQoL | 1 | -0.90 (-11.70 to 9.930) | - | 0 | - | - |

Abbreviations: MD: mean difference- SMD: standardized mean difference- CI: confidence interval- SBP: systolic blood pressure- DBP: diastolic blood pressure- LDL-C: low-density lipoprotein cholesterol- HDL-C: high-density lipoprotein cholesterol- FPG: fasting plasma glucose- HbA_1c_: glycated hemoglobin, HOMA-IR: homeostatic model assessment for insulin resistance- CRP: C-reactive protein- IL-6: interleukin 6- TNF-α: tumor necrosis factor-alpha- HRQoL: Health-related quality of life

*Obtained from random effects model.

†Inconsistency—percentage of variation across studies due to heterogeneity.

### Supplementary Table 12: Subgroup analyses on the effect of whole grains on adiposity measures in adults aged ≥18 years (pairwise meta-analyses).

|  |  |  | No of effect sizes | Pooled MD  (95% CI)* | I^2^ (%)† | P-heterogeneity‡ | P-group difference* |
| --- | --- | --- | --- | --- | --- | --- | --- |
| **Weight** | | |  |  |  |  |  |
|  | **Overall** | | 60 | -0.512 (-0.722 to -0.302) | 40.4 | 0.001 |  |
|  |  | **Predefined subgroup analyses** |  |  |  |  |  |
|  |  | Risk of bias |  |  |  |  | 0.801 |
|  |  | Low risk | 1 | -0.400 (-1.281 to 0.481) | - |  |  |
|  |  | High risk/some concerns | 59 | -0.517 (-0.732 to -0.301) | 41.4 | 0.001 |  |
|  |  | Health status** |  |  |  |  | 0.847 |
|  |  | Metabolically healthy | 25 | -0.493 (-0.793 to -0.194) | 47.4 | 0.005 |  |
|  |  | At risk | 36 | -0.535 (-0.836 to -0.235) | 34.4 | 0.024 |  |
|  |  | Intervention duration (week) |  |  |  |  | 0.188 |
|  |  | ≥8 | 34 | -0.654 (-0.991 to -0.316) | 56.3 | <0.001 |  |
|  |  | <8 | 26 | -0.391 (-0.588 to -0.195) | 0 | 0.575 |  |
|  |  | **Post hoc subgroup analyses** |  |  |  |  |  |
|  |  | Whole grain type†† |  |  |  |  | 0.511 |
|  |  | Mixed | 15 | -0.241 (-0.473 to -0.010) | 0 | 0.806 |  |
|  |  | Whole wheat | 7 | -0.687 (-1.469 to 0.095) | 57.8 | 0.027 |  |
|  |  | Brown rice | 15 | -0.873 (-1.431 to -0.316) | 70.8 | <0.001 |  |
|  |  | Barley | 2 | 0.043 (-1.597 to 1.683) | 0 | 0.942 |  |
|  |  | Oat | 13 | -0.457 (-0.923 to 0.009) | 17.1 | 0.266 |  |
|  |  | Rye | 5 | -0.723 (-1.288 to -0.158) | 24.8 | 0.256 |  |
|  |  | Quinoa | 3 | -0.648 (-1.915 to 0.620) | 0 | 0.905 |  |
|  |  | Buckwheat | 1 | -0.100 (-1.336 to 1.136) | - | - |  |
|  |  | Amaranth | - | - | - | - |  |
|  |  | Corn | 1 | 0.100 (-2.251 to 2.451) | - | - |  |
|  |  | Study design |  |  |  |  | 0.203 |
|  |  | Parallel | 44 | -0.583 (-0.860 to -0.305) | 46.8 | <0.001 |  |
|  |  | Crossover | 16 | -0.339 (-0.590 to -0.088) | 9.1 | 0.35 |  |
|  |  | Calorie restriction |  |  |  |  | 0.209 |
|  |  | Yes | 10 | -0.790 (-1.337 to -0.242) | 61.5 | 0.005 |  |
|  |  | No | 50 | -0.412 (-0.626 to -0.199) | 27.9 | 0.038 |  |
|  |  | Physical activity |  |  |  |  | 0.232 |
|  |  | Yes | 3 | 0.101 (-0.928 to 1.129) | 26.1 | 0.259 |  |
|  |  | No | 57 | -0.541 (-0.757 to -0.324) | 41.3 | 0.001 |  |
|  |  | Behavioral support |  |  |  |  | 0.738 |
|  |  | Yes | 13 | -0.444 (-0.952 to 0.064) | 56.6 | 0.006 |  |
|  |  | No | 47 | -0.539 (-0.770 to -0.309) | 34.8 | 0.011 |  |
|  |  | **Exploratory subgroup analyses** |  |  |  |  |  |
|  |  | Intervention duration (week) |  |  |  |  | 0.403 |
|  |  | ≥16 | 3 | -0.580 (-2.023 to 0.862) | 32.5 | 0.227 |  |
|  |  | 12 to <16 | 24 | -0.765 (-1.182 to -0.348) | 65.9 | <0.001 |  |
|  |  | 8 to <12 | 7 | -0.287 (-0.810 to 0.236) | 0 | 0.608 |  |
|  |  | <8 | 26 | -0.391 (-0.588 to -0.195) | 0 | 0.575 |  |
|  |  | Sex |  |  |  |  | 0.535 |
|  |  | Men and women | 51 | -0.464 (-0.701 to -0.226) | 42.0 | 0.001 |  |
|  |  | Men | 2 | -0.753 (-2.087 to 0.581) | 0 | 0.505 |  |
|  |  | Women | 7 | -0.739 (-1.184 to -0.293) | 28.6 | 0.210 |  |
|  |  | Baseline risk‡‡ |  |  |  |  | 0.302 |
|  |  | Low risk | 10 | -0.343 (-0.655 to -0.031) | 0 | 0.829 |  |
|  |  | High risk | 51 | -0.551 (-0.796 to -0.307) | 46.5 | <0.001 |  |
|  |  | Obesity status |  |  |  |  | 0.025 |
|  |  | Overweight/obese | 30 | -0.697 (-1.026 to -0.368) | 55.6 | <0.001 |  |
|  |  | Normal weight | - | - | - | - |  |
|  |  | Mixed | 30 | -0.251 (-0.457 to -0.045) | 0 | 0.512 |  |
|  |  | Comparator type⁋ |  |  |  |  | 0.958 |
|  |  | Minimal intervention | 9 | -0.442 (-1.093 to 0.209) | 57.4 | 0.016 |  |
|  |  | Low fiber/ refined grains | 50 | -0.535 (-0.759 to -0.311) | 37.1 | 0.005 |  |
|  |  | Healthy diet | 1 | -0.622 (-2.118 to 0.875) | - | - |  |
|  |  | Use of run-in period |  |  |  |  | 0.283 |
|  |  | Yes | 15 | -0.375 (-0.676 to -0.073) | 40.6 | 0.052 |  |
|  |  | No | 45 | -0.602 (-0.888 to -0.317) | 41.1 | 0.003 |  |
| **Waist circumference** | | |  |  |  |  |  |
|  | **Overall** | | 38 | -0.600 (-0.977 to -0.223) | 54.8 | <0.001 |  |
|  |  | **Predefined subgroup analyses** |  |  |  |  |  |
|  |  | Risk of bias |  |  |  |  | 0.831 |
|  |  | Low risk | 1 | -0.500 (-1.363 to 0.363) | - | - |  |
|  |  | High risk/some concerns | 37 | -0.604 (-1.000 to -0.207) | 56.1 | <0.001 |  |
|  |  | Health status** |  |  |  |  | 0.407 |
|  |  | Metabolically healthy | 11 | -0.358 (-0.976 to 0.261) | 37.1 | 0.103 |  |
|  |  | At risk | 27 | -0.685 (-1.153 to -0.218) | 59.8 | <0.001 |  |
|  |  | Intervention duration (week) |  |  |  |  | 0.363 |
|  |  | ≥8 | 25 | -0.712 (-1.198 to -0.226) | 62.0 | <0.001 |  |
|  |  | <8 | 13 | -0.362 (-0.939 to 0.215) | 32.7 | 0.121 |  |
|  |  | **Post hoc subgroup analyses** |  |  |  |  |  |
|  |  | Whole grain type†† |  |  |  |  | 0.102 |
|  |  | Mixed | 9 | 0.144 (-0.453 to 0.741) | 23.0 | 0.239 |  |
|  |  | Whole wheat | 6 | -0.565 (-1.193 to 0.063) | 0 | 0.661 |  |
|  |  | Brown rice | 13 | -0.919 (-1.716 to -0.123) | 75.1 | <0.001 |  |
|  |  | Barley | 1 | -0.054 (-2.942 to 2.835) | - | - |  |
|  |  | Oat | 6 | -0.937 (-1.677 to -0.198) | 16.4 | 0.305 |  |
|  |  | Rye | 2 | -1.699 (-2.944 to -0.454) | 0 | 0.365 |  |
|  |  | Quinoa | 3 | -0.774 (-1.940 to 0.391) | 0 | 0.644 |  |
|  |  | Buckwheat | - | - | - | - |  |
|  |  | Amaranth | - | - | - | - |  |
|  |  | Corn | - | - | - | - |  |
|  |  | Study design |  |  |  |  | 0.580 |
|  |  | Parallel | 30 | -0.643 (-1.079 to -0.208) | 58.5 | <0.001 |  |
|  |  | Crossover | 8 | -0.397 (-1.154 to 0.361) | 38.8 | 0.121 |  |
|  |  | Calorie restriction |  |  |  |  | 0.928 |
|  |  | Yes | 9 | -0.624 (-1.521 to 0.274) | 60.6 | 0.009 |  |
|  |  | No | 29 | -0.578 (-0.996 to -0.160) | 53.7 | <0.001 |  |
|  |  | Physical activity |  |  |  |  | 0.619 |
|  |  | Yes | 2 | 0.226 (-3.063 to 3.514) | 78.3 | 0.032 |  |
|  |  | No | 36 | -0.615 (-1.001 to -0.228) | 54.6 | <0.001 |  |
|  |  | Behavioral support |  |  |  |  | 0.755 |
|  |  | Yes | 9 | -0.500 (-1.233 to 0.233) | 53.0 | 0.030 |  |
|  |  | No | 29 | -0.637 (-1.085 to -0.188) | 56.9 | <0.001 |  |
|  |  | **Exploratory subgroup analyses** |  |  |  |  |  |
|  |  | Intervention duration (week) |  |  |  |  | 0.700 |
|  |  | ≥16 | 2 | -0.205 ( -1.378 to 0.969) | 61.0 | 0.109 |  |
|  |  | 12 to <16 | 19 | -0.790 (-1.391 to -0.189) | 66.2 | <0.001 |  |
|  |  | 8 to <12 | 4 | -0.675 (-1.592 to 0.241) | 0 | 0.568 |  |
|  |  | <8 | 13 | -0.362 (-0.939 to 0.215) | 32.7 | 0.121 |  |
|  |  | Sex |  |  |  |  | 0.722 |
|  |  | Men and women | 33 | -0.620 (-1.035 to -0.205) | 57.8 | <0.001 |  |
|  |  | Men | - | - | - | - |  |
|  |  | Women | 5 | -0.438 (-1.352 to 0.476) | 32.8 | 0.203 |  |
|  |  | Baseline risk‡‡ |  |  |  |  | 0.037 |
|  |  | Low risk | 2 | 0.580 (-0.519 to 1.680) | 0 | 0.345 |  |
|  |  | High risk | 36 | -0.659 (-1.045 to -0.274) | 54.5 | <0.001 |  |
|  |  | Obesity status |  |  |  |  | 0.760 |
|  |  | Overweight/obese | 24 | -0.562 (-0.999 to -0.125) | 38.8 | 0.028 |  |
|  |  | Normal weight | - | - | - | - |  |
|  |  | Mixed | 14 | -0.690 (-1.382 to 0.002) | 70.6 | <0.001 |  |
|  |  | Comparator type⁋ |  |  |  |  | 0.350 |
|  |  | Minimal intervention | 3 | -0.973 (-2.034 to 0.089) | 0 | 0.978 |  |
|  |  | Low fiber/ refined grains | 34 | -0.602 (-1.014 to -0.190) | 58.7 | <0.001 |  |
|  |  | Healthy diet | 1 | 0.246 (-1.050 to 1.543) | - | - |  |
|  |  | Use of run-in period |  |  |  |  | 0.308 |
|  |  | Yes | 11 | -0.332 (-0.856 to 0.193) | 38.6 | 0.092 |  |
|  |  | No | 27 | -0.709 (-1.209 to -0.208) | 58.9 | <0.001 |  |
| **Body fat percentage** | | |  |  |  |  |  |
|  | **Overall** | | 29 | -0.222 (-0.468 to 0.023) | 41.1 | 0.012 |  |
|  |  | **Predefined subgroup analyses** |  |  |  |  |  |
|  |  | Risk of bias |  |  |  |  | - |
|  |  | Low risk | - | - | - | - |  |
|  |  | High risk/some concerns | 29 | -0.222 (-0.468 to 0.023) | 41.1 | 0.012 |  |
|  |  | Health status** |  |  |  |  | 0.583 |
|  |  | Metabolically healthy | 17 | -0.311 (-0.695 to 0.074) | 57 | 0.002 |  |
|  |  | At risk | 12 | -0.178 (-0.455 to 0.099) | 0 | 0.502 |  |
|  |  | Intervention duration (week) |  |  |  |  | 0.480 |
|  |  | ≥8 | 18 | -0.304 (-0.678 to 0.070) | 54.6 | 0.003 |  |
|  |  | <8 | 11 | -0.137 (-0.410 to 0.135) | 0 | 0.458 |  |
|  |  | **Post hoc subgroup analyses** |  |  |  |  |  |
|  |  | Whole grain type†† |  |  |  |  | 0.696 |
|  |  | Mixed | 10 | 0.026 (-0.287 to 0.339) | 28.5 | 0.182 |  |
|  |  | Whole wheat | 3 | -0.316 (-1.280 to 0.648) | 30.6 | 0.236 |  |
|  |  | Brown rice | 6 | -0.821 (-2.168 to 0.526) | 74.4 | 0.002 |  |
|  |  | Barley | 1 | -0.220 (-1.962 to 1.522) | - | - |  |
|  |  | Oat | 5 | -0.311 (-0.725 to 0.103) | 0 | 0.445 |  |
|  |  | Rye | 3 | -0.292 (-0.936 to 0.353) | 40.1 | 0.188 |  |
|  |  | Quinoa | 2 | -0.500 (-1.225 to 0.225) | 0 | 1.00 |  |
|  |  | Buckwheat | - | - | - | - |  |
|  |  | Amaranth | - | - | - | - |  |
|  |  | Corn | - | - | - | - |  |
|  |  | Study design |  |  |  |  | 0.750 |
|  |  | Parallel | 22 | -0.255 (-0.593 to 0.082) | 50.7 | 0.004 |  |
|  |  | Crossover | 7 | -0.183 (-0.476 to 0.111) | 0 | 0.556 |  |
|  |  | Calorie restriction |  |  |  |  | 0.023 |
|  |  | Yes | 4 | -0.675 (-1.078 to -0.272) | 0 | 0.498 |  |
|  |  | No | 25 | -0.116 (-0.383 to 0.151) | 37.5 | 0.031 |  |
|  |  | Physical activity |  |  |  |  | 0.952 |
|  |  | Yes | 1 | -0.200 (-1.008 to 0.608) | - | - |  |
|  |  | No | 28 | -0.226 (-0.484 to 0.032) | 43.2 | 0.009 |  |
|  |  | Behavioral support |  |  |  |  | 0.970 |
|  |  | Yes | 6 | -0.236 (-0.654 to 0.183) | 46.0 | 0.099 |  |
|  |  | No | 23 | -0.226 (-0.535 to 0.084) | 42.2 | 0.018 |  |
|  |  | **Exploratory subgroup analyses** |  |  |  |  |  |
|  |  | Intervention duration (week) |  |  |  |  | 0.824 |
|  |  | ≥16 | 2 | -2.101 (-7.151 to 2.948) | 93.8 | <0.001 |  |
|  |  | 12 to <16 | 11 | -0.283 (-0.688 to 0.122) | 45.4 | 0.050 |  |
|  |  | 8 to <12 | 5 | -0.163 (-0.608 to 0.283) | 0 | 0.587 |  |
|  |  | <8 | 11 | -0.137 (-0.410 to 0.135) | 0 | 0.458 |  |
|  |  | Sex |  |  |  |  | 0.473 |
|  |  | Men and women | 24 | -0.147 (-0.402 to 0.109) | 33.5 | 0.057 |  |
|  |  | Men | 1 | -0.500 (-1.288 to 0.288) | - | - |  |
|  |  | Women | 4 | -0.653 (-1.660 to 0.354) | 73.3 | 0.010 |  |
|  |  | Baseline risk‡‡ |  |  |  |  | 0.196 |
|  |  | Low risk | 6 | -0.834 (-1.812 to 0.143) | 73.4 | 0.002 |  |
|  |  | High risk | 23 | -0.172 (-0.404 to 0.059) | 23.0 | 0.157 |  |
|  |  | Obesity status |  |  |  |  | 0.980 |
|  |  | Overweight/obese | 16 | -0.242 (-0.523 to 0.038) | 32.4 | 0.103 |  |
|  |  | Normal weight | - | - | - | - |  |
|  |  | Mixed | 13 | -0.235 (-0.710 to 0.240) | 51.9 | 0.015 |  |
|  |  | Comparator type⁋ |  |  |  |  | 0.532 |
|  |  | Minimal intervention | 5 | -0.559 (-1.370 to 0.251) | 41.8 | 0.143 |  |
|  |  | Low fiber/ refined grains | 23 | -0.198 (-0.472 to 0.077) | 44.3 | 0.012 |  |
|  |  | Healthy diet | 1 | 0.070 (-0.674 to 0.814) | - | - |  |
|  |  | Use of run-in period |  |  |  |  | 0.189 |
|  |  | Yes | 9 | -0.017 (-0.407 to 0.373) | 45.6 | 0.065 |  |
|  |  | No | 20 | -0.355 (-0.676 to -0.035) | 39.6 | 0.036 |  |

Abbreviations: MD: mean difference- CI: confidence interval

*Obtained from random effects model.

†Inconsistency—percentage of variation across studies due to heterogeneity.

‡Obtained from Q test.

**Studies that recruited adults with one or more of the following criteria: fasting plasma glucose ≥5.6 mmol/L, serum triglycerides ≥1.7 mmol/L, serum total cholesterol level ≥5.2 mmol/L, serum LDL cholesterol level ≥3.4 mmol/L, fasting serum HDL cholesterol <1.29 mmol/L (women) or <1.03 mmol/L (men), and/or blood pressure ≥130/≥85 mm Hg were included in the at risk subgroup, and studies that recruited participants without considering these criteria were included in the healthy subgroup.

††Studies that examined two types of whole grains were analysed separately in the subgroup analysis.

‡‡High baseline risk was defined as individuals with metabolic disease, medication users, or those with overweight/obesity.

⁋The minimum intervention was defined as the usual or conventional diets, routine care, no intervention or placebo, while low fiber/refined grains were defined as refined wheat, refined corn, white rice, or mixed refined grains.

### Supplementary Table 13: Subgroup analyses on the effect of whole grains on glycemic control in adults aged ≥18 years (pairwise meta-analyses).

|  |  |  | No of effect sizes | Pooled MD  (95% CI)* | I^2^ (%)† | P-heterogeneity‡ | P-group difference* |
| --- | --- | --- | --- | --- | --- | --- | --- |
| **FPG** | | |  |  |  |  |  |
|  | **Overall** | | 62 | -0.072 (-0.126 to -0.019) | 69.3 | <0.001 |  |
|  |  | **Predefined subgroup analyses** |  |  |  |  |  |
|  |  | Risk of bias |  |  |  |  | 0.890 |
|  |  | Low risk | 2 | -0.051 (-0.364 to 0.262) | 71.8 | 0.06 |  |
|  |  | High risk/some concerns | 60 | -0.074 (-0.128 to -0.019) | 69.7 | <0.001 |  |
|  |  | Health status**¥ |  |  |  |  | 0.181 |
|  |  | Metabolically healthy | 27 | -0.041 (-0.091 to 0.008) | 31.7 | 0.060 |  |
|  |  | At risk | 36 | -0.112 (-0.202 to -0.021) | 78.9 | <0.001 |  |
|  |  | Intervention duration (week) |  |  |  |  | 0.229 |
|  |  | ≥8 | 35 | -0.041 (-0.103 to 0.020) | 59.8 | <0.001 |  |
|  |  | <8 | 27 | -0.111 (-0.207 to -0.015) | 77 | <0.001 |  |
|  |  | **Post hoc subgroup analyses** |  |  |  |  |  |
|  |  | Whole grain type†† |  |  |  |  | 0.110 |
|  |  | Mixed | 20 | -0.044 (-0.085 to -0.002) | 1.6 | 0.437 |  |
|  |  | Whole wheat | 5 | -0.016 (-0.311 to 0.278) | 82.4 | <0.001 |  |
|  |  | Brown rice | 19 | -0.245 (-0.440 to -0.050) | 86.1 | <0.001 |  |
|  |  | Barley | 3 | -0.053 (-0.256 to 0.150) | 35.1 | 0.202 |  |
|  |  | Oat | 9 | -0.061 (-0.139 to 0.017) | 0 | 0.659 |  |
|  |  | Rye | 3 | 0.017 (-0.077 to 0.111) | 24.3 | 0.267 |  |
|  |  | Quinoa | 4 | -0.171 (-0.296 to -0.045) | 0 | 0.896 |  |
|  |  | Buckwheat | 1 | 0.350 (-0.184 to 0.884) | - | - |  |
|  |  | Amaranth | - | - | - | - |  |
|  |  | Corn | - | - | - | - |  |
|  |  | Study design |  |  |  |  | 0.175 |
|  |  | Parallel | 45 | -0.044 (-0.102 to 0.014) | 59.8 | <0.001 |  |
|  |  | Crossover | 17 | -0.135 (-0.252 to -0.018) | 81.6 | <0.001 |  |
|  |  | Calorie restriction |  |  |  |  | 0.510 |
|  |  | Yes | 8 | -0.043 (-0.122 to 0.036) | 26.4 | 0.218 |  |
|  |  | No | 54 | -0.077 (-0.139 to -0.015) | 71.9 | <0.001 |  |
|  |  | Physical activity |  |  |  |  | 0.363 |
|  |  | Yes | 1 | 0.011 (-0.165 to 0.187) | - | - |  |
|  |  | No | 61 | -0.075 (-0.129 to -0.020) | 69.7 | <0.001 |  |
|  |  | Behavioral support |  |  |  |  | 0.550 |
|  |  | Yes | 10 | -0.044 (-0.137 to 0.049) | 46.5 | 0.052 |  |
|  |  | No | 52 | -0.078 (-0.140 to -0.015) | 71.9 | <0.001 |  |
|  |  | **Exploratory subgroup analyses** |  |  |  |  |  |
|  |  | Use of glucose-lowering medications |  |  |  |  | 0.180 |
|  |  | Yes | 9 | -0.504 (-1.006 to -0.001) | 87.5 | <0.001 |  |
|  |  | No | 37 | -0.038 (-0.082 to 0.005) | 43.4 | 0.003 |  |
|  |  | Unclear | 16 | -0.068 (-0.183 to 0.048) | 66.4 | <0.001 |  |
|  |  | Intervention duration (week) |  |  |  |  | 0.586 |
|  |  | ≥16 | 5 | -0.092 (-0.219 to 0.036) | 15.9 | 0.313 |  |
|  |  | 12 to <16 | 22 | -0.034 (-0.101 to 0.033) | 52.8 | 0.002 |  |
|  |  | 8 to <12 | 8 | -0.084 (-0.293 to 0.125) | 79.1 | <0.001 |  |
|  |  | <8 | 27 | -0.111 (-0.207 to -0.015) | 77.0 | <0.001 |  |
|  |  | Sex |  |  |  |  | 0.160 |
|  |  | Men and women | 53 | -0.077 (-0.138 to -0.017) | 71.6 | <0.001 |  |
|  |  | Men | 2 | -0.294 (-0.787 to 0.198) | 25.6 | 0.246 |  |
|  |  | Women | 7 | 0.014 (-0.076 to 0.104) | 24.8 | 0.239 |  |
|  |  | Baseline risk‡‡ |  |  |  |  | 0.306 |
|  |  | Low risk | 14 | -0.037 (-0.099 to 0.026) | 8.4 | 0.360 |  |
|  |  | High risk | 49 | -0.084 (-0.151 to -0.018) | 74.7 | <0.001 |  |
|  |  | Diabetes status |  |  |  |  | 0.03 |
|  |  | Diabetic | 7 | -0.708 (-1.296 to -0.121) | 83.4 | <0.001 |  |
|  |  | Non-diabetic | 50 | -0.029 (-0.066 to 0.009) | 40.3 | 0.002 |  |
|  |  | Mixed | 5 | -0.404 (-0.932 to 0.125) | 82.4 | <0.001 |  |
|  |  | Comparator type⁋ |  |  |  |  | 0.385 |
|  |  | Minimal intervention | 11 | -0.160 (-0.295 to -0.025) | 60.7 | 0.005 |  |
|  |  | Low fiber/ refined grains | 50 | -0.056 (-0.116 to 0.003) | 71.3 | <0.001 |  |
|  |  | Healthy diet | 1 | -0.093 (-0.774 to 0.587) | - | - |  |
|  |  | Use of run-in period |  |  |  |  | 0.020 |
|  |  | Yes | 14 | -0.004 (-0.049 to 0.041) | 0 | 0.481 |  |
|  |  | No | 48 | -0.105 (-0.177 to -0.033) | 74.0 | <0.001 |  |
| **HbA_1c_** | | |  |  |  |  |  |
|  | **Overall** | | 24 | -0.049 (-0.096 to -0.002) | 58.1 | <0.001 |  |
|  |  | **Predefined subgroup analyses** |  |  |  |  |  |
|  |  | Risk of bias |  |  |  |  | - |
|  |  | Low risk | - | - | - | - |  |
|  |  | High risk/some concerns | 24 | -0.049 (-0.096 to -0.002) | 58.1 | <0.001 |  |
|  |  | Health status** |  |  |  |  | 0.102 |
|  |  | Metabolically healthy | 8 | -0.007 (-0.045 to 0.030) | 0 | 0.465 |  |
|  |  | At risk | 16 | -0.076 (-0.150 to -0.003) | 67.5 | <0.001 |  |
|  |  | Intervention duration (week) |  |  |  |  | 0.216 |
|  |  | ≥8 | 18 | -0.060 (-0.116 to -0.005) | 62.7 | <0.001 |  |
|  |  | <8 | 6 | 0.001 (-0.078 to 0.079) | 24.0 | 0.254 |  |
|  |  | **Post hoc subgroup analyses** |  |  |  |  |  |
|  |  | Whole grain type†† |  |  |  |  | 0.115 |
|  |  | Mixed | 4 | -0.043 (-0.120 to 0.034) | 59.8 | 0.059 |  |
|  |  | Whole wheat | 2 | 0.266 (-0.352 to 0.885) | 82.5 | 0.017 |  |
|  |  | Brown rice | 11 | -0.049 (-0.121 to 0.024) | 32.6 | 0.138 |  |
|  |  | Barley | 3 | 0.009 (-0.115 to 0.134) | 58.8 | 0.064 |  |
|  |  | Oat | 2 | -0.073 (-0.342 to 0.197) | 80.2 | 0.006 |  |
|  |  | Rye | 1 | -0.020 (-0.140 to 0.100) | - | - |  |
|  |  | Quinoa | 1 | -0.250 (-0.378 to -0.122) | - | - |  |
|  |  | Buckwheat | 1 | -0.120 (-0.381 to 0.141) | - | - |  |
|  |  | Amaranth | - | - | - | - |  |
|  |  | Corn | - | - | - | - |  |
|  |  | Study design |  |  |  |  | 0.825 |
|  |  | Parallel | 16 | -0.046 (-0.107 to 0.015) | 61.4 | 0.001 |  |
|  |  | Crossover | 8 | -0.058 (-0.137 to 0.022) | 53.4 | 0.036 |  |
|  |  | Calorie restriction |  |  |  |  | 0.239 |
|  |  | Yes | 1 | 0.000 (-0.076 to 0.076) | - | - |  |
|  |  | No | 23 | -0.055 (-0.106 to -0.004) | 59.5 | <0.001 |  |
|  |  | Physical activity |  |  |  |  | - |
|  |  | Yes | - | - | - | - |  |
|  |  | No | 24 | -0.049 (-0.096 to -0.002) | 58.1 | <0.001 |  |
|  |  | Behavioral support |  |  |  |  | 0.061 |
|  |  | Yes | 7 | -0.156 (-0.286 to -0.025) | 80.3 | <0.001 |  |
|  |  | No | 17 | -0.024 (-0.068 to 0.020) | 34.5 | 0.080 |  |
|  |  | **Exploratory subgroup analyses** |  |  |  |  |  |
|  |  | Use of glucose-lowering medications |  |  |  |  | 0.287 |
|  |  | Yes | 8 | -0.184 (-0.395 to 0.027) | 65.8 | 0.005 |  |
|  |  | No | 8 | -0.036 (-0.095 to 0.022) | 71.3 | 0.001 |  |
|  |  | Unclear | 8 | -0.013 (-0.063 to 0.036) | 0 | 0.682 |  |
|  |  | Intervention duration (week) |  |  |  |  | 0.462 |
|  |  | ≥16 | 4 | -0.155 (-0.354 to 0.044) | 69.0 | 0.021 |  |
|  |  | 12 to <16 | 8 | -0.016 (-0.066 to 0.034) | 42.0 | 0.099 |  |
|  |  | 8 to <12 | 6 | -0.067 (-0.192 to 0.059) | 50.9 | 0.070 |  |
|  |  | <8 | 6 | 0.001 (-0.078 to 0.079) | 24.0 | 0.254 |  |
|  |  | Sex |  |  |  |  | 0.568 |
|  |  | Men and women | 20 | -0.058 (-0.115 to -0.002) | 59.7 | <0.001 |  |
|  |  | Men | 1 | -0.070 (-0.679 to 0.539) | - | - |  |
|  |  | Women | 3 | -0.005 (-0.086 to 0.076) | 52.4 | 0.122 |  |
|  |  | Baseline risk‡‡ |  |  |  |  | 0.380 |
|  |  | Low risk | 5 | -0.014 (-0.093 to 0.065) | 9.2 | 0.354 |  |
|  |  | High risk | 19 | -0.057 (-0.112 to -0.002) | 64.2 | <0.001 |  |
|  |  | Diabetes status |  |  |  |  | 0.393 |
|  |  | Diabetic | 7 | -0.207 (-0.472 to 0.058) | 68.6 | 0.004 |  |
|  |  | Non-diabetic | 15 | -0.028 (-0.069 to 0.013) | 52.2 | 0.01 |  |
|  |  | Mixed | 2 | -0.070 (-0.247 to 0.107) | 0 | 1 |  |
|  |  | Comparator type⁋ |  |  |  |  | 0.061 |
|  |  | Minimal intervention | 5 | -0.082 (-0.302 to 0.138) | 79.4 | 0.001 |  |
|  |  | Low fiber/ refined grains | 18 | -0.026 (-0.065 to 0.013) | 37.7 | 0.054 |  |
|  |  | Healthy diet | 1 | -0.559 (-1.007 to -0.111) | - | - |  |
|  |  | Use of run-in period |  |  |  |  | 0.397 |
|  |  | Yes | 5 | -0.019 (-0.087 to 0.049) | 46.5 | 0.113 |  |
|  |  | No | 19 | -0.058 (-0.119 to 0.003) | 60.0 | <0.001 |  |
| **Insulin** | | |  |  |  |  |  |
|  | **Overall** | | 42 | -0.365 (-2.187 to 1.457) | 12.8 | 0.240 |  |
|  |  | **Predefined subgroup analyses** |  |  |  |  |  |
|  |  | Risk of bias |  |  |  |  | 0.053 |
|  |  | Low risk | 2 | -12.40 (-24.74 to -0.059) | 0 | 0.941 |  |
|  |  | High risk/some concerns | 40 | -0.078 (-1.856 to 1.700) | 9.6 | 0.298 |  |
|  |  | Health status**¥ |  |  |  |  | 0.209 |
|  |  | Metabolically healthy | 18 | -2.133 (-5.621 to 1.354) | 29.4 | 0.118 |  |
|  |  | At risk | 25 | 0.599 (-1.858 to 3.057) | 18.5 | 0.204 |  |
|  |  | Intervention duration (week) |  |  |  |  | 0.158 |
|  |  | ≥8 | 27 | 0.317 (-1.553 to 2.186) | 0 | 0.929 |  |
|  |  | <8 | 15 | -3.293 (-7.943 to 1.356) | 53.1 | 0.008 |  |
|  |  | **Post hoc subgroup analyses** |  |  |  |  |  |
|  |  | Whole grain type†† |  |  |  |  | 0.516 |
|  |  | Mixed | 17 | -1.009 (-4.632 to 2.614) | 19.8 | 0.223 |  |
|  |  | Whole wheat | 4 | 0.248 (-2.585 to 3.080) | 0 | 0.712 |  |
|  |  | Brown rice | 11 | -0.140 (-4.020 to 3.739) | 0 | 0.475 |  |
|  |  | Barley | 1 | 1.722 (-8.790 to 12.23) | - | - |  |
|  |  | Oat | 5 | -1.747 (-9.318 to 5.824) | 37.4 | 0.157 |  |
|  |  | Rye | 3 | 1.372 (-3.910 to 6.654) | 0 | 0.441 |  |
|  |  | Quinoa | 2 | -1.401 (-20.95 to 18.15) | 36.7 | 0.209 |  |
|  |  | Buckwheat | 1 | -15.84 (-29.16 to -2.524) | - | - |  |
|  |  | Amaranth | - | - | - | - |  |
|  |  | Corn | - | - | - | - |  |
|  |  | Study design |  |  |  |  | 0.327 |
|  |  | Parallel | 28 | 0.022 (-1.851 to 1.896) | 0 | 0.748 |  |
|  |  | Crossover | 14 | -2.398 (-6.863 to 2.066) | 48.2 | 0.022 |  |
|  |  | Calorie restriction |  |  |  |  | 0.893 |
|  |  | Yes | 6 | -0.258 (-3.448 to 2.933) | 0 | 0.595 |  |
|  |  | No | 36 | -0.522 (-2.712 to 1.667) | 19.2 | 0.158 |  |
|  |  | Physical activity |  |  |  |  | 0.364 |
|  |  | Yes | 1 | 7.800 (-9.941 to 25.54) | - | - |  |
|  |  | No | 41 | -0.456 (-2.297 to 1.384) | 13.4 | 0.231 |  |
|  |  | Behavioral support |  |  |  |  | 0.753 |
|  |  | Yes | 7 | 0.188 (-3.913 to 4.290) | 28.7 | 0.209 |  |
|  |  | No | 35 | -0.553 (-2.671 to 1.565) | 11.8 | 0.272 |  |
|  |  | **Exploratory subgroup analyses** |  |  |  |  |  |
|  |  | Use of glucose-lowering medications |  |  |  |  | 0.950 |
|  |  | Yes | 5 | -0.422 (-7.930 to 7.087) | 51.9 | 0.081 |  |
|  |  | No | 28 | -0.206 (-2.330 to 1.917) | 12.0 | 0.285 |  |
|  |  | Unclear | 9 | -0.952 (-4.992 to 3.087) | 0.00 | 0.450 |  |
|  |  | Intervention duration (week) |  |  |  |  | 0.538 |
|  |  | ≥16 | 2 | 1.531 (-4.721 to 7.783) | 0 | 0.940 |  |
|  |  | 12 to <16 | 18 | 0.291 (-2.165 to 2.747) | 0 | 0.828 |  |
|  |  | 8 to <12 | 7 | 0.034 (-3.215 to 3.283) | 0 | 0.599 |  |
|  |  | <8 | 15 | -3.293 (-7.943 to 1.356) | 53.1 | 0.008 |  |
|  |  | Sex |  |  |  |  | 0.606 |
|  |  | Men and women | 36 | -0.841 (-2.978 to 1.296) | 15.5 | 0.211 |  |
|  |  | Men | 2 | -8.670 (-38.42 to 21.08) | 76.9 | 0.037 |  |
|  |  | Women | 4 | 0.877 (-2.525 to 4.278) | 0 | 0.878 |  |
|  |  | Baseline risk‡‡ |  |  |  |  | 0.082 |
|  |  | Low risk | 8 | -6.796 (-14.56 to 0.972) | 38.9 | 0.120 |  |
|  |  | High risk | 35 | 0.311 (-1.613 to 2.236) | 14.5 | 0.228 |  |
|  |  | Diabetes status |  |  |  |  | 0.777 |
|  |  | Diabetic | 4 | -1.197 (-11.99 to 9.599) | 62.9 | 0.044 |  |
|  |  | Non-diabetic | 36 | -0.088 (-1.759 to 1.583) | 0.1 | 0.466 |  |
|  |  | Mixed | 2 | -9.550 (-36.61 to 17.51) | 73.5 | 0.052 |  |
|  |  | Comparator type⁋ |  |  |  |  | 0.871 |
|  |  | Minimal intervention | 5 | -1.125 (-9.979 to 7.730) | 28.2 | 0.234 |  |
|  |  | Low fiber/ refined grains | 37 | -0.375 (-2.246 to 1.497) | 13.1 | 0.246 |  |
|  |  | Healthy diet | 0 | - | - | - |  |
|  |  | Use of run-in period |  |  |  |  | 0.974 |
|  |  | Yes | 12 | -0.438 (-3.025 to 2.149) | 0 | 0.515 |  |
|  |  | No | 30 | -0.496 (-2.971 to 1.978) | 21.1 | 0.153 |  |
| **HOMA-IR** | | |  |  |  |  |  |
|  | **Overall** | | 30 | -0.240 (-0.394 to -0.085) | 66.4 | <0.001 |  |
|  |  | **Predefined subgroup analyses** |  |  |  |  |  |
|  |  | Risk of bias |  |  |  |  | 0.019 |
|  |  | Low risk | 2 | -0.600 (-0.888 to -0.312) | 0 | 1.00 |  |
|  |  | High risk/some concerns | 28 | -0.205 (-0.364 to -0.046) | 64.9 | <0.001 |  |
|  |  | Health status**¥ |  |  |  |  | 0.821 |
|  |  | Metabolically healthy | 9 | -0.253 (-0.542 to 0.036) | 52.0 | 0.034 |  |
|  |  | At risk | 22 | -0.212 (-0.418 to -0.006) | 76.7 | <0.001 |  |
|  |  | Intervention duration (week) |  |  |  |  | 0.279 |
|  |  | ≥8 | 20 | -0.182 (-0.361 to -0.003) | 64.0 | <0.001 |  |
|  |  | <8 | 10 | -0.389 (-0.719 to -0.059) | 73.1 | <0.001 |  |
|  |  | **Post hoc subgroup analyses** |  |  |  |  |  |
|  |  | Whole grain type†† |  |  |  |  | 0.005 |
|  |  | Mixed | 12 | -0.173 (-0.379 to 0.033) | 40.4 | 0.072 |  |
|  |  | Whole wheat | 2 | 0.053 (-0.239 to 0.346) | 0 | 0.466 |  |
|  |  | Brown rice | 9 | -0.332 (-0.722 to 0.058) | 71.1 | <0.001 |  |
|  |  | Barley | 1 | 0.090 (-0.270 to 0.451) | - | - |  |
|  |  | Oat | 4 | -0.309 (-0.737 to 0.119) | 59.7 | 0.042 |  |
|  |  | Rye | 2 | 0.132 (-0.294 to 0.557) | 63.2 | 0.099 |  |
|  |  | Quinoa | 1 | -0.500 (-0.756 to -0.244) | - | - |  |
|  |  | Buckwheat | 1 | -1.243 (-2.033 to -0.452) | - | - |  |
|  |  | Amaranth | - | - | - | - |  |
|  |  | Corn | - | - | - | - |  |
|  |  | Study design |  |  |  |  | 0.315 |
|  |  | Parallel | 21 | -0.187 (-0.363 to -0.011) | 58.9 | <0.001 |  |
|  |  | Crossover | 9 | -0.378 (-0.705 to -0.050) | 78.5 | <0.001 |  |
|  |  | Calorie restriction |  |  |  |  | 0.040 |
|  |  | Yes | 4 | -0.010 (-0.190 to 0.170) | 0 | 0.616 |  |
|  |  | No | 26 | -0.276 (-0.455 to -0.098) | 69.1 | <0.001 |  |
|  |  | Physical activity |  |  |  |  | - |
|  |  | Yes | - | - | - | - |  |
|  |  | No | 30 | -0.240 (-0.394 to -0.085) | 66.4 | <0.001 |  |
|  |  | Behavioral support |  |  |  |  | 0.426 |
|  |  | Yes | 6 | -0.371 (-0.728 to -0.013) | 74.6 | 0.001 |  |
|  |  | No | 24 | -0.209 (-0.385 to -0.032) | 64.8 | <0.001 |  |
|  |  | **Exploratory subgroup analyses** |  |  |  |  |  |
|  |  | Use of glucose-lowering medications |  |  |  |  | 0.568 |
|  |  | Yes | 5 | -0.473 (-1.020 to 0.074) | 61.1 | 0.036 |  |
|  |  | No | 19 | -0.188 (-0.369 to -0.006) | 67.3 | <0.001 |  |
|  |  | Unclear | 6 | -0.317 (-0.739 to 0.105) | 74.9 | 0.001 |  |
|  |  | Intervention duration (week) |  |  |  |  | 0.439 |
|  |  | ≥16 | 2 | -0.259 (-0.785 to 0.267) | 77.5 | 0.035 |  |
|  |  | 12 to <16 | 14 | -0.110 (-0.302 to 0.081) | 51.9 | 0.012 |  |
|  |  | 8 to <12 | 4 | -0.490 (-1.254 to 0.274) | 82.7 | 0.001 |  |
|  |  | <8 | 10 | -0.389 (-0.719 to -0.059) | 73.1 | <0.001 |  |
|  |  | Sex |  |  |  |  | <0.001 |
|  |  | Men and women | 28 | -0.215 (-0.363 to -0.067) | 60.0 | <0.001 |  |
|  |  | Men | 1 | -1.700 (-2.447 to -0.953) | - | - |  |
|  |  | Women | 1 | 0.100 (-0.218 to 0.418) | - | - |  |
|  |  | Baseline risk‡‡ |  |  |  |  | 0.927 |
|  |  | Low risk | 5 | -0.204 (-0.664 to 0.255) | 49.2 | 0.096 |  |
|  |  | High risk | 26 | -0.227 (-0.409 to -0.046) | 74.7 | <0.001 |  |
|  |  | Diabetes status |  |  |  |  | 0.172 |
|  |  | Diabetic | 4 | -0.684 (-1.244 to -0.123) | 37.5 | 0.187 |  |
|  |  | Non-diabetic | 24 | -0.167 (-0.315 to -0.020) | 60.6 | <0.001 |  |
|  |  | Mixed | 2 | -0.802 (-2.506 to 0.902) | 93.6 | <0.001 |  |
|  |  | Comparator type⁋ |  |  |  |  | 0.066 |
|  |  | Minimal intervention | 4 | -0.463 (-0.698 to -0.229) | 0 | 0.791 |  |
|  |  | Low fiber/ refined grains | 25 | -0.205 (-0.370 to -0.039) | 67.9 | <0.001 |  |
|  |  | Healthy diet | 1 | -0.962 (-1.820 to -0.104) | - | - |  |
|  |  | Use of run-in period |  |  |  |  | 0.261 |
|  |  | Yes | 10 | -0.134 (-0.329 to 0.061) | 38.4 | 0.102 |  |
|  |  | No | 20 | -0.300 (-0.514 to -0.086) | 73.2 | <0.001 |  |

Abbreviations: MD: mean difference-CI: confidence interval- FPG: fasting plasma glucose- HbA_1c_: glycated haemoglobin- HOMA-IR: homeostatic model assessment for insulin resistance

*Obtained from random effects model.

†Inconsistency—percentage of variation across studies due to heterogeneity.

‡Obtained from Q test.

**Studies that recruited adults with one or more of the following criteria: fasting plasma glucose ≥5.6 mmol/L, serum triglycerides ≥1.7 mmol/L, serum total cholesterol level ≥5.2 mmol/L, serum LDL cholesterol level ≥3.4 mmol/L, fasting serum HDL cholesterol <1.29 mmol/L (women) or <1.03 mmol/L (men), and/or blood pressure ≥130/≥85 mm Hg were included in the at risk subgroup, and studies that recruited participants without considering these criteria were included in the healthy subgroup.

¥Studies that reported findings separately for metabolically healthy and at-risk individuals were also separately included in the subgroup analysis.

††Studies that examined two types of whole grains were analysed separately in the subgroup analysis.

‡‡High baseline risk was defined as individuals with metabolic disease, medication users, or those who are overweight/obese.

⁋The minimum intervention was defined as the usual or conventional diets, routine care, no intervention or placebo, while low fiber/refined grains were defined as refined wheat, refined corn, white rice, or mixed refined grains.

### Supplementary Table 14: Subgroup analyses on the effect of whole grains on blood lipids in adults aged ≥18 years (pairwise meta-analyses).

|  |  |  | No of effect sizes | Pooled MD  (95% CI)* | I^2^ (%)† | P-heterogeneity‡ | P-group difference* |
| --- | --- | --- | --- | --- | --- | --- | --- |
| **Triglycerides** | | |  |  |  |  |  |
|  | **Overall** | | 70 | -0.052 (-0.087 to -0.018) | 48.2 | <0.001 |  |
|  |  | **Predefined subgroup analyses** |  |  |  |  |  |
|  |  | Risk of bias |  |  |  |  | 0.153 |
|  |  | Low risk | 2 | -0.180 (-0.355 to -0.004) | 0 | 0.824 |  |
|  |  | High risk/some concerns | 68 | -0.049 (-0.084 to -0.014) | 48.8 | <0.001 |  |
|  |  | Health status**¥ |  |  |  |  | 0.222 |
|  |  | Metabolically healthy | 27 | -0.027 (-0.072 to 0.017) | 45.2 | 0.006 |  |
|  |  | At risk | 44 | -0.069 (-0.120 to -0.019) | 47.5 | <0.001 |  |
|  |  | Intervention duration (week) |  |  |  |  | 0.587 |
|  |  | ≥8 | 37 | -0.063 (-0.115 to -0.012) | 50.8 | <0.001 |  |
|  |  | <8 | 33 | -0.044 (-0.091 to 0.003) | 46.4 | 0.002 |  |
|  |  | **Post hoc subgroup analyses** |  |  |  |  |  |
|  |  | Whole grain type†† |  |  |  |  | <0.001 |
|  |  | Mixed | 16 | 0.016 (-0.030 to 0.062) | 0 | 0.714 |  |
|  |  | Whole wheat | 7 | -0.105 (-0.227 to 0.018) | 41.4 | 0.115 |  |
|  |  | Brown rice | 16 | -0.133 (-0.237 to -0.029) | 48.3 | 0.016 |  |
|  |  | Barley | 3 | -0.071 (-0.180 to 0.037) | 52.6 | 0.096 |  |
|  |  | Oat | 19 | -0.073 (-0.108 to -0.038) | 0 | 0.624 |  |
|  |  | Rye | 4 | 0.042 (-0.012 to 0.096) | 0 | 0.544 |  |
|  |  | Quinoa | 4 | -0.002 (-0.346 to 0.343) | 81.1 | 0.001 |  |
|  |  | Buckwheat | 1 | 0.020 (-0.365 to 0.405) | - | - |  |
|  |  | Amaranth | 1 | 0.330 ( 0.031 to 0.629) | - | - |  |
|  |  | Corn | 1 | 0.235 ( 0.035 to 0.434) | - | - |  |
|  |  | Study design |  |  |  |  | 0.233 |
|  |  | Parallel | 51 | -0.067 (-0.113 to -0.021) | 52.9 | <0.001 |  |
|  |  | Crossover | 19 | -0.027 (-0.074 to 0.021) | 33.0 | 0.082 |  |
|  |  | Calorie restriction |  |  |  |  | 0.047 |
|  |  | Yes | 9 | 0.013 (-0.049 to 0.076) | 6.5 | 0.381 |  |
|  |  | No | 61 | -0.061 (-0.098 to -0.023) | 49.4 | <0.001 |  |
|  |  | Physical activity |  |  |  |  | 0.493 |
|  |  | Yes | 1 | 0.058 (-0.258 to 0.373) | - | - |  |
|  |  | No | 69 | -0.053 (-0.088 to -0.019) | 48.8 | <0.001 |  |
|  |  | Behavioral support |  |  |  |  | 0.635 |
|  |  | Yes | 12 | -0.031 (-0.128 to 0.065) | 67.5 | <0.001 |  |
|  |  | No | 58 | -0.056 (-0.093 to -0.020) | 41.5 | 0.001 |  |
|  |  | **Exploratory subgroup analyses** |  |  |  |  |  |
|  |  | Use of lipid-lowering medications |  |  |  |  | 0.363 |
|  |  | Yes | 6 | 0.030 (-0.097 to 0.157) | 0 | 0.796 |  |
|  |  | No | 48 | -0.050 (-0.088 to -0.012) | 47.1 | <0.001 |  |
|  |  | Unclear | 16 | -0.088 (-0.191 to 0.015) | 62.4 | <0.001 |  |
|  |  | Intervention duration (week) |  |  |  |  | 0.915 |
|  |  | ≥16 | 5 | -0.082 (-0.325 to 0.162) | 69.6 | 0.010 |  |
|  |  | 12 to <16 | 21 | -0.075 (-0.156 to 0.006) | 63.1 | <0.001 |  |
|  |  | 8 to <12 | 11 | -0.046 (-0.099 to 0.007) | 0 | 0.844 |  |
|  |  | <8 | 33 | -0.044 (-0.091 to 0.003) | 46.4 | 0.002 |  |
|  |  | Sex |  |  |  |  | 0.280 |
|  |  | Men and women | 61 | -0.060 (-0.100 to -0.021) | 50.4 | <0.001 |  |
|  |  | Men | 2 | 0.068 (-0.087 to 0.224) | 0 | 0.625 |  |
|  |  | Women | 7 | -0.040 (-0.112 to 0.031) | 35.7 | 0.156 |  |
|  |  | Baseline risk‡‡ |  |  |  |  | 0.931 |
|  |  | Low risk | 15 | -0.055 (-0.107 to -0.002) | 31.0 | 0.122 |  |
|  |  | High risk | 56 | -0.052 (-0.094 to -0.009) | 51.4 | <0.001 |  |
|  |  | Dyslipidaemia status |  |  |  |  | 0.081 |
|  |  | Dyslipidemia | 18 | -0.054 (-0.112 to 0.003) | 42.5 | 0.030 |  |
|  |  | Non-dyslipidemia | 1 | -0.141 (-0.213 to -0.069) | - | - |  |
|  |  | Mixed | 51 | -0.048 (-0.091 to -0.004) | 47.4 | <0.001 |  |
|  |  | Comparator type⁋ |  |  |  |  | 0.331 |
|  |  | Minimal intervention | 13 | -0.113 (-0.200 to -0.025) | 43.5 | 0.047 |  |
|  |  | Low fiber/ refined grains | 53 | -0.040 (-0.078 to -0.002) | 41.9 | 0.001 |  |
|  |  | Healthy diet | 4 | -0.047 (-0.213 to 0.120) | 78.1 | 0.003 |  |
|  |  | Use of run-in period |  |  |  |  | 0.046 |
|  |  | Yes | 14 | -0.001 (-0.048 to 0.045) | 17.5 | 0.263 |  |
|  |  | No | 56 | -0.065 (-0.107 to -0.023) | 49.2 | <0.001 |  |
| **Total cholesterol** | | |  |  |  |  |  |
|  | **Overall** | | 73 | -0.168 (-0.218 to -0.118) | 71.5 | <0.001 |  |
|  |  | **Predefined subgroup analyses** |  |  |  |  |  |
|  |  | Risk of bias |  |  |  |  | 0.493 |
|  |  | Low risk | 2 | -0.505 (-1.495 to 0.485) | 95.2 | <0.001 |  |
|  |  | High risk/some concerns | 71 | -0.158 (-0.207 to -0.110) | 67.8 | <0.001 |  |
|  |  | Health status**¥ |  |  |  |  | 0.211 |
|  |  | Metabolically healthy | 29 | -0.123 (-0.188 to -0.058) | 39.4 | 0.017 |  |
|  |  | At risk | 45 | -0.184 (-0.254 to -0.114) | 78.7 | <0.001 |  |
|  |  | Intervention duration (week) |  |  |  |  | 0.081 |
|  |  | ≥8 | 36 | -0.121 (-0.191 to -0.052) | 79.1 | <0.001 |  |
|  |  | <8 | 37 | -0.213 (-0.289 to -0.138) | 56.8 | <0.001 |  |
|  |  | **Post hoc subgroup analyses** |  |  |  |  |  |
|  |  | Whole grain type†† |  |  |  |  | 0.001 |
|  |  | Mixed | 17 | -0.046 (-0.126 to 0.033) | 30.5 | 0.113 |  |
|  |  | Whole wheat | 7 | -0.111 (-0.330 to 0.109) | 64.0 | 0.011 |  |
|  |  | Brown rice | 16 | -0.154 (-0.283 to -0.025) | 78.2 | <0.001 |  |
|  |  | Barley | 3 | -0.260 (-0.540 to 0.020) | 39.0 | 0.178 |  |
|  |  | Oat | 21 | -0.304 (-0.383 to -0.225) | 60.5 | <0.001 |  |
|  |  | Rye | 4 | -0.024 (-0.259 to 0.210) | 77.2 | 0.004 |  |
|  |  | Quinoa | 4 | -0.025 -0.204 0.155 | 0 | 0.868 |  |
|  |  | Buckwheat | 1 | -0.260 (-0.480 to -0.040) | - | - |  |
|  |  | Amaranth | 1 | 0.020 (-0.403 to 0.443) | - | - |  |
|  |  | Corn | 1 | -0.034 (-0.226 to 0.159) | - | - |  |
|  |  | Study design |  |  |  |  | 0.790 |
|  |  | Parallel | 53 | -0.163 (-0.217 to -0.109) | 65.5 | <0.001 |  |
|  |  | Crossover | 20 | -0.182 (-0.310 to -0.054) | 79.9 | <0.001 |  |
|  |  | Calorie restriction |  |  |  |  | 0.902 |
|  |  | Yes | 9 | -0.174 (-0.294 to -0.055) | 72.4 | <0.001 |  |
|  |  | No | 64 | -0.166 (-0.224 to -0.109) | 66.3 | <0.001 |  |
|  |  | Physical activity |  |  |  |  | 0.767 |
|  |  | Yes | 2 | -0.142 (-0.312 to 0.028) | 0 | 0.895 |  |
|  |  | No | 71 | -0.169 (-0.221 to -0.117) | 72.2 | <0.001 |  |
|  |  | Behavioral support |  |  |  |  | 0.400 |
|  |  | Yes | 13 | -0.136 (-0.201 to -0.070) | 13.6 | 0.308 |  |
|  |  | No | 60 | -0.174 (-0.234 to -0.113) | 75.1 | <0.001 |  |
|  |  | **Explanatory subgroups** |  |  |  |  |  |
|  |  | Use of lipid-lowering medications |  |  |  |  | 0.125 |
|  |  | Yes | 6 | -0.001 (-0.185 to 0.182) | 56.2 | 0.044 |  |
|  |  | No | 52 | -0.192 (-0.254 to -0.130) | 68.1 | <0.001 |  |
|  |  | Unclear | 15 | -0.135 (-0.234 to -0.036) | 56.3 | 0.004 |  |
|  |  | Intervention duration (week) |  |  |  |  | 0.259 |
|  |  | ≥16 | 5 | -0.046 (-0.219 to 0.126) | 51.7 | 0.082 |  |
|  |  | 12 to <16 | 21 | -0.126 (-0.222 to -0.030) | 58.5 | <0.001 |  |
|  |  | 8 to <12 | 11 | -0.153 (-0.291 to -0.014) | 60.7 | 0.005 |  |
|  |  | <8 | 36 | -0.213 (-0.289 to -0.138) | 79.1 | <0.001 |  |
|  |  | Sex |  |  |  |  | 0.359 |
|  |  | Men and women | 63 | -0.180 (-0.235 to -0.126) | 73.4 | <0.001 |  |
|  |  | Men | 2 | -0.045 (-0.277 to 0.188) | 0 | 0.477 |  |
|  |  | Women | 8 | -0.089 (-0.265 to 0.086) | 62.7 | 0.009 |  |
|  |  | Baseline risk‡‡ |  |  |  |  | 0.848 |
|  |  | Low risk | 17 | -0.155 (-0.264 to -0.047) | 59.0 | 0.001 |  |
|  |  | High risk | 57 | -0.167 (-0.225 to -0.109) | 73.7 | <0.001 |  |
|  |  | Dyslipidaemia status |  |  |  |  | 0.001 |
|  |  | Dyslipidemia | 20 | -0.218 (-0.314 to -0.121) | 73.2 | <0.001 |  |
|  |  | Non-dyslipidemia | 4 | -0.412 (-0.568 to -0.257) | 0 | 0.534 |  |
|  |  | Mixed | 49 | -0.120 (-0.176 to -0.065) | 59.7 | <0.001 |  |
|  |  | Comparator type⁋ |  |  |  |  | 0.099 |
|  |  | Minimal intervention | 12 | -0.263 (-0.374 to -0.153) | 35.3 | 0.108 |  |
|  |  | Low fiber/ refined grains | 57 | -0.153 (-0.213 to -0.094) | 74.4 | <0.001 |  |
|  |  | Healthy diet | 4 | -0.115 (-0.196 to -0.034) | 0 | 0.593 |  |
|  |  | Use of run-in period |  |  |  |  | 0.003 |
|  |  | Yes | 16 | -0.040 (-0.137 to 0.057) | 65.7 | <0.001 |  |
|  |  | No | 57 | -0.211 (-0.272 to -0.151) | 73.1 | <0.001 |  |
| **LDL-C** | | |  |  |  |  |  |
|  | **Overall** | | 69 | -0.127 (-0.171 to -0.083) | 57.9 | <0.001 |  |
|  |  | **Predefined subgroup analyses** |  |  |  |  |  |
|  |  | Risk of bias |  |  |  |  | 0.713 |
|  |  | Low risk | 2 | -0.066 (-0.389 to 0.256) | 0 | 0.609 |  |
|  |  | High risk/some concerns | 67 | -0.127 (-0.172 to -0.083) | 59.0 | <0.001 |  |
|  |  | Health status**¥ |  |  |  |  | 0.403 |
|  |  | Metabolically healthy | 25 | -0.100 (-0.160 to -0.039) | 38.5 | 0.027 |  |
|  |  | At risk | 45 | -0.136 (-0.195 to -0.077) | 64.1 | <0.001 |  |
|  |  | Intervention duration (week) |  |  |  |  | 0.153 |
|  |  | ≥8 | 38 | -0.097 (-0.169 to -0.026) | 68.0 | <0.001 |  |
|  |  | <8 | 31 | -0.159 (-0.206 to -0.113) | 27.4 | 0.081 |  |
|  |  | **Post hoc subgroup analyses** |  |  |  |  |  |
|  |  | Whole grain type†† |  |  |  |  | 0.001 |
|  |  | Mixed | 16 | -0.007 (-0.069 to 0.054) | 0 | 0.662 |  |
|  |  | Whole wheat | 6 | -0.090 (-0.340 to 0.159) | 69.3 | 0.006 |  |
|  |  | Brown rice | 15 | -0.113 (-0.243 to 0.016) | 76.9 | <0.001 |  |
|  |  | Barley | 3 | -0.168 (-0.365 to 0.029) | 37.2 | 0.189 |  |
|  |  | Oat | 20 | -0.205 (-0.250 to -0.159) | 0 | 0.646 |  |
|  |  | Rye | 4 | -0.056 (-0.272 to 0.159) | 82.0 | 0.001 |  |
|  |  | Quinoa | 4 | -0.045 (-0.204 to 0.115) | 0 | 0.844 |  |
|  |  | Buckwheat | 1 | -0.240 (-0.420 to -0.060) | - | - |  |
|  |  | Amaranth | 1 | -0.070 (-0.394 to 0.254) | - | - |  |
|  |  | Corn | 1 | -0.132 (-0.314 to 0.051) | - | - |  |
|  |  | Study design |  |  |  |  | 0.101 |
|  |  | Parallel | 51 | -0.148 (-0.200 to -0.097) | 58.5 | <0.001 |  |
|  |  | Crossover | 18 | -0.067 (-0.149 to 0.015) | 53.3 | 0.004 |  |
|  |  | Calorie restriction |  |  |  |  | 0.426 |
|  |  | Yes | 9 | -0.161 (-0.254 to -0.068) | 58.9 | 0.012 |  |
|  |  | No | 60 | -0.118 (-0.169 to -0.068) | 58.5 | <0.001 |  |
|  |  | Physical activity |  |  |  |  | 0.702 |
|  |  | Yes | 2 | -0.155 (-0.297 to -0.013) | 0 | 0.704 |  |
|  |  | No | 67 | -0.126 (-0.171 to -0.080) | 59.1 | <0.001 |  |
|  |  | Behavioral support |  |  |  |  | 0.421 |
|  |  | Yes | 13 | -0.153 (-0.222 to -0.084) | 39.5 | 0.070 |  |
|  |  | No | 56 | -0.117 (-0.171 to -0.063) | 61.1 | <0.001 |  |
|  |  | **Exploratory subgroup analyses** |  |  |  |  |  |
|  |  | Use of lipid-lowering medications |  |  |  |  | 0.439 |
|  |  | Yes | 6 | -0.027 (-0.192 to 0.138) | 58.1 | 0.036 |  |
|  |  | No | 48 | -0.134 (-0.189 to -0.079) | 61.2 | <0.001 |  |
|  |  | Unclear | 15 | -0.145 (-0.229 to -0.061) | 46.6 | 0.024 |  |
|  |  | Intervention duration (week) |  |  |  |  | 0.313 |
|  |  | ≥16 | 5 | -0.030 (-0.253 to 0.193) | 76.7 | 0.002 |  |
|  |  | 12 to <16 | 23 | -0.126 (-0.221 to -0.032) | 69.6 | <0.001 |  |
|  |  | 8 to <12 | 10 | -0.054 (-0.177 to 0.068) | 52.3 | 0.026 |  |
|  |  | <8 | 31 | -0.159 (-0.206 to -0.113) | 27.4 | 0.081 |  |
|  |  | Sex |  |  |  |  | 0.523 |
|  |  | Men and women | 61 | -0.137 (-0.184 to -0.091) | 55.6 | <0.001 |  |
|  |  | Men | 2 | -0.062 (-0.250 to 0.127) | 0 | 0.718 |  |
|  |  | Women | 6 | -0.053 (-0.234 to 0.127) | 79.2 | <0.001 |  |
|  |  | Baseline risk‡‡ |  |  |  |  | 0.912 |
|  |  | Low risk | 15 | -0.129 (-0.223 to -0.035) | 47.9 | 0.02 |  |
|  |  | High risk | 55 | -0.123 (-0.174 to -0.073) | 60.7 | <0.001 |  |
|  |  | Dyslipidaemia status |  |  |  |  | 0.006 |
|  |  | Dyslipidemia | 19 | -0.183 (-0.282 to -0.085) | 75.7 | <0.001 |  |
|  |  | Non-dyslipidemia | 2 | -0.350 (-0.535 to -0.165) | 59.9 | 0.114 |  |
|  |  | Mixed | 48 | -0.083 (-0.122 to -0.044) | 18.0 | 0.144 |  |
|  |  | Comparator type⁋ |  |  |  |  | 0.642 |
|  |  | Minimal intervention | 12 | -0.173 (-0.285 to -0.062) | 55.1 | 0.011 |  |
|  |  | Low fiber/ refined grains | 53 | -0.115 (-0.167 to -0.063) | 59.6 | <0.001 |  |
|  |  | Healthy diet | 4 | -0.136 (-0.264 to -0.008) | 44.0 | 0.147 |  |
|  |  | Use of run-in period |  |  |  |  | 0.101 |
|  |  | Yes | 15 | -0.068 (-0.148 to 0.013) | 59.9 | 0.001 |  |
|  |  | No | 54 | -0.148 (-0.200 to -0.096) | 56.4 | <0.001 |  |
| **HDL-C** | | |  |  |  |  |  |
|  | **Overall** | | 71 | 0.009 (-0.013 to 0.032) | 82.0 | <0.001 |  |
|  |  | **Predefined subgroup analyses** |  |  |  |  |  |
|  |  | Risk of bias |  |  |  |  | 0.741 |
|  |  | Low risk | 2 | 0.028 (-0.084 to 0.140) | 0 | 0.905 |  |
|  |  | High risk/some concerns | 69 | 0.009 (-0.013 to 0.032) | 82.4 | <0.001 |  |
|  |  | Health status**¥ |  |  |  |  | 0.017 |
|  |  | Metabolically healthy | 27 | -0.021 (-0.040 to -0.002) | 23.0 | 0.142 |  |
|  |  | At risk | 45 | 0.025 (-0.008 to 0.057) | 87.4 | <0.001 |  |
|  |  | Intervention duration (week) |  |  |  |  | 0.077 |
|  |  | ≥8 | 38 | 0.028 (-0.004 to 0.061) | 83.1 | <0.001 |  |
|  |  | <8 | 33 | -0.012 (-0.043 to 0.019) | 80.1 | <0.001 |  |
|  |  | **Post hoc subgroup analyses** |  |  |  |  |  |
|  |  | Whole grain type†† |  |  |  |  | 0.005 |
|  |  | Mixed | 16 | -0.047 (-0.066 to -0.027) | 0 | 0.542 |  |
|  |  | Whole wheat | 7 | 0.036 (-0.039 to 0.110) | 55.5 | 0.036 |  |
|  |  | Brown rice | 17 | 0.069 (-0.013 to 0.151) | 94.4 | <0.001 |  |
|  |  | Barley | 3 | -0.032 (-0.121 to 0.057) | 0 | 0.997 |  |
|  |  | Oat | 19 | 0.009 (-0.009 to 0.027) | 12.1 | 0.304 |  |
|  |  | Rye | 4 | -0.024 (-0.081 to 0.033) | 53.6 | 0.091 |  |
|  |  | Quinoa | 4 | 0.031 (-0.105 to 0.168) | 83.0 | 0.001 |  |
|  |  | Buckwheat | 1 | -0.020 (-0.054 to 0.014) | - | - |  |
|  |  | Amaranth | 1 | 0.000 (-0.093 to 0.093) | - | - |  |
|  |  | Corn | 1 | -0.010 (-0.092 to 0.072) | - | - |  |
|  |  | Study design |  |  |  |  | 0.358 |
|  |  | Parallel | 52 | 0.015 (-0.013 to 0.042) | 85.4 | <0.001 |  |
|  |  | Crossover | 19 | -0.005 (-0.037 to 0.027) | 54.3 | 0.003 |  |
|  |  | Calorie restriction |  |  |  |  | 0.228 |
|  |  | Yes | 9 | -0.014 (-0.051 to 0.022) | 59.4 | 0.011 |  |
|  |  | No | 62 | 0.013 (-0.012 to 0.039) | 83.3 | <0.001 |  |
|  |  | Physical activity |  |  |  |  | 0.091 |
|  |  | Yes | 1 | -0.036 (-0.085 to 0.013) | - | - |  |
|  |  | No | 70 | 0.010 (-0.013 to 0.033) | 82.1 | <0.001 |  |
|  |  | Behavioral support |  |  |  |  | 0.353 |
|  |  | Yes | 12 | 0.034 (-0.025 to 0.093) | 89.9 | <0.001 |  |
|  |  | No | 59 | 0.004 (-0.020 to 0.028) | 79.2 | <0.001 |  |
|  |  | **Exploratory subgroup analyses** |  |  |  |  |  |
|  |  | Use of lipid-lowering medications |  |  |  |  | 0.104 |
|  |  | Yes | 6 | 0.026 (-0.050 to 0.103) | 82.6 | <0.001 |  |
|  |  | No | 49 | 0.021 (-0.005 to 0.047) | 79.2 | <0.001 |  |
|  |  | Unclear | 16 | -0.037 (-0.085 to 0.011) | 82.4 | <0.001 |  |
|  |  | Intervention duration (week) |  |  |  |  | 0.331 |
|  |  | ≥16 | 5 | 0.042 (-0.082 to 0.166) | 90.2 | <0.001 |  |
|  |  | 12 to <16 | 22 | 0.037 (-0.009 to 0.082) | 86.8 | <0.001 |  |
|  |  | 8 to <12 | 11 | -0.001 (-0.041 to 0.039) | 45.3 | 0.051 |  |
|  |  | <8 | 33 | -0.012 (-0.043 to 0.019) | 80.1 | <0.001 |  |
|  |  | Sex |  |  |  |  | 0.274 |
|  |  | Men and women | 62 | 0.013 (-0.012 to 0.037) | 84.0 | <0.001 |  |
|  |  | Men | 2 | -0.020 (-0.094 to 0.054) | 0 | 1.00 |  |
|  |  | Women | 7 | -0.023 (-0.062 to 0.017) | 0 | 0.426 |  |
|  |  | Baseline risk‡‡ |  |  |  |  | 0.672 |
|  |  | Low risk | 15 | 0.002 (-0.029 to 0.033) | 18.8 | 0.244 |  |
|  |  | High risk | 57 | 0.011 (-0.015 to 0.036) | 84.9 | <0.001 |  |
|  |  | Dyslipidaemia status |  |  |  |  | 0.463 |
|  |  | Dyslipidemia | 19 | 0.026 (-0.006 to 0.059) | 69.7 | <0.001 |  |
|  |  | Non-dyslipidemia | 2 | -0.015 (-0.083 to 0.054) | 0 | 0.699 |  |
|  |  | Mixed | 50 | 0.005 (-0.024 to 0.033) | 83.9 | <0.001 |  |
|  |  | Comparator type⁋ |  |  |  |  | 0.531 |
|  |  | Minimal intervention | 12 | 0.022 (-0.048 to 0.092) | 81.9 | <0.001 |  |
|  |  | Low fiber/ refined grains | 55 | 0.009 (-0.017 to 0.035) | 83.4 | <0.001 |  |
|  |  | Healthy diet | 4 | -0.013 (-0.048 to 0.022) | 0.00 | 0.976 |  |
|  |  | Use of run-in period |  |  |  |  | 0.049 |
|  |  | Yes | 14 | -0.016 (-0.033 to -0.000) | 0 | 0.559 |  |
|  |  | No | 57 | 0.016 (-0.012 to 0.045) | 85.1 | <0.001 |  |

Abbreviations: MD: mean difference- CI: confidence interval- LDL-c: low-density lipoprotein cholesterol- HDL-c: high-density lipoprotein cholesterol

*Obtained from random effects model.

†Inconsistency—percentage of variation across studies due to heterogeneity.

‡Obtained from Q test.

**Studies that recruited adults with one or more of the following criteria: fasting plasma glucose ≥5.6 mmol/L, serum triglycerides ≥1.7 mmol/L, serum total cholesterol level ≥5.2 mmol/L, serum LDL cholesterol level ≥3.4 mmol/L, fasting serum HDL cholesterol <1.29 mmol/L (women) or <1.03 mmol/L (men), and/or blood pressure ≥130/≥85 mm Hg were included in the at risk subgroup, and studies that recruited participants without considering these criteria were included in the healthy subgroup.

¥Studies that reported findings separately for metabolically healthy and at-risk individuals were also separately included in the subgroup analysis.

††Studies that examined two types of whole grains were analysed separately in the subgroup analysis.

‡‡High baseline risk was defined as individuals with metabolic disease, medication users, or those who are overweight/obese.

⁋The minimum intervention was defined as the usual or conventional diets, routine care, no intervention or placebo, while low fiber/refined grains were defined as refined wheat, refined corn, white rice, or mixed refined grains.

### Supplementary Table 15: Subgroup analyses on the effect of whole grains on blood pressure in adults aged ≥18 years (pairwise meta-analyses).

|  |  |  | No of effect sizes | Pooled MD  (95% CI)* | I^2^ (%)† | P-heterogeneity‡ | P-group difference* |
| --- | --- | --- | --- | --- | --- | --- | --- |
| **SBP** | | |  |  |  |  |  |
|  | **Overall** | | 46 | -1.500 (-2.623 to -0.377) | 62.5 | <0.001 |  |
|  |  | **Predefined subgroup analyses** |  |  |  |  |  |
|  |  | Risk of bias |  |  |  |  | 0.493 |
|  |  | Low risk | 1 | -4.000 (-11.17 to 3.175) | - | - |  |
|  |  | High risk/some concerns | 45 | -1.461 (-2.598 to -0.324) | 63.2 | <0.001 |  |
|  |  | Health status** |  |  |  |  | 0.552 |
|  |  | Metabolically healthy | 16 | -1.026 (-2.748 to 0.697) | 55.0 | 0.004 |  |
|  |  | At risk | 30 | -1.705 (-3.135 to -0.274) | 63.6 | <0.001 |  |
|  |  | Intervention duration (week) |  |  |  |  | 0.606 |
|  |  | ≥8 | 29 | -1.748 (-3.356 to -0.139) | 70.5 | <0.001 |  |
|  |  | <8 | 17 | -1.192 (-2.559 to 0.174) | 33.8 | 0.086 |  |
|  |  | **Post hoc subgroup analyses** |  |  |  |  |  |
|  |  | Whole grain type†† |  |  |  |  | 0.310 |
|  |  | Mixed | 14 | -0.387 (-1.946 to 1.172) | 52.6 | 0.011 |  |
|  |  | Whole wheat | 6 | -3.036 (-6.739 to 0.667) | 65.9 | 0.012 |  |
|  |  | Brown rice | 12 | -2.335 (-5.507 to 0.837) | 72.7 | <0.001 |  |
|  |  | Barley | 2 | -3.650 (-8.296 to 0.995) | 32.4 | 0.228 |  |
|  |  | Oat | 10 | -1.850 (-4.396 to 0.697) | 49.5 | 0.031 |  |
|  |  | Rye | 1 | 1.110 (-1.230 to 3.450) | - | - |  |
|  |  | Quinoa | 2 | -1.218 (-3.982 to 1.546) | 0 | 0.949 |  |
|  |  | Buckwheat | 1 | -3.530 (-8.492 to 1.432) | - | - |  |
|  |  | Amaranth | - | - | - | - |  |
|  |  | Corn | - | - | - | - |  |
|  |  | Study design |  |  |  |  | 0.924 |
|  |  | Parallel | 35 | -1.488 (-2.872 to -0.104) | 66.4 | <0.001 |  |
|  |  | Crossover | 11 | -1.600 (-3.444 to 0.243) | 47.2 | 0.041 |  |
|  |  | Calorie restriction |  |  |  |  | 0.283 |
|  |  | Yes | 8 | -0.143 (-2.947 to 2.660) | 65.6 | 0.005 |  |
|  |  | No | 38 | -1.816 (-3.021 to -0.611) | 59.9 | <0.001 |  |
|  |  | Physical activity |  |  |  |  | 0.016 |
|  |  | Yes | 1 | 4.100 (-0.449 to 8.649) | - | - |  |
|  |  | No | 45 | -1.641 (-2.761 to -0.521) | 61.4 | <0.001 |  |
|  |  | Behavioral support |  |  |  |  | 0.172 |
|  |  | Yes | 8 | 0.076 (-2.444 to 2.596) | 63.3 | 0.008 |  |
|  |  | No | 38 | -1.874 (-3.093 to -0.656) | 59.4 | <0.001 |  |
|  |  | **Exploratory subgroup analyses** |  |  |  |  |  |
|  |  | Use of blood pressure-lowering medications |  |  |  |  | 0.428 |
|  |  | Yes | 10 | -0.628 (-2.035 to 0.779) | 0 | 0.679 |  |
|  |  | No | 26 | -1.685 (-3.318 to -0.052) | 71.9 | <0.001 |  |
|  |  | Unclear | 10 | -2.349 (-4.969 to 0.270) | 57.3 | 0.012 |  |
|  |  | Intervention duration (week) |  |  |  |  | 0.560 |
|  |  | ≥16 | 4 | -0.179 (-2.086 to 1.727) | 0 | 0.817 |  |
|  |  | 12 to <16 | 18 | -1.780 (-3.949 to 0.389) | 77.2 | <0.001 |  |
|  |  | 8 to <12 | 7 | -2.970 (-7.125 to 1.185) | 62.4 | 0.014 |  |
|  |  | <8 | 17 | -1.192 (-2.559 to 0.174) | 33.8 | 0.086 |  |
|  |  | Sex |  |  |  |  | 0.126 |
|  |  | Men and women | 40 | -1.680 (-2.916 to -0.443) | 65.1 | <0.001 |  |
|  |  | Men | 2 | -2.678 (-7.462 to 2.105) | 25.8 | 0.246 |  |
|  |  | Women | 4 | 0.836 (-1.395 to 3.068) | 0 | 0.546 |  |
|  |  | Baseline risk‡‡ |  |  |  |  | 0.606 |
|  |  | Low risk | 9 | -2.301 (-5.712 to 1.110) | 71.1 | 0.001 |  |
|  |  | High risk | 37 | -1.351 (-2.544 to -0.157) | 61.0 | <0.001 |  |
|  |  | Comparator type⁋ |  |  |  |  | 0.779 |
|  |  | Minimal intervention | 8 | -1.787 (-3.719 to 0.146) | 41.3 | 0.103 |  |
|  |  | Low fiber/ refined grains | 38 | -1.452 (-2.775 to -0.129) | 65.5 | <0.001 |  |
|  |  | Healthy diet | 0 |  |  |  |  |
|  |  | Use of run-in period |  |  |  |  | 0.085 |
|  |  | Yes | 10 | -0.082 (-1.737 to 1.574) | 42.2 | 0.076 |  |
|  |  | No | 36 | -1.961 (-3.318 to -0.604) | 63.5 | <0.001 |  |
| **DBP** | | |  |  |  |  |  |
|  | **Overall** | | 45 | -0.820 (-1.589 to -0.052) | 65.4 | <0.001 |  |
|  |  | **Predefined subgroup analyses** |  |  |  |  |  |
|  |  | Risk of bias |  |  |  |  | 0.593 |
|  |  | Low risk | 1 | -1.900 (-5.861 to 2.061) | - | - |  |
|  |  | High risk/some concerns | 44 | -0.799 (-1.580 to -0.018) | 66.1 | <0.001 |  |
|  |  | Health status** |  |  |  |  | 0.885 |
|  |  | Metabolically healthy | 16 | -0.704 (-1.675 to 0.268) | 31.0 | 0.115 |  |
|  |  | At risk | 29 | -0.809 (-1.857 to 0.239) | 73.2 | <0.001 |  |
|  |  | Intervention duration (week) |  |  |  |  | 0.133 |
|  |  | ≥8 | 29 | -1.219 (-2.216 to -0.221) | 70.0 | <0.001 |  |
|  |  | <8 | 16 | -0.064 (-1.193 to 1.065) | 48.3 | 0.016 |  |
|  |  | **Post hoc subgroup analyses** |  |  |  |  |  |
|  |  | Whole grain type†† |  |  |  |  | 0.705 |
|  |  | Mixed | 14 | -0.598 (-1.534 to 0.338) | 49.0 | 0.020 |  |
|  |  | Whole wheat | 6 | -1.376 (-2.950 to 0.198) | 20.7 | 0.278 |  |
|  |  | Brown rice | 12 | -2.013 (-4.612 to 0.585) | 84.0 | <0.001 |  |
|  |  | Barley | 2 | 0.596 (-2.719 to 3.911) | 26.7 | 0.256 |  |
|  |  | Oat | 9 | 0.047 (-1.365 to 1.458) | 26.2 | 0.203 |  |
|  |  | Rye | 1 | 0.020 (-1.696 to 1.736) | - | - |  |
|  |  | Quinoa | 2 | -0.215 (-2.342 to 1.912) | 0 | 0.511 |  |
|  |  | Buckwheat | 1 | 1.030 (-2.595 to 4.655) | - | - |  |
|  |  | Amaranth | - | - | - | - |  |
|  |  | Corn | - | - | - | - |  |
|  |  | Study design |  |  |  |  | 0.786 |
|  |  | Parallel | 34 | -0.893 (-1.755 to -0.030) | 63.4 | <0.001 |  |
|  |  | Crossover | 11 | -0.619 (-2.392 to 1.154) | 72.8 | <0.001 |  |
|  |  | Calorie restriction |  |  |  |  | 0.732 |
|  |  | Yes | 8 | -0.530 (-2.287 to 1.227) | 54.4 | 0.032 |  |
|  |  | No | 37 | -0.871 (-1.731 to -0.011) | 67.4 | <0.001 |  |
|  |  | Physical activity |  |  |  |  | 0.439 |
|  |  | Yes | 1 | 0.600 (-2.989 to 4.189) | - | - |  |
|  |  | No | 44 | -0.850 (-1.632 to -0.069) | 66.0 | <0.001 |  |
|  |  | Behavioral support |  |  |  |  | 0.270 |
|  |  | Yes | 7 | -0.082 (-1.352 to 1.188) | 29.2 | 0.205 |  |
|  |  | No | 38 | -0.952 (-1.834 to -0.071) | 67.9 | <0.001 |  |
|  |  | **Exploratory subgroup analyses** |  |  |  |  |  |
|  |  | Use of blood pressure-lowering     medications |  |  |  |  | 0.767 |
|  |  | Yes | 10 | -0.798 (-1.747 to 0.150) | 12.8 | 0.325 |  |
|  |  | No | 25 | -0.638 (-1.888 to 0.611) | 77.3 | <0.001 |  |
|  |  | Unclear | 10 | -1.229 (-2.372 to -0.087) | 17.5 | 0.282 |  |
|  |  | Intervention duration (week) |  |  |  |  | 0.071 |
|  |  | ≥16 | 4 | 0.136 (-1.044 to 1.317) | 0 | 0.736 |  |
|  |  | 12 to <16 | 18 | -1.206 (-2.667 to 0.256) | 78.9 | <0.001 |  |
|  |  | 8 to <12 | 7 | -1.849 (-3.067 to -0.631) | 0 | 0.526 |  |
|  |  | <8 | 16 | -0.064 (-1.193 to 1.065) | 48.3 | 0.016 |  |
|  |  | Sex |  |  |  |  | 0.858 |
|  |  | Men and women | 39 | -0.868 (-1.694 to -0.041) | 67.7 | <0.001 |  |
|  |  | Men | 2 | -0.053 (-2.992 to 2.887) | 0 | 0.420 |  |
|  |  | Women | 4 | -0.526 (-3.426 to 2.373) | 54.6 | 0.085 |  |
|  |  | Baseline risk‡‡ |  |  |  |  | 0.562 |
|  |  | Low risk | 9 | -1.201 (-2.348 to -0.054) | 0 | 0.982 |  |
|  |  | High risk | 36 | -0.769 (-1.674 to 0.136) | 72.0 | <0.001 |  |
|  |  | Comparator type⁋ |  |  |  |  | 0.698 |
|  |  | Minimal intervention | 8 | -0.603 (-1.680 to 0.473) | 17.3 | 0.294 |  |
|  |  | Low fiber/ refined grains | 37 | -0.883 (-1.802 to 0.035) | 69.5 | <0.001 |  |
|  |  | Healthy diet | 0 | - | - | - |  |
|  |  | Use of run-in period |  |  |  |  | 0.585 |
|  |  | Yes | 10 | -0.490 (-1.406 to 0.426) | 27.1 | 0.194 |  |
|  |  | No | 35 | -0.867 (-1.862 to 0.129) | 70.0 | <0.001 |  |

Abbreviations: MD: mean difference- CI: confidence interval- SBP: systolic blood pressure- DBP: diastolic blood pressure

*Obtained from random effects model.

†Inconsistency—percentage of variation across studies due to heterogeneity.

‡Obtained from Q test.

**Studies that recruited adults with one or more of the following criteria: fasting plasma glucose ≥5.6 mmol/L, serum triglycerides ≥1.7 mmol/L, serum total cholesterol level ≥5.2 mmol/L, serum LDL cholesterol level ≥3.4 mmol/L, fasting serum HDL cholesterol <1.29 mmol/L (women) or <1.03 mmol/L (men), and/or blood pressure ≥130/≥85 mm Hg were included in the at risk subgroup, and studies that recruited participants without considering these criteria were included in the healthy subgroup.

††Studies that examined two types of whole grains were analysed separately in the subgroup analysis.

₤ Includes studies in which all or some participants were using glucose- and/or blood pressure-lowering medications.

‡‡High baseline risk was defined as individuals with metabolic disease, medication users, or those who are overweight/obese.

⁋The minimum intervention was defined as the usual or conventional diets, routine care, no intervention or placebo, while low fiber/refined grains were defined as refined wheat, refined corn, white rice, or mixed refined grains.

### Supplementary Table 16: Subgroup analyses on the effect of whole grains on inflammatory biomarkers in adults aged ≥18 years (pairwise meta-analyses).

|  |  |  | No of effect sizes | Pooled MD  (95% CI)* | I^2^ (%)† | P-heterogeneity‡ | P-group difference* |
| --- | --- | --- | --- | --- | --- | --- | --- |
| **CRP** | | |  |  |  |  |  |
|  | **Overall** | | 31 | -0.381 (-0.550 to -0.212) | 83.1 | <0.001 |  |
|  |  | **Predefined subgroup analyses** |  |  |  |  |  |
|  |  | Risk of bias |  |  |  |  | 0.621 |
|  |  | Low risk | 2 | -1.188 (-4.391 to 2.015) | 75.7 | 0.042 |  |
|  |  | High risk/some concerns | 29 | -0.380 (-0.549 to -0.210) | 83.9 | <0.001 |  |
|  |  | Health status** |  |  |  |  | 0.938 |
|  |  | Metabolically healthy | 13 | -0.353 (-0.699 to -0.007) | 88.7 | <0.001 |  |
|  |  | At risk | 18 | -0.369 (-0.594 to -0.144) | 72.8 | <0.001 |  |
|  |  | Intervention duration (week) |  |  |  |  | 0.686 |
|  |  | ≥8 | 19 | -0.423 (-0.672 to -0.173) | 70.4 | <0.001 |  |
|  |  | <8 | 12 | -0.339 (-0.659 to -0.019) | 89.9 | <0.001 |  |
|  |  | **Post hoc subgroup analyses** |  |  |  |  |  |
|  |  | Whole grain type†† |  |  |  |  | <0.001 |
|  |  | Mixed | 15 | -0.117 (-0.415 to 0.181) | 68.7 | <0.001 |  |
|  |  | Whole wheat | 4 | -0.296 (-0.787 to 0.194) | 20.9 | 0.285 |  |
|  |  | Brown rice | 5 | -0.673 (-1.218 to -0.129) | 83.1 | <0.001 |  |
|  |  | Barley | 1 | -0.165 (-0.468 to 0.139) | - | - |  |
|  |  | Oat | 5 | -0.491 (-0.719 to -0.263) | 0 | 0.565 |  |
|  |  | Rye | 2 | -0.530 (-0.850 to -0.210) | 0 | 0.377 |  |
|  |  | Quinoa | 1 | -0.810 (-0.961 to -0.659) | - | - |  |
|  |  | Buckwheat | - | - | - | - |  |
|  |  | Amaranth | - | - | - | - |  |
|  |  | Study design |  |  |  |  | 0.298 |
|  |  | Parallel | 18 | -0.236 (-0.376 to -0.096) | 60.5 | <0.001 |  |
|  |  | Crossover | 13 | -0.435 (-0.782 to -0.088) | 77.5 | <0.001 |  |
|  |  | Calorie restriction |  |  |  |  | 0.258 |
|  |  | Yes | 6 | -0.604 (-1.017 to -0.192) | 25.2 | 0.245 |  |
|  |  | No | 25 | -0.345 (-0.525 to -0.165) | 85.2 | <0.001 |  |
|  |  | Physical activity |  |  |  |  | 0.058 |
|  |  | Yes | 1 | -2.600 (-4.900 to -0.300) | - | - |  |
|  |  | No | 30 | -0.369 (-0.537 to -0.201) | 83.3 | <0.001 |  |
|  |  | Behavioral support |  |  |  |  | 0.541 |
|  |  | Yes | 6 | -0.526 (-1.023 to -0.029) | 29.8 | 0.212 |  |
|  |  | No | 25 | -0.361 (-0.540 to -0.182) | 85.5 | <0.001 |  |
|  |  | **Exploratory subgroup analyses** |  |  |  |  |  |
|  |  | Intervention duration (week) |  |  |  |  | 0.827 |
|  |  | ≥16 | 1 | -0.142 (-0.983 to 0.700) | - | - |  |
|  |  | 12 to <16 | 13 | -0.486 (-0.770 to -0.202) | 53.2 | 0.012 |  |
|  |  | 8 to <12 | 5 | -0.322 (-1.110 to 0.466) | 81.8 | <0.001 |  |
|  |  | <8 | 12 | -0.339 (-0.659 to -0.019) | 89.9 | <0.001 |  |
|  |  | Sex |  |  |  |  | <0.001 |
|  |  | Men and women | 26 | -0.288 (-0.433 to -0.143) | 69.7 | <0.001 |  |
|  |  | Men | 2 | -0.817 (-0.965 to -0.669) | 0 | 0.639 |  |
|  |  | Women | 3 | -0.321 (-1.425 to 0.782) | 46.8 | 0.153 |  |
|  |  | Baseline risk‡‡ |  |  |  |  | 0.863 |
|  |  | Low risk | 5 | -0.411 (-0.714 to -0.107) | 0 | 0.858 |  |
|  |  | High risk | 26 | -0.379 (-0.563 to -0.195) | 85.6 | <0.001 |  |
|  |  | Comparator type⁋ |  |  |  |  | 0.557 |
|  |  | Minimal intervention | 1 | -0.142 (-0.983 to 0.700) | - | - |  |
|  |  | Low fiber/ refined grains | 29 | -0.378 (-0.551 to -0.205) | 84.1 | <0.001 |  |
|  |  | Healthy diet | 1 | -0.909 (-2.028 to 0.209) | - | - |  |
|  |  | Use of run-in period |  |  |  |  | 0.557 |
|  |  | Yes | 12 | -0.449 (-0.717 to -0.180) | 68.4 | <0.001 |  |
|  |  | No | 19 | -0.333 (-0.610 to -0.057) | 86.5 | <0.001 |  |
| **IL-6** | | |  |  |  |  |  |
|  | **Overall** | | 20 | -0.224 (-0.444 to -0.004) | 67.3 | <0.001 |  |
|  |  | **Predefined subgroup analyses** |  |  |  |  |  |
|  |  | Risk of bias |  |  |  |  | 0.553 |
|  |  | Low risk | 2 | -0.425 (-1.088 to 0.237) | 0 | 0.728 |  |
|  |  | High risk/some concerns | 18 | -0.213 (-0.444 to 0.019) | 70.4 | <0.001 |  |
|  |  | Health status** |  |  |  |  | 0.846 |
|  |  | Metabolically healthy | 7 | -0.265 (-0.527 to -0.003) | 49.8 | 0.063 |  |
|  |  | At risk | 13 | -0.222 (-0.567 to 0.123) | 73.6 | <0.001 |  |
|  |  | Intervention duration (week) |  |  |  |  | 0.688 |
|  |  | ≥8 | 11 | -0.188 (-0.434 to 0.059) | 49.7 | 0.030 |  |
|  |  | <8 | 9 | -0.288 (-0.713 to 0.137) | 79.1 | <0.001 |  |
|  |  | **Post hoc subgroup analyses** |  |  |  |  |  |
|  |  | Whole grain type†† |  |  |  |  | 0.095 |
|  |  | Mixed | 13 | -0.207 (-0.383 to -0.030) | 52.8 | 0.013 |  |
|  |  | Whole wheat | 4 | -0.027 (-0.346 to 0.292) | 13.3 | 0.326 |  |
|  |  | Brown rice | 1 | -3.640 (-7.317 to 0.037) | - | - |  |
|  |  | Barley | - | - | - | - |  |
|  |  | Oat | 3 | -6.785 (-15.93 to 2.357) | 92.0 | <0.001 |  |
|  |  | Rye | - | - | - | - |  |
|  |  | Quinoa | - | - | - | - |  |
|  |  | Buckwheat | - | - | - | - |  |
|  |  | Amaranth | - | - | - | - |  |
|  |  | Study design |  |  |  |  | 0.128 |
|  |  | Parallel | 13 | -0.071 (-0.209 to 0.067) | 0 | 0.555 |  |
|  |  | Crossover | 7 | -0.536 (-1.119 to 0.047) | 86.4 | <0.001 |  |
|  |  | Calorie restriction |  |  |  |  | 0.085 |
|  |  | Yes | 3 | 0.192 (-0.296 to 0.680) | 19.9 | 0.287 |  |
|  |  | No | 17 | -0.285 (-0.522 to -0.048) | 69.5 | <0.001 |  |
|  |  | Physical activity |  |  |  |  | 0.423 |
|  |  | Yes | 1 | -0.800 (-2.220 to 0.620) | - | - |  |
|  |  | No | 19 | -0.213 (-0.436 to 0.010) | 68.6 | <0.001 |  |
|  |  | Behavioral support |  |  |  |  | 0.670 |
|  |  | Yes | 3 | -0.593 (-2.222 to 1.036) | 69.8 | 0.037 |  |
|  |  | No | 17 | -0.235 (-0.457 to -0.014) | 68.5 | <0.001 |  |
|  |  | **Exploratory subgroup analyses** |  |  |  |  |  |
|  |  | Intervention duration (week) |  |  |  |  | 0.216 |
|  |  | ≥16 | - | - | - | - |  |
|  |  | 12 to <16 | 8 | -0.061 (-0.269 to 0.146) | 24.0 | 0.238 |  |
|  |  | 8 to <12 | 3 | -0.618 (-1.275 to 0.038) | 50.5 | 0.133 |  |
|  |  | <8 | 9 | -0.288 (-0.713 to 0.137) | 79.1 | <0.001 |  |
|  |  | Sex |  |  |  |  | 0.846 |
|  |  | Men and women | 18 | -0.218 (-0.444 to 0.008) | 62.4 | <0.001 |  |
|  |  | Men | - | - | - | - |  |
|  |  | Women | 2 | -0.111 (-1.162 to 0.939) | 87.1 | 0.005 |  |
|  |  | Baseline risk‡‡ |  |  |  |  | 0.833 |
|  |  | Low risk | 5 | -0.268 (-0.557 to 0.021) | 66.0 | 0.019 |  |
|  |  | High risk | 15 | -0.220 (-0.554 to 0.113) | 69.4 | <0.001 |  |
|  |  | Comparator type⁋ |  |  |  |  | 0.068 |
|  |  | Minimal intervention | 1 | -3.640 (-7.317 to 0.037) | - | - |  |
|  |  | Low fiber/ refined grains | 19 | -0.210 (-0.426 to 0.005) | 67.1 | <0.001 |  |
|  |  | Healthy diet | 0 | - | - | - |  |
|  |  | Use of run-in period |  |  |  |  | 0.194 |
|  |  | Yes | 7 | -0.101 (-0.257 to 0.055) | 0 | 0.601 |  |
|  |  | No | 13 | -0.388 (-0.792 to 0.016) | 77.2 | <0.001 |  |
| **TNF-α** | | |  |  |  |  |  |
|  | **Overall** | | 14 | -0.038 (-0.157 to 0.081) | 64.4 | <0.001 |  |
|  |  | **Predefined subgroup analyses** |  |  |  |  |  |
|  |  | Risk of bias |  |  |  |  | 0.597 |
|  |  | Low risk | 2 | 4.554 (-12.45 to 21.56) | 46.1 | 0.173 |  |
|  |  | High risk/some concerns | 12 | -0.033 (-0.154 to 0.088) | 68.0 | <0.001 |  |
|  |  | Health status** |  |  |  |  | 0.792 |
|  |  | Metabolically healthy | 6 | -0.110 (-0.599 to 0.379) | 67.0 | 0.010 |  |
|  |  | At risk | 8 | -0.042 (-0.173 to 0.089) | 67.2 | 0.003 |  |
|  |  | Intervention duration (week) |  |  |  |  | 0.189 |
|  |  | ≥8 | 8 | -0.033 (-0.118 to 0.052) | 52.1 | 0.041 |  |
|  |  | <8 | 6 | -0.756 (-1.832 to 0.320) | 77.2 | 0.001 |  |
|  |  | **Post hoc subgroup analyses** |  |  |  |  |  |
|  |  | Whole grain type†† |  |  |  |  | 0.565 |
|  |  | Mixed | 9 | -0.027 (-0.113 to 0.058) | 34.0 | 0.145 |  |
|  |  | Whole wheat | 2 | -47.30 (-151.1 to 56.46) | 88.4 | 0.003 |  |
|  |  | Brown rice | - | - | - | - |  |
|  |  | Barley | - | - | - | - |  |
|  |  | Oat | 3 | -2.233 (-9.582 to 5.116) | 87.0 | <0.001 |  |
|  |  | Rye | - | - | - | - |  |
|  |  | Quinoa | - | - | - | - |  |
|  |  | Buckwheat | - | - | - | - |  |
|  |  | Amaranth | - | - | - | - |  |
|  |  | Study design |  |  |  |  | 0.794 |
|  |  | Parallel | 9 | -0.048 (-0.158 to 0.061) | 57.7 | 0.015 |  |
|  |  | Crossover | 5 | -0.138 (-0.799 to 0.524) | 76.9 | 0.002 |  |
|  |  | Calorie restriction |  |  |  |  | 0.928 |
|  |  | Yes | 2 | -0.051 (-0.278 to 0.176) | 56.2 | 0.131 |  |
|  |  | No | 12 | -0.038 (-0.185 to 0.108) | 66.7 | 0.001 |  |
|  |  | Physical activity |  |  |  |  | 0.234 |
|  |  | Yes | 1 | -0.140 (-0.281 to 0.001) | - | - |  |
|  |  | No | 13 | -0.022 (-0.156 to 0.112) | 64.3 | 0.001 |  |
|  |  | Behavioral support |  |  |  |  | 0.234 |
|  |  | Yes | 1 | -0.140 (-0.281 to 0.001) | - | - |  |
|  |  | No | 13 | -0.022 (-0.156 to 0.112) | 64.3 | 0.001 |  |
|  |  | **Exploratory subgroup analyses** |  |  |  |  |  |
|  |  | Intervention duration (week) |  |  |  |  |  |
|  |  | ≥16 | - | - | - | - | 0.420 |
|  |  | 12 to <16 | 5 | -0.039 (-0.125 to 0.047) | 31.7 | 0.210 |  |
|  |  | 8 to <12 | 3 | -0.016 (-0.272 to 0.240) | 77.1 | 0.013 |  |
|  |  | <8 | 6 | -0.756 (-1.832 to 0.320) | 77.2 | 0.001 |  |
|  |  | Sex |  |  |  |  | 0.153 |
|  |  | Men and women | 13 | -0.055 (-0.176 to 0.066) | 65.4 | 0.001 |  |
|  |  | Men | - | - | - | - |  |
|  |  | Women | 1 | 0.300 (-0.171 to 0.771) | - | - |  |
|  |  | Baseline risk‡‡ |  |  |  |  | 0.726 |
|  |  | Low risk | 4 | -0.021 (-0.124 to 0.082) | 0 | 0.449 |  |
|  |  | High risk | 10 | -0.055 (-0.217 to 0.106) | 73.4 | <0.001 |  |
|  |  | Comparator type⁋ |  |  |  |  | - |
|  |  | Minimal intervention | 0 | - | - | - |  |
|  |  | Low fiber/ refined grains | 14 | -0.038 (-0.157 to 0.081) | 64.4 | <0.001 |  |
|  |  | Healthy diet | 0 | - | - | - |  |
|  |  | Use of run-in period |  |  |  |  | 0.937 |
|  |  | Yes | 5 | -0.014 (-0.085 to 0.058) | 0 | 0.512 |  |
|  |  | No | 9 | -0.023 (-0.239 to 0.193) | 75.8 | <0.001 |  |

Abbreviations: MD: mean difference- CI: confidence interval- CRP: C-reactive protein- IL6: interleukin 6- TNF-α: tumor necrosis factor alpha

*Obtained from random effects model.

†Inconsistency—percentage of variation across studies due to heterogeneity.

‡Obtained from Q test.

**Studies that recruited adults with one or more of the following criteria: fasting plasma glucose ≥5.6 mmol/L, serum triglycerides ≥1.7 mmol/L, serum total cholesterol level ≥5.2 mmol/L, serum LDL cholesterol level ≥3.4 mmol/L, fasting serum HDL cholesterol <1.29 mmol/L (women) or <1.03 mmol/L (men), and/or blood pressure ≥130/≥85 mm Hg were included in the at risk subgroup, and studies that recruited participants without considering these criteria were included in the healthy subgroup.

††Studies that examined two types of whole grains were analysed separately in the subgroup analysis.

₤ Includes studies in which some or all participants were using glucose-, lipid-, and/or blood pressure-lowering medications.

‡‡High baseline risk was defined as individuals with metabolic disease, medication users, or those who are overweight/obese.

⁋The minimum intervention was defined as the usual or conventional diets, routine care, no intervention, or placebo, while low fiber/refined grains were defined as refined wheat, refined corn, white rice, or mixed refined grains.

### Supplementary Table 17: Assessment of credibility of subgroup difference based on ICEMAN

| Variable | Q1 | Q2 | Q3 | Q4 | Q5 | Q6 | Q7 | Q8 | Overall interpretation |
| --- | --- | --- | --- | --- | --- | --- | --- | --- | --- |
| **Weight** |  |  |  |  |  |  |  |  |  |
| Obesity status | Completely between | Not applicable | Large | Definitely no | Chance a likely explanation | Definitely no | Definitely yes | Not applicable | Low |
| **Waist circumference** |  |  |  |  |  |  |  |  |  |
| Baseline risk | Completely between | Not applicable | Very small | Definitely no | Chance a likely explanation | Definitely no | Definitely yes | Not applicable | Low |
| **Body fat percentage** |  |  |  |  |  |  |  |  |  |
| Caloric restriction | Completely between | Not applicable | Rather small | Definitely no | Chance a likely explanation | Probably no | Definitely yes | Not applicable | Low |
| **FPG** |  |  |  |  |  |  |  |  |  |
| Use of run-in period | Completely between | Not applicable | Large | Definitely no | Chance a likely explanation | Definitely no | Definitely yes | Not applicable | Low |
| Diabetes status | Completely between | Not applicable | Rather large | Definitely no | Chance a likely explanation | Definitely no | Definitely yes | Not applicable | Low |
| **HbA1c** |  |  |  |  |  |  |  |  |  |
| Behavioral support | Completely between | Not applicable | Rather large | Definitely no | Chance a very likely explanation | Probably no | Definitely yes | Not applicable | Low |
| Comparator type | Completely between | Not applicable | Very small | Definitely no | Chance a very likely explanation | Definitely no | Definitely yes | Not applicable | Low |
| **Insulin** |  |  |  |  |  |  |  |  |  |
| Risk of bias | Completely between | Not applicable | Very small | Definitely no | Chance a very likely explanation | Definitely yes | Definitely yes | Not applicable | Low |
| Baseline risk | Completely between | Not applicable | Rather large | Definitely no | Chance a very likely explanation | Definitely no | Definitely yes | Not applicable | Low |
| **HOMA-IR** |  |  |  |  |  |  |  |  |  |
| Risk of bias | Completely between | Not applicable | Very small | Definitely no | Chance a likely explanation | Definitely yes | Definitely yes | Not applicable | Low |
| Whole grain type | Mostly between | Not applicable | Very small | Definitely no | Chance an unlikely explanation | Probably no | Definitely yes | Not applicable | Low |
| Caloric restriction | Completely between | Not applicable | Rather small | Definitely no | Chance a likely explanation | Probably no | Definitely yes | Not applicable | Low |
| Comparator type | Completely between | Not applicable | Very small | Definitely no | Chance a very likely explanation | Definitely no | Definitely yes | Not applicable | Low |
| Sex | Completely between | Not applicable | Very small | Definitely no | Chance an unlikely explanation | Definitely no | Definitely yes | Not applicable | Low |
| **Triglycerides** |  |  |  |  |  |  |  |  |  |
| Whole grain type | Mostly between | Not applicable | Very small | Definitely no | Chance an unlikely explanation | Probably no | Definitely yes | Not applicable | Low |
| Caloric restriction | Completely between | Not applicable | Rather large | Definitely no | Chance a likely explanation | Probably no | Definitely yes | Not applicable | Low |
| Dyslipidaemia status | Completely between | Not applicable | Very small | Definitely no | Chance a very likely explanation | Definitely no | Definitely yes | Not applicable | Low |
| Use of run-in period | Completely between | Not applicable | Large | Definitely no | Chance a likely explanation | Definitely no | Definitely yes | Not applicable | Low |
| **Total cholesterol** |  |  |  |  |  |  |  |  |  |
| Intervention duration | Completely between | Not applicable | Large | Definitely yes | Chance a very likely explanation | Definitely yes | Definitely yes | Probably yes | Low |
| Whole grain type | Mostly between | Not applicable | Very small | Definitely no | Chance an unlikely explanation | Probably no | Definitely yes | Not applicable | Low |
| Dyslipidaemia status | Completely between | Not applicable | Rather small | Definitely no | Chance an unlikely explanation | Definitely no | Definitely yes | Not applicable | Low |
| Comparator type | Completely between | Not applicable | Rather small | Definitely no | Chance a very likely explanation | Definitely no | Definitely yes | Not applicable | Low |
| Use of run-in period | Completely between | Not applicable | Large | Definitely no | Chance an unlikely explanation | Definitely no | Definitely yes | Not applicable | Low |
| **LDL-C** |  |  |  |  |  |  |  |  |  |
| Whole grain type | Mostly between | Not applicable | Very small | Definitely no | Chance an unlikely explanation | Probably no | Definitely yes | Not applicable | Low |
| Dyslipidaemia status | Completely between | Not applicable | Very small | Definitely no | Chance may not explain | Definitely no | Definitely yes | Not applicable | Low |
| **HDL-C** |  |  |  |  |  |  |  |  |  |
| Health status | Mostly between | Not applicable | Large | Definitely yes | Chance a likely explanation | Definitely yes | Definitely yes | Not applicable | Low |
| Intervention duration | Completely between | Not applicable | Large | Definitely no | Chance a very likely explanation | Definitely yes | Definitely yes | Probably yes | Low |
| Whole grain type | Mostly between | Not applicable | Very small | Definitely no | Chance an unlikely explanation | Probably no | Definitely yes | Not applicable | Low |
| Physical activity | Completely between | Not applicable | Very small | Definitely no | Chance a very likely explanation | Probably no | Definitely yes | Not applicable | Low |
| Use of run-in period | Completely between | Not applicable | Large | Definitely no | Chance a likely explanation | Definitely no | Definitely yes | Not applicable | Low |
| **SBP** |  |  |  |  |  |  |  |  |  |
| Physical activity | Completely between | Not applicable | Very small | Definitely no | Chance a likely explanation | Probably no | Definitely yes | Not applicable | Low |
| Use of run-in period | Completely between | Not applicable | Large | Definitely no | Chance a very likely explanation | Definitely no | Definitely yes | Not applicable | Low |
| **DBP** |  |  |  |  |  |  |  |  |  |
| Intervention duration | Completely between | Not applicable | Rather small or unclear | Definitely no | Chance a very likely explanation | Definitely no | Definitely yes | Definitely no | Low |
| **CRP** |  |  |  |  |  |  |  |  |  |
| Whole grain type | Mostly between | Not applicable | Very small | Definitely no | Chance an unlikely explanation | Probably no | Definitely yes | Not applicable | Low |
| Physical activity | Completely between | Not applicable | Very small | Definitely no | Chance a very likely explanation | Probably no | Definitely yes | Not applicable | Low |
| Sex | Completely between | Not applicable | Very small | Definitely no | Chance an unlikely explanation | Definitely no | Definitely yes | Not applicable | Low |
| **IL-6** |  |  |  |  |  |  |  |  |  |
| Whole grain type | Completely between | Not applicable | Rather small | Definitely no | Chance a very likely explanation | Probably no | Definitely yes | Not applicable | Low |
| Caloric restriction | Completely between | Not applicable | Rather small | Definitely no | Chance a very likely explanation | Probably no | Definitely yes | Not applicable | Low |
| Comparator type | Completely between | Not applicable | Very small | Definitely no | Chance a very likely explanation | Definitely no | Definitely yes | Not applicable | Low |

Abbreviations: ICEMAN: Instrument to assess the Credibility of Effect Modification Analyses- LDL-C: low-density lipoprotein cholesterol- HDL-C: high-density lipoprotein cholesterol- HbA1c: glycated hemoglobin, HOMA-IR: homeostatic model assessment for insulin resistance- CRP: C-reactive protein- IL-6: interleukin 6- SBP: systolic blood pressure- DBP: diastolic blood pressure.

Q1, Is the analysis of effect modification based on comparison within rather than between trials? Q2, For within-trial comparisons, is the effect modification similar from trial to trial? Q3, For between-trial comparisons, is the number of trials large? Q4, Was the direction of the effect modification correctly hypothesized priori? Q5, Does a test for interaction suggest that chance is an unlikely explanation of the apparent effect modification? Q6, Did the authors test only a small number of effect modifiers? Q7, Did the authors use a random effects model? Q8, If the effect modifier is a continuous variable, were arbitrary cut points avoided?

### Supplementary Table 18: Summary of the effects of whole grain consumption on adiposity measures obtained from non-linear dose-response analysis

| Whole grain (g/d) | Body weight (kg) | Waist circumference (cm) | Body fat percentage | Adverse events |
| --- | --- | --- | --- | --- |
| 0 | Ref | Ref | Ref | Ref |
| 20 | -0.13 (-0.33 to 0.06) | -0.29 (-0.55 to -0.03) | -0.17 (-0.37 to 0.02) | 1.67 (0.63 to 4.41) |
| 40 | -0.24 (-0.56 to 0.07) | -0.50 (-0.93 to -0.08) | -0.31 (-0.65 to 0.03) | 2.09 (0.55 to 7.94) |
| 60 | -0.33 (-0.68 to 0.02) | -0.62 (-1.11 to -0.14) | -0.37 (-0.78 to 0.04) | 2.05 (0.62 to 6.82) |
| 80 | -0.40 (-0.73 to -0.07) | -0.66 (-1.13 to -0.20) | -0.36 (-0.78 to 0.05) | 1.78 (0.72 to 4.40) |
| 100 | -0.46 (-0.75 to -0.16) | -0.65 (-1.07 to -0.22) | -0.31 (-0.69 to 0.06) | 1.51 (0.67 to 3.39) |
| 120 | -0.51 (-0.82 to -0.20) | -0.59 (-1.01 to -0.17) | -0.22 (-0.54 to 0.10) | 1.28 (0.46 to 3.56) |
| 140 | -0.55 (-0.95 to -0.16) | -0.50 (-1.01 to 0.00) | -0.11 (-0.40 to 0.19) | 1.09 (0.27 to 4.47) |
| 160 | -0.60 (-1.12 to -0.08) | -0.41 (-1.08 to 0.26) | 0.02 (-0.32 to 0.35) | 0.93 (0.14 to 6.01) |
| 180 | -0.65 (-1.31 to 0.00) | -0.32 (-1.19 to 0.54) | 0.14 (-0.29 to 0.57) | 0.79 (0.07 to 8.31) |
| 200 | -0.69 (-1.51 to 0.12) | -0.23 (-1.31 to 0.85) | 0.26 (-0.28 to 0.81) | 0.67 (0.04 to 11.6) |
| 220 | -0.74 (-1.71 to 0.24) | -0.14 (-1.43 to 1.16) | 0.39 (-0.29 to 1.07) | 0.57 (0.02 to 16.5) |
| P-nonlinearity | 0.63 | 0.11 | 0.08 | 0.36 |
| P-whole curve | 0.005 | 0.01 | 0.20 | 0.46 |

### Supplementary Table 19: Summary of the effects of whole grain consumption on glycemic control obtained from non-linear dose-response analysis

| Whole grain (g/d) | FPG (mmol/L) | Insulin (pmol/L) | HOMA-IR | HbA_1c_ (%) |
| --- | --- | --- | --- | --- |
| 0 | Ref | Ref | Ref | Ref |
| 20 | -0.01 (-0.06 to 0.04) | 0.02 (-1.14 to 1.19) | -0.08 (-0.19 to 0.03) | -0.04 (-0.11 to 0.04) |
| 40 | -0.02 (-0.11 to 0.06) | 0.04 (-1.91 to 1.98) | -0.15 (-0.34 to 0.04) | -0.07 (-0.20 to 0.07) |
| 60 | -0.03 (-0.12 to 0.06) | 0.04 (-2.23 to 2.31) | -0.20 (-0.43 to 0.03) | -0.08 (-0.24 to 0.08) |
| 80 | -0.04 (-0.11 to 0.03) | 0.03 (-2.24 to 2.30) | -0.23 (-0.46 to 0.01) | -0.08 (-0.22 to 0.06) |
| 100 | -0.05 (-0.11 to 0.01) | 0.02 (-2.11 to 2.15) | -0.24 (-0.45 to -0.02) | -0.07 (-0.18 to 0.04) |
| 120 | -0.06 (-0.13 to 0.01) | 0.00 (-2.12 to 2.12) | -0.24 (-0.44 to -0.03) | -0.06 (-0.13 to 0.02) |
| 140 | -0.07 (-0.17 to 0.03) | -0.02 (-2.53 to 2.48) | -0.23 (-0.46 to 0.01) | -0.04 (-0.10 to 0.02) |
| 160 | -0.08 (-0.23 to 0.07) | -0.05 (-3.32 to 3.22) | -0.21 (-0.51 to 0.08) | -0.03 (-0.11 to 0.06) |
| 180 | -0.09 (-0.29 to 0.11) | -0.07 (-4.32 to 4.17) | -0.20 (-0.59 to 0.18) | -0.01 (-0.14 to 0.11) |
| 200 | -0.10 (-0.35 to 0.16) | -0.10 (-5.41 to 5.20) | -0.19 (-0.67 to 0.29) | -0.00 (-0.17 to 0.17) |
| 220 | -0.11 (-0.41 to 0.20) | -0.13 (-6.54 to 6.29) | -0.18 (-0.76 to 0.40) | 0.01 (-0.20 to 0.23) |
| P-nonlinearity | 0.98 | 0.96 | 0.38 | 0.43 |
| P-whole curve | 0.15 | 0.99 | 0.08 | 0.31 |

Abbreviations: FPG: fasting plasma glucose- HbA_1c_: glycated hemoglobin, HOMA-IR: homeostatic model assessment for insulin resistance.

### Supplementary Table 20: Summary of the effects of whole grain consumption on blood lipids obtained from non-linear dose-response analysis

| Whole grain (g/d) | Total cholesterol (mmol/L) | HDL-C (mmol/L) | LDL-C (mmol/L) | Triglycerides (mmol/L) |
| --- | --- | --- | --- | --- |
| 0 | Ref | Ref | Ref | Ref |
| 20 | -0.10 (-0.14 to -0.06) | -0.02 (-0.04 to -0.00) | -0.08 (-0.12 to -0.04) | -0.04 (-0.07 to -0.01) |
| 40 | -0.17 (-0.23 to -0.10) | -0.03 (-0.06 to 0.00) | -0.14 (-0.20 to -0.07) | -0.07 (-0.12 to -0.02) |
| 60 | -0.20 (-0.27 to -0.13) | -0.02 (-0.05 to 0.01) | -0.16 (-0.23 to -0.09) | -0.08 (-0.13 to -0.03) |
| 80 | -0.21 (-0.27 to -0.15) | -0.00 (-0.03 to 0.03) | -0.16 (-0.22 to -0.10) | -0.08 (-0.13 to -0.04) |
| 100 | -0.20 (-0.26 to -0.13) | 0.03 (-0.01 to 0.06) | -0.15 (-0.21 to -0.09) | -0.07 (-0.11 to -0.03) |
| 120 | -0.18 (-0.26 to -0.10) | 0.05 ( 0.00 to 0.10) | -0.13 (-0.21 to -0.06) | -0.06 (-0.11 to -0.01) |
| 140 | -0.16 (-0.27 to -0.06) | 0.08 ( 0.01 to 0.15) | -0.12 (-0.22 to -0.01) | -0.05 (-0.11 to 0.01) |
| 160 | -0.15 (-0.29 to -0.01) | 0.11 ( 0.02 to 0.19) | -0.10 (-0.23 to 0.03) | -0.04 (-0.12 to 0.04) |
| 180 | -0.13 (-0.31 to 0.04) | 0.14 ( 0.03 to 0.24) | -0.08 (-0.25 to 0.08) | -0.03 (-0.13 to 0.07) |
| 200 | -0.12 (-0.33 to 0.10) | 0.16 ( 0.04 to 0.29) | -0.06 (-0.26 to 0.13) | -0.02 (-0.14 to 0.11) |
| 220 | -0.10 (-0.35 to 0.15) | 0.19 ( 0.05 to 0.34) | -0.05 (-0.28 to 0.19) | -0.01 (-0.15 to 0.14) |
| P-nonlinearity | 0.002 | 0.01 | 0.009 | 0.04 |
| P-whole curve | <0.001 | 0.03 | <0.001 | 0.002 |

Abbreviations: LDL-C: low-density lipoprotein cholesterol- HDL-C: high-density lipoprotein cholesterol

### Supplementary Table 21: Summary of the effects of whole grain consumption on blood pressure and inflammatory factors obtained from non-linear dose-response analysis

| Whole grain (g/d) | SBP (mm Hg) | DBP (mm Hg) | CRP (mg/L) | IL-6 (pg/mL) | TNF-α (pg/mL) |
| --- | --- | --- | --- | --- | --- |
| 0 | Ref | Ref | Ref | Ref | Ref |
| 20 | -1.26 (-2.26 to -0.25) | -0.81 (-1.65 to 0.02) | -0.30 (-0.47 to -0.13) | 0.00 (-0.16 to 0.17) | -0.02 (-0.09 to 0.04) |
| 40 | -2.12 (-3.77 to -0.46) | -1.29 (-2.57 to -0.00) | -0.50 (-0.78 to -0.23) | -0.01 (-0.29 to 0.27) | -0.04 (-0.14 t 0.07) |
| 60 | -2.44 (-4.29 to -0.58) | -1.42 (-2.78 to -0.06) | -0.58 (-0.86 to -0.30) | -0.04 (-0.35 to 0.28) | -0.04 (-0.15 to 0.08) |
| 80 | -2.35 (-4.09 to -0.62) | -1.31 (-2.54 to -0.08) | -0.50 (-0.73 to -0.27) | -0.09 (-0.37 to 0.20) | -0.03 (-0.14 to 0.08) |
| 100 | -2.01 (-3.53 to -0.49) | -1.03 (-2.15 to 0.10) | -0.31 (-0.63 to 0.01) | -0.14 (-0.37 to 0.09) | -0.02 (-0.13 to 0.08) |
| 120 | -1.54 (-2.97 to -0.12) | -0.66 (-1.96 to 0.63) | -0.04 (-0.62 to 0.54) | -0.20 (-0.41 to -0.00) | -0.01 (-0.12 to 0.09) |
| 140 | -1.07 (-2.67 to 0.54) | -0.28 (-2.00 to 1.44) | 0.28 (-0.63 to 1.19) | -0.27 (-0.50 to -0.03) | -0.00 (-0.12 to 0.11) |
| 160 | -0.59 (-2.57 to 1.40) | 0.10 (-2.17 to 2.36) | 0.61 (-0.65 to 1.88) | -0.33 (-0.63 to -0.02) | 0.01 (-0.13 to 0.14) |
| 180 | -0.11 (-2.58 to 2.37) | 0.48 (-2.39 to 3.34) | 0.95 (-0.68 to 2.57) | -0.39 (-0.79 to -0.00) | 0.01 (-0.14 to 0.17) |
| 200 | 0.37 (-2.65 to 3.40) | 0.85 (-2.63 to 4.34) | 1.28 (-0.71 to 3.27) | -0.46 (-0.95 to 0.00) | 0.02 (-0.16 to 0.20) |
| 220 | 0.85 (-2.75 to 4.46) | 1.23 (-2.89 to 5.35) | 1.62 (-0.74 to 3.97) | -0.51 (-1.12 to 0.09) | 0.03 (-0.17 to 0.24) |
| P-nonlinearity | 0.03 | 0.10 | 0.01 | 0.65 | 0.48 |
| P-whole curve | 0.03 | 0.11 | <0.001 | 0.07 | 0.78 |

Abbreviations: SBP: systolic blood pressure- DBP: diastolic blood pressure CRP: C reactive protein- IL-6: interleukin 6- TNF-α: tumor necrosis factor alpha

### Supplementary Table 22: Goodness-of-fit tests and measures for dose-response meta-analysis of whole grain consumption on adiposity measures and adverse events

| **Outcomes** | **Model** | **Deviance** | **P-value** | **R^2^** | **AIC** | **P-nonlinearity** | **P-whole curve** |
| --- | --- | --- | --- | --- | --- | --- | --- |
| Waist circumference | Linear | 46.1706 | 0.0167 | 0.20 | 93.98 | - | - |
|  | Quadratic | 43.1031 | 0.0255 | 0.25 | 124.20 | 0.09 | 0.01 |
|  | RCS (3 knots)^a^ | 43.2602 | 0.0246 | 0.25 | 111.36 | 0.11 | 0.01 |
|  | RCS (4 knots)^b^ | 43.0751 | 0.0190 | 0.26 | 122.74 | 0.26 | 0.03 |
| Body weight | Linear | 65.3224 | 0.0157 | 0.21 | 133.37 | - | - |
|  | Quadratic | 63.9296 | 0.0162 | 0.22 | 161.41 | 0.61 | 0.005 |
|  | RCS (3 knots)^a^ | 64.4484 | 0.0145 | 0.22 | 148.69 | 0.63 | 0.005 |
|  | RCS (4 knots)^b^ | 64.0429 | 0.0122 | 0.22 | 160.59 | 0.89 | 0.03 |
| Body fat percentage | Linear | 36.9752 | 0.0327 | 0.00 | 59.99 | - | - |
|  | Quadratic | 33.7568 | 0.0520 | 0.09 | 95.23 | 0.07 | 0.19 |
|  | RCS (3 knots)^a^ | 33.9598 | 0.0496 | 0.09 | 83.55 | 0.08 | 0.20 |
|  | RCS (4 knots)^b^ | 33.8981 | 0.0372 | 0.09 | 94.62 | 0.36 | 0.40 |
| Adverse events | Linear | 4.4282 | 0.6189 | 0.16 | 22.07 | - | - |
|  | Quadratic | 4.4281 | 0.4896 | 0.16 | 24.07 | 0.99 | 0.66 |
|  | RCS (3 knots)^a^ | 4.34 | 0.50 | 0.17 | 44.44 | 0.77 | 0.63 |
|  | RCS (4 knots)^b^ ^c^ | - | - | - | - | - | - |

^a^3 knots located at the 10th, 50th, and 90th percentiles of the distribution of whole grain

^b^ Due to the highly skewed distribution of the exposure variable and the large proportion of zero values, the default percentile-based knot selection (4th, 35th, 65th, 95th) resulted in non-unique knot locations and model convergence issues. Therefore, we specified knots manually at pre-defined, meaningful exposure levels to ensure appropriate model fitting and stability.

^c^Due to the small sample sizes and the limited number of studies, it was not possible to perform a cubic dose‑response analysis with four percentiles

### Supplementary Table 23: Goodness-of-fit tests and measures for dose-response meta-analysis of whole grain consumption on glycemic control

| **Outcomes** | **Model** | **Deviance** | **P-value** | **R^2^** | **AIC** | **P-nonlinearity** | **P-whole curve** |
| --- | --- | --- | --- | --- | --- | --- | --- |
| FPG | Linear | 132.991 | <0.001 | 0.09 | 46.44 | - | - |
|  | Quadratic | 127.722 | <0.001 | 0.13 | 66.61 | 0.94 | 0.28 |
|  | RCS (3 knots)^a^ | 129.130 | <0.001 | 0.12 | 53.54 | 0.98 | 0.15 |
|  | RCS (4 knots)^b^ | 126.699 | <0.001 | 0.14 | 65.65 | 0.73 | 0.58 |
| Insulin | Linear | 29.4409 | 0.4945 | 0.00 | 213.18 | - | - |
|  | Quadratic | 29.4181 | 0.4435 | 0.00 | 244.22 | 0.88 | 0.98 |
|  | RCS (3 knots)^a^ | 29.4390 | 0.4424 | 0.00 | 231.01 | 0.96 | 0.99 |
|  | RCS (4 knots)^b^ | 28.8963 | 0.4178 | 0.02 | 239.37 | 0.76 | 0.91 |
| HOMA-IR | Linear | 39.8447 | 0.0034 | 0.20 | 36.02 | - | - |
|  | Quadratic | 39.0606 | 0.0028 | 0.22 | 67.71 | 0.45 | 0.08 |
|  | RCS (3 knots)^a^ | 38.7100 | 0.0031 | 0.23 | 55.75 | 0.38 | 0.08 |
|  | RCS (4 knots)^b^ | 38.6518 | 0.0020 | 0.23 | 67.35 | 0.69 | 0.16 |
| HbA1c | Linear | 53.4148 | <0.001 | 0.08 | 7.50 | - | - |
|  | Quadratic | 53.3726 | <0.001 | 0.08 | 37.65 | 0.42 | 0.34 |
|  | RCS (3 knots)^a^ | 53.4086 | <0.001 | 0.08 | 26.77 | 0.43 | 0.31 |
|  | RCS (4 knots)^b^ | 52.2565 | <0.001 | 0.10 | 37.33 | 0.45 | 0.31 |

^a^3 knots located at the 10th, 50th, and 90th percentiles of the distribution of whole grain

^b^ Due to the highly skewed distribution of the exposure variable and the large proportion of zero values, the default percentile-based knot selection (4th, 35th, 65th, 95th) resulted in non-unique knot locations and model convergence issues. Therefore, we specified knots manually at pre-defined, meaningful exposure levels to ensure appropriate model fitting and stability.

### Supplementary Table 24: Goodness-of-fit tests and measures for dose-response meta-analysis of whole grain consumption on blood lipids

| **Outcomes** | **Model** | **Deviance** | **P-value** | **R^2^** | **AIC** | **P-nonlinearity** | **P-whole curve** |
| --- | --- | --- | --- | --- | --- | --- | --- |
| Total cholesterol | Linear | 147.4756 | <0.001 | 0.36 | 38.80 | - | - |
|  | Quadratic | 121.4762 | <0.001 | 0.47 | 39.49 | 0.004 | <0.001 |
|  | RCS (3 knots)^a^ | 120.6235 | <0.001 | 0.48 | 26.66 | 0.002 | <0.001 |
|  | RCS (4 knots)^b^ | 120.4013 | <0.001 | 0.48 | 41.03 | 0.005 | <0.001 |
| HDL-C | Linear | 305.3286 | <0.001 | 0.02 | 71.78 | - | - |
|  | Quadratic | 301.4881 | <0.001 | 0.03 | -55.72 | 0.05 | 0.09 |
|  | RCS (3 knots)^a^ | 290.4064 | <0.001 | 0.06 | -69.36 | 0.01 | 0.03 |
|  | RCS (4 knots)^b^ | 257.9879 | <0.001 | 0.17 | -59.70 | 0.01 | 0.02 |
| LDL-C | Linear | 136.2108 | <0.001 | 0.33 | 24.28 | - | - |
|  | Quadratic | 112.0 | <0.001 | 0.45 | 17.36 | <0.001 | <0.001 |
|  | RCS (3 knots)^a^ | 114.6 | <0.001 | 0.43 | 7.46 | 0.009 | <0.001 |
|  | RCS (4 knots)^b^ | 111.2 | <0.001 | 0.45 | 18.37 | 0.002 | <0.001 |
| Triglycerides | Linear | 101.7638 | 0.0002 | 0.15 | -22.25 | - | - |
|  | Quadratic | 91.86 | 0.001 | 0.23 | 10.57 | 0.01 | <0.001 |
|  | RCS (3 knots)^a^ | 94.17 | 0.001 | 0.22 | 0.21 | 0.04 | 0.002 |
|  | RCS (4 knots)^b^ | 90.57 | 0.001 | 0.25 | 12.10 | 0.05 | <0.001 |

^a^3 knots located at the 10th, 50th, and 90th percentiles of the distribution of whole grain

^b^ Due to the highly skewed distribution of the exposure variable and the large proportion of zero values, the default percentile-based knot selection (4th, 35th, 65th, 95th) resulted in non-unique knot locations and model convergence issues. Therefore, we specified knots manually at pre-defined, meaningful exposure levels to ensure appropriate model fitting and stability.

### Supplementary Table 25: Goodness-of-fit tests and measures for dose-response meta-analysis of whole grain consumption on blood pressure and inflammatory factors

| **Outcomes** | **Model** | **Deviance** | **P-value** | **R^2^** | **AIC** | **P-nonlinearity** | **P-whole curve** |
| --- | --- | --- | --- | --- | --- | --- | --- |
| SBP | Linear | 80.96 | <0.001 | 0.14 | 214.21 | - | - |
|  | Quadratic | 71.36 | 0.0001 | 0.24 | 220.32 | 0.02 | 0.02 |
|  | RCS (3 knots)^a^ | 72.32 | 0.0001 | 0.23 | 209.12 | 0.03 | 0.03 |
|  | RCS (4 knots)^b^ | 71.20 | 0.0001 | 0.24 | 216.53 | 0.08 | 0.06 |
| DBP | Linear | 108.70 | <0.001 | 0.10 | 205.97 | - | - |
|  | Quadratic | 102.97 | <0.001 | 0.14 | 193.06 | 0.10 | 0.11 |
|  | RCS (3 knots)^a^ | 104.95 | <0.001 | 0.13 | 180.03 | 0.10 | 0.11 |
|  | RCS (4 knots)^b^ | 102.12 | <0.001 | 0.15 | 190.35 | 0.47 | 0.41 |
| CRP | Linear | 107.38 | <0.001 | 0.13 | 121.31 | - | - |
|  | Quadratic | 87.36 | <0.001 | 0.29 | 100.57 | 0.02 | <0.001 |
|  | RCS (3 knots)^a^ | 79.87 | <0.001 | 0.36 | 87.61 | 0.02 | <0.001 |
|  | RCS (4 knots)^b^ | 48.13 | 0.0004 | 0.61 | 91.81 | <0.001 | <0.001 |
| IL-6 | Linear | 65.66 | <0.001 | 0.82 | 74.23 | - | - |
|  | Quadratic | 44.41 | <0.001 | 0.88 | 70.21 | 0.66 | 0.001 |
|  | RCS (3 knots)^a^ | 26.1292 | 0.006 | 0.93 | 59.09 | 0.65 | 0.07 |
|  | RCS (4 knots)^b^ | 19.3830 | 0.03 | 0.95 | 70.36 | 0.10 | 0.001 |
| TNF-α | Linear | 2.99 | 0.80 | 0.00 | 4.38 | - | - |
|  | Quadratic | 2.30 | 0.80 | 0.23 | 46.91 | 0.40 | 0.70 |
|  | RCS (3 knots)^a^ | 2.50 | 0.77 | 0.16 | 34.72 | 0.48 | 0.78 |
|  | RCS (4 knots)^b^ | 2.18 | 0.70 | 0.27 | 48.44 | 0.84 | 0.66 |

^a^3 knots located at the 10th, 50th, and 90th percentiles of the distribution of whole grain

^b^ Due to the highly skewed distribution of the exposure variable and the large proportion of zero values, the default percentile-based knot selection (4th, 35th, 65th, 95th) resulted in non-unique knot locations and model convergence issues. Therefore, we specified knots manually at pre-defined, meaningful exposure levels to ensure appropriate model fitting and stability.

### Supplementary Table 26: GRADE assessment for primary and secondary outcomes

| **Certainty assessment** | | | | | | | **№ of patients** | | **Effect** | | **Certainty** | **Importance** |
| --- | --- | --- | --- | --- | --- | --- | --- | --- | --- | --- | --- | --- |
| **№ of studies** | **Study design** | **Risk of bias** | **Inconsistency** | **Indirectness** | **Imprecision** | **Other considerations** | **[intervention]** | **[comparison]** | **Relative (95% CI)** | **Absolute (95% CI)** |  |  |
| **Low-density lipoprotein cholesterol** | | | | | | | | | | | | |
| 69 | randomised trials | serious^a^ | not serious^b^ | not serious | serious^c^ | dose response gradient^d^ | 3151 | 2781 | - | MD **0.13 mmol/L lower** (0.17 lower to 0.08 lower) | ⨁⨁⨁◯ Moderate^a,b,c,d^ | IMPORTANT |
| **Weight** | | | | | | | | | | | | |
| 60 | randomised trials | serious^e^ | not serious^f^ | not serious | not serious^g^ | dose response gradient^h^ | 2264 | 2125 | - | MD **0.51 Kg lower** (0.72 lower to 0.3 lower) | ⨁⨁⨁⨁ High^e,f,g,h^ | IMPORTANT |
| **Fasting plasma glucose** | | | | | | | | | | | | |
| 62 | randomised trials | serious^i^ | not serious^j^ | not serious | not serious^k^ | none | 2530 | 2198 | - | MD **0.07 mmol/L lower** (0.13 lower to 0.02 lower) | ⨁⨁⨁◯ Moderate^i,j,k^ | IMPORTANT |
| **Adverse events** | | | | | | | | | | | | |
| 7 | randomised trials | serious^l^ | not serious^m^ | not serious | serious^n^ | none | 20/406 (4.9%) | 9/336 (2.7%) | **RR 1.53** (0.72 to 3.22) | **2 more per 100** (from 1 fewer to 5 more) | ⨁⨁◯◯ Low^l,m,n^ | IMPORTANT |
| **Waist circumference** | | | | | | | | | | | | |
| 38 | randomised trials | serious^o^ | not serious^p^ | not serious | not serious^q^ | dose response gradient^r^ | 1666 | 1541 | - | MD **0.6 cm lower** (0.98 lower to 0.22 lower) | ⨁⨁⨁⨁ High^o,p,q,r^ | IMPORTANT |
| **Body fat percentage** | | | | | | | | | | | | |
| 29 | randomised trials | serious^s^ | not serious^t^ | not serious | not serious^u^ | none | 1448 | 1234 | - | MD **0.22 % lower** (0.47 lower to 0.02 higher) | ⨁⨁⨁◯ Moderate^s,t,u^ | IMPORTANT |
| **Triglycerides** | | | | | | | | | | | | |
| 70 | randomised trials | serious^v^ | not serious^w^ | not serious | serious^x^ | dose response gradient^y^ | 3058 | 2687 | - | MD **0.05 mmol/L lower** (0.09 lower to 0.02 lower) | ⨁⨁⨁◯ Moderate^v,w,x,y^ | IMPORTANT |
| **Total cholesterol** | | | | | | | | | | | | |
| 73 | randomised trials | serious^z^ | not serious^aa^ | not serious | not serious^ab^ | dose response gradient^ac^ | 3147 | 2760 | - | MD **0.17 mmol/L lower** (0.22 lower to 0.12 lower) | ⨁⨁⨁⨁ High^aa,ab,ac,z^ | IMPORTANT |
| **High-density lipoprotein cholesterol** | | | | | | | | | | | | |
| 71 | randomised trials | serious^ad^ | serious^ae^ | not serious | not serious^af^ | dose response gradient^ag^ | 3064 | 2690 | - | MD **0.01 mmol/L higher** (0.01 lower to 0.03 higher) | ⨁⨁⨁◯ Moderate^ad,ae,af,ag^ | IMPORTANT |
| **Insulin** | | | | | | | | | | | | |
| 42 | randomised trials | serious^ah^ | not serious^ai^ | not serious | not serious^aj^ | none | 1666 | 1477 | - | MD **0.37 pmol/L lower** (2.19 lower to 1.46 higher) | ⨁⨁⨁◯ Moderate^ah,ai,aj^ | IMPORTANT |
| **Homeostatic model assessment for insulin resistance** | | | | | | | | | | | | |
| 30 | randomised trials | serious^ak^ | serious^al^ | not serious | not serious^am^ | none | 1329 | 1157 | - | MD **0.24 lower** (0.39 lower to 0.09 lower) | ⨁⨁◯◯ Low^ak,al,am^ | IMPORTANT |
| **Glycated hemoglobin** | | | | | | | | | | | | |
| 24 | randomised trials | serious^an^ | not serious^ao^ | not serious | not serious^ap^ | none | 985 | 923 | - | MD **0.05 % lower** (0.1 lower to 0.01 lower) | ⨁⨁⨁◯ Moderate^an,ao,ap^ | IMPORTANT |
| **Systolic blood pressure** | | | | | | | | | | | | |
| 46 | randomised trials | serious^aq^ | not serious^ar^ | not serious | serious^as^ | dose response gradient^at^ | 1889 | 1720 | - | MD **1.5 mmHg lower** (2.62 lower to 0.38 lower) | ⨁⨁⨁◯ Moderate^aq,ar,as,at^ | IMPORTANT |
| **Diastolic blood pressure** | | | | | | | | | | | | |
| 45 | randomised trials | serious^au^ | not serious^av^ | not serious | not serious^aw^ | none | 1833 | 1689 | - | MD **0.82 mmHg lower** (1.59 lower to 0.05 lower) | ⨁⨁⨁◯ Moderate^au,av,aw^ | IMPORTANT |
| **C-reactive protein** | | | | | | | | | | | | |
| 31 | randomised trials | serious^ax^ | serious^ay^ | not serious | serious^az^ | dose response gradient^ba^ | 1361 | 1148 | - | MD **0.38 mg/L lower** (0.55 lower to 0.21 lower) | ⨁⨁◯◯ Low^ax,ay,az,ba^ | IMPORTANT |
| **Interleukin 6** | | | | | | | | | | | | |
| 20 | randomised trials | serious^bb^ | not serious^bc^ | not serious | not serious^bd^ | dose response gradient^be^ | 655 | 564 | - | MD **0.22 pg/mL lower** (0.44 lower to 0.01 lower) | ⨁⨁⨁⨁ High^bb,bc,bd,be^ | IMPORTANT |
| **Tumor necrosis factor-alpha** | | | | | | | | | | | | |
| 14 | randomised trials | serious^bf^ | not serious^bg^ | not serious | serious^bh^ | none | 354 | 343 | - | MD **0.04 pg/mL lower** (0.16 lower to 0.08 higher) | ⨁⨁◯◯ Low^bf,bg,bh^ | IMPORTANT |
| **Health-related quality of life** | | | | | | | | | | | | |
| 1 | randomised trials | serious^bi^ | not serious | serious^bj^ | serious^bk^ | none | 23 | 16 | - | MD **0.9 lower** (11.7 lower to 9.93 higher) | ⨁◯◯◯ Very low^bi,bj,bk^ | IMPORTANT |

**Abbreviations:** GRADE: grading of recommendations assessment, development, and evaluation; CI: confidence interval; MD: mean difference; RR: risk ratio; SMD: standardized mean difference

**Explanations**

a. Serious risk of bias since most RCTs were rated to have high risk of bias. Downgraded.

b. Statistically significant heterogeneity (I^2^ = 57.9%) explained by one outlier study (Geng et al. 2016, I^2^ = 45.2%). Not downgraded.

c. Serious imprecision since the 95% CI overlap with the minimally important difference for clinical benefit. Downgraded.

d. There was evidence of dose-response gradient based on non-linear analysis (P-whole curve<0.001). Upgraded.

e. Serious risk of bias since most RCTs were rated to have high risk of bias. Downgraded.

f. Not serious inconsistency (I^2^ = 40.4%). Not downgraded.

g. Not serious imprecision since the 95% CI does not overlap with the minimally important difference. Not downgraded.

h. There was evidence of dose-response gradient based on non-linear analysis (P-whole curve=0.002). Upgraded.

i. Serious risk of bias since most RCTs were rated to have high risk of bias. Downgraded.

j. Statistically significant heterogeneity (I^2^ = 69.3%) explained by two outlier studies (Hsu et al. 2008 and Ren et al. 2020, I^2^ = 47.2%). Not downgraded.

k. Not serious imprecision since the 95% CI does not overlap with the minimally important difference. Not downgraded.

l. Serious risk of bias since most RCTs were rated to have high risk of bias. Downgraded.

m. Not serious inconsistency (I^2^ = 0%). Not downgraded.

n. Serious imprecision since the number of events was lower than the optimal information size (n=29). Downgraded.

o. Serious risk of bias since most RCTs were rated to have high risk of bias. Downgraded.

p. Statistically significant heterogeneity (I^2^ = 54.8%) explained by one outlier study (Wang et al. 2013, I^2^ = 37.2%). Not downgraded.

q. Not serious imprecision since the 95% CI does not overlap with the minimally important difference. Not downgraded.

r. There was evidence of dose-response gradient based on non-linear analysis (P-whole curve=0.007). Upgraded.

s. Serious risk of bias since most RCTs were rated to have high risk of bias. Downgraded.

t. Not serious inconsistency (I^2^ = 41.1%). Not downgraded.

u. Not serious imprecision since the 95% CI does not overlap with the minimally important difference. Not downgraded.

v. Serious risk of bias since most RCTs were rated to have high risk of bias. Downgraded.

w. Not serious inconsistency (I^2^ = 48.2%). Not downgraded.

x. Serious imprecision since the 95% CI overlap with the minimally important difference for clinical benefit. Downgraded.

y. There was evidence of dose-response gradient based on non-linear analysis (P-whole curve<0.001). Upgraded.

z. Serious risk of bias since most RCTs were rated to have high risk of bias. Downgraded.

aa. Statistically significant heterogeneity (I^2^ = 71.5%) explained by health status; trials with metabolically healthy participants showed smaller effect size (P for subgroup difference was not significant) with I^2^ = 39.4%. Not downgraded.

ab. Not serious imprecision since the 95% CI does not overlap with the minimally important difference. Not downgraded.

ac. There was evidence of dose-response gradient based on non-linear analysis (P-whole curve<0.001). Upgraded.

ad. Serious risk of bias since most RCTs were rated to have high risk of bias. Downgraded.

ae. Serious inconsistency (I^2^ = 81.9%,) that remained unexplained in the pre-specified subgroup and sensitivity analyses. Downgraded.

af. Not serious imprecision since the 95% CI does not overlap with the minimally important difference. Not downgraded.

ag. There was evidence of dose-response gradient based on non-linear analysis (P-whole curve=0.04). Upgraded.

ah. Serious risk of bias since most RCTs were rated to have high risk of bias. Downgraded.

ai. Not serious inconsistency (I^2^ = 12.8%). Not downgraded.

aj. Not serious imprecision since the 95% CI does not overlap with the minimally important difference. Not downgraded.

ak. Serious risk of bias since most RCTs were rated to have high risk of bias. Downgraded.

al. Serious inconsistency (I^2^ = 66.4%,) that remained unexplained in the pre-specified subgroup and sensitivity analyses. Downgraded.

am. Not serious imprecision since the 95% CI does not overlap with the minimally important difference. Not downgraded.

an. Serious risk of bias since most RCTs were rated to have high risk of bias. Downgraded.

ao. Statistically significant heterogeneity (I^2^ = 58.1%) explained by two outlier study (Nazari et al. 2021 and Na et al. 2023, I^2^ = 46.3%). Not downgraded.

ap. Not serious imprecision since the 95% CI does not overlap with the minimally important difference. Not downgraded.

aq. Serious risk of bias since most RCTs were rated to have high risk of bias. Downgraded.

ar. Statistically significant heterogeneity (I^2^ = 62.5%) explained by two outlier studies (Liao et al. 2018 and Geng et al. 2016, I^2^ = 44.2%). Not downgraded.

as. Serious imprecision since the 95% CI overlap with the minimally important difference for clinical benefit. Downgraded.

at. There was evidence of dose-response gradient based on non-linear analysis (P- whole curve=0.02). Upgraded.

au. Serious risk of bias since most RCTs were rated to have high risk of bias. Downgraded.

av. Statistically significant heterogeneity (I^2^ = 65.4%) explained by two outlier studies (Hsu et al. 2008 and Geng et al. 2016, I^2^ = 26.8%). Not downgraded.

aw. Not serious imprecision since the 95% CI does not overlap with the minimally important difference. Not downgraded.

ax. Serious risk of bias since most RCTs were rated to have high risk of bias. Downgraded.

ay. Serious inconsistency (I^2^ = 83.1%,) that remained unexplained in the pre-specified subgroup and sensitivity analyses. Downgraded.

az. Serious imprecision since the 95% CI overlap with the minimally important difference for clinical benefit. Downgraded.

ba. There was evidence of dose-response gradient based on non-linear analysis (P-whole curve<0.001). Upgraded.

bb. Serious risk of bias since most RCTs were rated to have high risk of bias. Downgraded.

bc. Statistically significant heterogeneity (I^2^ = 67.3%) explained by one outlier study (Pavadhgul et al. 2019, I^2^ = 45.7%). Not downgraded.

bd. Not serious imprecision since the 95% CI does not overlap with the minimally important difference. Not downgraded.

be. There was evidence of dose-response gradient based on non-linear analysis (P-whole curve=0.07). Upgraded.

bf. Serious risk of bias since most RCTs were rated to have high risk of bias. Downgraded.

bg. Statistically significant heterogeneity (I^2^ = 64.4%) explained by two outlier study (Pavadhgul et al. 2019 and Vitaglione et al. 2014, I^2^ = 23.2%). Not downgraded.

bh. Serious imprecision since optimal information size was not met (n= 697 participants). Downgraded.

bi. Serious risk of bias since the included RCT was rated to has high risk of bias. Downgraded.

bj. Serious indirectness since only one study was included. Downgraded.

bk. Serious imprecision since optimal information size was not met (n= 39 participants). Downgraded.

Records identified through database searching
(n=82,881)

Screening

Included

Eligibility

Identification

Records after duplicates removed
(n=63,674)

Records screened
(n=63,674)

Records excluded
(n=63,463)

Full-text articles assessed for eligibility
(n=211)

Full-text articles excluded

- Duplicate publication (n=17)
- Not relevant outcomes (n=11)
- Not relevant intervention (n=2)
- Not sufficient data (n=1)
- In critically ill patients (n=3)
- Acute feeding trials (n=27)
- Non-randomized trials (n=2)
- Active control (n=6)
- Shorter than 2 weeks (n=1)
- Lack of washout period (n=7)
- Comparison of two types of whole grains (n=15)
- Comparison of new and old wheat varieties (n=5)
- Semi-whole grain consumption in the control group (n=2)
- Multicomponent intervention (n=11)

Publications included in qualitative synthesis
(n=101)

Publications included in quantitative synthesis (meta-analysis)
(n=101)

### Supplementary Figure 1: Flow diagram of study selection

Supplementary Figure 2: Forest plot for the effect of whole grain consumption on LDL-C concentrations (mmol/L) in adults aged ≥18 years, expressed as mean differences between the intervention and control groups. LDL-C, low-density lipoprotein cholesterol; WMD, weighted mean difference; CI: confidence interval.

Supplementary Figure 3: Forest plot for the effect of whole grain consumption on body weight (kg) in adults aged ≥18 years, expressed as mean differences between the intervention and control groups. WMD, weighted mean difference; CI: confidence interval.

Supplementary Figure 4: Forest plot for the effect of whole grain consumption on FPG concentrations (mmol/L) in adults aged ≥18 years, expressed as mean differences between the intervention and control groups. FPG, fasting plasma glucose; WMD, weighted mean difference; CI: confidence interval.

Supplementary Figure 5: Forest plot for the effect of whole grain consumption on adverse events in adults aged ≥18 years, expressed as risk ratio. CI: confidence interval.

Supplementary Figure 6: Forest plot for the effect of whole grain consumption on adverse events in adults aged ≥18 years, expressed as risk difference. CI: confidence interval.

Supplementary Figure 7: Forest plot for the effect of whole grain consumption on waist circumference (cm) in adults aged ≥18 years, expressed as mean differences between the intervention and control groups. WMD, weighted mean difference; CI: confidence interval.

Supplementary Figure 8: Forest plot for the effect of whole grain consumption on body fat percentage in adults aged ≥18 years, expressed as mean differences between the intervention and control groups. WMD, weighted mean difference; CI: confidence interval.

Supplementary Figure 9: Forest plot for the effect of whole grain consumption on insulin concentrations (pmol/L) in adults aged ≥18 years, expressed as mean differences between the intervention and control groups. WMD, weighted mean difference; CI: confidence interval.

Supplementary Figure 10: Forest plot for the effect of whole grain consumption on HOMA-IR in adults aged ≥18 years, expressed as mean differences between the intervention and control groups. HOMA-IR, homeostasis model assessment of insulin resistance; WMD, weighted mean difference; CI: confidence interval.

Supplementary Figure 11: Forest plot for the effect of whole grain consumption on HbA1c (%) in adults aged ≥18 years, expressed as mean differences between the intervention and control groups. HbA_1c_, glycated hemoglobin; WMD, weighted mean difference; CI: confidence interval.

Supplementary Figure 12: Forest plot for the effect of whole grain consumption on triglycerides concentrations (mmol/L) in adults aged ≥18 years, expressed as mean differences between the intervention and control groups. WMD, weighted mean difference; CI: confidence interval.

Supplementary Figure 13: Forest plot for the effect of whole grain consumption on total cholesterol concentrations (mmol/L) in adults aged ≥18 years, expressed as mean differences between the intervention and control groups. WMD, weighted mean difference; CI: confidence interval.

Supplementary Figure 14: Forest plot for the effect of whole grain consumption on HDL-C concentrations (mmol/L) in adults aged ≥18 years, expressed as mean differences between the intervention and control groups. HDL-C, high-density lipoprotein cholesterol; WMD, weighted mean difference; CI: confidence interval.

Supplementary Figure 15: Forest plot for the effect of whole grain consumption on SBP (mmHg) in adults aged ≥18 years, expressed as mean differences between the intervention and control groups. SBP, Systolic blood pressure; WMD, weighted mean difference; CI: confidence interval.

Supplementary Figure 16: Forest plot for the effect of whole grain consumption on DBP (mmHg) in adults aged ≥18 years, expressed as mean differences between the intervention and control groups. DBP, Diastolic blood pressure; WMD, weighted mean difference; CI: confidence interval.

Supplementary Figure 17: Forest plot for the effect of whole grain consumption on CRP (mg/L) in adults aged ≥18 years, expressed as mean differences between the intervention and control groups. CRP, C-reactive protein; WMD, weighted mean difference; CI: confidence interval.

Supplementary Figure 18: Forest plot for the effect of whole grain consumption on IL-6 (pg/mL) in adults aged ≥18 years, expressed as mean differences between the intervention and control groups. IL-6, interleukin 6; WMD, weighted mean difference; CI: confidence interval.

Supplementary Figure 19: Forest plot for the effect of whole grain consumption on TNF-α (pg/mL) in adults aged ≥18 years, expressed as mean differences between the intervention and control groups. TNF-α, tumor necrosis factor alpha; WMD, weighted mean difference; CI: confidence interval.

Supplementary Figure 20: Forest plot for the effect of whole grain consumption on HRQoL in adults aged ≥18 years, expressed as mean differences between the intervention and control groups. HRQoL, health-related quality of life; WMD, weighted mean difference; CI: confidence interval.

Supplementary Figure 21: Contour-enhanced Funnel plot for LDL-C. LDL-C, low-density lipoprotein cholesterol.

### Supplementary Figure 22: Contour-enhanced Funnel plot for body weight

Supplementary Figure 23: Contour-enhanced Funnel plot for FPG**.** FPG, fasting plasma glucose.

### Supplementary Figure 24: Contour-enhanced Funnel plot for adverse events

### Supplementary Figure 25: Contour-enhanced Funnel plot for waist circumference

### Supplementary Figure 26: Contour-enhanced Funnel plot for body fat percentage

### Supplementary Figure 27: Contour-enhanced Funnel plot for insulin

Supplementary Figure 28: Contour-enhanced Funnel plot for HOMA-IR. HOMA-IR, homeostatic model assessment for insulin resistance.

Supplementary Figure 29: Contour-enhanced Funnel plot for HbA1c**.** HbA_1c_, glycated hemoglobin.

### Supplementary Figure 30: Contour-enhanced Funnel plot for triglycerides

### Supplementary Figure 31: Contour-enhanced Funnel plot for total cholesterol

Supplementary Figure 32: Contour-enhanced Funnel plot for HDL-C. HDL-c, high density lipoprotein cholesterol.

Supplementary Figure 33: Contour-enhanced Funnel plot for SBP**.** SBP, systolic blood pressure.

Supplementary Figure 34: Contour-enhanced Funnel plot for DBP**.** DBP, diastolic blood pressure.

Supplementary Figure 35: Contour-enhanced Funnel plot for C-reactive protein. CRP, C-reactive protein.

Supplementary Figure 36: Contour-enhanced Funnel plot for IL-6. IL-6, interleukin 6.

Supplementary Figure 37: Contour-enhanced Funnel plot for TNF-α. TNF-α, tumor necrosis factor alpha.

Supplementary Figure 38: Sensitivity analysis for the effect of whole grain consumption on LDL-C (mmol/L). LDL-C, low-density lipoprotein cholesterol concentrations; WMD, weighted mean difference; CI: confidence interval.

Supplementary Figure 39: Sensitivity analysis for the effect of whole grain consumption on body weight (kg). WMD, weighted mean difference; CI: confidence interval.

Supplementary Figure 40: Sensitivity analysis for the effect of whole grain consumption on FPG concentrations (mmol/L). FPG, fasting plasma glucose; WMD, weighted mean difference; CI: confidence interval.

Supplementary Figure 41: Sensitivity analysis for the effect of whole grain consumption on adverse events. CI: confidence interval.

Supplementary Figure 42: Sensitivity analysis for the effect of whole grain consumption on waist circumference (cm). WMD, weighted mean difference; CI: confidence interval.

Supplementary Figure 43: Sensitivity analysis for the effect of whole grain consumption on body fat percentage. WMD, weighted mean difference; CI: confidence interval.

Supplementary Figure 44: Sensitivity analysis for the effect of whole grain consumption on insulin concentrations (pmol/L). WMD, weighted mean difference; CI: confidence interval.

Supplementary Figure 45: Sensitivity analysis for the effect of whole grain consumption on HOMA-IR. HOMA-IR, homeostatic model assessment for insulin resistance; WMD, weighted mean difference; CI: confidence interval.

Supplementary Figure 46: Sensitivity analysis for the effect of whole grain consumption on HbA1_c_ (%). HbA_1c_, glycated hemoglobin; WMD, weighted mean difference; CI: confidence interval.

Supplementary Figure 47: Sensitivity analysis for the effect of whole grain consumption on triglycerides concentrations (mmol/L). WMD, weighted mean difference; CI: confidence interval.

Supplementary Figure 48: Sensitivity analysis for the effect of whole grain consumption on total cholesterol concentrations (mmol/L). WMD, weighted mean difference; CI: confidence interval.

Supplementary Figure 49: Sensitivity analysis for the effect of whole grain consumption on HDL-C concentrations (mmol/L). HDL-c, high density lipoprotein cholesterol; WMD, weighted mean difference; CI: confidence interval.

Supplementary Figure 50: Sensitivity analysis for the effect of whole grain consumption on SBP (mmHg). SBP, systolic blood pressure; WMD, weighted mean difference; CI: confidence interval.

Supplementary Figure 51: Sensitivity analysis for the effect of whole grain consumption on DBP (mmHg). DBP, diastolic blood pressure; WMD, weighted mean difference; CI: confidence interval.

Supplementary Figure 52: Sensitivity analysis for the effect of whole grain consumption on CRP (mg/L). CRP, C-reactive protein; WMD, weighted mean difference; CI: confidence interval.

Supplementary Figure 53: Sensitivity analysis for the effect of whole grain consumption on IL-6 (pg/mL). IL-6, interleukin 6; WMD, weighted mean difference; CI: confidence interval.

Supplementary Figure 54: Sensitivity analysis for the effect of whole grain consumption on TNF-α (pg/mL). TNF-α, tumor necrosis factor alpha; WMD, weighted mean difference; CI: confidence interval.

Supplementary Figure 55: Forest plot for the LDL-C (mmol/L) based on 48 g increase in whole grain consumption in adults aged ≥18 years. LDL-C, low-density lipoprotein cholesterol; WMD, weighted mean difference; CI: confidence interval.

Supplementary Figure 56: Forest plot for the body weight (kg) based on 48 g increase in whole grain consumption in adults aged ≥18 years**.** WMD, weighted mean difference; CI: confidence interval.

Supplementary Figure 57: Forest plot for the FPG (mmol/L) based on 48 g increase in whole grain consumption in adults aged ≥18 years. FPG, fasting plasma glucose; WMD, weighted mean difference; CI: confidence interval.

Supplementary Figure 58: Forest plot for the adverse events based on 48 g increase in whole grain consumption in adults aged ≥18 years. CI: confidence interval.

Supplementary Figure 59: Forest plot for the waist circumference (cm) based on 48 g increase in whole grain consumption in adults aged ≥18 years. WMD, weighted mean difference; CI: confidence interval.

Supplementary Figure 60: Forest plot for the body fat percentage based on 48 g increase in whole grain consumption in adults aged ≥18 years. WMD, weighted mean difference; CI: confidence interval.

Supplementary Figure 61: Forest plot for the insulin (pmol/L) based on 48 g increase in whole grain consumption in adults aged ≥18 years. WMD, weighted mean difference; CI: confidence interval.

Supplementary Figure 62: Forest plot for the HOMA-IR based on 48 g increase in whole grain consumption in adults aged ≥18 years. HOMA-IR, homeostatic model assessment for insulin resistance; WMD, weighted mean difference; CI, confidence interval.

Supplementary Figure 63: Forest plot for the HbA1c (%) based on 48 g increase in whole grain consumption in adults aged ≥18 years. HbA_1c_, glycated hemoglobin; WMD, weighted mean difference; CI, confidence interval.

Supplementary Figure 64: Forest plot for the triglycerides (mmol/L) based on 48 g increase in whole grain consumption in adults aged ≥18 years. TG, triglycerides; WMD, weighted mean difference; CI: confidence interval.

Supplementary Figure 65: Forest plot for the total cholesterol (mmol/L) based on 48 g increase in whole grain consumption in adults aged ≥18 years. WMD, weighted mean difference; CI: confidence interval.

Supplementary Figure 66: Forest plot for the HDL-C (mmol/L) based on 48 g increase in whole grain consumption in adults aged ≥18 years. HDL-c, high density lipoprotein cholesterol; WMD, weighted mean difference; CI: confidence interval.

Supplementary Figure 67: Forest plot for the SBP (mmHg) based on 48 g increase in whole grain consumption in adults aged ≥18 years. SBP, systolic blood pressure; WMD, weighted mean difference; CI, confidence interval.

Supplementary Figure 68: Forest plot for the DBP (mmHg) based on 48 g increase in whole grain consumption in adults aged ≥18 years. DBP, diastolic blood pressure; WMD, weighted mean difference; CI, confidence interval.

Supplementary Figure 69: Forest plot for the CRP (mg/L) based on 48 g increase in whole grain consumption in adults aged ≥18 years. CRP, C-reactive protein; WMD, weighted mean difference; CI, confidence interval.

Supplementary Figure 70: Forest plot for the IL-6 (pg/mL) based on 48 g increase in whole grain consumption in adults aged ≥18 years. IL-6, interleukin 6; WMD, weighted mean difference; CI, confidence interval.

Supplementary Figure 71: Forest plot for the TNF-α (pg/mL) based on 48 g increase in whole grain consumption in adults aged ≥18 years. TNF-α, tumor necrosis factor alpha; WMD, weighted mean difference; CI, confidence interval.

### **Supplementary Figure 72:** Dose‑response effects of whole grain consumption using the quadratic model on weight, waist circumference, body fat percentage, and adverse events in adults aged ≥18 years

Supplementary Figure 73: Dose‑response effects of whole grain consumption using the quadratic model on FPG, HbA1c, insulin, and HOMA-IR in adults aged ≥18 years. FPG: fasting plasma glucose, HbA1c: glycated haemoglobin, HOMA-IR: homeostasis model assessment of insulin resistance

Supplementary Figure 74: Dose‑response effects of whole grain consumption using the quadratic model triglycerides, total cholesterol, LDL-C, and HDL-C in adults aged ≥18 years. LDL-C: low-density lipoprotein cholesterol, HDL-C: high-density lipoprotein cholesterol.

Supplementary Figure 75: Dose‑response effects of whole grain consumption using the quadratic model on SBP and DBP in adults aged ≥18 years. SBP: systolic blood pressure, DBP: diastolic blood pressure.

Supplementary Figure 76: Dose‑response effects of whole grain consumption using the quadratic model on CRP, IL-6, and TNF-α in adults aged ≥18 years. CRP: C-reactive protein, IL-6: interleukin 6, TNF-α: tumor necrosis factor alpha.

Supplementary Figure 77: Dose‑response effects of whole grain consumption using the RCS (with four knots) model on weight, waist circumference, and body fat percentage in adults aged ≥18 years. RCS: restricted cubic spline

Supplementary Figure 78: Dose‑response effects of whole grain consumption using the RCS (with four knots) model on FPG, HbA1c, insulin, and HOMA-IR in adults aged ≥18 years. RCS: restricted cubic spline, FPG: fasting plasma glucose, HbA_1c_: glycated haemoglobin, HOMA-IR: homeostasis model assessment of insulin resistance.

Supplementary Figure 79: Dose‑response effects of whole grain consumption using the RCS (with four knots) model on triglycerides, total cholesterol, LDL-C, and HDL-C in adults aged ≥18 years. RCS: restricted cubic spline, LDL-C: low-density lipoprotein cholesterol, HDL-C: high-density lipoprotein cholesterol.

Supplementary Figure 80: Dose‑response effects of whole grain consumption using the RCS (with four knots) model on SBP and DBP in adults aged ≥18 years. RCS: restricted cubic spline, SBP: systolic blood pressure, DBP: diastolic blood pressure.

Supplementary Figure 81: Dose‑response effects of whole grain consumption using the RCS (with four knots) model on CRP, IL-6, and TNF-α in adults aged ≥18 years. RCS: restricted cubic spline, CRP: C-reactive protein, IL-6: interleukin 6, TNF-α: tumor necrosis factor alpha.

Supplementary Figure 82: Dose‑response effects of whole grain consumption on weight, waist circumference, and body fat percentage in adults aged ≥18 years using linear, RCS, and quadratic models in the same graph**.** RCS: restricted cubic spline

Supplementary Figure 83: Dose‑response effects of whole grain consumption on FPG, HbA1c, insulin, and HOMA-IR in adults aged ≥18 years using linear, RCS, and quadratic models in the same graph. RCS: restricted cubic spline, FPG: fasting plasma glucose, HbA_1c_: glycated haemoglobin, HOMA-IR: homeostasis model assessment of insulin resistance.

Supplementary Figure 84: Dose‑response effects of whole grain consumption on triglycerides, total cholesterol, LDL-C, and HDL-C in adults aged ≥18 years using linear, RCS, and quadratic models in the same graph. RCS: restricted cubic spline, LDL-C: low-density lipoprotein cholesterol, HDL-C: high-density lipoprotein cholesterol.

Supplementary Figure 85: Dose‑response effects of whole grain consumption on SBP and DBP in adults aged ≥18 years using linear, RCS, and quadratic models in the same graph. RCS: restricted cubic spline, SBP: systolic blood pressure, DBP: diastolic blood pressure.

Supplementary Figure 86: Dose‑response effects of whole grain consumption on CRP, IL-6, and TNF-α in adults aged ≥18 years using linear, RCS, and quadratic models in the same graph. RCS: restricted cubic spline, CRP: C-reactive protein, IL-6: interleukin 6, TNF-α: tumor necrosis factor alpha.

### **Supplementary Figure 87:** Decorrelated residuals versus dose for the quadratic model of whole grain intake on weight, waist circumference, body fat percentage, and adverse events.

Supplementary Figure 88: Decorrelated residuals versus dose for the quadratic model of whole grain intake on FPG, HbA1c, insulin, and HOMA-IR. FPG: fasting plasma glucose, HbA_1c_: glycated haemoglobin, HOMA-IR: homeostasis model assessment of insulin resistance.

Supplementary Figure 89: Decorrelated residuals versus dose for the quadratic model of whole grain intake on triglycerides, total cholesterol, LDL-C, and HDL-C. LDL-C: low-density lipoprotein cholesterol, HDL-C: high-density lipoprotein cholesterol.

Supplementary Figure 90: Decorrelated residuals versus dose for the quadratic model of whole grain intake on SBP and DBP. SBP: systolic blood pressure, DBP: diastolic blood pressure.

Supplementary Figure 91: Decorrelated residuals versus dose for the quadratic model of whole grain intake on CRP, IL-6, and TNF-α. CRP: C-reactive protein, IL-6: interleukin 6, TNF-α: tumor necrosis factor alpha.

Supplementary Figure 92: Decorrelated residuals versus dose for the RCS (with three knots) model of whole grain intake on weight, waist circumference, body fat percentage, and adverse events**.** RCS: restricted cubic spline

Supplementary Figure 93: Decorrelated residuals versus dose for the RCS (with three knots) model of whole grain intake on FPG, HbA1c, insulin, and HOMA-IR. RCS: restricted cubic spline, FPG: fasting plasma glucose, HbA_1c_: glycated haemoglobin, HOMA-IR: homeostasis model assessment of insulin resistance.

Supplementary Figure 94: Decorrelated residuals versus dose for the RCS (with three knots) model of whole grain intake on triglycerides, total cholesterol, LDL-C, and HDL-C. RCS: restricted cubic spline, LDL-C: low-density lipoprotein cholesterol, HDL-C: high-density lipoprotein cholesterol.

Supplementary Figure 95: Decorrelated residuals versus dose for the RCS (with three knots) model of whole grain intake on SBP and DBP. RCS: restricted cubic spline, SBP: systolic blood pressure, DBP: diastolic blood pressure.

Supplementary Figure 96: Decorrelated residuals versus dose for the RCS (with three knots) model of whole grain intake on CRP, IL-6, and TNF-α. RCS: restricted cubic spline, CRP: C-reactive protein, IL-6: interleukin 6, TNF-α: tumor necrosis factor alpha.

Supplementary Figure 97: Decorrelated residuals versus dose for the RCS (with four knots) model of whole grain intake on weight, waist circumference, and body fat percentage. RCS: restricted cubic spline

Supplementary Figure 98: Decorrelated residuals versus dose for the RCS (with four knots) model of whole grain intake on FPG, HbA1c, insulin, and HOMA-IR. RCS: restricted cubic spline, FPG: fasting plasma glucose, HbA_1c_: glycated haemoglobin, HOMA-IR: homeostasis model assessment of insulin resistance.

Supplementary Figure 99: Decorrelated residuals versus dose for the RCS (with four knots) model of whole grain intake on triglycerides, total cholesterol, LDL-C, and HDL-C. RCS: restricted cubic spline, LDL-C: low-density lipoprotein cholesterol, HDL-C: high-density lipoprotein cholesterol.

Supplementary Figure 100: Decorrelated residuals versus dose for the RCS (with four knots) model of whole grain intake on SBP and DBP. RCS: restricted cubic spline, SBP: systolic blood pressure, DBP: diastolic blood pressure.

Supplementary Figure 101: Decorrelated residuals versus dose for the RCS (with four knots) model of whole grain intake on CRP, IL-6, and TNF-α. RCS: restricted cubic spline, CRP: C-reactive protein, IL-6: interleukin 6, TNF-α: tumor necrosis factor alpha.

**Supplementary References:**

1.	Deeks JJ, Higgins JP, Altman DG, Group obotCSM. Analysing data and undertaking meta-analyses. In. Cochrane Handbook for Systematic Reviews of Interventions; 2019, p241-284.

2.	Chi KY, Li MY, Chen C, Kang E. Ten circumstances and solutions for finding the sample mean and standard deviation for meta-analysis. *Syst Rev* 2023;**12**:62. doi: 10.1186/s13643-023-02217-1

3.	Higgins JP, White IR, Anzures-Cabrera J. Meta-analysis of skewed data: combining results reported on log-transformed or raw scales. *Stat Med* 2008;**27**:6072-6092. doi: 10.1002/sim.3427

4.	Altman DG, Bland JM. How to obtain the confidence interval from a P value. *Bmj* 2011;**343**:d2090. doi: 10.1136/bmj.d2090

5.	Higgins JP, Li T, Deeks JJ. Choosing effect measures and computing estimates of effect. In. Cochrane Handbook for Systematic Reviews of Interventions; 2019, p143-176.

6.	Furukawa TA, Barbui C, Cipriani A, Brambilla P, Watanabe N. Imputing missing standard deviations in meta-analyses can provide accurate results. *J Clin Epidemiol* 2006;**59**:7-10. doi: 10.1016/j.jclinepi.2005.06.006

7.	Elbourne DR, Altman DG, Higgins JP*, et al.* Meta-analyses involving cross-over trials: methodological issues. *Int J Epidemiol* 2002;**31**:140-149. doi: 10.1093/ije/31.1.140

8.	Follmann D, Elliott P, Suh I, Cutler J. Variance imputation for overviews of clinical trials with continuous response. *J Clin Epidemiol* 1992;**45**:769-773. doi: 10.1016/0895-4356(92)90054-q

9.	Balk EM, Earley A, Patel K, Trikalinos TA, Dahabreh IJ. Empirical assessment of within-arm correlation imputation in trials of continuous outcomes. 2013. doi:

10.	Higgins JP, Thomas J, Chandler J*, et al.* Cochrane handbook for systematic reviews of interventions: John Wiley & Sons; 2019.

11.	DerSimonian R, Laird N. Meta-analysis in clinical trials. *Control Clin Trials* 1986;**7**:177-188. doi: 10.1016/0197-2456(86)90046-2

12.	Knapp G, Hartung J. Improved tests for a random effects meta-regression with a single covariate. *Stat Med* 2003;**22**:2693-2710. doi: 10.1002/sim.1482

13.	Raudenbush SW. Analyzing effect sizes: Random-effects models. In. The handbook of research synthesis and meta-analysis, 2nd ed. New York, NY, US: Russell Sage Foundation; 2009, p295-315.

14.	Ioannidis JP, Patsopoulos NA, Evangelou E. Uncertainty in heterogeneity estimates in meta-analyses. *Bmj* 2007;**335**:914-916. doi: 10.1136/bmj.39343.408449.80

15.	Schandelmaier S, Briel M, Varadhan R*, et al.* Development of the Instrument to assess the Credibility of Effect Modification Analyses (ICEMAN) in randomized controlled trials and meta-analyses. *Cmaj* 2020;**192**:E901-e906. doi: 10.1503/cmaj.200077

16.	Peters JL, Sutton AJ, Jones DR, Abrams KR, Rushton L. Contour-enhanced meta-analysis funnel plots help distinguish publication bias from other causes of asymmetry. *J Clin Epidemiol* 2008;**61**:991-996. doi: 10.1016/j.jclinepi.2007.11.010

17.	Egger M, Davey Smith G, Schneider M, Minder C. Bias in meta-analysis detected by a simple, graphical test. *Bmj* 1997;**315**:629-634. doi: 10.1136/bmj.315.7109.629

18.	Sterne JA, Sutton AJ, Ioannidis JP*, et al.* Recommendations for examining and interpreting funnel plot asymmetry in meta-analyses of randomised controlled trials. *Bmj* 2011;**343**:d4002. doi: 10.1136/bmj.d4002

19.	Crippa A, Orsini N. Dose-response meta-analysis of differences in means. *BMC Med Res Methodol* 2016;**16**:91. doi: 10.1186/s12874-016-0189-0

20.	Ross AB, Kristensen M, Seal CJ, Jacques P, McKeown NM. Recommendations for reporting whole-grain intake in observational and intervention studies. *Am J Clin Nutr* 2015;**101**:903-907. doi: 10.3945/ajcn.114.098046

21.	Zhang G, Pan A, Zong G*, et al.* Substituting white rice with brown rice for 16 weeks does not substantially affect metabolic risk factors in middle-aged Chinese men and women with diabetes or a high risk for diabetes. *J Nutr* 2011;**141**:1685-1690. doi: 10.3945/jn.111.142224

22.	Kazemzadeh M, Safavi SM, Nematollahi S, Nourieh Z. Effect of Brown Rice Consumption on Inflammatory Marker and Cardiovascular Risk Factors among Overweight and Obese Non-menopausal Female Adults. *Int J Prev Med* 2014;**5**:478-488. doi:

23.	Araki R, Ushio R, Fujie K*, et al.* Effect of partially-abraded brown rice consumption on body weight and the indicators of glucose and lipid metabolism in pre-diabetic adults: A randomized controlled trial. *Clinical Nutrition ESPEN* 2017;**19**:9-15. doi: <https://doi.org/10.1016/j.clnesp.2017.01.015>

24.	Liao MY, Shen YC, Chiu HF*, et al.* Down-regulation of partial substitution for staple food by oat noodles on blood lipid levels: A randomized, double-blind, clinical trial. *J Food Drug Anal* 2019;**27**:93-100. doi: 10.1016/j.jfda.2018.04.001

25.	Tucker AJ, Mackay KA, Robinson LE*, et al.* The effect of whole grain wheat sourdough bread consumption on serum lipids in healthy normoglycemic/normoinsulinemic and hyperglycemic/hyperinsulinemic adults depends on presence of the APOE E3/E3 genotype: a randomized controlled trial. *Nutr Metab (Lond)* 2010;**7**:37. doi: 10.1186/1743-7075-7-37

26.	MacKay KA, Tucker AJ, Duncan AM, Graham TE, Robinson LE. Whole grain wheat sourdough bread does not affect plasminogen activator inhibitor-1 in adults with normal or impaired carbohydrate metabolism. *Nutr Metab Cardiovasc Dis* 2012;**22**:704-711. doi: 10.1016/j.numecd.2010.10.018

27.	Gilhooly CH, Das SK, Golden JK*, et al.* Use of cereal fiber to facilitate adherence to a human caloric restriction program. *Aging Clin Exp Res* 2008;**20**:513-520. doi: 10.1007/bf03324878

28.	Johnston L, Reynolds HR, Patz M*, et al.* Cholesterol-lowering benefits of a whole grain oat ready-to-eat cereal. *Nutr Clin Care* 1998;**1**:6-12. doi:

29.	Karmally W, Montez MG, Palmas W*, et al.* Cholesterol-lowering benefits of oat-containing cereal in Hispanic americans. *J Am Diet Assoc* 2005;**105**:967-970. doi: 10.1016/j.jada.2005.03.006

30.	Maki KC, Beiseigel JM, Jonnalagadda SS*, et al.* Whole-grain ready-to-eat oat cereal, as part of a dietary program for weight loss, reduces low-density lipoprotein cholesterol in adults with overweight and obesity more than a dietary program including low-fiber control foods. *J Am Diet Assoc* 2010;**110**:205-214. doi: 10.1016/j.jada.2009.10.037

31.	Reynolds HR, Quiter E, Hunninghake DB. Whole Grain Oat Cereal Lowers Serum Lipids. *Topics in Clinical Nutrition* 2000;**15**:74-83. doi:

32.	Li J, Kaneko T, Qin LQ, Wang J, Wang Y. Effects of barley intake on glucose tolerance, lipid metabolism, and bowel function in women. *Nutrition* 2003;**19**:926-929. doi: 10.1016/s0899-9007(03)00182-5

33.	U.S. Department of Agriculture and U.S. Department of Health and Human Services. Dietary Guidelines for Americans, 2020-2025. 9th Edition. December 2020. Available at DietaryGuidelines.gov. doi:

34.	Naghshi S, Kiani S, Ostadrahimi A*, et al.* Effect of whole grain consumption on cardiometabolic risk factors: a protocol for a systematic review and dose-response meta-analysis of randomised controlled trials. PROSPERO 2024 Available from <https://www.crd.york.ac.uk/PROSPERO/view/CRD42024565726>. doi:

35.	Guyatt GH, Oxman AD, Vist G*, et al.* GRADE guidelines: 4. Rating the quality of evidence—study limitations (risk of bias). *Journal of clinical epidemiology* 2011;**64**:407-415. doi:

36.	Guyatt GH, Oxman AD, Kunz R*, et al.* GRADE guidelines: 7. Rating the quality of evidence—inconsistency. *Journal of clinical epidemiology* 2011;**64**:1294-1302. doi:

37.	Guyatt GH, Oxman AD, Kunz R*, et al.* GRADE guidelines: 8. Rating the quality of evidence—indirectness. *Journal of clinical epidemiology* 2011;**64**:1303-1310. doi:

38.	Guyatt GH, Oxman AD, Kunz R*, et al.* GRADE guidelines 6. Rating the quality of evidence--imprecision. *J Clin Epidemiol* 2011;**64**:1283-1293. doi: 10.1016/j.jclinepi.2011.01.012

39.	Guyatt GH, Oxman AD, Montori V*, et al.* GRADE guidelines: 5. Rating the quality of evidence--publication bias. *J Clin Epidemiol* 2011;**64**:1277-1282. doi: 10.1016/j.jclinepi.2011.01.011

40.	Xu D, Feng M, Chu Y*, et al.* The Prebiotic Effects of Oats on Blood Lipids, Gut Microbiota, and Short-Chain Fatty Acids in Mildly Hypercholesterolemic Subjects Compared With Rice: A Randomized, Controlled Trial. *Front Immunol* 2021;**12**:787797. doi: 10.3389/fimmu.2021.787797

41.	Xu D, Wang S, Feng M*, et al.* Serum Metabolomics Reveals Underlying Mechanisms of Cholesterol-Lowering Effects of Oat Consumption: A Randomized Controlled Trial in a Mildly Hypercholesterolemic Population. *Mol Nutr Food Res* 2021;**65**:e2001059. doi: 10.1002/mnfr.202001059

42.	Wan W, Jiang X, Zhao R*, et al.* Branched Chain Amino Acids, New Target of Germinated Brown Rice against Type 2 Diabetes Mellitus: A Randomized Controlled Trial. *Mol Nutr Food Res* 2023;**67**:e2200481. doi: 10.1002/mnfr.202200481

43.	Ding Q, Ren J, Zhou Y*, et al.* Whole grain germinated brown rice regulates intestinal immune homeostasis and gastrointestinal hormones in type 2 diabetic patients-a randomized control trial. *Food Funct* 2022;**13**:8274-8282. doi: 10.1039/d2fo00477a

44.	de Mello VD, Schwab U, Kolehmainen M*, et al.* A diet high in fatty fish, bilberries and wholegrain products improves markers of endothelial function and inflammation in individuals with impaired glucose metabolism in a randomised controlled trial: the Sysdimet study. *Diabetologia* 2011;**54**:2755-2767. doi: 10.1007/s00125-011-2285-3

45.	Lankinen M, Kolehmainen M, Jääskeläinen T*, et al.* Effects of whole grain, fish and bilberries on serum metabolic profile and lipid transfer protein activities: a randomized trial (Sysdimet). *PLoS One* 2014;**9**:e90352. doi: 10.1371/journal.pone.0090352

46.	Schutte S, Esser D, Hoevenaars FPM*, et al.* A 12-wk whole-grain wheat intervention protects against hepatic fat: the Graandioos study, a randomized trial in overweight subjects. *Am J Clin Nutr* 2018;**108**:1264-1274. doi: 10.1093/ajcn/nqy204

47.	Hoevenaars FPM, Esser D, Schutte S*, et al.* Whole Grain Wheat Consumption Affects Postprandial Inflammatory Response in a Randomized Controlled Trial in Overweight and Obese Adults with Mild Hypercholesterolemia in the Graandioos Study. *J Nutr* 2019;**149**:2133-2144. doi: 10.1093/jn/nxz177

48.	Giacco R, Lappi J, Costabile G*, et al.* Effects of rye and whole wheat versus refined cereal foods on metabolic risk factors: a randomised controlled two-centre intervention study. *Clin Nutr* 2013;**32**:941-949. doi: 10.1016/j.clnu.2013.01.016

49.	Giacco R, Costabile G, Della Pepa G*, et al.* A whole-grain cereal-based diet lowers postprandial plasma insulin and triglyceride levels in individuals with metabolic syndrome. *Nutr Metab Cardiovasc Dis* 2014;**24**:837-844. doi: 10.1016/j.numecd.2014.01.007

50.	Vanegas SM, Meydani M, Barnett JB*, et al.* Substituting whole grains for refined grains in a 6-wk randomized trial has a modest effect on gut microbiota and immune and inflammatory markers of healthy adults. *Am J Clin Nutr* 2017;**105**:635-650. doi: 10.3945/ajcn.116.146928

51.	Ma X, Gu J, Zhang Z*, et al.* Effects of Avena nuda L. on metabolic control and cardiovascular disease risk among Chinese patients with diabetes and meeting metabolic syndrome criteria: secondary analysis of a randomized clinical trial. *Eur J Clin Nutr* 2013;**67**:1291-1297. doi: 10.1038/ejcn.2013.201

52.	Li X, Cai X, Ma X*, et al.* Short- and Long-Term Effects of Wholegrain Oat Intake on Weight Management and Glucolipid Metabolism in Overweight Type-2 Diabetics: A Randomized Control Trial. *Nutrients* 2016;**8**. doi: 10.3390/nu8090549

53.	Karl JP, Meydani M, Barnett JB*, et al.* Substituting whole grains for refined grains in a 6-wk randomized trial favorably affects energy-balance metrics in healthy men and postmenopausal women. *Am J Clin Nutr* 2017;**105**:589-599. doi: 10.3945/ajcn.116.139683

54.	Lankinen M, Schwab U, Kolehmainen M*, et al.* Whole grain products, fish and bilberries alter glucose and lipid metabolism in a randomized, controlled trial: the Sysdimet study. *PLoS One* 2011;**6**:e22646. doi: 10.1371/journal.pone.0022646

55.	Xue K, Liu Y, Iversen KN*, et al.* Impact of a Fermented High-Fiber Rye Diet on Helicobacter pylori and Cardio-Metabolic Risk Factors: A Randomized Controlled Trial Among Helicobacter pylori-Positive Chinese Adults. *Front Nutr* 2020;**7**:608623. doi: 10.3389/fnut.2020.608623

56.	Juntunen KS, Laaksonen DE, Poutanen KS, Niskanen LK, Mykkänen HM. High-fiber rye bread and insulin secretion and sensitivity in healthy postmenopausal women. *Am J Clin Nutr* 2003;**77**:385-391. doi: 10.1093/ajcn/77.2.385

57.	Moazzami AA, Bondia-Pons I, Hanhineva K*, et al.* Metabolomics reveals the metabolic shifts following an intervention with rye bread in postmenopausal women--a randomized control trial. *Nutr J* 2012;**11**:88. doi: 10.1186/1475-2891-11-88

58.	Ampatzoglou A, Atwal KK, Maidens CM*, et al.* Increased whole grain consumption does not affect blood biochemistry, body composition, or gut microbiology in healthy, low-habitual whole grain consumers. *J Nutr* 2015;**145**:215-221. doi: 10.3945/jn.114.202176

59.	Ampatzoglou A, Williams CL, Atwal KK*, et al.* Effects of increased wholegrain consumption on immune and inflammatory markers in healthy low habitual wholegrain consumers. *Eur J Nutr* 2016;**55**:183-195. doi: 10.1007/s00394-015-0836-y

60.	Andersson A, Tengblad S, Karlström B*, et al.* Whole-grain foods do not affect insulin sensitivity or markers of lipid peroxidation and inflammation in healthy, moderately overweight subjects. *J Nutr* 2007;**137**:1401-1407. doi: 10.1093/jn/137.6.1401

61.	Aoe S, Nakamura F, Fujiwara S. Effect of Wheat Bran on Fecal Butyrate-Producing Bacteria and Wheat Bran Combined with Barley on Bacteroides Abundance in Japanese Healthy Adults. *Nutrients* 2018;**10**. doi: 10.3390/nu10121980

62.	Brownlee IA, Moore C, Chatfield M*, et al.* Markers of cardiovascular risk are not changed by increased whole-grain intake: the WHOLEheart study, a randomised, controlled dietary intervention. *Br J Nutr* 2010;**104**:125-134. doi: 10.1017/s0007114510000644

63.	Akamine Y, Millman JF, Uema T*, et al.* Fermented brown rice beverage distinctively modulates the gut microbiota in Okinawans with metabolic syndrome: A randomized controlled trial. *Nutr Res* 2022;**103**:68-81. doi: 10.1016/j.nutres.2022.03.013

64.	Cooper DN, Kable ME, Marco ML*, et al.* The Effects of Moderate Whole Grain Consumption on Fasting Glucose and Lipids, Gastrointestinal Symptoms, and Microbiota. *Nutrients* 2017;**9**. doi: 10.3390/nu9020173

65.	Dorosti M, Jafary Heidarloo A, Bakhshimoghaddam F, Alizadeh M. Whole-grain consumption and its effects on hepatic steatosis and liver enzymes in patients with non-alcoholic fatty liver disease: a randomised controlled clinical trial. *Br J Nutr* 2020;**123**:328-336. doi: 10.1017/s0007114519002769

66.	Durazzo A, Carcea M, Adlercreutz H*, et al.* Effects of consumption of whole grain foods rich in lignans in healthy postmenopausal women with moderate serum cholesterol: a pilot study. *Int J Food Sci Nutr* 2014;**65**:637-645. doi: 10.3109/09637486.2014.893283

67.	Esmaeilpour M, Ghasemian S, Alizadeh M. Diets enriched with whole grains reduce premenstrual syndrome scores in nurses: an open-label parallel randomised controlled trial. *Br J Nutr* 2019;**121**:992-1001. doi: 10.1017/s0007114519000333

68.	Geng S, Zhu Y, Zhang J, Cai Y. Original article lowering effect of pre-germinated brown rice on dyslipidemia in Chinese patients. 2016;**9**:22317-22323. doi:

69.	Gulati S, Misra A, Pandey RM. Effects of 3 g of soluble fiber from oats on lipid levels of Asian Indians - a randomized controlled, parallel arm study. *Lipids Health Dis* 2017;**16**:71. doi: 10.1186/s12944-017-0460-3

70.	Iversen KN, Carlsson F, Andersson A*, et al.* A hypocaloric diet rich in high fiber rye foods causes greater reduction in body weight and body fat than a diet rich in refined wheat: A parallel randomized controlled trial in adults with overweight and obesity (the RyeWeight study). *Clin Nutr ESPEN* 2021;**45**:155-169. doi: 10.1016/j.clnesp.2021.07.007

71.	Katcher HI, Legro RS, Kunselman AR*, et al.* The effects of a whole grain-enriched hypocaloric diet on cardiovascular disease risk factors in men and women with metabolic syndrome. *Am J Clin Nutr* 2008;**87**:79-90. doi: 10.1093/ajcn/87.1.79

72.	Keenan JM, Pins JJ, Frazel C, Moran A, Turnquist L. Oat ingestion reduces systolic and diastolic blood pressure in patients with mild or borderline hypertension: a pilot trial. *J Fam Pract* 2002;**51**:369. doi:

73.	Kikuchi Y, Nozaki S, Makita M*, et al.* Effects of Whole Grain Wheat Bread on Visceral Fat Obesity in Japanese Subjects: A Randomized Double-Blind Study. *Plant Foods Hum Nutr* 2018;**73**:161-165. doi: 10.1007/s11130-018-0666-1

74.	Kim JY, Kim JH, Lee DH, Kim SH, Lee SS. Meal replacement with mixed rice is more effective than white rice in weight control, while improving antioxidant enzyme activity in obese women. *Nutr Res* 2008;**28**:66-71. doi: 10.1016/j.nutres.2007.12.006

75.	Kondo K, Morino K, Nishio Y*, et al.* Fiber-rich diet with brown rice improves endothelial function in type 2 diabetes mellitus: A randomized controlled trial. *PLoS One* 2017;**12**:e0179869. doi: 10.1371/journal.pone.0179869

76.	Kopf JC, Suhr MJ, Clarke J*, et al.* Role of whole grains versus fruits and vegetables in reducing subclinical inflammation and promoting gastrointestinal health in individuals affected by overweight and obesity: a randomized controlled trial. *Nutr J* 2018;**17**:72. doi: 10.1186/s12937-018-0381-7

77.	Kristensen M, Pelletier X, Ross AB, Thielecke F. A High Rate of Non-Compliance Confounds the Study of Whole Grains and Weight Maintenance in a Randomised Intervention Trial-The Case for Greater Use of Dietary Biomarkers in Nutrition Intervention Studies. *Nutrients* 2017;**9**. doi: 10.3390/nu9010055

78.	Kristensen M, Toubro S, Jensen MG*, et al.* Whole grain compared with refined wheat decreases the percentage of body fat following a 12-week, energy-restricted dietary intervention in postmenopausal women. *J Nutr* 2012;**142**:710-716. doi: 10.3945/jn.111.142315

79.	Li L, Lietz G, Bal W*, et al.* Effects of Quinoa (Chenopodium quinoa Willd.) Consumption on Markers of CVD Risk. *Nutrients* 2018;**10**. doi: 10.3390/nu10060777

80.	Mai TT, Trang TT, Hai TT. Effectiveness of germinated brown rice on metabolic syndrome: A randomized control trial in Vietnam. *AIMS Public Health* 2020;**7**:33-43. doi: 10.3934/publichealth.2020005

81.	Malik VS, Sudha V, Wedick NM*, et al.* Substituting brown rice for white rice on diabetes risk factors in India: a randomised controlled trial. *Br J Nutr* 2019;**121**:1389-1397. doi: 10.1017/s000711451900076x

82.	Meng H, Matthan NR, Fried SK*, et al.* Effect of Dietary Carbohydrate Type on Serum Cardiometabolic Risk Indicators and Adipose Tissue Inflammatory Markers. *J Clin Endocrinol Metab* 2018;**103**:3430-3438. doi: 10.1210/jc.2018-00667

83.	Na G, Zhang J, Lv D*, et al.* Germinated Brown rice enhanced n-3 PUFA metabolism in type 2 diabetes patients: A randomized controlled trial. *Clin Nutr* 2023;**42**:579-589. doi: 10.1016/j.clnu.2023.02.001

84.	Nakayama T, Nagai Y, Uehara Y*, et al.* Eating glutinous brown rice twice a day for 8 weeks improves glycemic control in Japanese patients with diabetes mellitus. *Nutr Diabetes* 2017;**7**:e273. doi: 10.1038/nutd.2017.26

85.	Nakayama T, Nagai Y, Yasunaka Y*, et al.* Effect of Eating Glutinous Brown Rice Twice a Day for 6 Weeks on Serum 1,5-Anhydroglucitol in Japanese Subjects without Diabetes. *J Nutr Metab* 2020;**2020**:8847781. doi: 10.1155/2020/8847781

86.	Kirwan JP, Malin SK, Scelsi AR*, et al.* A Whole-Grain Diet Reduces Cardiovascular Risk Factors in Overweight and Obese Adults: A Randomized Controlled Trial. *J Nutr* 2016;**146**:2244-2251. doi: 10.3945/jn.116.230508

87.	Navarro-Perez D, Radcliffe J, Tierney A, Jois M. Quinoa Seed Lowers Serum Triglycerides in Overweight and Obese Subjects: A Dose-Response Randomized Controlled Clinical Trial. *Curr Dev Nutr* 2017;**1**:e001321. doi: 10.3945/cdn.117.001321

88.	Nazari J, Yadegari N, Khodam S, Almasi-Hashian A, Amini S. Effect of Consumption of Whole-Wheat Breads on FBS, HbA1c, and Blood Lipids in Patients with Type 2 Diabetes. *Prev Nutr Food Sci* 2021;**26**:269-274. doi: 10.3746/pnf.2021.26.3.269

89.	Pereira MA, Jacobs DR, Jr., Pins JJ*, et al.* Effect of whole grains on insulin sensitivity in overweight hyperinsulinemic adults. *Am J Clin Nutr* 2002;**75**:848-855. doi: 10.1093/ajcn/75.5.848

90.	Pushpass RG, Alzoufairi S, Mancini A*, et al.* Chronic consumption of probiotics, oats, and apples has differential effects on postprandial bile acid profile and cardiometabolic disease risk markers compared with an isocaloric control (cornflakes): a randomized trial. *Am J Clin Nutr* 2023;**117**:252-265. doi: 10.1016/j.ajcnut.2022.10.013

91.	Qiu J, Liu Y, Yue Y, Qin Y, Li Z. Dietary tartary buckwheat intake attenuates insulin resistance and improves lipid profiles in patients with type 2 diabetes: a randomized controlled trial. *Nutr Res* 2016;**36**:1392-1401. doi: 10.1016/j.nutres.2016.11.007

92.	Reiners S, Hebestreit S, Wedekind L*, et al.* Effect of a regular consumption of traditional and roasted oat and barley flakes on blood lipids and glucose metabolism-A randomized crossover trial. *Front Nutr* 2023;**10**:1095245. doi: 10.3389/fnut.2023.1095245

93.	Ross AB, Bruce SJ, Blondel-Lubrano A*, et al.* A whole-grain cereal-rich diet increases plasma betaine, and tends to decrease total and LDL-cholesterol compared with a refined-grain diet in healthy subjects. *Br J Nutr* 2011;**105**:1492-1502. doi: 10.1017/s0007114510005209

94.	Shimabukuro M, Higa M, Kinjo R*, et al.* Effects of the brown rice diet on visceral obesity and endothelial function: the BRAVO study. *Br J Nutr* 2014;**111**:310-320. doi: 10.1017/s0007114513002432

95.	Thongoun P, Pavadhgul P, Bumrungpert A*, et al.* Effect of oat consumption on lipid profiles in hypercholesterolemic adults. *J Med Assoc Thai* 2013;**96 Suppl 5**:S25-32. doi:

96.	Tighe P, Duthie G, Vaughan N*, et al.* Effect of increased consumption of whole-grain foods on blood pressure and other cardiovascular risk markers in healthy middle-aged persons: a randomized controlled trial. *Am J Clin Nutr* 2010;**92**:733-740. doi: 10.3945/ajcn.2010.29417

97.	Van Horn L, Emidy LA, Liu KA*, et al.* Serum lipid response to a fat-modified, oatmeal-enhanced diet. *Prev Med* 1988;**17**:377-386. doi: 10.1016/0091-7435(88)90012-6

98.	Van Horn L, Moag-Stahlberg A, Liu KA*, et al.* Effects on serum lipids of adding instant oats to usual American diets. *Am J Public Health* 1991;**81**:183-188. doi: 10.2105/ajph.81.2.183

99.	Vitaglione P, Mennella I, Ferracane R*, et al.* Whole-grain wheat consumption reduces inflammation in a randomized controlled trial on overweight and obese subjects with unhealthy dietary and lifestyle behaviors: role of polyphenols bound to cereal dietary fiber. *Am J Clin Nutr* 2015;**101**:251-261. doi: 10.3945/ajcn.114.088120

100.	Wang B, Medapalli R, Xu J*, et al.* Effects of a whole rice diet on metabolic parameters and inflammatory markers in prediabetes. *e-SPEN Journal* 2013;**8**:e15-e20. doi: <https://doi.org/10.1016/j.clnme.2012.11.001>

101.	Zhang J, Li L, Song P*, et al.* Randomized controlled trial of oatmeal consumption versus noodle consumption on blood lipids of urban Chinese adults with hypercholesterolemia. *Nutr J* 2012;**11**:54. doi: 10.1186/1475-2891-11-54

102.	Harris Jackson K, West SG, Vanden Heuvel JP*, et al.* Effects of whole and refined grains in a weight-loss diet on markers of metabolic syndrome in individuals with increased waist circumference: a randomized controlled-feeding trial. *Am J Clin Nutr* 2014;**100**:577-586. doi: 10.3945/ajcn.113.078048

103.	Saltzman E, Das SK, Lichtenstein AH*, et al.* An oat-containing hypocaloric diet reduces systolic blood pressure and improves lipid profile beyond effects of weight loss in men and women. *J Nutr* 2001;**131**:1465-1470. doi: 10.1093/jn/131.5.1465

104.	Bodinham CL, Hitchen KL, Youngman PJ, Frost GS, Robertson MD. Short-term effects of whole-grain wheat on appetite and food intake in healthy adults: a pilot study. *Br J Nutr* 2011;**106**:327-330. doi: 10.1017/s0007114511000225

105.	Hsu TF, Kise M, Wang MF*, et al.* Effects of pre-germinated brown rice on blood glucose and lipid levels in free-living patients with impaired fasting glucose or type 2 diabetes. *J Nutr Sci Vitaminol (Tokyo)* 2008;**54**:163-168. doi: 10.3177/jnsv.54.163

106.	Roager HM, Vogt JK, Kristensen M*, et al.* Whole grain-rich diet reduces body weight and systemic low-grade inflammation without inducing major changes of the gut microbiome: a randomised cross-over trial. *Gut* 2019;**68**:83-93. doi: 10.1136/gutjnl-2017-314786

107.	Maier SM, Turner ND, Lupton JR. Serum Lipids in Hypercholesterolemic Men and Women Consuming Oat Bran and Amaranth Products. *Cereal Chemistry* 2000;**77**:297-302. doi: <https://doi.org/10.1094/CCHEM.2000.77.3.297>

108.	Melanson KJ, Angelopoulos TJ, Nguyen VT*, et al.* Consumption of whole-grain cereals during weight loss: effects on dietary quality, dietary fiber, magnesium, vitamin B-6, and obesity. *J Am Diet Assoc* 2006;**106**:1380-1388; quiz 1389-1390. doi: 10.1016/j.jada.2006.06.003

109.	Davidson MH, Dugan LD, Burns JH*, et al.* The hypocholesterolemic effects of beta-glucan in oatmeal and oat bran. A dose-controlled study. *Jama* 1991;**265**:1833-1839. doi:

110.	Connolly ML, Tzounis X, Tuohy KM, Lovegrove JA. Hypocholesterolemic and Prebiotic Effects of a Whole-Grain Oat-Based Granola Breakfast Cereal in a Cardio-Metabolic "At Risk" Population. *Front Microbiol* 2016;**7**:1675. doi: 10.3389/fmicb.2016.01675

111.	Charlton KE, Tapsell LC, Batterham MJ*, et al.* Effect of 6 weeks' consumption of β-glucan-rich oat products on cholesterol levels in mildly hypercholesterolaemic overweight adults. *Br J Nutr* 2012;**107**:1037-1047. doi: 10.1017/s0007114511003850

112.	Kuroda Y, Matsuzaki K, Wakatsuki H*, et al.* Influence of Ultra-High Hydrostatic Pressurizing Brown Rice on Cognitive Functions and Mental Health of Elderly Japanese Individuals: A 2-Year Randomized and Controlled Trial. *J Nutr Sci Vitaminol (Tokyo)* 2019;**65**:S80-s87. doi: 10.3177/jnsv.65.S80

113.	Leinonen KS, Poutanen KS, Mykkänen HM. Rye bread decreases serum total and LDL cholesterol in men with moderately elevated serum cholesterol. *J Nutr* 2000;**130**:164-170. doi: 10.1093/jn/130.2.164

114.	Ren C, Hong B, Zheng X*, et al.* Improvement of germinated brown rice quality with autoclaving treatment. *Food Sci Nutr* 2020;**8**:1709-1717. doi: 10.1002/fsn3.1459

115.	Joo NS, Han SI, Kim KN*, et al.* Black rice with giant embryo ameliorates serum C-reactive protein in adults with metabolic syndrome. *J Clin Biochem Nutr* 2020;**67**:344-348. doi: 10.3164/jcbn.20-72

116.	Laborde GMR, Estrada MR, Romero MV. Brown rice consumption and changes in the metabolic risk factors of non-communicable diseases in selected overweight and obese filipinos. *Philippine Journal of Science* 2021;**150**:473-484. doi:

117.	Pavadhgul P, Bumrungpert A, Harjani Y, Kurilich A. Oat porridge consumption alleviates markers of inflammation and oxidative stress in hypercholesterolemic adults. *Asia Pac J Clin Nutr* 2019;**28**:260-265. doi: 10.6133/apjcn.201906_28(2).0008

118.	Chang HC, Huang CN, Yeh DM*, et al.* Oat prevents obesity and abdominal fat distribution, and improves liver function in humans. *Plant Foods Hum Nutr* 2013;**68**:18-23. doi: 10.1007/s11130-013-0336-2

119.	Van Horn LV, Liu K, Parker D*, et al.* Serum lipid response to oat product intake with a fat-modified diet. *J Am Diet Assoc* 1986;**86**:759-764. doi:

120.	Zeng H, Cai X, Qiu Z, Liang Y, Huang L. Glucolipid metabolism improvement in impaired glucose tolerance subjects consuming a Quinoa-based diet: a randomized parallel clinical trial. *Front Physiol* 2023;**14**:1179587. doi: 10.3389/fphys.2023.1179587

121.	De Carvalho FG, Ovídio PP, Padovan GJ*, et al.* Metabolic parameters of postmenopausal women after quinoa or corn flakes intake--a prospective and double-blind study. *Int J Food Sci Nutr* 2014;**65**:380-385. doi: 10.3109/09637486.2013.866637

122.	Qiu J, Li Z, Qin Y, Yue Y, Liu Y. Protective effect of tartary buckwheat on renal function in type 2 diabetics: a randomized controlled trial. *Ther Clin Risk Manag* 2016;**12**:1721-1727. doi: 10.2147/tcrm.s123008

123.	Peräaho M, Kaukinen K, Mustalahti K*, et al.* Effect of an oats-containing gluten-free diet on symptoms and quality of life in coeliac disease. A randomized study. *Scand J Gastroenterol* 2004;**39**:27-31. doi: 10.1080/00365520310007783

124.	Ye M, Sun J, Chen Y*, et al.* Oatmeal induced gut microbiota alteration and its relationship with improved lipid profiles: a secondary analysis of a randomized clinical trial. *Nutr Metab (Lond)* 2020;**17**:85. doi: 10.1186/s12986-020-00505-4

125.	Liedike B, Khatib M, Tabarsi B*, et al.* Evaluating the Effects of Corn Flour Product Consumption on Cardiometabolic Outcomes and the Gut Microbiota in Adults with Elevated Cholesterol: A Randomized Crossover. *J Nutr* 2024;**154**:2437-2447. doi: 10.1016/j.tjnut.2024.06.003

126.	Li C, Li Y, Wang N*, et al.* Comprehensive modulatory effects of whole grain consumption on immune-mediated inflammation in middle-aged and elderly community residents: A real-world randomized controlled trial. *Redox Biol* 2024;**76**:103337. doi: 10.1016/j.redox.2024.103337

127.	Khoramnazari R, Salehi M, Kojuri J. The Effect of Whole Wheat and White Breads on Serum Lipid Profile, Malondialdehyde, and C-Reactive Protein in Over-Weight and Obese Patients with Coronary Stent. *International Journal of Nutrition Sciences* 2017;**2**:203-208. doi:

128.	Suhr J, Vuholm S, Iversen KN, Landberg R, Kristensen M. Wholegrain rye, but not wholegrain wheat, lowers body weight and fat mass compared with refined wheat: a 6-week randomized study. *Eur J Clin Nutr* 2017;**71**:959-967. doi: 10.1038/ejcn.2017.12

129.	Knopp JL, Holder-Pearson L, Chase JG. Insulin Units and Conversion Factors: A Story of Truth, Boots, and Faster Half-Truths. *J Diabetes Sci Technol* 2019;**13**:597-600. doi: 10.1177/1932296818805074

130.	Ge L, Sadeghirad B, Ball GD*, et al.* Comparison of dietary macronutrient patterns of 14 popular named dietary programmes for weight and cardiovascular risk factor reduction in adults: systematic review and network meta-analysis of randomised trials. *bmj* 2020;**369**. doi:

131.	Jovanovski E, Mazhar N, Komishon A*, et al.* Effect of viscous fiber supplementation on obesity indicators in individuals consuming calorie-restricted diets: a systematic review and meta-analysis of randomized controlled trials. *European journal of nutrition* 2020:1-12. doi:

132.	Goldenberg JZ, Day A, Brinkworth GD*, et al.* Efficacy and safety of low and very low carbohydrate diets for type 2 diabetes remission: systematic review and meta-analysis of published and unpublished randomized trial data. *bmj* 2021;**372**. doi:

133.	Norman GR, Sloan JA, Wyrwich KW. Interpretation of changes in health-related quality of life: the remarkable universality of half a standard deviation. *Medical care* 2003:582-592. doi:
